# Primary health care quality indicators: An umbrella review

**DOI:** 10.1371/journal.pone.0220888

**Published:** 2019-08-16

**Authors:** André Ramalho, Pedro Castro, Manuel Gonçalves-Pinho, Juliana Teixeira, João Vasco Santos, João Viana, Mariana Lobo, Paulo Santos, Alberto Freitas

**Affiliations:** 1 MEDCIDS–Department of Community Medicine, Information and Health Decision Sciences, Faculty of Medicine, University of Porto, Porto, Portugal; 2 CINTESIS–Centre for Health Technology and Services Research, Porto, Portugal; 3 USF Camélias, ACeS Grande Porto VII (ARS Norte)–Vila Nova de Gaia, Portugal; 4 Public Health Unit, ACeS Grande Porto VIII (ARS Norte)–Espinho/Gaia, Portugal; University of Mississippi Medical Center, UNITED STATES

## Abstract

Nowadays, evaluating the quality of health services, especially in primary health care (PHC), is increasingly important. In a historical perspective, the Department of Health (United Kingdom) developed and proposed a range of indicators in 1998, and lately several health, social and political organizations have defined and implemented different sets of PHC quality indicators. Some systematic reviews in PHC quality indicators are reported but only in specific contexts and conditions. The aim of this study is to characterize and provide a list of indicators discussed in the literature to support managers and clinicians in decision-making processes, through an umbrella review on PHC quality indicators. The methodology was performed according to PRISMA Statement. Indicators from 33 eligible systematic reviews were categorized according to the dimensions of care, function, type of care, domains and condition contexts. Of a total of 727 indicators or groups of indicators, 74.5% (n = 542) were classified in process category and 89.5% (n = 537) with chronic type of care (n = 428; 58.8%) and effective domain (n = 423; 58.1%) with the most frequent values in categorizations by dimensions. The results of this overview of reviews are valuable and imply the need for future research and practice regarding primary health care quality indicators in the most varied conditions and contexts to generate new discussions about their use, comparison and implementation.

## Introduction

Primary health care (PHC) is defined by the World Health Organization (WHO) as the “essential health care based on scientifically sound and socially acceptable methods and technology, which make universal health care accessible to all individuals and families in a community. It is through their full participation and at a cost that the community and the country can afford to maintain at every stage of their development in the spirit of self-reliance and self-determination" [1]. Some studies suggest that health systems with better financial and clinical results are those with a greater focus on PHC, thus enhancing the sustainability of the entire health system [2–4]. This depends on providing high quality primary health care, hence raising the need to develop methods for quality assessment and monitoring [5]. One of these methods is the use of quality indicators—a quantitative measure of the activities, that can assist as a guideline for quality monitoring and evaluation of relevant patient care and support services [6,7,8].

Quality of care was defined by the Institute of Medicine (IOM) in 1999 as the degree to which health services increase the likelihood of desired outcomes and are consistent with current professional knowledge [9]. The evaluation of the degree of quality of care is done through indicators, a set of measures that assist health care quality monitoring and evaluation in several areas, such as governance, management, assistance and support [10,11]. The importance of indicators is given by the fact that they allow for signalling opportunities of improvement, and controlling compliance with the best existing clinical practices, through quantitative parameters (planning, organizational, clinical) aiming better processes and outcomes [12,13].

Studies of how quality can be assessed were conducted by Donabedian and Fleming, who categorized the information from which inferences can be drawn on the topic, in three categories: structure, process and outcome [14]. The “three-part” assessment approach performed by the authors is only possible because a good structure increases the probability of a good health care processes, and good processes increase the probability of good outcomes [14]. Importantly, for a process to be a valid measure of quality, it must be closely related to a result that people care about [12]. It is also worth remembering that we often find factors that interfere with patients' survival and health-disease dynamics, and in these cases, it may be useful for outcome measures to be adjusted for other factors (such as lifestyle, disease) to control confounders that may affect the analysis of outcome indicators [10]. The development and selection of indicators must meet requirements for use, such as validity, reliability, relevance, pertinence, applicability, data availability, minimum bias, and moreover based on the best evidence available [15,16].

For historical contextualization only, the National Health Service Executive and the Department of Health in United Kingdom (UK)—pioneers in this area—proposed a range of indicators in 1998, many of which would apply to primary health care groups [17]. The interest in assessing the quality of primary health care services has increased, especially after 2004, when the Quality and Outcomes Framework (QOF) was introduced in the UK [18–20]. After the development of the QOF, some pay-for-performance systems have been developed over the years. These were based on the concept of allocative efficiency: “the optimal use of resources to achieve the intended outcomes” [21]. As such, financial incentive schemes are being used for PHC units worldwide and professionals, representing a way of rewarding improvements in productivity and/or adaptation to better quality healthcare provision [22].

Lately, several health, social and political organizations such as World Health Organization (WHO), Organization for Economic Cooperation and Development (OECD), European Commission and the Agency for Research and Quality of Health Care (AHRQ), have defined and implemented different sets of quality indicators for primary care [23–25]. There are several studies proposing PHC quality indicators in different countries, which have led to some systematic reviews revealing substantial geographical variability regarding quality of primary care services [26]. Identifying papers referring to PHC quality assessment projects, these systematic reviews reported that the number and content of indicators and their domains varied among studies. Moreover, they demonstrated that the lack of standardization of collection tools across projects would lead to invalid comparisons [27–31].

Considering the importance of understanding the PHC context, identifying and measuring quality indicators are essential factors for the achievement of high-quality care [32]. Some systematic reviews related to the topic are available in the literature but with focus on specific contexts, making it necessary to synthesize and understand the reality of these indicators in a broader scope. The aim of this umbrella review is not an exercise for a meta-review, but rather to identify systematic reviews of studies on quality indicators (QI) for PHC to provide a list of selected indicators considered in systematic reviews.

## Methods

**Fig 1:**
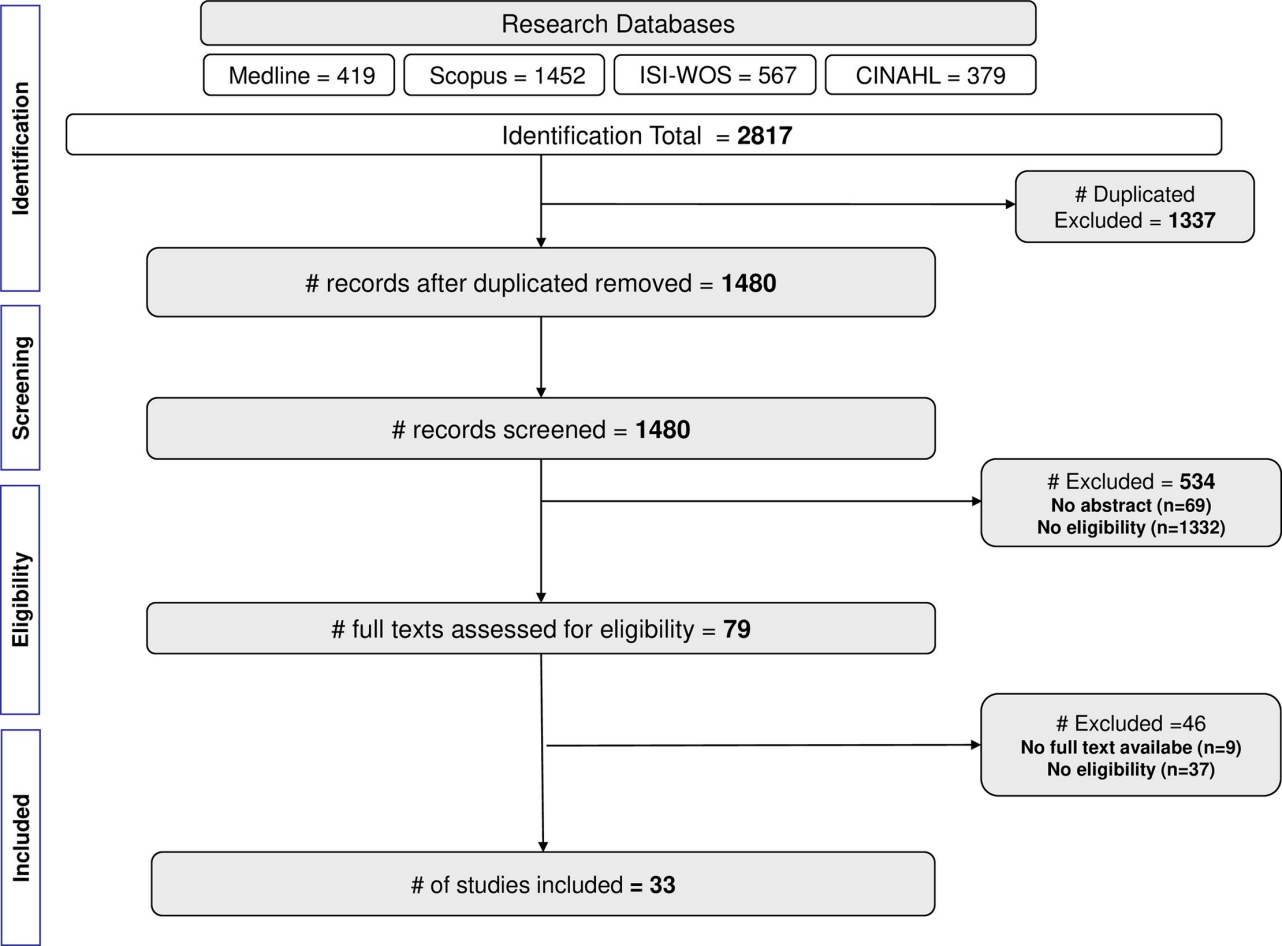
PRISMA flowchart.

An umbrella review was conducted, to collect and extract data from all systematic review studies uncovering PHC quality indicators. The methodology was performed according to PRISMA Statement [33] (**Fig 1** and **S1 File**). All the phases were performed by two independent reviewers with a third as a tie-breaker, considering the eligibility criteria. Composing PICO, participants were the primary care systems and the intervention to be analysed is the implementation of quality indicators. The comparator was the categories such as context, dimension, type and domain of care, and the main outcome was the primary health care quality indicators to present a summary list of the indicators used in PHC. The protocol was registered at PROSPERO [34,35] with number CRD42019124170 (**S2 File**).

### Search strategy

#### Identification phase

From an initial set of studies, a search expression was defined and calibrated [36,37] through test rounds for each and combined terms in electronic databases. The search database eligible for calibration was MEDLINE. There were no restrictions on publication period or language. We considered studies from inception until 20^th^ December 2018, the date when the search was performed. After the calibration, the most relevant search expression (**S3 File**) was used in four databases (MEDLINE, Web of Science, Scopus and CINAHL plus via EBSCOhost). The terms related to systematic reviews were chosen using information of balance between sensitivity and specificity terms, available in the literature [38–42].

### Study selection

#### Eligibility criteria

Included studies 1) are systematic reviews regardless of their objective or nature (including studies that have used a formal systematic review as their starting point) 2) have a primary health care scope and 3) aim at quality indicators assessment or development. We excluded studies that 1) did not have an abstract in the screening phase or 2) which, in the eligibility phase, did not have the full-text version available, even after direct contact with the author.

#### Screening phase

Once we obtained all the articles, duplicate between databases were identified and excluded using Endnote. From 2817 articles, a total of 1480 remained after removing the duplicates and were evaluated in the screening phase (reading of title and abstracts) by two independent reviewers and a third as a tie-breaker.

#### Eligibility phase

Full-texts of all the included articles were extracted (n = 33). As it was planned to contact the corresponding author if the full text of the article was not available, we used the ResearchGate website to extract full text articles, or to contact the authors for the articles that were not available. All eligible articles were assessed in full text format. The eligibility criteria were reapplied by two independent reviewers and a third as a tie-breaker, and the reference lists of each eligible article were scrutinized for any omitted studies.

#### Quality assessment and risk of bias

The evaluation of the quality and risk of bias of the eligible systematic reviews was carried out by evaluation through AMSTAR-2 tool [43]. The disagreement between the reviewers was solved by consensus in an agreement meeting by three reviewers. The AMSTAR-2 tool was considered for the definition of quality classification, fulfilling the systematic review research model. Articles that meet AMSTAR-2 requirements have been classified as "HIGH"; those that did not meet up to 2 relevant requirements were classified as "MODERATE", and those with more than 2 requirements not appraised were classified as "LOW". This quality assessment was carried out in order to understand how the studies were conducted and how the indicators were selected. However, none of the selected articles were excluded based on this assessment because the objective of this umbrella review does not include results from implementation of indicators, only a list of indicators implemented. The AMSTAR-2 items #11 and #12 were not applicable to the studies.

#### Data collection process

In first stage, a standard data extraction form was created, and general data extracted from each study included the following characteristics: article title, name of first author, publication type, country of origin, year of publication and indicators identified in the studies. Three reviewers independently extracted the data. Differences in data extracted was resolved by consensus method.

A second stage consisted in abstracting information regarding quality indicators using the primary studies in the systematic reviews included. This was necessary since some indicators identified in the systematic reviews lacked a proper description. Finally, indicators duplicated were identified by the reviewers involved in the first and second stage of data extraction and excluded through consensus.

#### Synthesis analysis

Analysis of the indicators were carried independently by two reviewers and third as a tie-breaker, who categorized the indicators presented in the systematic reviews included, according to five classifications frameworks: Context reflects the WHO ICPC-2 chapters categorization (General and Unspecified; Blood, Blood Forming Organs and Immune Mechanism; Digestive; Eye; Ear; Cardiovascular; Musculoskeletal; Neurological; Psychological; Respiratory; Skin; Endocrine/Metabolic and Nutritional; Urological; Pregnancy, Childbearing, Family Planning; Female Genital; Male Genital; Social Problems) [44]; the dimensions of care was defined based on the framework proposed by Donabedian to assess quality of healthcare (structure, process and outcome)[10,14], type of care reflects whether an indicator is associated with acute, chronic, or preventive care [10,45]; function of care conveys information about the purpose of health care (screening and prevention, diagnosis, treatment, follow up and continuity) [10,45] and domains and domain of health care quality was defined based on the framework proposed by National Academy of Medicine (NAM)(former Institute of Medicine) in 2001 (safe, effective, efficient, timely, patient-centred, equitable)[9].

Frequencies were computed based on these frameworks to analyse and summarize the information extracted, in two perspectives: Indicators by Context and Dimensions of care; and Type, Function and Domain by Dimensions of care.

## Results

### Search and study selection

The identification phase results returned 2817 articles (being 419 MEDLINE, 1452 Scopus, 567 ISI-WOS, 379 CINAHL via EBSCOhost). After removal of duplicate articles our research started with 1480 articles. Title and abstract were scrutinized for relevance based on inclusion and exclusion criteria. From a total of 1401 excluded articles, 1332 did not meet the eligibility criteria and 69 had no abstract available. The eligibility phase started with 79 articles that were read in their full-text versions, checking for the eligibility criteria. The studies identified by that involved RAND methodology, their inclusion in the umbrella review was justified since the methods included an initial systematic review prior the implementation of a panel discussion for validating appropriateness of indicators. Since the goal was to be as inclusive/comprehensive as possible, these studies were also included. In the perspective of the authors, the exclusion of these studies could compromise comprehensiveness of the umbrella review. The excluded studies (n = 46) did not have a full text version available or did not meet the eligibility criteria. Thirty-three articles were selected [29,30,46–77], for qualitative analysis and for the quality and risk of bias assessment. (Fig 1)

The Quality and Risk of Bias Assessment was carried out using the AMSTAR-2 assessment tool [45]. This assessment performed by the reviewers classified the confidence rate of each review as "low" (n = 14), “moderate” (n = 17) or “high” (n = 3) (**S4 File**).

Among the studies with low overall confidence rate, the main points of non-compliance with the requirements were, the non-performance of adequate studies selection with no extraction in duplicate (at least two independent reviewers); studies presented the quantity of excluded articles but without proper justification; not considering risk of bias (RoB) in individual studies when interpreting / discussing the results of the review; not using a satisfactory technique to assess RoB in individual studies that were included in the review and did not provide a satisfactory explanation for, or discussion of any heterogeneity observed in the results.

#### Study characteristics

**Fig 2:**
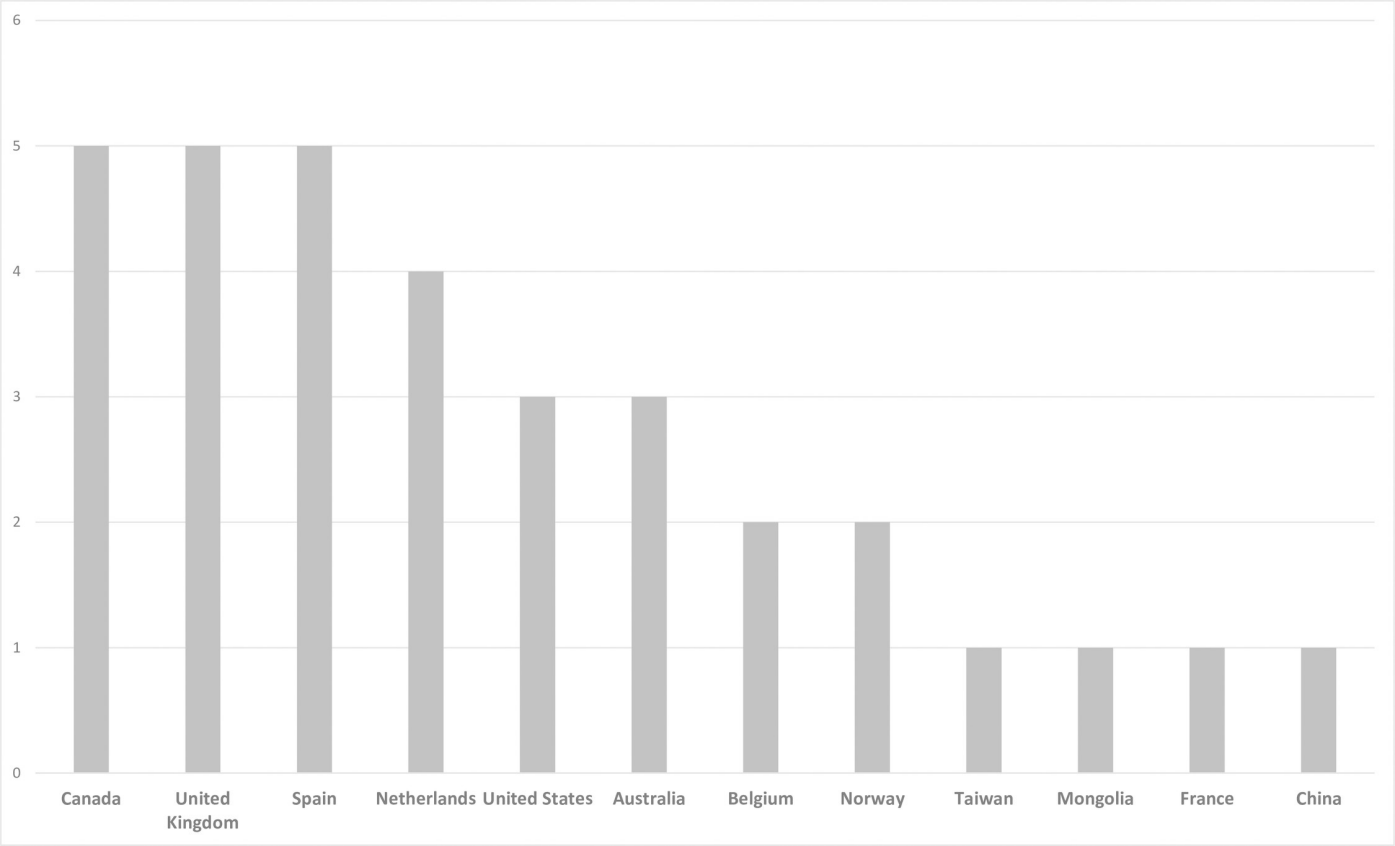
Included systematic reviews by country.

The 33 articles in this umbrella review included articles from Canada (n = 5), Spain (n = 5) and the United Kingdom (n = 5), among other countries (Fig 2).

Although the diversity of countries where the systematic reviews were performed, all articles were evaluated in the English version, except article #16 (Spanish).

The reviews comprised a total of 1406 included primary studies and 21 national guidelines, The databases used to search for these articles were the most varied, with the most used databases: MEDLINE (100%) and EMBASE (70%) (**Table 1**). Seven hundred and twenty seven (n = 727) indicators were extracted from the systematic reviews and primary studies in the reviews (Supplementary Material **S1 Appendix)**.

**Table 1:** Studies characteristics.

ID	Authors	Year	Country	Studies included (n)	Databases Searched	Main outcome
1	Jan C-F et al	2018	Taiwan	Missing	MEDLINE, National Digital Library of Theses and Dissertations in Taiwan, Airiti Library	Presents a narrative synthesis of the first 10 years since the launching of the Family Practice Integrated Care Project in Taiwan
2	Mazur A et al	2018	USA	20	MEDLINE, Web of Science, POPLINE	Presents indicators used for measuring youth-friendly sexual and reproductive health services
3	Kringos DS et al	2010	Netherlands	85	MEDLINE, Embase, Cochrane Library, CINAHL, King’s Fund Database, IDEAS Database, and EconLit	Identifies core dimensions that constitute a primary care system
4	Menear M, et al	2015	Canada	46	MEDLINE, Embase, PsycINFO, CINAHL, Cochrane Controlled Trials Register	Identifies data on quality of pharmacotherapy, psychotherapy, combined measures of treatment quality, and follow-up care. Conclusions state that chronic physical comorbidity does not consistently lead to lower quality of depression treatment or follow-up care in primary care
5	Batbaatar E, et al	2017	Mongolia, Italy	109	MEDLINE, CINAHL, Scopus	Identifies several determinants of patient satisfaction; Health care service quality indicators were the most influential determinants of patient satisfaction across the studies.
6	Bekkering GE, et al	2016	Belgium	6	MEDLINE, Embase, Cinahl, PsychInfo; Guidelines International Network; The National Guideline Clearinghouse; The New Zealand Guidelines Group; The Scottish Intercollegiate Guidelines Network (SIGN); Domus Medica (Belgian Association for Flemish General Practitioners); Nederlands Huisartsen Genootschap (Dutch Association for General Practitioners); Dutch Institute of Healthcare Improvement CBO; Société Scientifique de Médecine Générale (SSMG); National Institute of Clinical Excellence (NICE); Ebmpracticenet; World Health Organization (WHO guidelines on mental health and substance abuse); Resultaten Scoren Kenniscentrum Verslaving; National Quality Measures Clearinghouse; Trimbos Instituut; and CQAIMH databank.	Identifies a set of indicators of care for alcohol use disorder, a total of 10 process and outcome indicators.
7	Lopez-Vazquez P, et al.	2016	Spain	46	MEDLINE, Embase	Describes how to o use quality and/or quantity indicators to define ‘misprescription’
8	Barber CE, et al.	2015	Canada, USA	20	MEDLINE, Embase, CINAHL, Web of Science	Describes eleven for care in patients with RA have been developed and are rated as highly relevant, valid, and feasible by an international multidisciplinary panel.
9	Caughey GE, et al	2014	Australia	Missing	MEDLINE, Embase	The study provides a set of face and content validated indicators of medication-related potentially preventable hospitalisations
10	Boeckxstaens P, et al	2011	Belgium	27	MEDLINE, Econlit	Concerning equity in treatment and (intermediate) treatment outcomes, the study shows that overall quality scores generally improved. For almost of the observed indicators, all citizens benefit from this improvement
11	To, et al.	2010	Canada	135	Cochrane Database of Systematic Reviews, MEDLINE, EMBASE, CINAHL	Defines a setlist of performance indicators of asthma care, organized in five domains: access to care, clinical effectiveness, patient centeredness, system integration and coordination and patient safety.
12	Flodgren G, et al	2016	Norway, UK, Canada	2	Cochrane Central Register of Controlled Trials (CENTRAL), MEDLINE, Embase, Database of Abstracts of Reviews of Effectiveness, HMIC, ClinicalTrials.gov and the World Health Organization International Clinical Trials Registry Platform.	Shows that there are few high-quality controlled evaluations of the effectiveness and the cost-effectiveness of external inspection systems. One study reported improved compliance scores with hospital accreditation standards.
13	Addington D et al	2010	Canada	57	CINAHL, EMBASE, MEDLINE, and PsycINFO	Shows that successful implementation of quality measures can occur, but that success depends on the interaction of multiple factors, including measure characteristics (key attributes), promotional messages, implementation strategies, resources, the intended adopters, and the intraorganizational and interorganizational contexts.
14	Sans-Corrales M, et al	2006	Spain	20	MEDLINE and Cochrane Library (The Cochrane Controlled Trial Register)	Identifies attributes of Family Medicine that are related to the outcomes with respect to dimensions of satisfaction, health and costs.
15	Spencer R, et al	2014	United Kingdom	85	Embase, CINAHL, MEDLINE, MEDLINE (Ovid 1996 onward), Health Management Information Consortium, and Web of Science.	Identifies and updates a set of prescribing safety indicators for assessing the safety of prescribing in general practice, and to estimate the risk of harm to patients associated with each indicator.
16	Lima AOD, et al	2017	Spain	5	MEDLINE, EMBASE and CINAHL	Review focused on indicators of care in osteoarthrosis, chronicity, childhood asthma, clinical effectiveness and indicators on prescription safety.
17	James DH, et al	2008	United Kingdom	14	Metalib on the MEDLINE, Embase, MEDLINE and PsycINFO databases and individual journal searches for the Pharmaceutical Journal and International Journal of Pharmacy Practice. The PSNC, National Pharmacy Association (NPA) and RPSGB websites were also accessed.	Developsexplicit criteria against which the quality of medicines use review referral documentation can be assessed.
18	Kronenberg C, et al	2017	United Kingdom	27	Applied Social Sciences Index and Abstracts (ASSIA); CENTRAL; Cochrane Database of Systematic Reviews; Conference Proceedings Citation Index-Science (CPCI-S); Database of Abstracts of Reviews of Effects (DARE); EMBASE; Ovid MEDLINE In Process & Other Non-Indexed Citations and Ovid MEDLINE; PsycINFO; and MEDLINE.	Creates a list of quality indicators relevant to patients with serious mental illnesses that could be captured using routine data, and which could be used to monitor or incentivise better-quality primary care.
19	Ruiz-Canela-Cáceres J, et al	2015	Spain	167	MEDLINE and Embase.	Identifies indicators regarding Asthma Care
20	Smits KPJ, et al	2016	Netherlands	31	MEDLINE and Embase.	Identifies quality indicators (QI) measuring processes of care for chronic kidney disease, and identifies the QIs that have content, face, operational and/or predictive validity
21	Yazdany J, et al.	2009	USA	Missing	MEDLINE and Embase.	Identifies set of QI for systemic lupus erythematosus (SLE).
22	Le Maréchal M, et al.	2008	France	54	MEDLINE.	Identifies a set of outpatient QIs to measure the appropriateness of antibiotic use
23	Duhoux A, et al	2011	Canada	65	MEDLINE, Embase and PsycINFO	Identifies indicators used to measure the quality of depression treatment in primary care and explore factors leading to divergent results
24	Fujita K, et al	2018	Australia	131	CINAHL, Embase, Global Health, International Pharmaceutical Abstract, MEDLINE, and Web of Science	This study was the first systematic review classifying QIs using multiple frameworks
25	Chin WY, et al	2011	China	21 national guidelines + 33 studies	Ovid MEDLINE,Cochrane Database, RAND (Research and Development) Corporation Health Database, the ACOVE (Assessing the Care of Vulnerable Elders) project and clinical guidelines	Identifies the factors determining quality of care for nurse-led and allied health personnel–led clinics on six programmes (fall prevention, continence care, pulmonary rehabilitation, mental wellness, medication compliance, and wound care).
26	Hagen KB, et al	2016	Norway	15	MEDLINE, Embase, PsychInfo and Cinahl	Evaluates the state of quality of care for Osteoarthritis, specially the care provided to patients.
27	Lake R, et al.	2017	Australia	10	MEDLINE, EMBASE, CINAHL, Web of Science and the Cochrane Library	Determines the scope, consistency and generalisability of findings in relation to the governance, safety and quality of telephone triage and advice services.
28	Sidorenkov G, et al	2011	Netherlands	24	MEDLINE and Embase	Assesses whether quality indicators for diabetes care are related to patient outcome.
29	Forbes LJ, et al	2017	United Kingdom	8	COCHRANE, MEDLINE, EMBASE and Health Management Information Consortium	Assesses evidences supporting that the Quality and Outcomes Framework (QOF) has improved quality of care for patients with long term conditions.
30	Fernández-Urrusuno R, et al	2015	Spain	3	COCHRANE, DOCUMED, EMBASE, ERIC, IBECS, IME-Biomedicina, LILACS, MEDLINE, SciELO.	Develop basic indicators for monitoring the prescription and proper use of antimicrobials in primary care.
31	Martirosya L, et al	2010	Netherlands	59	MEDLINE and EMBASE	Describes the validity of existing QI for type 2 diabetes mellitus and cardiovascular risk management.
32	Byrne MJ, et al	2018	United Kingdom	22	MEDLINE, Psychinfo, EMBASE, Health and Psychosocial Instruments and Social Policy and Practice via OVID.	Identifies measures used to assess quality in primary care dentistry.
33	Pugh MJ, Bet al.	2007	USA	Missing	MEDLINE and CINAHL	Presents quality indicators for evaluating care of adults with epilepsy, in primary care and general neurology clinics.

The dimension of care with the highest number of indicators by context was process (n = 548, 74.5%), followed by outcome (n = 146, 20.0%) and structure (n = 46, 6.0%). The frequency of indicators among the classification by dimension of care and condition contexts is shown in **Table 2**. When analysed by dimension of care and condition context, the indicator totals within each dimension (columns) could not be added up because there were indicators (n = 13) that participate in more than one context category within each dimension of care. The total number of indicators analysed was the denominator of the percentage in parentheses and refers to the total number of indicators in the extraction list (n = 727) indicated in the heading. The same is observed in context totals (lines). The ranking of the highest number of indicators found were classified in the categories A—general and non-specific followed by the K—Circulatory System categories specific, P–Psychological and R—Respiratory System. The categories B—Blood, hematopoietic and lymphatic organs, H—Ears and Z—Social Issues, had no indicators presented in the included studies.

**Table 2:** Dimensions of care by context.

Contexts1	Total Indicators (n = 727, 100%)				
	Structure (n, %)	Process (n, %)	Outcome (n, %)	Total (n, %)	Rank
**A General and non-specific**	1 (0.1)	88 (12.4)	24 (8.3)	**112 (15.4)**	**1**
**B Blood, hematopoietic and lymphatic organs**	0	0	0	**0**	**NA**
**D Digestive tract**	0	12 (1.7)	13 (1.8)	**25 (3.4)**	**9**
**F Eyes**	0	1 (0.1)	0	**1 (0.1)**	**13**
**H Ears**	0	0	0	**0**	**NA**
**K Circulatory system**	0	90 (12.4)	21 (2.9)	**111 (15.3)**	**2**
**L Musculoskeletal System**	0	60 (8.3)	5 (0.7)	**65 (8.9)**	**6**
**N Nervous system**	0	2 (0.3)	1 (0.1)	**3 (0.4)**	**12**
**P Psychological**	9 (1.2)	78 (10.7)	16 (2.2)	**103 (14.2)**	**3**
**R Respiratory system**	0	63 (8.7)	29 (4.0)	**92 (12.7)**	**5**
**S Skin**	1 (0.1)	0	0	**1 (0.1)**	**13**
**T Endocrine, metabolic and nutritional**	0	36 (5.0)	20 (2.8)	**56 (7.7)**	**7**
**U Urinary System**	0	30 (4.1)	5 (0.7)	**35 (4.8)**	**8**
**W Pregnancy and family planning**	1 (0.1)	16 (2.2)	2 (0.3)	**19 (2.6)**	**10**
**X Female genital tract (including breast)**	2 (0.3)	10 (1.4)	1 (0.1)	**13 (1.8)**	**11**
**Y Male genital tract**	1 (0.1)	1 (0.1)	1 (0.1)	**3 (0.4)**	**12**
**Z Social issues**	0	0	0	**0**	**NA**
**Not defined**	31 (4.3)	61 (8.4)	8 (1.1)	**100 (13.8)**	**4**
**TOTAL by Dimensions of Care**	**46 (6.0)**	**548 (75.0)**	**146 (20.0)**	** **	

Among the indicators of structure (n = 45), the indicators with the most frequent type of care were those classified in all three categories—Acute, Chronic and Preventive (n = 34, 45.3%), e.g. Professional profiles; Primary care expenditures; Availability of primary care services. Those of specific category of type of care were less frequent (**Table 3**).

**Table 3:** Indicators by type of care, function and domain in structure dimension.

Structure Indicators (n = 45)		
**Type of care**	**n**	**Examples**
**Acute**	3	Crisis management and out-of-hours services; Abortion services; Accommodation "patient-focused on": Out-of-hours service
**Chronic**	6	Wound care clinics; Informal carer; Register of patients with serious mental health problems
**Preventive**	2	Pregnant and parenting teen services; Sexually transmitted infections services
**>3 categories**	**34**	Professional profiles; Primary care expenditures; Availability of primary care services
**Function**	**n**	**Examples**
**Diagnosis**	4	Sexually transmitted infections services; Register of patients with dementia; Register of patients with learning disability
**Screening and Prevention**	0	Not addressed
**Follow up and continuity**	3	Integration of primary care in the health care system; Wound care clinics; Pregnant and parenting teen services
**Treatment**	3	Abortion services; Need for accessibility; Cost of treatment per unit
**>3 categories**	**35**	Availability: Number of physicians per unit of population; Availability: Number of hospital beds per unit of population; Technical efficiency
**Domain**	**n**	**Examples**
**Effective**	**22**	Governance: (De)centralization of primary care management and service development; Integration of primary care in the health care system; Appropriate technology in primary care
**Efficiency**	8	Efficiency in performance of primary care workforce; Technical efficiency; Allocative and productive efficiency
**Timely**	1	Need for accessibility
**Patient-centered**	2	Employment status; Accommodation "patient-focused on": Out-of-hours service
**Safe**	0	Not addressed
**>3 categories**	12	Future development of the primary care workforce; Education and retention; Income of primary care workforce

Structure indicators were more commonly assigned to more than three functions of care (n = 35, 77.7%) (Diagnosis, Screening and Prevention, Follow-up and continuity, Treatment), eg Availability: Number of physicians per unit of population; Availability: Number of hospital beds per unit of population; Technical efficiency.

Most structure indicators were associated with the effective domain of health care quality (n = 22, 48.8%) e.g. Governance: (From) centralization of primary care management and service development; Integration of primary care in the health care system; Appropriate technology in primary care. No structure indicators was associated with the safe domain of health care quality.

Among the indicators of process (n = 542), Chronic care was the most frequent type of care observed (n = 355, 65.5%), e.g. Comorbid psychiatric conditions and response to treatment; Follow-up contacts during treatment episode after initial evaluation; Comprehensive diabetes care: HbA1c testing. Preventive care (n = 88, 16.2%) and all types of care (n = 80, 14.7%) shared similar frequencies (**Table 4**).

**Table 4:** Indicators by type of care, function and domain in process dimension.

Process Indicators (n = 542)		
**Type of care**	**n**	**Examples**
**Acute**	38	Patients initiating depression treatment; Emergency contraception; Patient compliance to advice given to seek emergency care
**Chronic**	**355**	Comorbid psychiatric conditions and response to treatment; Follow-up contacts during treatment episode after initial evaluation; Comprehensive diabetes care: HbA1c testing
**Preventive**	88	Quality of maternal and child health care: occurrence of preventive screening for pregnant women; Pap smears and pregnancy tests; Elderly Influenza Vaccination
**> 2 categories**	80	Waiting time to treatment; Up-to-date and confidential medical record keeping; Patient compliance to advice given to seek GP
**Function**	**n**	**Examples**
**Diagnosis**	24	Diagnosis and treatment—primary care: Re-measurement of blood pressure for those with high blood pressure; Cardiovascular disease risk assessment; Percentage of patients with a new diagnosis of dementia with record of tests to exclude reversible cause; Quality of diagnosis and treatment in primary care
**Screening and Prevention**	111	Pap smear rate; Urinary incontinence during initial dementia evaluation; Preventive care Immunizable conditions; Medical attention for nephropathy
**Follow up and continuity**	111	Follow up by the same clinician; Plan for follow up care explained and scheduled; Extra pyramidal effects monitoring; Percentage of patients with asthma and measures of variability or reversibility recorded
**Treatment**	254	Tranquilisers prescribed: % of the recommended; Possible contraindications should be taken into account when antibiotics are prescribed; Co-prescription of itraconazole with simvastatin, or with atorvastatin at a dose ≥80mg
**>3 categories**	42	Sufficient time for consultation; Comfort in communicating; Child healthcare in general practice; Privacy and Confidentiality
**Domain**	**n**	**Examples**
**Effective**	**310**	Follow-up contacts during treatment episode after initial evaluation; Coordinated care; Asthma: Percentage of children with follow-up from the same doctor for at least 80% of their visits
**Efficiency**	6	Utilisation of primary care services; Resolution capacity; Gatekeeping system
**Equitable**	5	Equality in access; Access to services; Governance: Policy on equity in access to primary care services;
**Timely**	7	Waiting time to treatment; Availability of telephone triage and advice services; Promptness of antidepressant treatment follow-up
**Patient-centered**	57	Communication centred on the patient (recorded interview and perception of the patient); Patient education; Patient advocacy
**Safe**	152	Detection of Falls; Polyfarmacy; Systemic Lupus Erythematosus: Discussion about teratogenic risks of medication
**>3 categories**	6	First contact for common health problems; Informational continuity of care; Primary care-supportive governmental policies for delivery of preventive care

Treatment was the most frequent function of care of the process indicators (n = 254, 46.8%), e.g Tranquilisers prescribed: % of the recommended; Possible contraindications should be taken into account when antibiotics are prescribed; Co-prescription of itraconazole with simvastatin, or with atorvastatin at a dose ≥80mg. Screening and prevention and Follow up and continuity were also common, associated with 111 indicators each. Examples of Screening and Prevention indicators are: Pap smear rate; Urinary incontinence during initial dementia evaluation; Preventive care Immunizable conditions; Medical attention for nephropathy; and of Follow up and Continuity: Follow up by the same clinician; Plan for follow up care explained and scheduled; Extra pyramidal effects monitoring; Percentage of patients with asthma and measures of variability or reversibility recorded. Most process indicators were also associated with the effective domain of health care quality (n = 310, 57.2%) e.g. Follow-up contacts during treatment episode after initial evaluation; Coordinated care; Asthma: Percentage of children with follow-up from the same doctor for at least 80% of their visits. Also a common domain of health care quality in the listing was Safe (n = 152, 28%), e.g. Detection of Falls; Polyfarmacy; Systemic Lupus Erythematosus: Discussion about teratogenic risks of medication.

Among the indicators of Outcome (n = 140), Chronic care was the most frequent type of care observed (n = 67, 47.8%), e.g. Absenteeism from Work/School for Asthma; Proportion with increased BMI / abdominal waist line; Prevention of pressure ulcers in patients included in the chronic dependent patients care program; Duration of untreated psychosis. The frequency of indicators regarding acute care only (n = 51, 36.4%) and preventive care (n = 44, 31.4%) were similar (**Table 5**).

**Table 5:** Indicators by type of care, function and domain in outcome dimension.

Outcome Indicators (n = 140)		
**Type of care**	**n**	**Examples**
**Acute**	51	Quality of health promotion: Gonorrhoea/chlamydia rates; Duration of untreated psychosis; Potentially preventable hospitalisation clinical indicator of Serotonin toxicity
**Chronic**	**67**	Absenteeism from Work/School for Asthma; Proportion with increased BMI / abdominal waist line; Prevention of pressure ulcers in patients included in the chronic dependent patients care program; Duration of untreated psychosis
**Preventive**	44	Potentially preventable hospitalisation clinical indicator of Arrhythmia; Percentage of patients with diabetes who have had influenza immunisation; Frequency of adverse events, errors and hospitalisation rates
**>3 categories**	15	Reduction in absolute risk; Quality from the Patient’s Perspective Questionnaire; Questions on satisfaction, communication, personal relationship, awareness of problems and interest in the effects of the problem on personal and family quality of life
**Function**	**n**	**Examples**
**Diagnosis**	22	Severity of symptoms; Preventable adverse events in primary care related to diagnosis; Proportion of patients who have an increased blood glucose level
**Screening and Prevention**	25	Quality of maternal and child health care: maternal mortality rates; Quality of health promotion: Smoking rate; Preventive care: Low birth weight rate
**Follow up and continuity**	17	Asthma: Days free of symptoms in the two previous weeks; Patient satisfaction with the family physician/specialist coordination of care; Quality of Life in patients with urinary Incontinence
**Treatment**	57	Sedation side effects; Number of deaths in seven days between those whose calls were handled by doctors or nurses
**>3 categories**	19	Dental patient feedback on consultation skills (DPFCS); Proportion of patients that is satisfied with the quality of contact with his care giver(s)
**Domain**	**n**	**Examples**
**Effective**	91	Potentially preventable hospitalisation clinical indicator of Chronic Obstructive Pulmonary Disease; Comorbid psychiatric conditions and response to treatment
**Efficiency**	7	Asthma: Percentage of children with one or more visits to ER in a year; Cumulative hospitalization days in patients with chronic conditions
**Timely**	1	Delayed diagnosis
**Patient-centered**	26	Patients with multiple chronic conditions and medications attended in primary care; Patient Quality of Life; Patient satisfaction with the family physician/specialist coordination of care
**Safe**	13	Asthma: Patient with two or more rounds of corticoids due to an attack in three months and with no prescribed basic treatment; Preventable adverse events in primary care related to drugs; MRSA (methicillin-resistant Staphylococcus aureus) infection rates

Treatment was the most frequent function of care within outcome indicators (n = 57, 40.7%), e.g Sedation side effects; Number of deaths in seven days between those whose calls were handled by doctors or nurses. Screening and Prevention (n = 25, 17.8%) and Diagnosis (n = 22, 15.7%) followed with similar number of outcome indicators. Examples of Screening and Prevention indicators are: Quality of maternal and child health care: maternal mortality rates; Quality of health promotion: Smoking rate; Preventive care: Low birth weight rate.

Finally, most outcome indicators were associated with the effective domain of health care quality (n = 91, 65%) e.g. Potentially preventable hospitalisation clinical indicator of Chronic Obstructive Pulmonary Disease; Comorbid psychiatric conditions and response to treatment. Also, a common health care quality domain in the listing was Patient-centered (n = 26, 18.5%), e.g. Patients with multiple chronic conditions and medications attended in primary care; Patient Quality of Life; Patient satisfaction with the family physician/specialist coordination of care.

## Discussion

Primary health care (PHC) is where the patient's first contact with the health system occurs and comprises a range of actions which includes many dimensions, domains, and contexts [14]. Due to these characteristics, it becomes important to evaluate and monitor the quality of primary care [78–80]. It is established that primary care can lead to better health outcomes, lower costs, and greater equity in health [81] and this can be achieved by using QIs, a set of objective measures with clinical evidence [6,82–83] that can represent an acceptable standard of care across a specific patient population [84].

As the aim of this umbrella review of systematic reviews was to search indexed literature, in order to find a setlist of QI useful for monitoring quality in PHC, our study shows interesting answers to what was proposed, identifying 33 systematic reviews of studies on quality indicators in primary health care and providing a list of selected indicators considered in the included reviews. The study resulted in 727 quality indicators, which were later categorized by context, dimension, type of care, function and domain.

### Context

Context of care was classified according to the International Classification of Primary Care (ICPC-2), which is recommended by the World Organization of Family Doctors (WONCA) for codification in this level of care.[44]. Although practical and useful for primary care, this classification represents a simplification and attempt at uniformization with other classification systems such as the International Classification of Diseases (ICD-11), which is not achieved completely [85]. Furthermore, since ICPC-2 is a classification based primarily in the location of the symptoms or disease, the authors could not define the context for 100 indicators, since they relate mostly to organizational measures not contemplated in adopted system. “Not defined” was the fourth most common context, representing 13.8% of the total of indicators found.

The majority of the indicators belong to the context category “A–General and Unspecified” (n = 112, 15.4%), which may reflect an attempt at creating indicators applicable to a wide range of procedures and contexts. Circulatory, psychological, respiratory, musculoskeletal and endocrine/metabolic diseases are the next most frequent contexts, indicating also a bigger concern for areas which are more prevalent in primary care **(see Table 2)**.

### Dimension

Most of the indicators found by the authors were related to the dimension of Process (n = 542, 74.5% of total). As defined by Donabedian, this dimension focuses in what is actually done, such as patient’s procedures in seeking care and practitioner’s activities while providing it [14]. Since QIs represent an opportunity for improvement in areas where quality standards are not met, process indicators may help implementing better procedures and guidelines, resulting in better health care. Outcome dimension was the second most frequent dimension (n = 140, 19.2%); since healthcare outcomes depend on the care provided, these indicators evaluate the result of the course of action of PHC professionals, unlike process indicators which evaluate a single aspect of care.

### Type of care

Type of care was classified as acute, chronic or preventive, with “Chronic” being the most frequent. Indicators focused on chronic care are very helpful, since family doctors follow their patients longitudinally for many years, monitoring and managing the chronic diseases they develop throughout their lives [79]. The management and control of chronic conditions/diseases in the population is one of the main focuses of the activities of primary health care, being also the most studied and evaluated by the QIs, as our study demonstrates. Indicators such as control of prescriptions and monitoring of diseases such as asthma, COPD, hypertension and diabetes, as well as indicators of ambulatory care sensitive conditions that can generate avoidable hospitalizations are part of the list of indicators presented [30].

### Function

Indicators relating to “Treatment” were the most frequent, followed by “Screening and Prevention” and “Follow-up and Continuity”. Once again, the results mirror important aspects of PHC. The consideration of the patient as a whole and the approach of disease in a holistic perspective imply that the healthcare provider must consider indications, potential adverse effects and comorbidities of each patient before elaborating a treatment plan [79]. Within outcome indicators, most these were focused on treatment, contributing to the evaluation of its complications and preventable hospitalizations, once again alerting providers to re-evaluate their patients and review therapeutic options.

Regarding “Screening and Prevention”, the prevention of disease as well as early diagnosis are the main focus of this level of care [1]; the development of screening programs for oncological conditions and adequate follow-up for prevention of complications contribute to better health care in this aspect.

### Domain

“Effective” was the most common domain among the three dimensions of care. Indicators under this domain focus on the capacitation of PHC providers and their articulation with secondary care. Since the effectiveness of a health system depends on the quality of its primary care [29,86], it would be expected that this would be an area of interest.

Other domains such as “Patient-centered” or “Safety” were also commonly evaluated through QIs, demonstrating once again the concern for a holistic approach of PHC.

### Limitations

Although there is a significant amount of literature on health quality indicators, some of them are not directly linked to PHC, making it difficult to extrapolate the conclusions of the QI that are applied mainly to the secondary and tertiary levels of attention. Most articles published on QIs in PHC tend to choose very limited and specific areas of health care, without a generic approach to PHC as a whole. The uniqueness and heterogeneity found in these studies show the importance of comprehensive systematic reviews on PHC.

Systematic reviews included in this paper selected primary studies using slightly different methodological assessment and statistical pooling; some of these articles did not discriminate how many primary studies were included in the analysis. The use of different databases in each systematic review and different methods for choosing search terms, calibration and specificity of the search expressions must be considered when interpreting the results.

The authors of this article have searched the primary studies included in each systematic review in order to obtain a list of PHC quality indicators. The lack of a uniform method to collect and present the QIs among the included reviews limited the ability to withdraw complete information from every paper. As an example, most studies were missing information regarding the numerator, denominator and calculation method for each QI.

## Conclusions

This is, to the best of our knowledge, the first umbrella review focusing on QIs for primary healthcare in a border scope. We present a final list of indicators (S1 Appendix **supplementary material**) from eligible systematic reviews summarizing the indicators available in the literature, allowing us to understand which areas of primary care are better covered by these measures. The results of our umbrella review are valuable and imply the need for future research and practice regarding quality indicators, as a great opportunity for further studies to test the acceptability, feasibility, reliability, comparison tools and validity of those indicators, while also checking for problems with their implementation to PHC, with adequate information and registration systems. It also provides a ready way for clinicians, managers and health decision makers to gain a clear understanding of the most evidence-based publications related to PHC quality indicators.

## Supporting information

S1 File Prisma statement checklist. (PDF)

S2 File PROSPERO protocol register. (PDF)

S3 File Search expression query. (PDF)

S4 File Quality and risk of bias assessment. (PDF)

S1 Appendix Supplementary material–indicators list. (PDF)
