# Supplementary material for: Primary health care quality indicators: An umbrella review
Source: PLoS One. 2019 Aug 16;14(8):e0220888. doi: 10.1371/journal.pone.0220888 (PMC6697344; doi:10.1371/journal.pone.0220888)
Supplement: S2 File — (PDF) [file pone.0220888.s002.pdf]

Systematic review

1. * Review title.

Give the working title of the review, for example the one used for obtaining funding. Ideally the title should state succinctly the interventions or exposures being reviewed and the associated health or social problems. Where appropriate, the title should use the PI(E)COS structure to contain information on the Participants, Intervention (or Exposure) and Comparison groups, the Outcomes to be measured and Study designs to be included.

Primary health care quality indicators: an umbrella review

2. Original language title.

For reviews in languages other than English, this field should be used to enter the title in the language of the review. This will be displayed together with the English language title.

3. * Anticipated or actual start date.

Give the date when the systematic review commenced, or is expected to commence.

01/10/2018

4. * Anticipated completion date.

Give the date by which the review is expected to be completed.

09/03/2019

5. * Stage of review at time of this submission.

Indicate the stage of progress of the review by ticking the relevant Started and Completed boxes. Additional information may be added in the free text box provided.
Please note: Reviews that have progressed beyond the point of completing data extraction at the time of initial registration are not eligible for inclusion in PROSPERO. Should evidence of incorrect status and/or completion date being supplied at the time of submission come to light, the content of the PROSPERO record will be removed leaving only the title and named contact details and a statement that inaccuracies in the stage of the review date had been identified.
This field should be updated when any amendments are made to a published record and on completion and publication of the review. If this field was pre-populated from the initial screening questions then you are not able to edit it until the record is published.

The review has not yet started: No

Review stage	Started	Completed
Preliminary searches	Yes	Yes
Piloting of the study selection process	Yes	Yes
Formal screening of search results against eligibility criteria	Yes	Yes
Data extraction	Yes	Yes
Risk of bias (quality) assessment	Yes	Yes
Data analysis	Yes	No

Provide any other relevant information about the stage of the review here (e.g. Funded proposal, protocol not yet finalised).

6. * Named contact.

The named contact acts as the guarantor for the accuracy of the information presented in the register record.

Andre Luis Charro Ramalho

Email salutation (e.g. "Dr Smith" or "Joanne") for correspondence:

Dr Ramalho

7. * Named contact email.

Give the electronic mail address of the named contact.

andrelcramalho@gmail.com

8. Named contact address

Give the full postal address for the named contact.

CINTESIS - Center for Research in Health Technologies and Services, R. Dr. Placido da Costa, 4200-450

Porto, Portugal

9. Named contact phone number.

Give the telephone number for the named contact, including international dialling code.

+351927635899

10. * Organisational affiliation of the review.

Full title of the organisational affiliations for this review and website address if available. This field may be completed as 'None' if the review is not affiliated to any organisation.

Faculty of Medicine - University of Porto

Organisation web address:

www.cintesis.eu

11. * Review team members and their organisational affiliations.

Give the title, first name, last name and the organisational affiliations of each member of the review team. Affiliation refers to groups or organisations to which review team members belong.

Dr Andre Ramalho. Faculty of Medicine - University of Porto
Dr Joao Vasco Santos. Faculty of Medicine - University of Porto
Dr Juliana Teixeira. Faculty of Medicine - University of Porto
Dr Manuel Gonçalves-Pinho. Faculty of Medicine - University of Porto
Dr Pedro Castro. Faculty of Medicine - University of Porto
Professor Alberto Freitas. Faculty of Medicine - University of Porto
Mr João Viana. Faculty of Medicine - University of Porto

12. * Funding sources/sponsors.

Give details of the individuals, organizations, groups or other legal entities who take responsibility for initiating, managing, sponsoring and/or financing the review. Include any unique identification numbers assigned to the review by the individuals or bodies listed.

Supported by National Funds through FCT - Fundação para a Ciência e a Tecnologia within CINTESIS, R&D Unit (reference UID/IC/4255/2019)

13. * Conflicts of interest.

List any conditions that could lead to actual or perceived undue influence on judgements concerning the main topic investigated in the review.

None

14. Collaborators.

Give the name and affiliation of any individuals or organisations who are working on the review but who are not listed as review team members.

15. * Review question.

State the question(s) to be addressed by the review, clearly and precisely. Review questions may be specific or broad. It may be appropriate to break very broad questions down into a series of related more specific questions. Questions may be framed or refined using PI(E)COS where relevant.

To provide a set list based on an umbrella review assessing the evidence about the use of quality indicators in primary health care.

16. * Searches.

Give details of the sources to be searched, search dates (from and to), and any restrictions (e.g. language or publication period). The full search strategy is not required, but may be supplied as a link or attachment.

MEDLINE, Web of Science, Scopus, and CINAHL plus via EBSCOhost were searched for relevant literature. The gray literature, or reports and articles that are not commercially published will be used for further research into the set of predicted indicators at the end of the study.

Additional search strategy information can be found in the attached PDF document (link provided below).

17. URL to search strategy.

Give a link to a published pdf/word document detailing either the search strategy or an example of a search strategy for a specific database if available (including the keywords that will be used in the search strategies), or upload your search strategy.Do NOT provide links to your search results.

https://www.crd.york.ac.uk/PROSPEROFILES/124170_STRATEGY_20190305.pdf

Alternatively, upload your search strategy to CRD in pdf format. Please note that by doing so you are consenting to the file being made publicly accessible.

Do not make this file publicly available until the review is complete

18. * Condition or domain being studied.

Give a short description of the disease, condition or healthcare domain being studied. This could include health and wellbeing outcomes.

Primary health care: we are investigating the quality indicators.

19. * Participants/population.

Give summary criteria for the participants or populations being studied by the review. The preferred format includes details of both inclusion and exclusion criteria.

Participants in the primary health care system.

20. * Intervention(s), exposure(s).

Give full and clear descriptions or definitions of the nature of the interventions or the exposures to be reviewed.

The use or implementation of quality indicators in the primary health care system.

21. * Comparator(s)/control.

Where relevant, give details of the alternatives against which the main subject/topic of the review will be compared (e.g. another intervention or a non-exposed control group). The preferred format includes details of both inclusion and exclusion criteria.

Indicators, domains and dimensions of care.

22. * Types of study to be included.

Give details of the types of study (study designs) eligible for inclusion in the review. If there are no restrictions on the types of study design eligible for inclusion, or certain study types are excluded, this should be stated. The preferred format includes details of both inclusion and exclusion criteria.

Systematic reviews and overviews of reviews.

23. Context.

Give summary details of the setting and other relevant characteristics which help define the inclusion or exclusion criteria.

Health quality indicators in the primary care setting.

24. * Main outcome(s).

Give the pre-specified main (most important) outcomes of the review, including details of how the outcome is defined and measured and when these measurement are made, if these are part of the review inclusion criteria.

Primary health care quality indicators: to present a summary of the indicators used in systematic reviews

regarded as having good quality and a low risk of bias.

Timing and effect measures

Not applicable.

25. * Additional outcome(s).

List the pre-specified additional outcomes of the review, with a similar level of detail to that required for main outcomes. Where there are no additional outcomes please state 'None' or 'Not applicable' as appropriate to the review

None.

Timing and effect measures

26. * Data extraction (selection and coding).

Give the procedure for selecting studies for the review and extracting data, including the number of researchers involved and how discrepancies will be resolved. List the data to be extracted.

Screening Phase:

Once we obtained all the articles from the databases, duplicate articles between databases were identified and excluded using EndNote®. A total of 1480 articles remained after removing the duplicates and were evaluated by reading of title and abstracts by three independent reviewers. The reviewers read the titles and abstracts of all articles resulting from the search.

Eligibility Phase:

Full-texts of all the included articles were extracted. As it was planned to contact the corresponding author if the full text of the article was not available, we reach ResearchGate website to extract full articles available or to ask for the articles. Also, we have sent an email for authors asking for articles that were not obtained. All those eligible articles were assessed in full text format. The eligibility criteria were reapplied by three independent reviewers. The reference lists of each eligible article scrutinized for any omitted studies.

Data Collection Process:

A standard data extraction form was created and general data extracted from each study included the following variables: article titles, first authors' names, publication type, country of origin, year of publication, objectives, areas by field of health, specific diagnostics, programs, indicators and its measures available. Every reviewer will independently extract the data. Differences in data extracted will also be resolved by consensus method. Missing data was requested from study authors.

27. * Risk of bias (quality) assessment.

State whether and how risk of bias will be assessed (including the number of researchers involved and how discrepancies will be resolved), how the quality of individual studies will be assessed, and whether and how this will influence the planned synthesis.

The evaluation of the quality and risk of biases of the eligible systematic reviews will be carried out by evaluation through AMSTAR-2 tool. The disagreement between the reviewers was solved by consensus in an agreement meeting. AMSTAR-2 assessment tool comprises 16 items, each of which provides a short single-sentence question with additional guidance on selecting response options (expressed as: 'yes', partial yes and 'no'). This tool was chosen because we believe it is the most adequate to present characteristics as a critical evaluation and because it is usable and self-contained.

28. * Strategy for data synthesis.

Give the planned general approach to synthesis, e.g. whether aggregate or individual participant data will be used and whether a quantitative or narrative (descriptive) synthesis is planned. It is acceptable to state that a quantitative synthesis will be used if the included studies are sufficiently homogenous.

Initially, a qualitative analysis of the data is planned, and the construction of a set list of quality indicators, with the characteristics of those indicators presented from the source studies.

According to the availability of the studies presenting quantitative measures, a meta-analysis of the results may also be performed.

29. * Analysis of subgroups or subsets.

Give details of any plans for the separate presentation, exploration or analysis of different types of participants (e.g. by age, disease status, ethnicity, socioeconomic status, presence or absence or co-morbidities); different types of intervention (e.g. drug dose, presence or absence of particular components of intervention); different settings (e.g. country, acute or primary care sector, professional or family care); or different types of study (e.g. randomised or non-randomised).

Subgroup analyses will be performed by publication year, country and domains.

30. * Type and method of review.

Select the type of review and the review method from the lists below. Select the health area(s) of interest for your review.

Type of review

Cost effectiveness
No

Diagnostic
No

Epidemiologic
No

Individual patient data (IPD) meta-analysis
No

Intervention
No

Meta-analysis
No

Methodology
No

Narrative synthesis
No

Network meta-analysis

No

Pre-clinical
No

Prevention
No

Prognostic
No

Prospective meta-analysis (PMA)
No

Review of reviews
Yes

Service delivery
Yes

Synthesis of qualitative studies
No

Systematic review
Yes

Other
No

Health area of the review

Alcohol/substance misuse/abuse
No

Blood and immune system
No

Cancer
No

Cardiovascular
No

Care of the elderly
No

Child health
No

Complementary therapies
No

Crime and justice
No

Dental
No

Digestive system
No

Ear, nose and throat
No

Education
No

Endocrine and metabolic disorders
No

Eye disorders
No

General interest

No

Genetics
No

Health inequalities/health equity
No

Infections and infestations
No

International development
No

Mental health and behavioural conditions
No

Musculoskeletal
No

Neurological
No

Nursing
No

Obstetrics and gynaecology
No

Oral health
No

Palliative care
No

Perioperative care
No

Physiotherapy
No

Pregnancy and childbirth
No

Public health (including social determinants of health)
Yes

Rehabilitation
No

Respiratory disorders
No

Service delivery
Yes

Skin disorders
No

Social care
No

Surgery
No

Tropical Medicine
No

Urological
No

Wounds, injuries and accidents
No

Violence and abuse
No

31. Language.

Select each language individually to add it to the list below, use the bin icon to remove any added in error.
English

There is not an English language summary

32. Country.

Select the country in which the review is being carried out from the drop down list. For multi-national collaborations select all the countries involved.

Portugal

33. Other registration details.

Give the name of any organisation where the systematic review title or protocol is registered (such as with The Campbell Collaboration, or The Joanna Briggs Institute) together with any unique identification number assigned. (N.B. Registration details for Cochrane protocols will be automatically entered). If extracted data will be stored and made available through a repository such as the Systematic Review Data Repository (SRDR), details and a link should be included here. If none, leave blank.

Supported by National Funds through FCT - Fundação para a Ciência e a Tecnologia within CINTESIS, R&D Unit (reference UID/IC/4255/2019).

34. Reference and/or URL for published protocol.

Give the citation and link for the published protocol, if there is one

Give the link to the published protocol.

Alternatively, upload your published protocol to CRD in pdf format. Please note that by doing so you are consenting to the file being made publicly accessible.

No I do not make this file publicly available until the review is complete

Please note that the information required in the PROSPERO registration form must be completed in full even if access to a protocol is given.

35. Dissemination plans.

Give brief details of plans for communicating essential messages from the review to the appropriate audiences.

These results may result in an article for submission to an indexed scientific journal and/or oral communication in international congress.

The theme is pertinent to the academic community that seeks to standardize processes within care based on the best evidence available in clinical practice.

In addition to the publications, it is expected that this work will contribute to the scientific community, since it can be replicated in different clinical conditions and services, nationally and internationally.

It also aims to help strengthen the field of study and provide relevant information to other researchers and health decision makers.

Do you intend to publish the review on completion?

Yes

36. Keywords.

Give words or phrases that best describe the review. Separate keywords with a semicolon or new line. Keywords will help users find the review in the Register (the words do not appear in the public record but are included in searches). Be as specific and precise as possible. Avoid acronyms and abbreviations unless these are in wide use.

Quality indicators

Primary health care

Systematic review

Family practice

Community health

37. Details of any existing review of the same topic by the same authors.

Give details of earlier versions of the systematic review if an update of an existing review is being registered, including full bibliographic reference if possible.

38. * Current review status.

Review status should be updated when the review is completed and when it is published. For newregistrations the review must be Ongoing.
Please provide anticipated publication date

Review_Ongoing

39. Any additional information.

Provide any other information the review team feel is relevant to the registration of the review.

40. Details of final report/publication(s).

This field should be left empty until details of the completed review are available.

Give the link to the published review.
