# Supplementary material for: Primary health care quality indicators: An umbrella review
Source: PLoS One. 2019 Aug 16;14(8):e0220888. doi: 10.1371/journal.pone.0220888 (PMC6697344; doi:10.1371/journal.pone.0220888)
Supplement: S3 File — (PDF) [file pone.0220888.s003.pdf]

Supporting Information 3 – Search Expression

#1 - "systematic review"[Title/Abstract]
#2 - "systematic review"[Publication Type]
#3 - meta analysis [MeSH Terms]
#4 - "meta analysis" [Title/Abstract]
#5 - "meta analysis"[Publication Type]
#6 - #1 OR #2 OR #3 OR #4 OR #5
#7 – “primary health care” [Title/Abstract]
#8 – “primary healthcare” [Title/Abstract]
#9 – “primary care” [Title/Abstract]
#10 – “general practice” [Title/Abstract]
#11 – “family medicine” [Title/Abstract]
#12 – “community medicine” [Title/Abstract]
#13 – “family health” [Title/Abstract]
#14 – “community health” [Title/Abstract]
#15 – “community care” [Title/Abstract]
#16 – “family care” [Title/Abstract]
#17 – “ambulatory care” [Title/Abstract]
#18 - #7 OR #8 OR #9 OR #10 OR #11 OR #12 OR #13 OR #14 OR #15 OR #16 OR #17
#19 – indicator*[Title/Abstract]
#20 – quality [Title/Abstract]
#21 – (#6 OR #18)
#22 – #21 AND #19 AND #20.
