# Supplementary material for: Primary health care quality indicators: An umbrella review
Source: PLoS One. 2019 Aug 16;14(8):e0220888. doi: 10.1371/journal.pone.0220888 (PMC6697344; doi:10.1371/journal.pone.0220888)
Supplement: S1 Appendix — (PDF) [file pone.0220888.s005.pdf]

QUALITY INDICATORS SETLIST

Article ID	Indicator ID	Indicator Name	Dimension of Care	Type of Care	Function	Domain	Context	Description	Reference
4	1	Follow-up contacts during treatment episode after initial evaluation	Process	Chronic	Follow up and continuity	Effective	P - Psychological	Number of follow-up contacts during treatment episode after initial evaluation of bipolar case defined as the presence of a documented diagnosis of bipolar disorder by the consulting psychiatrist, primary care physician, or care manager, and a positive result on the semi-structured Composite International Diagnostic Interview Version 3.0	1. Cerimele JM, Chan Y-F, Chwastiak LA, et al. Bipolar disorder in primary care: clinical characteristics of 740 primary care patients with bipolar disorder. Psychiatr Serv 2014; 65(8): 1041–1046 || 2. Kronenberg C, Doran T, Goddard M, Kendrick T, Gilbody S, Dare CR, et al. Identifying primary care quality indicators for people with serious mental illness: a systematic review. The British journal of general practice : the journal of the Royal College of General Practitioners. 2017;67(661):e519-e30.
4	2	Any follow-up contact within 2 weeks after initial assessment	Process	Chronic	Follow up and continuity	Effective	P - Psychological	Any follow-up contact within 2 weeks after initial assessment of bipolar case defined as the presence of a documented diagnosis of bipolar disorder by the consulting psychiatrist, primary care physician, or care manager, and a positive result on the semi-structured Composite International Diagnostic Interview Version 3.0	1. Cerimele JM, Chan Y-F, Chwastiak LA, et al. Bipolar disorder in primary care: clinical characteristics of 740 primary care patients with bipolar disorder. Psychiatr Serv 2014; 65(8): 1041–1047 || 2. Kronenberg C, Doran T, Goddard M, Kendrick T, Gilbody S, Dare CR, et al. Identifying primary care quality indicators for people with serious mental illness: a systematic review. The British journal of general practice : the journal of the Royal College of General Practitioners. 2017;67(661):e519-e30.
4	3	Any follow-up contact within 4 weeks after initial assessment	Process	Chronic	Follow up and continuity	Effective	P - Psychological	Any follow-up contact within 4 weeks after initial assessment of bipolar case defined as the presence of a documented diagnosis of bipolar disorder by the consulting psychiatrist, primary care physician, or care manager, and a positive result on the semi-structured Composite International Diagnostic Interview Version 3.0	1. Cerimele JM, Chan Y-F, Chwastiak LA, et al. Bipolar disorder in primary care: clinical characteristics of 740 primary care patients with bipolar disorder. Psychiatr Serv 2014; 65(8): 1041–1048 || 2. Kronenberg C, Doran T, Goddard M, Kendrick T, Gilbody S, Dare CR, et al. Identifying primary care quality indicators for people with serious mental illness: a systematic review. The British journal of general practice : the journal of the Royal College of General Practitioners. 2017;67(661):e519-e30.
4	4	Crisis management and out-of-hours services	Structure	Acute	All	Effective	P - Psychological	Existence of a crisis plan and management, including out-of-hours attendence	1. Ware NC, Dickey B, Tugenberg T, McHorney CA. CONNECT: a measure of continuity of care in mental health services. Ment Health Serv Res 2003; 5(4): 209–221. || 2. Kronenberg C, Doran T, Goddard M, Kendrick T, Gilbody S, Dare CR, et al. Identifying primary care quality indicators for people with serious mental illness: a systematic review. The British journal of general practice : the journal of the Royal College of General Practitioners. 2017;67(661):e519-e30. || 3. Johansen, I. H., Morken, T., & Hunskaar, S. (2010). Contacts related to mental illness and substance abuse in primary health care: A cross-sectional study comparing patients' use of daytime versus out-of-hours primary care in Norway. Scandinavian Journal of Primary Health Care, 28(3), 160–165. doi: 10.3109/02813432.2010.493310 || 4. Parmar A, Kaloiya G. Comorbidity of Personality Disorder among Substance Use Disorder Patients: A Narrative Review. Indian J Psychol Med. 2018;40(6):517-527.
4	5	Waiting time to treatment	Process	All	Treatment	Timely	P - Psychological	Waiting time between registration and start of treatment	1. Kronenberg C, Doran T, Goddard M, Kendrick T, Gilbody S, Dare CR, et al. Identifying primary care quality indicators for people with serious mental illness: a systematic review. The British journal of general practice : the journal of the Royal College of General Practitioners. 2017;67(661):e519-e30. || 2. Parameswaran SG, Spaeth-Rublee B, Pincus HA. Measuring the quality of mental health care: consensus perspectives from selected industrialized countries. Adm Policy Ment Health 2015; 42(3): 288–295.
4	6	Examination in patients with new treatment episode	Process	All	Treatment	Effective	P - Psychological	Number of patients that had a comprehensive mental status examination and history conducted in a new treatment episode.	1. Kronenberg C, Doran T, Goddard M, Kendrick T, Gilbody S, Dare CR, et al. Identifying primary care quality indicators for people with serious mental illness: a systematic review. The British journal of general practice : the journal of the Royal College of General Practitioners. 2017;67(661):e519-e30.
6	7	Urinary incontinence during initial dementia evaluation	Process	Acute	Screening and prevention	Effective	P - Psychological	Vulnerable elders that should have documentation of the presence or absence of urinary incontinence during the initial evaluation and annually	1. Fujita K., Moles RJ, Chen TF. Quality indicators for responsible use of medicines: a systematic review BMJ Open 2018;8:e020437. doi: 10.1136/bmjopen-2017-020437 || 2. Kröger E, Tourigny A, Morin D, et al. Selecting process quality indicators for the integrated care of vulnerable older adults affected by cognitive impairment or dementia. BMC Health Serv Res 2007;7:195. || 3. Chin WY, Lam CL, Lo SV. Quality of care of nurse-led and allied health personnel-led primary care clinics. Hong Kong medical journal = Xianggang yi xue za zhi. 2011;17(3):217-30.
1 [24]	8	Antidepressant medication management: effective acute phase treatment	Process	Acute	Treatment	Effective	P - Psychological	Percentage of patients 18 years of age and older as of April 30 of the measurement year, who were diagnosed with a new episode of depression, were treated with antidepressant medication, and remained on an antidepressant drug during the entire 84-day (12-week) acute treatment phase.	Friedberg MW, Coltin KL, Pearson SD, Kleinman KP, Zheng J, Singer JA, et al: Does affiliation of physician groups with one another produce higher quality primary care? J Gen Intern Med 2007, 22:1385-1392. // Kringos DS, Boerma WG, Hutchinson A, van der Zee 465 J, Groenewegen PP. The breadth of primary care: a systematic literature review of its core dimensions. 467 BMC Health Serv Res. 2010;10:65. Published 2010 Mar 13. doi:10.1186/1472-468 6963-10-65
1 [24]	9	Antidepressant medication management: optimal practitioner contacts during acute phase	Process	Acute	Follow up and continuity	Effective	P - Psychological	Percentage of members 18 years of age and older as of April 30 of the measurement year, who were diagnosed with a new episode of depression and treated with antidepressant medication, and who had at least 3 followup contacts with a non-mental health practitioner or mental health practitioner coded with a mental health diagnosis during the 84-day (12-week) acute treatment phase.	Friedberg MW, Coltin KL, Pearson SD, Kleinman KP, Zheng J, Singer JA, et al: Does affiliation of physician groups with one another produce higher quality primary care? J Gen Intern Med 2007, 22:1385-1392. // Kringos DS, Boerma WG, Hutchinson A, van der Zee J, Groenewegen PP. The breadth of primary care: a systematic literature review of its core dimensions. BMC Health Serv Res. 2010;10:65. Published 2010 Mar 13. doi:10.1186/1472-6963-10-65
12	10	Rate of Clostridium difficile infections	Outcome	Acute	Diagnosis	Safe	D - Digestive	Percentage of Clostridium difficile infections	2.Balsells, E., Shi, T., Leese, C., Lyell, I., Burrows, J., Wiuff, C., … Nair, H. (2018). Global burden of Clostridium difficile infections: a systematic review and meta-analysis. Journal of global health, 9(1), 010407. doi:10.7189/jogh.09.010407
15	11	Prescription of a penicillin-containing preparation to a patient with a history of allergy to penicillin	Process	Acute	Treatment	Safe	A - General and unspecified	Number of cases with prescription of penicillin-containing preparation and with a history of allergy to penicillin	4. Bhattacharya S. (2010). The facts about penicillin allergy: a review. Journal of advanced pharmaceutical technology & research, 1(1), 11–17.
15	12	Prescription of clarithromycin or erythromycin to a patient who is also receiving simvastatin, with no evidence that the patient has been advised to stop the simvastatin while taking the antibiotic	Process	Acute	Treatment	Safe	A - General and unspecified	Number of cases with prescription of clarithromycin or erythromycin to a patient who is also receiving simvastatin, with no evidence that the patient has been advised to stop the simvastatin while taking the antibiotic	5.Spencer, R. and Serumaga, B. (2011), Concurrent macrolide and statin – a common interaction. Prescriber, 22: 49-50. doi:10.1002/psb.796
22	13	Antibiotics prescribed for (most) bacterial infections	Process	Acute	Treatment	Effective	A - General and unspecified	Number of female patients older than 18 years old (yo) diagnosed with cystitis or other urinary infection prescribed antibacterial for systemic use	6. Le Maréchal, M., Tebano, G., Monnier, A. A., Adriaenssens, N., Gyssens, I. C., Huttner, B., … DRIVE-AB WP1 group (2018). Quality indicators assessing antibiotic use in the outpatient setting: a systematic review followed by an international multidisciplinary consensus procedure. The Journal of antimicrobial chemotherapy, 73(suppl_6), vi40–vi49. doi:10.1093/jac/dky117
22	14	Antibiotics prescribed for (most) bacterial infections	Process	Acute	Treatment	Effective	A - General and unspecified	Number of patients aged between 18 and 65 yo diagnosed with pneumonia prescribed antibacterial for systemic use	6. Le Maréchal, M., Tebano, G., Monnier, A. A., Adriaenssens, N., Gyssens, I. C., Huttner, B., … DRIVE-AB WP1 group (2018). Quality indicators assessing antibiotic use in the outpatient setting: a systematic review followed by an international multidisciplinary consensus procedure. The Journal of antimicrobial chemotherapy, 73(suppl_6), vi40–vi49. doi:10.1093/jac/dky117
22	15	Antibiotics prescribed for (most) viral infections or self-limiting bacterial infections	Process	Acute	Treatment	Effective	A - General and unspecified	Number of patients aged between 18 and 75 yo diagnosed with acute bronchitis or bronchiolitis prescribed antibacterial for systemic use	6. Le Maréchal, M., Tebano, G., Monnier, A. A., Adriaenssens, N., Gyssens, I. C., Huttner, B., … DRIVE-AB WP1 group (2018). Quality indicators assessing antibiotic use in the outpatient setting: a systematic review followed by an international multidisciplinary consensus procedure. The Journal of antimicrobial chemotherapy, 73(suppl_6), vi40–vi49. doi:10.1093/jac/dky117
22	16	Outpatients receive antibiotic therapy compliant with guidelines; this includes, but is not limited to indication, choice of the antibiotic, duration, dose and timing	Process	Acute	Treatment	Effective	A - General and unspecified	Number of patients older than 18 yo diagnosed with cystitis or other urinary infection prescribed the recommended antibacterial	6. Le Maréchal, M., Tebano, G., Monnier, A. A., Adriaenssens, N., Gyssens, I. C., Huttner, B., … DRIVE-AB WP1 group (2018). Quality indicators assessing antibiotic use in the outpatient setting: a systematic review followed by an international multidisciplinary consensus procedure. The Journal of antimicrobial chemotherapy, 73(suppl_6), vi40–vi49. doi:10.1093/jac/dky117
22	17	Acute upper respiratory infections and bronchitis should not be treated with antibiotics within the first three days, unless there is documented indication for treatment	Process	Acute	Treatment	Effective	A - General and unspecified	Delayed antibiotics prescribing strategy should be agreed for patients with the included conditions	6. Le Maréchal, M., Tebano, G., Monnier, A. A., Adriaenssens, N., Gyssens, I. C., Huttner, B., … DRIVE-AB WP1 group (2018). Quality indicators assessing antibiotic use in the outpatient setting: a systematic review followed by an international multidisciplinary consensus procedure. The Journal of antimicrobial chemotherapy, 73(suppl_6), vi40–vi49. doi:10.1093/jac/dky117
22	18	Outpatients with acute tonsillitis/pharyngitis should undergo a group A streptococcal diagnostic test to decide whether or not they should receive antibiotics	Process	Acute	Diagnosis	Effective	A - General and unspecified	Patients with a group A Streptococcus test	6. Le Maréchal, M., Tebano, G., Monnier, A. A., Adriaenssens, N., Gyssens, I. C., Huttner, B., … DRIVE-AB WP1 group (2018). Quality indicators assessing antibiotic use in the outpatient setting: a systematic review followed by an international multidisciplinary consensus procedure. The Journal of antimicrobial chemotherapy, 73(suppl_6), vi40–vi49. doi:10.1093/jac/dky117
22	19	Outpatients with an acute tonsillitis/pharyngitis and positive group A streptococcal diagnostic test should be treated with antibiotics	Process	Acute	Treatment	Effective	R - Respiratory	Patients with acute tonsillitis or pharyngitis and a positive Streptococcus A test treated with Antibiotics	6. Le Maréchal, M., Tebano, G., Monnier, A. A., Adriaenssens, N., Gyssens, I. C., Huttner, B., … DRIVE-AB WP1 group (2018). Quality indicators assessing antibiotic use in the outpatient setting: a systematic review followed by an international multidisciplinary consensus procedure. The Journal of antimicrobial chemotherapy, 73(suppl_6), vi40–vi49. doi:10.1093/jac/dky117
22	20	Antibiotics for an acute tonsillitis/pharyngitis should be withheld, discontinued or not prescribed if an outpatient presents a diagnostic test (rapid antigen test or throat culture) negative for group A streptococci	Process	Acute	Treatment	Effective	R - Respiratory	Patients with diagnostic test negative for group A streptococci where antimicrobial therapy is not prescribed, withhold or discontinued	6. Le Maréchal, M., Tebano, G., Monnier, A. A., Adriaenssens, N., Gyssens, I. C., Huttner, B., … DRIVE-AB WP1 group (2018). Quality indicators assessing antibiotic use in the outpatient setting: a systematic review followed by an international multidisciplinary consensus procedure. The Journal of antimicrobial chemotherapy, 73(suppl_6), vi40–vi49. doi:10.1093/jac/dky117
22	21	Possible contraindications should be taken into account when antibiotics are prescribed	Process	Acute	Treatment	Safe	A - General and unspecified	Prescription of Clarithromycin or erythromycin to a patient who is also receiving simvastatin, with no evidence that the patient has been advised to stop the simvastatin while taking the Antibiotics	6. Le Maréchal, M., Tebano, G., Monnier, A. A., Adriaenssens, N., Gyssens, I. C., Huttner, B., … DRIVE-AB WP1 group (2018). Quality indicators assessing antibiotic use in the outpatient setting: a systematic review followed by an international multidisciplinary consensus procedure. The Journal of antimicrobial chemotherapy, 73(suppl_6), vi40–vi49. doi:10.1093/jac/dky117
30	22	Amoxicillin percentage on the consumption of amoxicillin and amoxicillin/ clavulanic acid	Process	Acute	Treatment	Effective	A - General and unspecified	Amoxicillin percentage on the consumption of amoxicillin and amoxicillin + clavulanic acid	9. Fernández Urrusuno, Rocío & Flores Dorado, Macarena & Moreno-Campoy, Eva & Carmen Montero-Balosa, M. (2014). Selección de indicadores para la monitorización continua del impacto de programas de optimización de uso de antimicrobianos en Atención Primaria. Enfermedades Infecciosas y Microbiología Clínica. 33. 10.1016/j.eimc.2014.07.011.
2	23	Abortion services	Structure	Acute	Treatment	All	X - Female Genital	Abortion services	1. A. Mazur, C. D. Brindis e M. D. J. , "Assessing youth-friendly sexual and reproductive health services: a systematic review," BMC Health Services Research, pp. 1-12, 2018.
3	24	Accommodation "patient-focused on": Use of urgent appointments	Process	Acute	All	Effective	Not Defined	Number of urgent appointments	1. Kringos, D. S., Boerma, W. G., Hutchinson, A., van der Zee, J., & Groenewegen, P. P. (2010). The breadth of primary care: a systematic literature review of its core dimensions. BMC Health Services Research, 10(1) // 2. Ansari, Z. (2007). A Review of Literature on Access to Primary Health Care. Australian Journal of Primary Health, 13(2), 80.
3	25	Quality of health promotion: Gonorrhoea/chlamydia rates	Outcome	Acute	Screening and prevention	All	X - Female Genital / Y - Male Genital	Gonorrhoea/chlamydia rates	1. Kringos, D. S., Boerma, W. G., Hutchinson, A., van der Zee, J., & Groenewegen, P. P. (2010). The breadth of primary care: a systematic literature review of its core dimensions. BMC Health Services Research, 10(1) // 2. Marshall M, Klazinga N, Leatherman S, Hardy C, Bergmann E, Pisco L, et al: OECD Health Care Quality Indicator Project. The expert panel on primary care prevention and health promotion. Int J Qual Health Care 2006, 18(Suppl 1):21-25.
3	26	Preventive care: Blood typing and antibody screening for prenatal patients	Process	Preventive	Screening and prevention	Effective	W - Pregnancy, Childbearing, Family Planning	Preventive care: Blood typing and antibody screening for prenatal patients	1. Kringos, D. S., Boerma, W. G., Hutchinson, A., van der Zee, J., & Groenewegen, P. P. (2010). The breadth of primary care: a systematic literature review of its core dimensions. BMC Health Services Research, 10(1) // 2. Marshall M, Klazinga N, Leatherman S, Hardy C, Bergmann E, Pisco L, et al: OECD Health Care Quality Indicator Project. The expert panel on primary care prevention and health promotion. Int J Qual Health Care 2006, 18(Suppl 1):21-25.
3	27	Congestive heart failure (CHF) readmission rate	Outcome	Acute / Chronic	Treatment	Effective	K - Cardiovascular	Diagnosis and treatment - primary care: Congestive heart failure readmission rate	1. Kringos, D. S., Boerma, W. G., Hutchinson, A., van der Zee, J., & Groenewegen, P. P. (2010). The breadth of primary care: a systematic literature review of its core dimensions. BMC Health Services Research, 10(1) // 2. Marshall M, Klazinga N, Leatherman S, Hardy C, Bergmann E, Pisco L, et al: OECD Health Care Quality Indicator Project. The expert panel on primary care prevention and health promotion. Int J Qual Health Care 2006, 18(Suppl 1):21-25.
9	28	Potentially preventable hospitalisation clinical indicator of Acute confusion	Outcome	Acute / Preventive	Treatment	Effective	N - Neurological	1. Patient aged ≥65 years, 2. Use of two or more agents with anticholinergic activity OR use of an agent with high anticholinergic activity, 3. Use of multiple psychotropic medications (eg, benzodiazepines, tricyclic antidepressants)	1. Caughey, G. E., Kalisch Ellett, L. M., & Wong, T. Y. (2014). Development of evidence-based Australian medication-related indicators of potentially preventable hospitalisations: a modified RAND appropriateness method. BMJ Open, 4(4), e004625. // 2. Howard R, Avery A, Slavenburg S, et al. Which drugs cause preventable admissions to hospital? A systematic review. Br J Clin Pharmacol 2007;63:136–47.
9	29	Potentially preventable hospitalisation clinical indicator of acute coronary syndrome	Outcome	Acute / Preventive	Treatment	Effective	K - Cardiovascular	1. History of MI (in 2 years prior to admission), 2. Not on aspirin, β-blocker, ACEI or ARB and statin (in 3 months prior to admission) // OR // 1. Patient has coronary artery stent (in 1 year prior to admission) 2. No use of aspirin or clopidogrel (in 12 months prior to admission)	1. Caughey, G. E., Kalisch Ellett, L. M., & Wong, T. Y. (2014). Development of evidence-based Australian medication-related indicators of potentially preventable hospitalisations: a modified RAND appropriateness method. BMJ Open, 4(4), e004625. // 2. Howard R, Avery A, Slavenburg S, et al. Which drugs cause preventable admissions to hospital? A systematic review. Br J Clin Pharmacol 2007;63:136–47.
9	30	Potentially preventable hospitalisation clinical indicator of Arrhythmia	Outcome	Acute / Preventive	Treatment	Effective	K - Cardiovascular	1. Concurrent use of calcitriol with digoxin, 2. Calcium concentration not monitored in the previous 3 months	1. Caughey, G. E., Kalisch Ellett, L. M., & Wong, T. Y. (2014). Development of evidence-based Australian medication-related indicators of potentially preventable hospitalisations: a modified RAND appropriateness method. BMJ Open, 4(4), e004625. // 2. Howard R, Avery A, Slavenburg S, et al. Which drugs cause preventable admissions to hospital? A systematic review. Br J Clin Pharmacol 2007;63:136–47.
9	31	Potentially preventable hospitalisation clinical indicator of Asthma	Outcome	Acute / Preventive	Treatment	Effective	R - Respiratory	1. History of asthma 2. Use of SABA more than 3 times/week or use of LABA 3. No use of inhaled corticosteroids	1. Caughey, G. E., Kalisch Ellett, L. M., & Wong, T. Y. (2014). Development of evidence-based Australian medication-related indicators of potentially preventable hospitalisations: a modified RAND appropriateness method. BMJ Open, 4(4), e004625. // 2. Howard R, Avery A, Slavenburg S, et al. Which drugs cause preventable admissions to hospital? A systematic review. Br J Clin Pharmacol 2007;63:136–47.
9	32	Potentially preventable hospitalisation clinical indicator of Asthma or Chronic Obstructive Pulmonary Disease	Outcome	Acute / Preventive	Treatment	Effective	R - Respiratory	Potentially preventable hospitalisation clinical indicator of Asthma or Chronic Obstructive Pulmonary Disease: History of asthma or COPD	1. Caughey, G. E., Kalisch Ellett, L. M., & Wong, T. Y. (2014). Development of evidence-based Australian medication-related indicators of potentially preventable hospitalisations: a modified RAND appropriateness method. BMJ Open, 4(4), e004625. // 2. Howard R, Avery A, Slavenburg S, et al. Which drugs cause preventable admissions to hospital? A systematic review. Br J Clin Pharmacol 2007;63:136–47.
9	33	Potentially preventable hospitalisation clinical indicator of Cardiovascular disease	Outcome	Acute / Preventive	Treatment	Effective	K - Cardiovascular	1. History of diabetes 2. Not on lipid lowering drug	1. Caughey, G. E., Kalisch Ellett, L. M., & Wong, T. Y. (2014). Development of evidence-based Australian medication-related indicators of potentially preventable hospitalisations: a modified RAND appropriateness method. BMJ Open, 4(4), e004625. // 2. Howard R, Avery A, Slavenburg S, et al. Which drugs cause preventable admissions to hospital? A systematic review. Br J Clin Pharmacol 2007;63:136–47.
9	34	Potentially preventable hospitalisation clinical indicator of Chronic constipation or impaction	Outcome	Acute / Preventive	Treatment	Effective	D - Digestive	1. Use of two or more agents with low-to-moderate anticholinergic activity; OR use of a highly anticholinergic agent // 1. Regular use of a strong opioid analgesic (fentanyl, oxycodone, morphine) 2. No concurrent use of a laxative	1. Caughey, G. E., Kalisch Ellett, L. M., & Wong, T. Y. (2014). Development of evidence-based Australian medication-related indicators of potentially preventable hospitalisations: a modified RAND appropriateness method. BMJ Open, 4(4), e004625. // 2. Howard R, Avery A, Slavenburg S, et al. Which drugs cause preventable admissions to hospital? A systematic review. Br J Clin Pharmacol 2007;63:136–47.
9	35	Potentially preventable hospitalisation clinical indicator of chronic heart failure	Outcome	Acute / Preventive	Treatment	Effective	K - Cardiovascular	1. History of CHF (in 2 years prior to admission), 2. Not on an ACEI or ARB (in 3 months prior to admission // 1. History of CHF (in 2 years prior to admission) 2. Not on a heart failure indicated β-blocker (in 3 months prior to admission) // 1. History of CHF 2. Use of rosiglitazone or pioglitazone (in 6 months prior to admission) // 1. History of CHF 2. Use of NSAID (in 3 months prior to admission)	1. Caughey, G. E., Kalisch Ellett, L. M., & Wong, T. Y. (2014). Development of evidence-based Australian medication-related indicators of potentially preventable hospitalisations: a modified RAND appropriateness method. BMJ Open, 4(4), e004625. // 2. Howard R, Avery A, Slavenburg S, et al. Which drugs cause preventable admissions to hospital? A systematic review. Br J Clin Pharmacol 2007;63:136–47.
9	36	Potentially preventable hospitalisation clinical indicator of chronic heart failure and / or heart block	Outcome	Acute / Preventive	Treatment	Effective	K - Cardiovascular	1. History of CHF with heart block or advanced bradycardia (in 2 years prior to admission) 2. Use of digoxin (in 6 months prior to admission)	1. Caughey, G. E., Kalisch Ellett, L. M., & Wong, T. Y. (2014). Development of evidence-based Australian medication-related indicators of potentially preventable hospitalisations: a modified RAND appropriateness method. BMJ Open, 4(4), e004625. // 2. Howard R, Avery A, Slavenburg S, et al. Which drugs cause preventable admissions to hospital? A systematic review. Br J Clin Pharmacol 2007;63:136–47.
9	37	Potentially preventable hospitalisation clinical indicator of chronic heart failure or cardiac ischaemic event	Outcome	Acute / Preventive	Treatment	Effective	K - Cardiovascular	1. History of IHD (in 2 years prior to admission) 2. Use of rosiglitazone (in 6 months prior to admission)	1. Caughey, G. E., Kalisch Ellett, L. M., & Wong, T. Y. (2014). Development of evidence-based Australian medication-related indicators of potentially preventable hospitalisations: a modified RAND appropriateness method. BMJ Open, 4(4), e004625. // 2. Howard R, Avery A, Slavenburg S, et al. Which drugs cause preventable admissions to hospital? A systematic review. Br J Clin Pharmacol 2007;63:136–47.
9	38	Potentially preventable hospitalisation clinical indicator of chronic heart failure or myocardial infarction	Outcome	Acute / Preventive	Treatment	Effective	K - Cardiovascular	1. Concurrent use of insulin and rosiglitazone	1. Caughey, G. E., Kalisch Ellett, L. M., & Wong, T. Y. (2014). Development of evidence-based Australian medication-related indicators of potentially preventable hospitalisations: a modified RAND appropriateness method. BMJ Open, 4(4), e004625. // 2. Howard R, Avery A, Slavenburg S, et al. Which drugs cause preventable admissions to hospital? A systematic review. Br J Clin Pharmacol 2007;63:136–47.
9	39	Potentially preventable hospitalisation clinical indicator of Chronic Obstructive Pulmonary Disease	Outcome	Acute / Preventive	Treatment	Effective	R - Respiratory	1. Moderate to severe COPD with frequent exacerbation 2. Use of long-acting β-agonist or anticholinergic 3. No use of inhaled corticosteroids	1. Caughey, G. E., Kalisch Ellett, L. M., & Wong, T. Y. (2014). Development of evidence-based Australian medication-related indicators of potentially preventable hospitalisations: a modified RAND appropriateness method. BMJ Open, 4(4), e004625. // 2. Howard R, Avery A, Slavenburg S, et al. Which drugs cause preventable admissions to hospital? A systematic review. Br J Clin Pharmacol 2007;63:136–47.
9	40	Potentially preventable hospitalisation clinical indicator of Fracture	Outcome	Acute / Preventive	Treatment	Effective	L - Musculoskeletal	Female patient: 1. History of osteoporosis or fracture 2. No use of HRT, bisphosphonate, teriparatide, selective oestrogen receptor modulators or strontium // Male patient: 1. History of osteoporosis or fracture 2. No use of bisphosphonate or teriparatide // Patient aged ≥65 years: 1. History of osteoporosis 2. Patient not receiving adequate levels of calcium and vitamin D // Patient on high dose inhaled corticosteroid (≥400 μg fluticasone daily or equivalent) for more than 1 year: Bone mineral density not measured in the previous 24 months // Patient aged ≥65 years: 1. Use of a falls-risk medicine (eg, long-acting hypnotic or anxiolytic, tricyclic antidepressant)	1. Caughey, G. E., Kalisch Ellett, L. M., & Wong, T. Y. (2014). Development of evidence-based Australian medication-related indicators of potentially preventable hospitalisations: a modified RAND appropriateness method. BMJ Open, 4(4), e004625. // 2. Howard R, Avery A, Slavenburg S, et al. Which drugs cause preventable admissions to hospital? A systematic review. Br J Clin Pharmacol 2007;63:136–47.
9	41	Potentially preventable hospitalisation clinical indicator of gastrointestinal bleed, perforation or ulcer or gastritis	Outcome	Acute / Preventive	Treatment	Effective	D - Digestive	1. History of GI ulcer or bleeding 2. NSAID use for at least 1 month 3. No use of gastroprotective agent (eg, PPI)	1. Caughey, G. E., Kalisch Ellett, L. M., & Wong, T. Y. (2014). Development of evidence-based Australian medication-related indicators of potentially preventable hospitalisations: a modified RAND appropriateness method. BMJ Open, 4(4), e004625. // 2. Howard R, Avery A, Slavenburg S, et al. Which drugs cause preventable admissions to hospital? A systematic review. Br J Clin Pharmacol 2007;63:136–47.
9	42	Potentially preventable hospitalisation clinical indicator of gastrointestinal ulcer	Outcome	Acute / Preventive	Treatment	Effective	D - Digestive	1. Patient with dyspepsia 2. PPI not prescribed 3. Patient with a positive test for Helicobacter pylori 4. Not prescribed H pylori eradication therapy	1. Caughey, G. E., Kalisch Ellett, L. M., & Wong, T. Y. (2014). Development of evidence-based Australian medication-related indicators of potentially preventable hospitalisations: a modified RAND appropriateness method. BMJ Open, 4(4), e004625. // 2. Howard R, Avery A, Slavenburg S, et al. Which drugs cause preventable admissions to hospital? A systematic review. Br J Clin Pharmacol 2007;63:136–47.
9	43	Potentially preventable hospitalisation clinical indicator of gastrointestinal ulcer or bleed	Outcome	Acute / Preventive	Treatment	Effective	D - Digestive	1. Patient with osteoarthritis 2. Dispensed long-term NSAIDs (including COX-2) therapy	1. Caughey, G. E., Kalisch Ellett, L. M., & Wong, T. Y. (2014). Development of evidence-based Australian medication-related indicators of potentially preventable hospitalisations: a modified RAND appropriateness method. BMJ Open, 4(4), e004625. // 2. Howard R, Avery A, Slavenburg S, et al. Which drugs cause preventable admissions to hospital? A systematic review. Br J Clin Pharmacol 2007;63:136–47.
9	44	Potentially preventable hospitalisation clinical indicator of Hypercalcaemia	Outcome	Acute / Preventive	Treatment	Effective	T - Endocrine/Metabolic and Nutritional	1. Use of an oral hypoglycaemic agent, 2. HbA1c level not monitored in the previous 6 months	1. Caughey, G. E., Kalisch Ellett, L. M., & Wong, T. Y. (2014). Development of evidence-based Australian medication-related indicators of potentially preventable hospitalisations: a modified RAND appropriateness method. BMJ Open, 4(4), e004625. // 2. Howard R, Avery A, Slavenburg S, et al. Which drugs cause preventable admissions to hospital? A systematic review. Br J Clin Pharmacol 2007;63:136–47.
9	45	Potentially preventable hospitalisation clinical indicator of Hyperglycaemia/ hypoglycaemia	Outcome	Acute / Preventive	Treatment	Effective	T - Endocrine/Metabolic and Nutritional	1. Use of insulin, 2. HbA1c level not monitored in the previous 6 months	1. Caughey, G. E., Kalisch Ellett, L. M., & Wong, T. Y. (2014). Development of evidence-based Australian medication-related indicators of potentially preventable hospitalisations: a modified RAND appropriateness method. BMJ Open, 4(4), e004625. // 2. Howard R, Avery A, Slavenburg S, et al. Which drugs cause preventable admissions to hospital? A systematic review. Br J Clin Pharmacol 2007;63:136–47.
9	46	Potentially preventable hospitalisation clinical indicator of Hypoglycaemia	Outcome	Acute / Preventive	Treatment	Effective	T - Endocrine/Metabolic and Nutritional	1. Use of glibenclamide or glimepiride, 2. Renal function not monitored in the previous year, 3. Use of a long-acting oral hypoglycaemic agent (glibenclamide or glimepiride) 2. HbA1c level not monitored in the previous 6 months	1. Caughey, G. E., Kalisch Ellett, L. M., & Wong, T. Y. (2014). Development of evidence-based Australian medication-related indicators of potentially preventable hospitalisations: a modified RAND appropriateness method. BMJ Open, 4(4), e004625. // 2. Howard R, Avery A, Slavenburg S, et al. Which drugs cause preventable admissions to hospital? A systematic review. Br J Clin Pharmacol 2007;63:136–47.
9	47	Potentially preventable hospitalisation clinical indicator of Influenza-related pneumonia	Outcome	Acute / Preventive	Treatment	Effective	R - Respiratory	1. Patient aged ≥65 years 2. No contraindication to influenza vaccine 3. No influenza vaccine in the previous year	1. Caughey, G. E., Kalisch Ellett, L. M., & Wong, T. Y. (2014). Development of evidence-based Australian medication-related indicators of potentially preventable hospitalisations: a modified RAND appropriateness method. BMJ Open, 4(4), e004625. // 2. Howard R, Avery A, Slavenburg S, et al. Which drugs cause preventable admissions to hospital? A systematic review. Br J Clin Pharmacol 2007;63:136–47.
9	48	Potentially preventable hospitalisation clinical indicator of Ischaemic stroke	Outcome	Acute / Preventive	Treatment	Effective	K - Cardiovascular	1. History of chronic AF or ischaemic stroke (in 2 years prior to admission) 2. No use of warfarin or aspirin (in 3 months prior to admission)	1. Caughey, G. E., Kalisch Ellett, L. M., & Wong, T. Y. (2014). Development of evidence-based Australian medication-related indicators of potentially preventable hospitalisations: a modified RAND appropriateness method. BMJ Open, 4(4), e004625. // 2. Howard R, Avery A, Slavenburg S, et al. Which drugs cause preventable admissions to hospital? A systematic review. Br J Clin Pharmacol 2007;63:136–47.
9	49	Potentially preventable hospitalisation clinical indicator of Oesophagitis, oesophageal ulceration or stricture	Outcome	Acute / Preventive	Treatment	Effective	D - Digestive	1. History of oesophageal disorders (active oesophagitis, oesophageal ulceration, stricture or achalasia), 2. Use of alendronate	1. Caughey, G. E., Kalisch Ellett, L. M., & Wong, T. Y. (2014). Development of evidence-based Australian medication-related indicators of potentially preventable hospitalisations: a modified RAND appropriateness method. BMJ Open, 4(4), e004625. // 2. Howard R, Avery A, Slavenburg S, et al. Which drugs cause preventable admissions to hospital? A systematic review. Br J Clin Pharmacol 2007;63:136–47.

9	50	Potentially preventable hospitalisation clinical indicator of Osteoporosis or fracture	Outcome	Acute / Preventive	Treatment	Effective	L - Musculoskeletal	1. Use of systemic corticosteroids for at least 3 months, 2. No osteoporosis prophylaxis (women: no use of HRT, bisphosphonate, teriparatide, selective oestrogen receptor modulators or strontium; men: no use of bisphosphonate or teriparatide)	1. Caughey, G. E., Kalisch Ellett, L. M., & Wong, T. Y. (2014). Development of evidence-based Australian medication-related indicators of potentially preventable hospitalisations: a modified RAND appropriateness method. BMJ Open, 4(4), e004625. // 2. Howard R, Avery A, Slavenburg S, et al. Which drugs cause preventable admissions to hospital? A systematic review. Br J Clin Pharmacol 2007;63:136–47.
9	51	Potentially preventable hospitalisation clinical indicator of Pneumococcal pneumonia or sepsis	Outcome	Acute / Preventive	Treatment	Effective	R - Respiratory	1. Patient aged ≥65 years<br>2. No contraindication to pneumococcal vaccine<br>3. No pneumococcal vaccine in the previous 6 years	1. Caughey, G. E., Kalisch Ellett, L. M., & Wong, T. Y. (2014). Development of evidence-based Australian medication-related indicators of potentially preventable hospitalisations: a modified RAND appropriateness method. BMJ Open, 4(4), e004625. // 2. Howard R, Avery A, Slavenburg S, et al. Which drugs cause preventable admissions to hospital? A systematic review. Br J Clin Pharmacol 2007;63:136–47.
9	52	Potentially preventable hospitalisation clinical indicator of Renal failure	Outcome	Acute / Preventive	Treatment	Effective	U - Urological	1. NSAID use for >3 months, 2. Serum creatinine not monitored in the previous 12 months // 1. Use of lithium, 2. Serum creatinine not monitored in previous 6 months	1. Caughey, G. E., Kalisch Ellett, L. M., & Wong, T. Y. (2014). Development of evidence-based Australian medication-related indicators of potentially preventable hospitalisations: a modified RAND appropriateness method. BMJ Open, 4(4), e004625. // 2. Howard R, Avery A, Slavenburg S, et al. Which drugs cause preventable admissions to hospital? A systematic review. Br J Clin Pharmacol 2007;63:136–47.
9	53	Potentially preventable hospitalisation clinical indicator of Renal failure or nephropathy	Outcome	Acute / Preventive	Treatment	Effective	U - Urological	1. History of diabetes, 2. Microalbuminuria and plasma creatinine not monitored in the previous 12 months, 3. Patient not on ACEI or ARB	1. Caughey, G. E., Kalisch Ellett, L. M., & Wong, T. Y. (2014). Development of evidence-based Australian medication-related indicators of potentially preventable hospitalisations: a modified RAND appropriateness method. BMJ Open, 4(4), e004625. // 2. Howard R, Avery A, Slavenburg S, et al. Which drugs cause preventable admissions to hospital? A systematic review. Br J Clin Pharmacol 2007;63:136–47.
9	54	Potentially preventable hospitalisation clinical indicator of Serotonin toxicity	Outcome	Acute / Preventive	Treatment	Effective	P - Psychological	Concurrent treatment with strong CYP1A2 inhibitors (eg, duloxetine) with fluvoxamine	1. Caughey, G. E., Kalisch Ellett, L. M., & Wong, T. Y. (2014). Development of evidence-based Australian medication-related indicators of potentially preventable hospitalisations: a modified RAND appropriateness method. BMJ Open, 4(4), e004625. // 2. Howard R, Avery A, Slavenburg S, et al. Which drugs cause preventable admissions to hospital? A systematic review. Br J Clin Pharmacol 2007;63:136–47.
9	55	Potentially preventable hospitalisation clinical indicator of Urinary retention	Outcome	Acute / Preventive	Treatment	Effective	U - Urological	1. History of BPH<br>2. Use of an anticholinergic agent	1. Caughey, G. E., Kalisch Ellett, L. M., & Wong, T. Y. (2014). Development of evidence-based Australian medication-related indicators of potentially preventable hospitalisations: a modified RAND appropriateness method. BMJ Open, 4(4), e004625. // 2. Howard R, Avery A, Slavenburg S, et al. Which drugs cause preventable admissions to hospital? A systematic review. Br J Clin Pharmacol 2007;63:136–47.
9	56	Potentially preventable hospitalisation clinical indicator of venous thromboembolism or stroke	Outcome	Acute / Preventive	Treatment	Effective	K - Cardiovascular	1. History of coronary artery disease or VTE<br>2. Use of raloxifene	1. Caughey, G. E., Kalisch Ellett, L. M., & Wong, T. Y. (2014). Development of evidence-based Australian medication-related indicators of potentially preventable hospitalisations: a modified RAND appropriateness method. BMJ Open, 4(4), e004625. // 2. Howard R, Avery A, Slavenburg S, et al. Which drugs cause preventable admissions to hospital? A systematic review. Br J Clin Pharmacol 2007;63:136–47.
11	57	Acute Health Services Use - Emergency Department Visits for Asthma	Outcome	Acute	Treatment	Effective	R - Respiratory	Acute Health Services Use - Emergency Department Visits for Asthma	To, T., Guttmann, A., Lougheed, M. D., Gershon, A. S., Dell, S. D., Stanbrook, M. B., … Fisman, D. N. (2010). Evidence-based performance indicators of primary care for asthma: a modified RAND Appropriateness Method. International Journal for Quality in Health Care, 22(6), 476–485.
11	58	Acute Health Services Use - Urgent Care Visits for Asthma	Outcome	Acute	Treatment	Effective	R - Respiratory	Acute Health Services Use - Urgent Care Visits for Asthma	To, T., Guttmann, A., Lougheed, M. D., Gershon, A. S., Dell, S. D., Stanbrook, M. B., … Fisman, D. N. (2010). Evidence-based performance indicators of primary care for asthma: a modified RAND Appropriateness Method. International Journal for Quality in Health Care, 22(6), 476–485.
16	59	Asthma: children attended in emergency care due to an asthma agudization and re-evaluated by their doctor within 72h	Outcome	Acute	Treatment	Effective	R - Respiratory	Asthma: children attended in emergency care due to an asthma agudization and re-evaluated by their doctor within 72h	Olry de Labry Lima, A., García Mochón, L., & Bermúdez Tamayo, C. (2017). Identificación de indicadores de resultado en salud en atención primaria. Una revisión de revisiones sistemáticas. Revista de Calidad Asistencial, 32(5), 278–288.
16	60	Cumulative hospitalization days in patients with chronic conditions	Outcome	Acute	Treatment	Efficient	A - General and unspecified	Cumulative hospitalization days in patients with chronic conditions	Olry de Labry Lima, A., García Mochón, L., & Bermúdez Tamayo, C. (2017). Identificación de indicadores de resultado en salud en atención primaria. Una revisión de revisiones sistemáticas. Revista de Calidad Asistencial, 32(5), 278–288.
16	61	Hospital care readmissions in patients with chronic conditions	Outcome	Acute	Treatment	Effective	A - General and unspecified	Hospital care readmissions in patients with chronic conditions	Olry de Labry Lima, A., García Mochón, L., & Bermúdez Tamayo, C. (2017). Identificación de indicadores de resultado en salud en atención primaria. Una revisión de revisiones sistemáticas. Revista de Calidad Asistencial, 32(5), 278–288.
16	62	Urgency care use by patients with chronic conditions	Outcome	Acute	Treatment	Efficient	A - General and unspecified	Urgency care use by patients with chronic conditions	Olry de Labry Lima, A., García Mochón, L., & Bermúdez Tamayo, C. (2017). Identificación de indicadores de resultado en salud en atención primaria. Una revisión de revisiones sistemáticas. Revista de Calidad Asistencial, 32(5), 278–288.
19	63	Asthma: Percentage of children hospitalised for asthma with a check-up before two weeks	Outcome	Acute	Treatment	Effective	R - Respiratory	Asthma: Percentage of children hospitalised for asthma with a check-up before two weeks	Ruiz-Canela-Cáceres, J., Aquino-Llinares, N., Sánchez-Diaz, J. M., García-Gestoso, M. L., de Jaime-Revuelta, M. E., & Praena-Crespo, M. (2015). Indicators for childhood asthma in Spain, using the Rand method. Allergologia et Immunopathologia, 43(2), 147–156.
19	64	Asthma: Percentage of children hospitalised in one year	Outcome	Acute	Treatment	Efficient	R - Respiratory	Asthma: Percentage of children hospitalised in one year	Ruiz-Canela-Cáceres, J., Aquino-Llinares, N., Sánchez-Diaz, J. M., García-Gestoso, M. L., de Jaime-Revuelta, M. E., & Praena-Crespo, M. (2015). Indicators for childhood asthma in Spain, using the Rand method. Allergologia et Immunopathologia, 43(2), 147–156.
19	65	Asthma: Percentage of children seen at ER due toan attack and reassessed bytheir doctor within 72 hours	Outcome	Acute	Treatment	Effective	R - Respiratory	Asthma: Percentage of children seen at ER due toan attack and reassessed bytheir doctor within 72 hours	Ruiz-Canela-Cáceres, J., Aquino-Llinares, N., Sánchez-Diaz, J. M., García-Gestoso, M. L., de Jaime-Revuelta, M. E., & Praena-Crespo, M. (2015). Indicators for childhood asthma in Spain, using the Rand method. Allergologia et Immunopathologia, 43(2), 147–156.
19	66	Asthma: Percentage of children with one or more visits to ER in a year	Outcome	Acute	Treatment	Efficient	R - Respiratory	Asthma: Percentage of children with one or more visits to ER in a year	Ruiz-Canela-Cáceres, J., Aquino-Llinares, N., Sánchez-Diaz, J. M., García-Gestoso, M. L., de Jaime-Revuelta, M. E., & Praena-Crespo, M. (2015). Indicators for childhood asthma in Spain, using the Rand method. Allergologia et Immunopathologia, 43(2), 147–156.
19	67	Asthma: Percentage of repeat visits to ER	Outcome	Acute	Treatment	Efficient	R - Respiratory	Asthma: Percentage of repeat visits to ER	Ruiz-Canela-Cáceres, J., Aquino-Llinares, N., Sánchez-Diaz, J. M., García-Gestoso, M. L., de Jaime-Revuelta, M. E., & Praena-Crespo, M. (2015). Indicators for childhood asthma in Spain, using the Rand method. Allergologia et Immunopathologia, 43(2), 147–156.
19	68	Asthma: Use of spirometry during attack	Outcome	Acute	Treatment	Effective	R - Respiratory	Asthma: Use of spirometry during attack	Ruiz-Canela-Cáceres, J., Aquino-Llinares, N., Sánchez-Diaz, J. M., García-Gestoso, M. L., de Jaime-Revuelta, M. E., & Praena-Crespo, M. (2015). Indicators for childhood asthma in Spain, using the Rand method. Allergologia et Immunopathologia, 43(2), 147–156.
4	69	Any psychiatric consultation during treatment	Process	Acute / Chronic	Follow up and continuity	Effective	P - Psychological	Any psychiatric consultation during treatment, either a psychiatrist reviewing a patient's case or an in-person consultation with the psychiatrist of bipolar case defined as the presence of a documented diagnosis of bipolar disorder by the consulting psychiatrist, primary care physician, or care manager, and a positive result on the semi-structured Composite International Diagnostic Interview Version 3.0	1. Cerimele JM, Chan Y-F, Chwastiak LA, et al. Bipolar disorder in primary care: clinical characteristics of 740 primary care patients with bipolar disorder. Psychiatr Serv 2014; 65(8): 1041–1050 || 2. Kronenberg C, Doran T, Goddard M, Kendrick T, Gilbody S, Dare CR, et al. Identifying primary care quality indicators for people with serious mental illness: a systematic review. The British journal of general practice : the journal of the Royal College of General Practitioners. 2017;67(661):e519-e30.
4	70	Duration of untreated psychosis	Outcome	Acute / Chronic	Treatment	Efficient	P - Psychological	Length of untreated psycosis	1. Kronenberg C, Doran T, Goddard M, Kendrick T, Gilbody S, Dare CR, et al. Identifying primary care quality indicators for people with serious mental illness: a systematic review. The British journal of general practice : the journal of the Royal College of General Practitioners. 2017;67(661):e519-e30. || 2. Parameswaran SG, Spaeth-Rublee B, Pincus HA. Measuring the quality of mental health care: consensus perspectives from selected industrialized countries. Adm Policy Ment Health 2015; 42(3): 288–295. || 3. Ruud T. Mental health quality and outcome measurement and improvement in Norway. Curr Opin Psychiatry 2009; 22(6): 631–635
4	71	Sedation side effects	Outcome	Acute / Chronic	Treatment	Safe	P - Psychological	Patients with neurological, sexual, sleeping, and sedation side effects	1. Kronenberg C, Doran T, Goddard M, Kendrick T, Gilbody S, Dare CR, et al. Identifying primary care quality indicators for people with serious mental illness: a systematic review. The British journal of general practice : the journal of the Royal College of General Practitioners. 2017;67(661):e519-e30. || 2. Mainz J, Hansen AM, Palshof T, Bartels PD. National quality measurement using clinical indicators: the Danish National Indicator Project. J Surg Oncol 2009; 99(8): 500–504.
4	72	Refferal to specialist mental health assessment	Process	Acute / Chronic	All	Effective	P - Psychological	Number of patients referral for specialist mental health assessment	1. Kronenberg C, Doran T, Goddard M, Kendrick T, Gilbody S, Dare CR, et al. Identifying primary care quality indicators for people with serious mental illness: a systematic review. The British journal of general practice : the journal of the Royal College of General Practitioners. 2017;67(661):e519-e30.
4	73	Comorbid psychiatric conditions and response to treatment	Outcome	Acute / Chronic	Treatment	Effective	P - Psychological	Patient assessed for comorbid psychiatric conditions and response to treatment	1. Kronenberg C, Doran T, Goddard M, Kendrick T, Gilbody S, Dare CR, et al. Identifying primary care quality indicators for people with serious mental illness: a systematic review. The British journal of general practice : the journal of the Royal College of General Practitioners. 2017;67(661):e519-e30.
4	74	Severity of symptoms	Outcome	Acute / Chronic	Diagnosis	Effective	P - Psychological	Number of patients with severity of symptoms reassessed	1. Kronenberg C, Doran T, Goddard M, Kendrick T, Gilbody S, Dare CR, et al. Identifying primary care quality indicators for people with serious mental illness: a systematic review. The British journal of general practice : the journal of the Royal College of General Practitioners. 2017;67(661):e519-e30.
4	75	Delayed diagnosis	Outcome	Acute / Chronic	Diagnosis	Timely	P - Psychological	Patients with delayed diagnosis of serious mental illness	1. Kronenberg C, Doran T, Goddard M, Kendrick T, Gilbody S, Dare CR, et al. Identifying primary care quality indicators for people with serious mental illness: a systematic review. The British journal of general practice : the journal of the Royal College of General Practitioners. 2017;67(661):e519-e30.
5	76	Antidepressant Use	Process	Acute / Chronic	Treatment	Effective	P - Psychological	Patients under prescription and antidepressant use	1. Duhoux A, Fournier L, Menear M. Quality Indicators for Depression Treatment in Primary Care: A Systematic Literature Review. Current Psychiatry Reviews (2011) 7: 104. https://doi.org/10.2174/157340011796391166
5	77	Dosage of antidepressants	Process	Acute / Chronic	Treatment	Effective	P - Psychological	Adequate dosage of antidepressant treatment	1. Duhoux A, Fournier L, Menear M. Quality Indicators for Depression Treatment in Primary Care: A Systematic Literature Review. Current Psychiatry Reviews (2011) 7: 104. https://doi.org/10.2174/157340011796391166
5	78	Duration of antidepressant treatment	Process	Chronic	Treatment	Effective	P - Psychological	Adequate duration of antidepressant treatment	1. Duhoux A, Fournier L, Menear M. Quality Indicators for Depression Treatment in Primary Care: A Systematic Literature Review. Current Psychiatry Reviews (2011) 7: 104. https://doi.org/10.2174/157340011796391166
5	79	Intensity of antidepressant treatment follow-up	Process	Chronic	Follow up and continuity	Effective	P - Psychological	Adequate intensity of antidepressant treatment follow-up	1. Duhoux A, Fournier L, Menear M. Quality Indicators for Depression Treatment in Primary Care: A Systematic Literature Review. Current Psychiatry Reviews (2011) 7: 104. https://doi.org/10.2174/157340011796391166
5	80	Psychotherapy use	Process	Chronic	Treatment	Effective	P - Psychological	Patients in psychotherapy use	1. Duhoux A, Fournier L, Menear M. Quality Indicators for Depression Treatment in Primary Care: A Systematic Literature Review. Current Psychiatry Reviews (2011) 7: 104. https://doi.org/10.2174/157340011796391166
5	81	Psychotherapy intensity use	Process	Chronic	Treatment	Effective	P - Psychological	Adequate intensity of psychotherapy	1. Duhoux A, Fournier L, Menear M. Quality Indicators for Depression Treatment in Primary Care: A Systematic Literature Review. Current Psychiatry Reviews (2011) 7: 104. https://doi.org/10.2174/157340011796391166
5	82	Psychotherapy lenght of visits	Process	Chronic	Treatment	Effective	P - Psychological	Adequate psychotherapy length of visits	1. Duhoux A, Fournier L, Menear M. Quality Indicators for Depression Treatment in Primary Care: A Systematic Literature Review. Current Psychiatry Reviews (2011) 7: 104. https://doi.org/10.2174/157340011796391166
5	83	Patients initiating depression treatment	Process	Acute / Chronic	Treatment	Effective	P - Psychological	Patients in treatment initiation (either pharmacotherapy or psychotherapy)	1. Duhoux A, Fournier L, Menear M. Quality Indicators for Depression Treatment in Primary Care: A Systematic Literature Review. Current Psychiatry Reviews (2011) 7: 104. https://doi.org/10.2174/157340011796391166
5	84	Professional profiles	Structure	All	All	Effective	P - Psychological	Physicians with basic or specific education on depression	1. Duhoux A, Fournier L, Menear M. Quality Indicators for Depression Treatment in Primary Care: A Systematic Literature Review. Current Psychiatry Reviews (2011) 7: 104. https://doi.org/10.2174/157340011796391166
6	85	Older adults with cognitive impairment or dementia	Outcome	Chronic	Diagnosis	Effective	P - Psychological	Number of vulnerable older adults affected by cognitive impairment/dementia and being treated in an integrated service system	1. Fujita K., Moles RJ, Chen TF. Quality indicators for responsible use of medicines: a systematic review BMJ Open 2018;8:e020437. doi: 10.1136/bmjopen-2017-020437 || 2. Kröger E, Tourigny A, Morin D, et al. Selecting process quality indicators for the integrated care of vulnerable older adults affected by cognitive impairment or dementia. BMC Health Serv Res 2007;7:195.
1[25,26]	86	Anti-depressants prescribed: % of the recommended	Process	Acute / Chronic	Treatment	Effective	P - Psychological	The prescription of a high proportion of anti depressants with a demonstrably high efficacy and safety; adherence to a selection of drugs recommended by evidence-based guidelines for the treatment of common primary health care mental problems	Friedberg MW, Coltin KL, Pearson SD, Kleinman KP, Zheng J, Singer JA, et al: Does affiliation of physician groups with one another produce higher quality primary care? J Gen Intern Med 2007, 22:1385-1392. // Kringos DS, Boerma WG, Hutchinson A, van der Zee 465 J, Groenewegen PP. 466 The breadth of primary care: a systematic literature review of its core dimensions. BMC Health Serv Res. 2010;10:65. Published 2010 Mar 13. doi:10.1186/1472-6963-10-65
1[25,26]	87	Tranquilisers prescribed: % of the recommended	Process	Acute / Chronic	Treatment	Effective	P - Psychological	The prescription of a high proportion of tranquilisers with a demonstrably high efficacy and safety; adherence to a selection of drugs recommended by evidence-based guidelines for the treatment of common primary health care mental problems	Friedberg MW, Coltin KL, Pearson SD, Kleinman KP, Zheng J, Singer JA, et al: Does affiliation of physician groups with one another produce higher quality primary care? J Gen Intern Med 2007, 22:1385-1392. // Kringos DS, Boerma WG, Hutchinson A, van der Zee 465 J, Groenewegen PP. The breadth of primary care: a systematic literature review of its core dimensions. BMC Health Serv Res. 2010;10:65. Published 2010 Mar 13. doi:10.1186/1472-6963-10-65
2	88	Antidepressant treatment	Process	Acute / Chronic	Treatment	Effective	P - Psychological	Patients under antidepressant treatment	Nuyen J, et al. The influence of specific chronic somatic conditions on the care for comorbid depression in general practice. Psychol Med 2008;38:265–77. // Sewitch MJ, et al. Cross-generational comparison of dispensed pharmacotherapy for depression. Int J Health Care Qual Assur 2009;22:300–12. // Kendrick T, et al. Management of depression in UK general practice in relation to scores on depression severity questionnaires: analysis of medical record data. BMJ 2009;338:b750. // Gill JM, Klinkman MS, Chen YX. Antidepressant medication use for primary care patients with and without medical comorbidities: a national electronic health record (EHR) network study. J Am Board Fam Med 2010;23:499–508.
2	89	Adequate pharmacotherapy (adequate treatment follow-up)	Process	Acute / Chronic	Follow up and continuity	Effective	P - Psychological	Adequate pharmacotherapy (adequate treatment follow-up)	Kurdyak PA, Gnam WH. Medication management of depression: the impact of comorbid chronic medical conditions. J Psychosom Res 2004;57:565–71 // .DeVeaugh-Geiss AM, West SL, Miller WC, Sleath B, Gaynes BN, Kroenke K. The adverse effects of comorbid pain on depression outcomes in primary care patients: results from the ARTIST trial. Pain Med 2010;11(5):732–41. // Jordan N, et al. Effect of care setting on evidence-based depression treatment for veterans with COPD and comorbid depression. J Gen Intern Med 2007;22: 1447–52.Dunn RL, Donoghue JM, Ozminkowski RJ, Stephenson D, Hylan TR. Longitudinal patterns of antidepressant prescribing in primary care in the UK: comparison with treatment guidelines. J Psychopharmacol 1999;13(2):136–43. // Bech P, et al. Association between clinically depressed subgroups, type of treatment and patient retention in the LIDO study. Psychol Med 2003;33:1051–9. // Simon GE, Von Korff M, Lin E. Clinical and functional outcomes of depression treatment in patients with and without chronic medical illness. Psychol Med 2005;35:271–9. // Ettner SL, et al. Association of general medical and psychiatric comorbidities with receipt of guideline-concordant care for depression. Psychiatr Serv 2010;<br>61:1255–9.
2	90	Minimally adequate treatment (adequate pharmacotherapy and/or adequate psychotherapy)	Process	Acute / Chronic	Treatment	Effective	P - Psychological	A minimum visits over a given time period and/or A minimum lenght of visits over a given time period (six or more psychotherapy sessions in the first 6 months after depression diagnosis)	Duhoux A, et al. Guideline concordance of treatment for depressive disorders in Canada. Soc Psychiatry Psychiatr Epidemiol 2009;44:385–92. // Unutzer J, et al. Depression treatment in a sample of 1,801 depressed older adults in primary care. J Am Geriatr Soc 2003;51:505–14. // Lagomasino IT, et al. Disparities in depression treatment for Latinos and site of care.<br>Psychiatr Serv 2005;56:1517–23. // Fernandez A, et al. Is major depression adequately diagnosed and treated by general practitioners? Results froman epidemiological study. Gen Hosp Psychiatry 2010;32: 201–9. // Boenisch S, et al. Who receives depression-specific treatment? A secondary databased analysis of outpatient care received by over 780,000 statutory healthinsured individuals diagnosed with depression. Soc Psychiatry Psychiatr Epidemiol 2012;47:475–86.
2	91	Treatment changes	Process	Acute / Chronic	Treatment	Patient-centered	P - Psychological	Number of prescription changes	Joo JH, Solano FX, Mulsant BH, Reynolds CF, Lenze EJ. Predictors of adequacy of depression management in the primary care setting. Psychiatr Serv 2005;56(12):1524–8. // Rost K, et al. The role of competing demands in the treatment provided primary care patients with major depression. Arch Fam Med 2000;9:150–4. // Wang PS, et al. Suboptimal antidepressant use in the elderly. J Clin Psychopharmacol 2005;25:118–26.
3	92	Prescription of a benzodiazepine or Z drug for ≥21 days, in a patient aged >65 years not receiveing BZD on a long term basis	Process	Acute / Chronic	Treatment	Safe	P - Psychological	Prescription of a benzodiazepine or Z drug for ≥21 days, in a patient aged >65 years, who is not receiving benzodiazepines or Z drugs on a long-term basis	Spencer R, Bell B, Avery AJ, Gookey G, Campbell SM. Royal College of<br>529 General Practitioners. Identification of an updated set of prescribing--safety<br>530 indicators for GPs. Br J Gen Pract. 2014;64(621):e181-90.
3	93	Initiation of prescription of benzodiazepine or Z drugs for ≥21 days in a patient aged >65 years with depression	Process	Acute / Chronic	Treatment	Safe	P - Psychological	Initiation of prescription of benzodiazepine or Z drugs for ≥21 days in a patient aged >65 years with depression	Spencer R, Bell B, Avery AJ, Gookey G, Campbell SM. Royal College of<br>529 General Practitioners. Identification of an updated set of prescribing--safety<br>530 indicators for GPs. Br J Gen Pract. 2014;64(621):e181-90.
3	94	Antipsychotics prescribed for >6 weeks in the over 65s with dementia but not psychosis	Process	Acute / Chronic	Treatment	Safe	P - Psychological	Antipsychotics prescribed for >6 weeks in the over 65s with dementia but not psychosis	Spencer R, Bell B, Avery AJ, Gookey G, Campbell SM. Royal College of<br>529 General Practitioners. Identification of an updated set of prescribing--safety<br>530 indicators for GPs. Br J Gen Pract. 2014;64(621):e181-90.
7	95	Access to depression and anxiety treatment	Process	Acute / Chronic	Treatment	Timely	P - Psychological	Access to services of psychological therapy and medicamentous treatment	Shield T, Campbell S, Rogers A, Worrall A, Chew-Graham<br>C, Gask L. Quality indicators for primary care mental health<br>services. Qual Saf Health Care 2003;12:100-6.
8	96	Percentage of patients with a new diagnosis of dementia with record of tests to exclude reversible cause	Process	Acute / Chronic	Diagnosis	Safe	P - Psychological	Patients with a new diagnosis of dementia that were submited to tests that excluded organic reasons.	Lake R, Georgiou A, Li J, Li L, Byrne M, Robinson M, et al. The quality, safety and governance of telephone triage and advice services - an overview of evidence from systematic reviews. BMC health services research. 2017;17(1):616
8	97	Percentage of patients with new diagnosis of depression with review soon after diagnosis	Process	Acute / Chronic	Treatment	Effective	P - Psychological	Patients with new diagnosis of depression with review soon after diagnosis	Lake R, Georgiou A, Li J, Li L, Byrne M, Robinson M, et al. The quality, safety and governance of telephone triage and advice services - an overview of evidence from systematic reviews. BMC health services research. 2017;17(1):617
4	98	Coordinated care	Process	Chronic	Follow up and continuity	Effective	P - Psychological	Coordinated care requires the identity of the current 'key-worker' (usually a social worker or community psychiatric nurse) to be available.	1. Holden J. An audit of the care of 266 patients with schizophrenia in 16 general practices. Irish J Psychologic Med 1998; 15(2): 61–63. || 2. Kronenberg C, Doran T, Goddard M, Kendrick T, Gilbody S, Dare CR, et al. Identifying primary care quality indicators for people with serious mental illness: a systematic review. The British journal of general practice : the journal of the Royal College of General Practitioners. 2017;67(661):e519-e30.
4	99	Staff continuity (turnover)	Structure	All	All	Effective	P - Psychological	Good communication between staff and infrequent staff change (turnover - replacing an employee with a new employee. A health organization's turnover is measured as a percentage rate, which is referred to as its turnover rate). Turnover rate is the percentage of employees in a workforce that leave during a certain period of time. Breakdowns between organizations and employees may consist of dismissal, retirement, death, inter-agency transfers, and waivers.	1. Sweeney A, Rose D, Clement S, et al. Understanding service user-defined continuity of care and its relationship to health and social measures: a crosssectional study. BMC Health Serv Res 2012; 12: 145. -- | 2. Warren B. Turnover: The Cost, the Causes, and a Sustainable Solution. http://cdn2.hubspot.net/hubfs/120635/HC_Turnover.pdf (acessed 21 Mar 2019) || 3. Kronenberg C, Doran T, Goddard M, Kendrick T, Gilbody S, Dare CR, et al. Identifying primary care quality indicators for people with serious mental illness: a systematic review. The British journal of general practice : the journal of the Royal College of General Practitioners. 2017;67(661):e519-e30.
4	100	Continuity of care	Process	Chronic	Follow up and continuity	Patient-centered	P - Psychological	Patient questionnaire (CONNECT) with 72 items, each rated on a five-point scale, with 13 scales and one single-item indicator: General coordination — 'Overall, is your mental health treatment well coordinated?' Primary care scales — 'How often is psychiatrist in contact with your primary care doctor?' The measure is administered in interview format.	1. Ware NC, Dickey B, Tugenberg T, McHorney CA. CONNECT: a measure of continuity of care in mental health services. Ment Health Serv Res 2003; 5(4): 209–221. || 2. Kronenberg C, Doran T, Goddard M, Kendrick T, Gilbody S, Dare CR, et al. Identifying primary care quality indicators for people with serious mental illness: a systematic review. The British journal of general practice : the journal of the Royal College of General Practitioners. 2017;67(661):e519-e30.
4	101	Register of all serious mental illness patients	Process	Chronic	Diagnosis	Effective	P - Psychological	Practice can produce register of all serious mental illness patients	1. Lester H, Tritter JQ, Sorohan H. Managing crisis: the role of primary care for people with serious mental illness. Fam Med 2004; 36(1): 28–34. || 2. Kronenberg C, Doran T, Goddard M, Kendrick T, Gilbody S, Dare CR, et al. Identifying primary care quality indicators for people with serious mental illness: a systematic review. The British journal of general practice : the journal of the Royal College of General Practitioners. 2017;67(661):e519-e30.
4	102	Service user registration with a primary health care service	Process	All	Diagnosis	Effective	A - General and unspecified	Service user registration with a primary health organisation	1. Parameswaran SG, Spaeth-Rublee B, Pincus HA. Measuring the quality of mental health care: consensus perspectives from selected industrialized countries. Adm Policy Ment Health 2015; 42(3): 288–295. || 2. Kronenberg C, Doran T, Goddard M, Kendrick T, Gilbody S, Dare CR, et al. Identifying primary care quality indicators for people with serious mental illness: a systematic review. The British journal of general practice : the journal of the Royal College of General Practitioners. 2017;67(661):e519-e30.
4	103	Markers of care recorded	Process	All	All	Effective	P - Psychological	Markers of care recorded: contact with secondary health services, written care plans, 6-month mental health review, identified care coordinator, evidence of physical examination	1. McCullagh M, Morley S, Dodwell D. A systematic, confidential approach to improving community care for patients with non-affective psychosis. Prim Care Psychiatr 2003; 8(4): 127–130. || 2. Kronenberg C, Doran T, Goddard M, Kendrick T, Gilbody S, Dare CR, et al. Identifying primary care quality indicators for people with serious mental illness: a systematic review. The British journal of general practice : the journal of the Royal College of General Practitioners. 2017;67(661):e519-e30.
4	104	Access to services	Process	All	All	Equitable	P - Psychological	Access to services (primary and mental health care) and range of services related	1. Sweeney A, Rose D, Clement S, et al. Understanding service user-defined continuity of care and its relationship to health and social measures: a crosssectional study. BMC Health Serv Res 2012; 12: 145 || 2. Kronenberg C, Doran T, Goddard M, Kendrick T, Gilbody S, Dare CR, et al. Identifying primary care quality indicators for people with serious mental illness: a systematic review. The British journal of general practice : the journal of the Royal College of General Practitioners. 2017;67(661):e519-e30.
4	105	Family care	Process	All	All	Effective	P - Psychological	Record of families living with person with schizophrenia	1. Holden J. An audit of the care of 266 patients with schizophrenia in 16 general practices. Irish J Psychologic Med 1998; 15(2): 61–63. || 2. Kronenberg C, Doran T, Goddard M, Kendrick T, Gilbody S, Dare CR, et al. Identifying primary care quality indicators for people with serious mental illness: a systematic review. The British journal of general practice : the journal of the Royal College of General Practitioners. 2017;67(661):e519-e30. || 2. Kronenberg C, Doran T, Goddard M, Kendrick T, Gilbody S, Dare CR, et al. Identifying primary care quality indic

4	106	Mental health review by General Practictioner	Process	Chronic	Follow up and continuity	Effective	P - Psychological	Percentage of patients given annual mental health review by General Practicioner	1. Kronenberg C, Doran T, Goddard M, Kendrick T, Gilbody S, Dare CR, et al. Identifying primary care quality indicators for people with serious mental illness: a systematic review. The British journal of general practice : the journal of the Royal College of General Practitioners. 2017;67(661):e519-e30.
4	107	Informal carer	Structure	Chronic	All	Effective	P - Psychological	If exists, the number of informal carer contacts	1. Kronenberg C, Doran T, Goddard M, Kendrick T, Gilbody S, Dare CR, et al. Identifying primary care quality indicators for people with serious mental illness: a systematic review. The British journal of general practice : the journal of the Royal College of General Practitioners. 2017;67(661):e519-e30.
4	108	Employment status	Structure	All	All	Patient-centered	P - Psychological	Information on employment status	1. Kronenberg C, Doran T, Goddard M, Kendrick T, Gilbody S, Dare CR, et al. Identifying primary care quality indicators for people with serious mental illness: a systematic review. The British journal of general practice : the journal of the Royal College of General Practitioners. 2017;67(661):e519-e30.
1	109	Prevalence of mental disorders	Outcome	Chronic	Diagnosis	Effective	P - Psychological	Patients with mental disorders in a moment of time in a population	Friedberg MW, Coltin KL, Pearson SD, Kleinman KP, Zheng J, Singer JA, et al: Does affiliation of physician groups with one another produce higher quality primary care? J Gen Intern Med 2007, 22:1385-1392. // Kringos DS, Boerma WG, Hutchinson A, van der Zee 465 J, Groenewegen PP. The breadth of primary care: a systematic literature review of its core dimensions. BMC Health Serv Res. 2010;10:65. Published 2010 Mar 13. doi:10.1186/1472-6963-10-65
7	110	Need for accessibility	Structure	All	Treatment	Timely	P - Psychological	Access to attendence including out-of-hours point of contact	Shield T, Campbell S, Rogers A, Worrall A, Chew-Graham C, Gask L. Quality indicators for primary care mental health services. Qual Saf Health Care 2003;12:100-6.
7	111	Practice policies and procedures	Process	All	All	Effective	Not Defined	If there are policies and standard procedures	Shield T, Campbell S, Rogers A, Worrall A, Chew-Graham C, Gask L. Quality indicators for primary care mental health services. Qual Saf Health Care 2003;12:100-6.
7	112	Information for patients and carers	Process	All	All	Patient-centered	P - Psychological	If there is given adequate information for patients and carers. Patients are given information about their condition, treatments, medication (including side effects) and coping strategies. Information (i.e. practice information leaflets, health promotion leaflets) is easy to understand and available in appropriate languages for patients and carers whose first language is not English	Shield T, Campbell S, Rogers A, Worrall A, Chew-Graham C, Gask L. Quality indicators for primary care mental health services. Qual Saf Health Care 2003;12:100-6.
7	113	Up-to-date and confidential medical record keeping	Process	All	Follow up and continuity	Patient-centered	Not Defined	Up-to-date and confidential medical record keeping	Shield T, Campbell S, Rogers A, Worrall A, Chew-Graham C, Gask L. Quality indicators for primary care mental health services. Qual Saf Health Care 2003;12:100-6.
7	114	Confidentiality and consent	Process	All	All	Patient-centered	Not Defined	Provide forms and settings for confidentiality and consent for patients under treatment	Shield T, Campbell S, Rogers A, Worrall A, Chew-Graham C, Gask L. Quality indicators for primary care mental health services. Qual Saf Health Care 2003;12:100-6.
8	115	Register of patients with dementia	Structure	Chronic	Diagnosis	Effective	P - Psychological	Number of patients registered with dementia diagnosis	Lake R, Georgiou A, Li J, Li L, Byrne M, Robinson M, et al. The quality, safety and governance of telephone triage and advice services - an overview of evidence from systematic reviews. BMC health services research. 2017;17(1):614
8	116	Register of patients with learning disability	Structure	Chronic	Diagnosis	Effective	P - Psychological	Number of patients registered with learning disability	Lake R, Georgiou A, Li J, Li L, Byrne M, Robinson M, et al. The quality, safety and governance of telephone triage and advice services - an overview of evidence from systematic reviews. BMC health services research. 2017;17(1):618
8	117	Register of patients with serious mental health problems	Structure	Chronic	Diagnosis	Effective	P - Psychological	Number of patients registered with serious mental problems	Lake R, Georgiou A, Li J, Li L, Byrne M, Robinson M, et al. The quality, safety and governance of telephone triage and advice services - an overview of evidence from systematic reviews. BMC health services research. 2017;17(1):619
8	118	Percentage of patients with serious mental health problems with comprehensive care plan	Process	Chronic	Treatment	Effective	P - Psychological	% of patients with serious mental health problems with comprehensive care plan	Lake R, Georgiou A, Li J, Li L, Byrne M, Robinson M, et al. The quality, safety and governance of telephone triage and advice services - an overview of evidence from systematic reviews. BMC health services research. 2017;17(1):620
32	119	Burdens in Oral Surgery Questionnaire (BiOS-Q)	Outcome	All	All	Patient-centered	D - Digestive	Patient satisfaction scale of the perceived burdens of the processes of dental treatment during oral surgical procedures	Reissmann DR, Semmusch J, Farhan D et al. Development and validation of the Burdens in Oral Surgery Questionnaire (BiOSQ). J Oral Rehabil 2013 40: 780–787.
32	120	Burdens in Prosthetic Dentistry Questionnaire (BiPD-Q)	Outcome	All	All	Patient-centered	D - Digestive	Patient satisfaction scale of the perceived burdens of the processes of dental treatment during prosthetic dental procedures	1. Heissmann DR, Hacker T, Farhan D et al. The Burdens in Prosthetic Dentistry Questionnaire (BiPD-Q): development and validation of a patient-based measure for process-related quality of care in prosthetic dentistry. Int J Prosthodont 2013 26: 250–259. // 2. Hacker T, Heydecke G, Reissmann DR. Impact of procedures during prosthodontic treatment on patients' perceived burdens. J Dent 2015 43: 51–57.
32	121	Dental Management Survey Brazil (Dimension 6) (DMS-BR)	Outcome	All	All	Safe	D - Digestive	Self-assessment tool for use by dentists and practice managers to assess the quality of safety and organisational aspects of dental care delivery	Gonzales PS, Martins IEF, Biazevic MG et al. Dental Management Survey Brazil (DMS-BR): creation and validation of a management instrument. Braz Oral Res 2017 31: e26.
32	122	Dental patient feedback on consultation skills (DPFCS)	Outcome	All	All	Patient-centered	D - Digestive	Patient satisfaction scale on the quality of information provided by the dentist to patients in consultations and the atmosphere of trust generated	Cheng BS, McGrath C, Bridges SM et al. Development and evaluation of a Dental Patient Feedback on Consultation skills (DPFC) measure to enhance communication. Community Dent Health 2015 32: 226–230. // Wong HM, Bridges SM, McGrath CP et al. Impact of prominent themes in clinician-patient conversations on caregiver's perceived quality of communication with paediatric dental visits. PLoS ONE 2017 12: e0169059.
32	123	Dental Satisfaction Questionnaire (DSQ)	Outcome	All	All	Patient-centered	D - Digestive	Patient satisfaction scale, assessing ease of access, communication and thoroughness of care	#Davies AR, Ware JE Jr. Measuring patient satisfaction with dental care. Soc Sci Med A 1981 15: 751–760. // Lee CT, Zhang S, Leung YY et al. Patients' satisfaction and prevalence of complications on surgical extraction of third molar. Patient Prefer Adherence 2015 9: 257–263. // Milgrom P, Spiekerman C, Grembowski D. Dissatisfaction with dental care among mothers of Medicaid-enrolled children. Community Dent Oral Epidemiol 2008 36: 451–458. // Skaret E, Berg E, Raadal M et al. Reliability and validity of the Dental Satisfaction Questionnaire in a population of 23-yearolds in Norway. Community Dent Oral Epidemiol 2004 32: 25–30. // Brennan DS, Gaughwin A, Spencer AJ. Differences in dimensions of satisfaction with private and public dental care among children. Int Dent J 2001 51: 77–82. // Mascarenhas AK. Patient satisfaction with the comprehensive care model of dental care delivery. J Dent Educ 2001 65: 1266–1271. // Chapko MK, Bergner M, Green K et al. Development and validation of a measure of dental patient satisfaction. Med Care 1985 23: 39–49.
32	124	Dental Visit Satisfaction Scale (DVSS)	Outcome	All	All	Patient-centered	D - Digestive	Patient Satisfaction scale, communication of oral health, rapport with dentist and comfort during treatment	#Corah NL, O'Shea RM, Pace LF et al. Development of a patient measure of satisfaction with the dentist: the Dental Visit Satisfaction Scale. J Behav Med 1984 7: 367–373. # Olausson M, Esfahani N, Ostlin J et al. Native-born versus foreign-born patients' perception of communication and care in Swedish dental service. Swed Dent J 2016 40: 91–100. // Sun N, Burnside G, Harris R. Patient satisfaction with care by dental therapists. Br Dent J 2010 208: E9; discussion 212–213. // Hakeberg M, Heidari E, Norinder M et al. A Swedish version of the Dental Visit Satisfaction Scale. Acta Odontol Scand 2000 58: 19–24. // Stouthard ME, Hartman CA, Hoogstraten J. Development of a Dutch version of the Dental Visit Satisfaction Scale. Community Dent Oral Epidemiol 1992 20: 351–353.
32	125	Quality from the Patient's Perspective Questionnaire	Outcome	All	All	Patient-centered	D - Digestive	Patient satisfaction scale regarding the communication, information given and environment of care deliver	Larsson BW, Bergstrom K. Adolescents' perception of the quality of orthodontic treatment. Scand J Caring Sci 2005 19: 95–101.
32	126	Survey of Organisational Aspects of Dental Care (SOADC)	Process	All	All	Effective	D - Digestive	Self-assessment tool of structural elements of the delivery of dental care, with focus on teamwork, leadership and the implementation of change within a practice	Goetz K, Hasse P, Szecsenyi J et al. Questionnaire for measuring organisational attributes in dental-care practices: psychometric properties and test-retest reliability. Int Dent J 2016 66: 93–98.
12	127	Patients' perceptions of hospital cleanliness and hand-washing among doctors and nurses	Process	All	All	Patient-centered	A - General and unspecified	Patients' perceptions of hospital cleanliness and hand-washing among doctors and nurses	3. Joshi, S.C. & Diwan, Vishal & Tamhankar, Ashok & Joshi, R & Shah, H & Sharma, Megha & Pathak, Ashish & Macaden, Ragini & Stålsby Lundborg, Cecilia. (2012). Qualitative study on perception of hand hygiene among hospital staff in a rural teaching hospital in India. The Journal of hospital infection. 80. 340-4. 10.1016/j.jhin.2011.12.017.
22	128	Prescribed antibiotics chosen from an essential list/formulary	Process	All	Treatment	Effective	A - General and unspecified	Percentage of drugs prescribed from essential drugs list or formulary	6. Le Maréchal, M., Tebano, G., Monnier, A. A., Adriaenssens, N., Gyssens, I. C., Huttner, B., … DRIVE-AB WP1 group (2018). Quality indicators assessing antibiotic use in the outpatient setting: a systematic review followed by an international multidisciplinary consensus procedure. The Journal of antimicrobial chemotherapy, 73(suppl_6), vi40–vi49. doi:10.1093/jac/dky117
22	129	Possible contraindications should be taken into account when antibiotics are prescribed	Process	All	Treatment	Effective	A - General and unspecified	Number of prescription including tetracyclines to a pregnant woman	6. Le Maréchal, M., Tebano, G., Monnier, A. A., Adriaenssens, N., Gyssens, I. C., Huttner, B., … DRIVE-AB WP1 group (2018). Quality indicators assessing antibiotic use in the outpatient setting: a systematic review followed by an international multidisciplinary consensus procedure. The Journal of antimicrobial chemotherapy, 73(suppl_6), vi40–vi49. doi:10.1093/jac/dky117
22	130	Standard antibiotic treatment guidelines available in health facilities	Process	All	Treatment	Effective	A - General and unspecified	Availability of Standard antibiotic treatment guidelines at public health facilities	6. Le Maréchal, M., Tebano, G., Monnier, A. A., Adriaenssens, N., Gyssens, I. C., Huttner, B., … DRIVE-AB WP1 group (2018). Quality indicators assessing antibiotic use in the outpatient setting: a systematic review followed by an international multidisciplinary consensus procedure. The Journal of antimicrobial chemotherapy, 73(suppl_6), vi40–vi49. doi:10.1093/jac/dky117
22	131	Health facilities should have access to the Summary of Product Characteristics of prescribed antibiotics, written in a local language	Process	All	Treatment	Effective	A - General and unspecified	Health facilities with access to the summary of product characteristics in a local language	6. Le Maréchal, M., Tebano, G., Monnier, A. A., Adriaenssens, N., Gyssens, I. C., Huttner, B., … DRIVE-AB WP1 group (2018). Quality indicators assessing antibiotic use in the outpatient setting: a systematic review followed by an international multidisciplinary consensus procedure. The Journal of antimicrobial chemotherapy, 73(suppl_6), vi40–vi49. doi:10.1093/jac/dky117
22	132	Antibiotics not to be sold without prescription	Process	All	Treatment	Safe	A - General and unspecified	Percentage of prescription medicines bought with no prescription	6. Le Maréchal, M., Tebano, G., Monnier, A. A., Adriaenssens, N., Gyssens, I. C., Huttner, B., … DRIVE-AB WP1 group (2018). Quality indicators assessing antibiotic use in the outpatient setting: a systematic review followed by an international multidisciplinary consensus procedure. The Journal of antimicrobial chemotherapy, 73(suppl_6), vi40–vi49. doi:10.1093/jac/dky117
22	133	Outpatients and Outpatient Parenteral Antibiotic Therapy patients with an antibiotic prescription should be educated on how to take it, on the dosage, on expected side effects, and on the natural history of the disease	Process	All	Treatment	Safe	A - General and unspecified	Patients knowledge of correct dosage	6. Le Maréchal, M., Tebano, G., Monnier, A. A., Adriaenssens, N., Gyssens, I. C., Huttner, B., … DRIVE-AB WP1 group (2018). Quality indicators assessing antibiotic use in the outpatient setting: a systematic review followed by an international multidisciplinary consensus procedure. The Journal of antimicrobial chemotherapy, 73(suppl_6), vi40–vi49. doi:10.1093/jac/dky117
30	134	Antibiotic percentage of first step antibiotics	Process	All	Treatment	Effective	A - General and unspecified	Antibiotic percentage of first step antibiotics	9. Fernández Urrusuno, Rocio & Flores Dorado, Macarena & Moreno-Campoy, Eva & Carmen Montero-Balosa, M. (2014). Selección de indicadores para la monitorización continua del impacto de programas de optimización de uso de antimicrobianos en Atención Primaria. Enfermedades Infecciosas y Microbiología Clínica. 33. 10.1016/j.eimc.2014.07.011.
30	135	Cephalosporin consumption rate	Process	All	Treatment	Effective	A - General and unspecified	Cephalosporin consumption rate	9. Fernández Urrusuno, Rocio & Flores Dorado, Macarena & Moreno-Campoy, Eva & Carmen Montero-Balosa, M. (2014). Selección de indicadores para la monitorización continua del impacto de programas de optimización de uso de antimicrobianos en Atención Primaria. Enfermedades Infecciosas y Microbiología Clínica. 33. 10.1016/j.eimc.2014.07.011.
30	136	Consumption rate of antibiotics for systemic use	Process	All	Treatment	Effective	A - General and unspecified	Consumption rate of antibiotics for systemic use	9. Fernández Urrusuno, Rocio & Flores Dorado, Macarena & Moreno-Campoy, Eva & Carmen Montero-Balosa, M. (2014). Selección de indicadores para la monitorización continua del impacto de programas de optimización de uso de antimicrobianos en Atención Primaria. Enfermedades Infecciosas y Microbiología Clínica. 33. 10.1016/j.eimc.2014.07.011.
30	137	Consumption rate of macrolides, lincosamides and streptogramins	Process	All	Treatment	Effective	A - General and unspecified	Consumption rate of macrolides, lincosamides and streptogramins	9. Fernández Urrusuno, Rocio & Flores Dorado, Macarena & Moreno-Campoy, Eva & Carmen Montero-Balosa, M. (2014). Selección de indicadores para la monitorización continua del impacto de programas de optimización de uso de antimicrobianos en Atención Primaria. Enfermedades Infecciosas y Microbiología Clínica. 33. 10.1016/j.eimc.2014.07.011.
30	138	Percentage of 3rd generation cephalosporins	Process	All	Treatment	Effective	A - General and unspecified	Percentage of 3rd generation cephalosporins	9. Fernández Urrusuno, Rocio & Flores Dorado, Macarena & Moreno-Campoy, Eva & Carmen Montero-Balosa, M. (2014). Selección de indicadores para la monitorización continua del impacto de programas de optimización de uso de antimicrobianos en Atención Primaria. Enfermedades Infecciosas y Microbiología Clínica. 33. 10.1016/j.eimc.2014.07.011.
0	139	Percentage of antibiotics of first step antibiotics in pediatrics (<15 years)	Process	All	Treatment	Effective	A - General and unspecified	Percentage of antibiotics of first step antibiotics in pediatrics (<15 years)	9. Fernández Urrusuno, Rocio & Flores Dorado, Macarena & Moreno-Campoy, Eva & Carmen Montero-Balosa, M. (2014). Selección de indicadores para la monitorización continua del impacto de programas de optimización de uso de antimicrobianos en Atención Primaria. Enfermedades Infecciosas y Microbiología Clínica. 33. 10.1016/j.eimc.2014.07.011.
30	140	Percentage of combinations of penicillins and beta-lactamase inhibitors	Process	All	Treatment	Effective	A - General and unspecified	Percentage of combinations of penicillins and beta-lactamase inhibitors	9. Fernández Urrusuno, Rocio & Flores Dorado, Macarena & Moreno-Campoy, Eva & Carmen Montero-Balosa, M. (2014). Selección de indicadores para la monitorización continua del impacto de programas de optimización de uso de antimicrobianos en Atención Primaria. Enfermedades Infecciosas y Microbiología Clínica. 33. 10.1016/j.eimc.2014.07.011. 10 .Lister, P. D. (2000), β-Lactamase Inhibitor Combinations with Extended-Spectrum Penicillins: Factors Influencing Antibacterial Activity against Enterobacteriaceae and Pseudomonas aeruginosa. Pharmacotherapy: The Journal of Human Pharmacology and Drug Therapy, 20: 213S-218S. doi:10.1592/phco.20.14.213S.35045
30	141	Percentage of fluoroquinolones	Process	All	Treatment	Effective	A - General and unspecified	Percentage of fluoroquinolones	9. Fernández Urrusuno, Rocio & Flores Dorado, Macarena & Moreno-Campoy, Eva & Carmen Montero-Balosa, M. (2014). Selección de indicadores para la monitorización continua del impacto de programas de optimización de uso de antimicrobianos en Atención Primaria. Enfermedades Infecciosas y Microbiología Clínica. 33. 10.1016/j.eimc.2014.07.011.
30	142	Percentage of macrolides	Process	All	Treatment	Effective	A - General and unspecified	Percentage of macrolides	9. Fernández Urrusuno, Rocio & Flores Dorado, Macarena & Moreno-Campoy, Eva & Carmen Montero-Balosa, M. (2014). Selección de indicadores para la monitorización continua del impacto de programas de optimización de uso de antimicrobianos en Atención Primaria. Enfermedades Infecciosas y Microbiología Clínica. 33. 10.1016/j.eimc.2014.07.011. 11. Sanchez, G. V., Shapiro, D. J., Hersh, A. L., Hicks, L. A., & Fleming-Dutra, K. E. (2017). Outpatient Macrolide Antibiotic Prescribing in the United States, 2008-2011. Open forum infectious diseases, 4(4), ofx220. doi:10.1093/ofid/ofx220
30	143	Percentage of second step antibiotics in pediatrics (<15 years)	Process	All	Treatment	Effective	A - General and unspecified	Percentage of second step antibiotics in pediatrics (<15 years)	9. Fernández Urrusuno, Rocio & Flores Dorado, Macarena & Moreno-Campoy, Eva & Carmen Montero-Balosa, M. (2014). Selección de indicadores para la monitorización continua del impacto de programas de optimización de uso de antimicrobianos en Atención Primaria. Enfermedades Infecciosas y Microbiología Clínica. 33. 10.1016/j.eimc.2014.07.011. 12. Rogawski, E. T., Platts-Mills, J. A., Seidman, J. C., John, S., Mahfuz, M., Ulak, M., … Guerrant, R. L. (2016). Use of antibiotics in children younger than two years in eight countries: a prospective cohort study. Bulletin of the World Health Organization, 95(1), 49–61. doi:10.2471/BLT.16.176123
30	144	Percentage of second-tier antibiotics	Process	All	Treatment	Effective	A - General and unspecified	Percentage of second-tier antibiotics	9. Fernández Urrusuno, Rocio & Flores Dorado, Macarena & Moreno-Campoy, Eva & Carmen Montero-Balosa, M. (2014). Selección de indicadores para la monitorización continua del impacto de programas de optimización de uso de antimicrobianos en Atención Primaria. Enfermedades Infecciosas y Microbiología Clínica. 33. 10.1016/j.eimc.2014.07.011. 12. Rogawski, E. T., Platts-Mills, J. A., Seidman, J. C., John, S., Mahfuz, M., Ulak, M., … Guerrant, R. L. (2016). Use of antibiotics in children younger than two years in eight countries: a prospective cohort study. Bulletin of the World Health Organization, 95(1), 49–61. doi:10.2471/BLT.16.176123
30	145	Percentage of third-tier antibiotics	Process	All	Treatment	Effective	A - General and unspecified	Percentage of third-tier antibiotics	9. Fernández Urrusuno, Rocio & Flores Dorado, Macarena & Moreno-Campoy, Eva & Carmen Montero-Balosa, M. (2014). Selección de indicadores para la monitorización continua del impacto de programas de optimización de uso de antimicrobianos en Atención Primaria. Enfermedades Infecciosas y Microbiología Clínica. 33. 10.1016/j.eimc.2014.07.011. 12. Rogawski, E. T., Platts-Mills, J. A., Seidman, J. C., John, S., Mahfuz, M., Ulak, M., … Guerrant, R. L. (2016). Use of antibiotics in children younger than two years in eight countries: a prospective cohort study. Bulletin of the World Health Organization, 95(1), 49–61. doi:10.2471/BLT.16.176123
30	146	Third-tier antibiotics in pediatrics (<15 years)	Process	All	Treatment	Effective	A - General and unspecified	Percentage of third-tier antibiotics in pediatrics (<15 years)	9. Fernández Urrusuno, Rocio & Flores Dorado, Macarena & Moreno-Campoy, Eva & Carmen Montero-Balosa, M. (2014). Selección de indicadores para la monitorización continua del impacto de programas de optimización de uso de antimicrobianos en Atención Primaria. Enfermedades Infecciosas y Microbiología Clínica. 33. 10.1016/j.eimc.2014.07.011. 12. Rogawski, E. T., Platts-Mills, J. A., Seidman, J. C., John, S., Mahfuz, M., Ulak, M., … Guerrant, R. L. (2016). Use of antibiotics in children younger than two years in eight countries: a prospective cohort study. Bulletin of the World Health Organization, 95(1), 49–61. doi:10.2471/BLT.16.176123
3	147	Informational continuity of care	Process	All	Follow up and continuity	Patient-centered	Not Defined	An organized collection of each patient's medical information readily available to any health care provider caring for the patient. This can be reached through medical record keeping, clinical support and referral systems	1. Kringos, D. S., Boerma, W. G., Hutchinson, A., van der Zee, J., & Groenewegen, P. P. (2010). The breadth of primary care: a systematic literature review of its core dimensions. BMC Health Services Research, 10(1) // 2. Engels Y, Campbell S, Dautzenberg M, van den HP, Brinkmann H, Szecsenyi J, et al: Developing a framework of, and quality indicators for, general practice management in Europe. Fam Pract 2005, 22:215-222. // 3. Hung DY: Improving the delivery of preventive care services. Manag Care Interface 2007, 20:38-44.// 4. McInnes DK, Saltman DC, Kidd MR: General practitioners' use of computers for prescribing and electronic health records: Results from a national survey. Med J Aust 2006, 185:88-91. // 5. Naithani S, Gulliford M, Morgan M: Patients' perceptions and experiences of 'continuity of care' in diabetes. Health Expect 2006, 9:118-129. // 6. Shi L, Starfield B, Xu J, Politzer R, Regan J: Primary care quality: community health center and health maintenance organization. South Med J 2003, 96:787-795. // 7. Shi L, Starfield B, Xu J, Politzer R, Regan J: Primary care quality: community health center and health maintenance organization. South Med J 2003, 96:787-795. // 8. Stokes T, Tarrant C, Mainous AG III, Schers H, Freeman G, Baker R: Continuity of care: Is the personal doctor still important? A survey of general practitioners and family physicians in England and Wales, the United States, and the Netherlands. Ann Fam Med 2005, 3:353-359. // 9.
3	148	Availability: Number of physicians per unit of population	Structure	All	All	Efficient	Not Defined	Number of physicians per unit of population	1. Kringos, D. S., Boerma, W. G., Hutchinson, A., van der Zee, J., & Groenewegen, P. P. (2010). The breadth of primary care: a systematic literature review of its core dimensions. BMC Health Services Research, 10(1) // 2. Ansari, Z. (2007). A Review of Literature on Access to Primary Health Care. Australian Journal of Primary Health, 13(2), 80.
3	149	Availability: Number of hospital beds per unit of population	Structure	All	All	Efficient	Not Defined	Number of hospital beds per unit of population	1. Kringos, D. S., Boerma, W. G., Hutchinson, A., van der Zee, J., & Groenewegen, P. P. (2010). The breadth of primary care: a systematic literature review of its core dimensions. BMC Health Services Research, 10(1) // 2. Ansari, Z. (2007). A Review of Literature on Access to Primary Health Care. Australian Journal of Primary Health, 13(2), 80.
3	150	Availability: Number of ambulances per unit of population, and per unit of geographic area	Structure	All	All	Effective	Not Defined	Number of ambulances per unit of population, and per unit of geographic area	1. Kringos, D. S., Boerma, W. G., Hutchinson, A., van der Zee, J., & Groenewegen, P. P. (2010). The breadth of primary care: a systematic literature review of its core dimensions. BMC Health Services Research, 10(1) // 2. Ansari, Z. (2007). A Review of Literature on Access to Primary Health Care. Australian Journal of Primary Health, 13(2), 80.
3	151	Accessibility: Accessibility/Remoteness Index of Australia Plus (ARIA+) derived from measures of road distance between populated localities and service centres	Structure	All	All	Effective	Not Defined	Measures of road distance between populated localities and service centres	1. Kringos, D. S., Boerma, W. G., Hutchinson, A., van der Zee, J., & Groenewegen, P. P. (2010). The breadth of primary care: a systematic literature review of its core dimensions. BMC Health Services Research, 10(1) // 2. Ansari, Z. (2007). A Review of Literature on Access to Primary Health Care. Australian Journal of Primary Health, 13(2), 80.
3	152	Accessibility: Software connecting latitude and longitude of patient post codes with those of hospital post codes allowing mean distance travelled to hospital by patients (children admitted ambulatory care sensitive conditions) to be calculated for individual localities	Structure	All	All	Effective	Not Defined	Software connecting latitude and longitude of patient post codes with those of hospital post codes allowing mean distance travelled to hospital by patients (children admitted ambulatory care sensitive conditions) to be calculated for individual localities	1. Kringos, D. S., Boerma, W. G., Hutchinson, A., van der Zee, J., & Groenewegen, P. P. (2010). The breadth of primary care: a systematic literature review of its core dimensions. BMC Health Services Research, 10(1) // 2. Ansari, Z. (2007). A Review of Literature on Access to Primary Health Care. Australian Journal of Primary Health, 13(2), 80.
3	153	Accommodation "patient-focused on": How early and how late a surgery opened for patient appointments	Process	All	Treatment	Patient-centered	Not Defined	How early and how late a surgery opened for patient appointments	1. Kringos, D. S., Boerma, W. G., Hutchinson, A., van der Zee, J., & Groenewegen, P. P. (2010). The breadth of primary care: a systematic literature review of its core dimensions. BMC Health Services Research, 10(1) // 2. Ansari, Z. (2007). A Review of Literature on Access to Primary Health Care. Australian Journal of Primary Health, 13(2), 80.
3	154	Accommodation "patient-focused on": Out-of-hours service	Structure	Acute	All	Patient-centered	Not Defined	Amount of out-of-hours used for the care	1. Kringos, D. S., Boerma, W. G., Hutchinson, A., van der Zee, J., & Groenewegen, P. P. (2010). The breadth of primary care: a systematic literature review of its core dimensions. BMC Health Services Research, 10(1) // 2. Ansari, Z. (2007). A Review of Literature on Access to Primary Health Care. Australian Journal of Primary Health, 13(2), 80.
3	155	Accommodation "patient-focused on": Practice-based triage services	Process	All	Screening and prevention	Patient-centered	Not Defined	Existence of a practice-based triage services	1. Kringos, D. S., Boerma, W. G., Hutchinson, A., van der Zee, J., & Groenewegen, P. P. (2010). The breadth of primary care: a systematic literature review of its core dimensions. BMC Health Services Research, 10(1) // 2. Ansari, Z. (2007). A Review of Literature on Access to Primary Health Care. Australian Journal of Primary Health, 13(2), 80.
3	156	Acceptability: Patient Satisfaction	Outcome	All	All	Patient-centered	Not Defined	Consumer satisfaction and perceived access derived from postal surveys and focus groups	1. Kringos, D. S., Boerma, W. G., Hutchinson, A., van der Zee, J., & Groenewegen, P. P. (2010). The breadth of primary care: a systematic literature review of its core dimensions. BMC Health Services Research, 10(1) // 2. Ansari, Z. (2007). A Review of Literature on Access to Primary Health Care. Australian Journal of Primary Health, 13(2), 80.

3	157	Preventive care: HIV screen for prenatal patients	Process	Preventive	Screening and prevention	Effective	W - Pregnancy, Childbearing, Family Planning	Preventive care: HIV screen for prenatal patients	1. Kringos, D. S., Boerma, W. G., Hutchinson, A., van der Zee, J., & Groenewegen, P. P. (2010). The breadth of primary care: a systematic literature review of its core dimensions. BMC Health Services Research, 10(1) // 2. Marshall M, Klazinga N, Leatherman S, Hardy C, Bergmann E, Pisco L, et al: OECD Health Care Quality Indicator Project. The expert panel on primary care prevention and health promotion. Int J Qual Health Care 2006, 18(Suppl 1):21-25.
3	158	Preventive care: Bacteriuria screen for prenatal patients	Process	Preventive	Screening and prevention	Effective	W - Pregnancy, Childbearing, Family Planning	Preventive care: Bacteriuria screen for prenatal patients	1. Kringos, D. S., Boerma, W. G., Hutchinson, A., van der Zee, J., & Groenewegen, P. P. (2010). The breadth of primary care: a systematic literature review of its core dimensions. BMC Health Services Research, 10(1) // 2. Marshall M, Klazinga N, Leatherman S, Hardy C, Bergmann E, Pisco L, et al: OECD Health Care Quality Indicator Project. The expert panel on primary care prevention and health promotion. Int J Qual Health Care 2006, 18(Suppl 1):21-25.
3	159	Preventive care: Immunizable conditions	Process	Preventive	Screening and prevention	Effective	W - Pregnancy, Childbearing, Family Planning	Preventive care: Immunizable conditions	1. Kringos, D. S., Boerma, W. G., Hutchinson, A., van der Zee, J., & Groenewegen, P. P. (2010). The breadth of primary care: a systematic literature review of its core dimensions. BMC Health Services Research, 10(1) // 2. Marshall M, Klazinga N, Leatherman S, Hardy C, Bergmann E, Pisco L, et al: OECD Health Care Quality Indicator Project. The expert panel on primary care prevention and health promotion. Int J Qual Health Care 2006, 18(Suppl 1):21-25.
3	160	Preventive care: Low birth weight rate	Outcome	Preventive	Screening and prevention	Effective	W - Pregnancy, Childbearing, Family Planning	Preventive care: Low birth weight rate	1. Kringos, D. S., Boerma, W. G., Hutchinson, A., van der Zee, J., & Groenewegen, P. P. (2010). The breadth of primary care: a systematic literature review of its core dimensions. BMC Health Services Research, 10(1) // 2. Marshall M, Klazinga N, Leatherman S, Hardy C, Bergmann E, Pisco L, et al: OECD Health Care Quality Indicator Project. The expert panel on primary care prevention and health promotion. Int J Qual Health Care 2006, 18(Suppl 1):21-25.
3	161	Preventive care: Adolescent immunization	Process	Preventive	Screening and prevention	Effective	A - General and unspecified	Preventive care: Adolescent immunization	1. Kringos, D. S., Boerma, W. G., Hutchinson, A., van der Zee, J., & Groenewegen, P. P. (2010). The breadth of primary care: a systematic literature review of its core dimensions. BMC Health Services Research, 10(1) // 2. Marshall M, Klazinga N, Leatherman S, Hardy C, Bergmann E, Pisco L, et al: OECD Health Care Quality Indicator Project. The expert panel on primary care prevention and health promotion. Int J Qual Health Care 2006, 18(Suppl 1):21-25.
3	162	Preventive care: Anaemia screening for pregnant women	Process	Preventive	Screening and prevention	Effective	W - Pregnancy, Childbearing, Family Planning	Preventive care: Anaemia screening for pregnant women	1. Kringos, D. S., Boerma, W. G., Hutchinson, A., van der Zee, J., & Groenewegen, P. P. (2010). The breadth of primary care: a systematic literature review of its core dimensions. BMC Health Services Research, 10(1) // 2. Marshall M, Klazinga N, Leatherman S, Hardy C, Bergmann E, Pisco L, et al: OECD Health Care Quality Indicator Project. The expert panel on primary care prevention and health promotion. Int J Qual Health Care 2006, 18(Suppl 1):21-25.
3	163	Preventive care: Cervical gonorrhoea screening for pregnant women	Process	Preventive	Screening and prevention	Effective	W - Pregnancy, Childbearing, Family Planning	Preventive care: Cervical gonorrhoea screening for pregnant women	1. Kringos, D. S., Boerma, W. G., Hutchinson, A., van der Zee, J., & Groenewegen, P. P. (2010). The breadth of primary care: a systematic literature review of its core dimensions. BMC Health Services Research, 10(1) // 2. Marshall M, Klazinga N, Leatherman S, Hardy C, Bergmann E, Pisco L, et al: OECD Health Care Quality Indicator Project. The expert panel on primary care prevention and health promotion. Int J Qual Health Care 2006, 18(Suppl 1):21-25.
3	164	Preventive care: Hepatitis B screen for pregnant women	Process	Preventive	Screening and prevention	Effective	W - Pregnancy, Childbearing, Family Planning	Preventive care: Hepatitis B screen for pregnant women	1. Kringos, D. S., Boerma, W. G., Hutchinson, A., van der Zee, J., & Groenewegen, P. P. (2010). The breadth of primary care: a systematic literature review of its core dimensions. BMC Health Services Research, 10(1) // 2. Marshall M, Klazinga N, Leatherman S, Hardy C, Bergmann E, Pisco L, et al: OECD Health Care Quality Indicator Project. The expert panel on primary care prevention and health promotion. Int J Qual Health Care 2006, 18(Suppl 1):21-25.
3	165	Preventive care: Hepatitis B documentation in record at time of delivery	Process	Preventive	Screening and prevention	Effective	W - Pregnancy, Childbearing, Family Planning	Preventive care: Hepatitis B documentation in record at time of delivery	1. Kringos, D. S., Boerma, W. G., Hutchinson, A., van der Zee, J., & Groenewegen, P. P. (2010). The breadth of primary care: a systematic literature review of its core dimensions. BMC Health Services Research, 10(1) // 2. Marshall M, Klazinga N, Leatherman S, Hardy C, Bergmann E, Pisco L, et al: OECD Health Care Quality Indicator Project. The expert panel on primary care prevention and health promotion. Int J Qual Health Care 2006, 18(Suppl 1):21-25.
3	166	Preventive care: Hepatitis B immunization for high-risk groups	Process	Preventive	Screening and prevention	Effective	A - General and unspecified	Preventive care: Hepatitis B immunization for high-risk groups	1. Kringos, D. S., Boerma, W. G., Hutchinson, A., van der Zee, J., & Groenewegen, P. P. (2010). The breadth of primary care: a systematic literature review of its core dimensions. BMC Health Services Research, 10(1) // 2. Marshall M, Klazinga N, Leatherman S, Hardy C, Bergmann E, Pisco L, et al: OECD Health Care Quality Indicator Project. The expert panel on primary care prevention and health promotion. Int J Qual Health Care 2006, 18(Suppl 1):21-25.
3	167	Preventive care: Influenza vaccination for high-risk groups	Process	Preventive	Screening and prevention	Effective	A - General and unspecified	Preventive care: Influenza vaccination for high-risk groups	1. Kringos, D. S., Boerma, W. G., Hutchinson, A., van der Zee, J., & Groenewegen, P. P. (2010). The breadth of primary care: a systematic literature review of its core dimensions. BMC Health Services Research, 10(1) // 2. Marshall M, Klazinga N, Leatherman S, Hardy C, Bergmann E, Pisco L, et al: OECD Health Care Quality Indicator Project. The expert panel on primary care prevention and health promotion. Int J Qual Health Care 2006, 18(Suppl 1):21-25.
3	168	Preventive care: Pneumococcal vaccination for high-risk groups	Process	Preventive	Screening and prevention	Effective	A - General and unspecified	Preventive care: Pneumococcal vaccination for high-risk groups	1. Kringos, D. S., Boerma, W. G., Hutchinson, A., van der Zee, J., & Groenewegen, P. P. (2010). The breadth of primary care: a systematic literature review of its core dimensions. BMC Health Services Research, 10(1) // 2. Marshall M, Klazinga N, Leatherman S, Hardy C, Bergmann E, Pisco L, et al: OECD Health Care Quality Indicator Project. The expert panel on primary care prevention and health promotion. Int J Qual Health Care 2006, 18(Suppl 1):21-25.
3	169	Quality of maternal and child health care: maternal mortality rates	Outcome	Preventive	Screening and prevention	All	W - Pregnancy, Childbearing, Family Planning	Maternal mortality rate	1. Kringos, D. S., Boerma, W. G., Hutchinson, A., van der Zee, J., & Groenewegen, P. P. (2010). The breadth of primary care: a systematic literature review of its core dimensions. BMC Health Services Research, 10(1) // 2. Macinko J, Starfield B, Shi L: The contribution of primary care systems to health outcomes within Organization for Economic Cooperation and Development (OECD) countries, 1970-1998. Health Serv Res 2003, 38:831-865 // 3. Starfield B, Shi L, Macinko J: Contribution of primary care to health systems and health. Milbank Q 2005, 83:457-502. // 4. Marshall M, Klazinga N, Leatherman S, Hardy C, Bergmann E, Pisco L, et al: OECD Health Care Quality Indicator Project. The expert panel on primary care prevention and health promotion. Int J Qual Health Care 2006, 18(Suppl 1):21-25.
3	170	Quality of maternal and child health care: occurrence of preventive screening for pregnant women	Process	Preventive	Screening and prevention	Effective	W - Pregnancy, Childbearing, Family Planning	Quality of maternal and child health care: occurrence of preventive screening for pregnant women	1. Kringos, D. S., Boerma, W. G., Hutchinson, A., van der Zee, J., & Groenewegen, P. P. (2010). The breadth of primary care: a systematic literature review of its core dimensions. BMC Health Services Research, 10(1) // 2. Macinko J, Starfield B, Shi L: The contribution of primary care systems to health outcomes within Organization for Economic Cooperation and Development (OECD) countries, 1970-1998. Health Serv Res 2003, 38:831-865 // 3. Starfield B, Shi L, Macinko J: Contribution of primary care to health systems and health. Milbank Q 2005, 83:457-502. // 4. Marshall M, Klazinga N, Leatherman S, Hardy C, Bergmann E, Pisco L, et al: OECD Health Care Quality Indicator Project. The expert panel on primary care prevention and health promotion. Int J Qual Health Care 2006, 18(Suppl 1):21-25.
3	171	Quality of maternal and child health care: Infant vaccination	Process	Preventive	Screening and prevention	Effective	W - Pregnancy, Childbearing, Family Planning	Quality of maternal and child health care: Infant vaccination	1. Kringos, D. S., Boerma, W. G., Hutchinson, A., van der Zee, J., & Groenewegen, P. P. (2010). The breadth of primary care: a systematic literature review of its core dimensions. BMC Health Services Research, 10(1) // 2. Macinko J, Starfield B, Shi L: The contribution of primary care systems to health outcomes within Organization for Economic Cooperation and Development (OECD) countries, 1970-1998. Health Serv Res 2003, 38:831-865 // 3. Starfield B, Shi L, Macinko J: Contribution of primary care to health systems and health. Milbank Q 2005, 83:457-502. // 4. Marshall M, Klazinga N, Leatherman S, Hardy C, Bergmann E, Pisco L, et al: OECD Health Care Quality Indicator Project. The expert panel on primary care prevention and health promotion. Int J Qual Health Care 2006, 18(Suppl 1):21-25.
14	172	Additional mortality avoided	Outcome	Preventive	All	Safe	A - General and unspecified	Additional mortality avoided	1. Sans-Corrales, M. (2006). Family medicine attributes related to satisfaction, health and costs. Family Practice, 23(3), 308–316. // 2. Wasson JH, Sauvigne AE, Mogielnicki RP et al. Continuity of outpatient medical care in elderly men. A randomised trial. JAMA 1984; 252: 2413–2417. // 3. Hjortdahl P, Laerum P. Continuity of care in general practice: effect on patient satisfaction. Br Med J 1992; 304: 1287–1290. // 4. McColl A, Roderick P, Gabbay J, Smith H, Moore M. Performance indicators for primary care groups: an evidence based approach. Br Med J 1998; 317: 1354–1360. // 5. Wilson A, Childs S. The relationship between consultation length, process and outcomes in general practice: a systematic review. Br J Gen Pract 2002; 52: 1012–1020. // 6. Stewart M, Brown JB, Donner A, McWhinney I, Oates J, Weston W. The impact of patient-centered care on outcomes. J Fam Pract 2000; 49: 796–804. // 7. Hartley LA. Examination of primary care characteristics in a community-based clinic. J Nurs Scholarsh 2002; 34: 377–382. // 8. Safran DJ. Defining the future of primary care: what can we learn from patients? Ann Intern Med 2003; 138: 248–255
14	173	Communication centred on the patient (recorded interview and perception of the patient)	Process	All	All	Patient-centered	Not Defined	Communication centred on the patient (recorded interview and perception of the patient)	1. Sans-Corrales, M. (2006). Family medicine attributes related to satisfaction, health and costs. Family Practice, 23(3), 308–316. // 2. Wasson JH, Sauvigne AE, Mogielnicki RP et al. Continuity of outpatient medical care in elderly men. A randomised trial. JAMA 1984; 252: 2413–2417. // 3. Hjortdahl P, Laerum P. Continuity of care in general practice: effect on patient satisfaction. Br Med J 1992; 304: 1287–1290. // 4. McColl A, Roderick P, Gabbay J, Smith H, Moore M. Performance indicators for primary care groups: an evidence based approach. Br Med J 1998; 317: 1354–1360. // 5. Wilson A, Childs S. The relationship between consultation length, process and outcomes in general practice: a systematic review. Br J Gen Pract 2002; 52: 1012–1020. // 6. Stewart M, Brown JB, Donner A, McWhinney I, Oates J, Weston W. The impact of patient-centered care on outcomes. J Fam Pract 2000; 49: 796–804. // 7. Hartley LA. Examination of primary care characteristics in a community-based clinic. J Nurs Scholarsh 2002; 34: 377–382. // 8. Safran DJ. Defining the future of primary care: what can we learn from patients? Ann Intern Med 2003; 138: 248–255
14	174	GPAS questionnaire (waiting-list time for an appointment with a specific doctor, or any doctor; waiting time spent in the consulting room)	Process	All	All	Patient-centered	A - General and unspecified	GPAS questionnaire (waiting-list time for an appointment with a specific doctor, or any doctor; waiting time spent in the consulting room)	1. Sans-Corrales, M. (2006). Family medicine attributes related to satisfaction, health and costs. Family Practice, 23(3), 308–316. // 2. Wasson JH, Sauvigne AE, Mogielnicki RP et al. Continuity of outpatient medical care in elderly men. A randomised trial. JAMA 1984; 252: 2413–2417. // 3. Hjortdahl P, Laerum P. Continuity of care in general practice: effect on patient satisfaction. Br Med J 1992; 304: 1287–1290. // 4. McColl A, Roderick P, Gabbay J, Smith H, Moore M. Performance indicators for primary care groups: an evidence based approach. Br Med J 1998; 317: 1354–1360. // 5. Wilson A, Childs S. The relationship between consultation length, process and outcomes in general practice: a systematic review. Br J Gen Pract 2002; 52: 1012–1020. // 6. Stewart M, Brown JB, Donner A, McWhinney I, Oates J, Weston W. The impact of patient-centered care on outcomes. J Fam Pract 2000; 49: 796–804. // 7. Hartley LA. Examination of primary care characteristics in a community-based clinic. J Nurs Scholarsh 2002; 34: 377–382. // 8. Safran DJ. Defining the future of primary care: what can we learn from patients? Ann Intern Med 2003; 138: 248–255
14	175	Indicator of continuity in the process (index of modified continuity) and of continuity in the outcomes (acute problems, chronic, prevention and psychosocial)	Process	All	All	Effective	A - General and unspecified	Indicator of continuity in the process (index of modified continuity) and of continuity in the outcomes (acute problems, chronic, prevention and psychosocial)	1. Sans-Corrales, M. (2006). Family medicine attributes related to satisfaction, health and costs. Family Practice, 23(3), 308–316. // 2. Wasson JH, Sauvigne AE, Mogielnicki RP et al. Continuity of outpatient medical care in elderly men. A randomised trial. JAMA 1984; 252: 2413–2417. // 3. Hjortdahl P, Laerum P. Continuity of care in general practice: effect on patient satisfaction. Br Med J 1992; 304: 1287–1290. // 4. McColl A, Roderick P, Gabbay J, Smith H, Moore M. Performance indicators for primary care groups: an evidence based approach. Br Med J 1998; 317: 1354–1360. // 5. Wilson A, Childs S. The relationship between consultation length, process and outcomes in general practice: a systematic review. Br J Gen Pract 2002; 52: 1012–1020. // 6. Stewart M, Brown JB, Donner A, McWhinney I, Oates J, Weston W. The impact of patient-centered care on outcomes. J Fam Pract 2000; 49: 796–804. // 7. Hartley LA. Examination of primary care characteristics in a community-based clinic. J Nurs Scholarsh 2002; 34: 377–382. // 8. Safran DJ. Defining the future of primary care: what can we learn from patients? Ann Intern Med 2003; 138: 248–255
14	176	MAAS-global Questionnaire (Quality of communication (MAAS-global Questionnaire), satisfaction (EVA-PAT Questionnaire) and consultation time)	Outcome	All	All	Patient-centered	Not Defined	MAAS-global Questionnaire (Quality of communication (MAAS-global Questionnaire), satisfaction (EVA-PAT Questionnaire) and consultation time)	1. Sans-Corrales, M. (2006). Family medicine attributes related to satisfaction, health and costs. Family Practice, 23(3), 308–316. // 2. Wasson JH, Sauvigne AE, Mogielnicki RP et al. Continuity of outpatient medical care in elderly men. A randomised trial. JAMA 1984; 252: 2413–2417. // 3. Hjortdahl P, Laerum P. Continuity of care in general practice: effect on patient satisfaction. Br Med J 1992; 304: 1287–1290. // 4. McColl A, Roderick P, Gabbay J, Smith H, Moore M. Performance indicators for primary care groups: an evidence based approach. Br Med J 1998; 317: 1354–1360. // 5. Wilson A, Childs S. The relationship between consultation length, process and outcomes in general practice: a systematic review. Br J Gen Pract 2002; 52: 1012–1020. // 6. Stewart M, Brown JB, Donner A, McWhinney I, Oates J, Weston W. The impact of patient-centered care on outcomes. J Fam Pract 2000; 49: 796–804. // 7. Hartley LA. Examination of primary care characteristics in a community-based clinic. J Nurs Scholarsh 2002; 34: 377–382. // 8. Safran DJ. Defining the future of primary care: what can we learn from patients? Ann Intern Med 2003; 138: 248–255
14	177	Mortality avoided	Outcome	Preventive	All	Safe	A - General and unspecified	Mortality avoided	1. Sans-Corrales, M. (2006). Family medicine attributes related to satisfaction, health and costs. Family Practice, 23(3), 308–316. // 2. Wasson JH, Sauvigne AE, Mogielnicki RP et al. Continuity of outpatient medical care in elderly men. A randomised trial. JAMA 1984; 252: 2413–2417. // 3. Hjortdahl P, Laerum P. Continuity of care in general practice: effect on patient satisfaction. Br Med J 1992; 304: 1287–1290. // 4. McColl A, Roderick P, Gabbay J, Smith H, Moore M. Performance indicators for primary care groups: an evidence based approach. Br Med J 1998; 317: 1354–1360. // 5. Wilson A, Childs S. The relationship between consultation length, process and outcomes in general practice: a systematic review. Br J Gen Pract 2002; 52: 1012–1020. // 6. Stewart M, Brown JB, Donner A, McWhinney I, Oates J, Weston W. The impact of patient-centered care on outcomes. J Fam Pract 2000; 49: 796–804. // 7. Hartley LA. Examination of primary care characteristics in a community-based clinic. J Nurs Scholarsh 2002; 34: 377–382. // 8. Safran DJ. Defining the future of primary care: what can we learn from patients? Ann Intern Med 2003; 138: 248–255
14	178	Mortality theoretically avoided	Outcome	Preventive	All	Safe	A - General and unspecified	Mortality theoretically avoided	1. Sans-Corrales, M. (2006). Family medicine attributes related to satisfaction, health and costs. Family Practice, 23(3), 308–316. // 2. Wasson JH, Sauvigne AE, Mogielnicki RP et al. Continuity of outpatient medical care in elderly men. A randomised trial. JAMA 1984; 252: 2413–2417. // 3. Hjortdahl P, Laerum P. Continuity of care in general practice: effect on patient satisfaction. Br Med J 1992; 304: 1287–1290. // 4. McColl A, Roderick P, Gabbay J, Smith H, Moore M. Performance indicators for primary care groups: an evidence based approach. Br Med J 1998; 317: 1354–1360. // 5. Wilson A, Childs S. The relationship between consultation length, process and outcomes in general practice: a systematic review. Br J Gen Pract 2002; 52: 1012–1020. // 6. Stewart M, Brown JB, Donner A, McWhinney I, Oates J, Weston W. The impact of patient-centered care on outcomes. J Fam Pract 2000; 49: 796–804. // 7. Hartley LA. Examination of primary care characteristics in a community-based clinic. J Nurs Scholarsh 2002; 34: 377–382. // 8. Safran DJ. Defining the future of primary care: what can we learn from patients? Ann Intern Med 2003; 138: 248–255
14	179	Number and proportion of persons eligible for each preventive intervention among 100 000 citizens	Process	Preventive	Screening and prevention	Effective	A - General and unspecified	Number and proportion of persons eligible for each preventive intervention among 100 000 citizens	1. Sans-Corrales, M. (2006). Family medicine attributes related to satisfaction, health and costs. Family Practice, 23(3), 308–316. // 2. Wasson JH, Sauvigne AE, Mogielnicki RP et al. Continuity of outpatient medical care in elderly men. A randomised trial. JAMA 1984; 252: 2413–2417. // 3. Hjortdahl P, Laerum P. Continuity of care in general practice: effect on patient satisfaction. Br Med J 1992; 304: 1287–1290. // 4. McColl A, Roderick P, Gabbay J, Smith H, Moore M. Performance indicators for primary care groups: an evidence based approach. Br Med J 1998; 317: 1354–1360. // 5. Wilson A, Childs S. The relationship between consultation length, process and outcomes in general practice: a systematic review. Br J Gen Pract 2002; 52: 1012–1020. // 6. Stewart M, Brown JB, Donner A, McWhinney I, Oates J, Weston W. The impact of patient-centered care on outcomes. J Fam Pract 2000; 49: 796–804. // 7. Hartley LA. Examination of primary care characteristics in a community-based clinic. J Nurs Scholarsh 2002; 34: 377–382. // 8. Safran DJ. Defining the future of primary care: what can we learn from patients? Ann Intern Med 2003; 138: 248–255
14	180	Number of deaths prevented if 100% of the population received the intervention	Outcome	Preventive	Treatment	Effective	A - General and unspecified	Number of deaths prevented if 100% of the population received the intervention	1. Sans-Corrales, M. (2006). Family medicine attributes related to satisfaction, health and costs. Family Practice, 23(3), 308–316. // 2. Wasson JH, Sauvigne AE, Mogielnicki RP et al. Continuity of outpatient medical care in elderly men. A randomised trial. JAMA 1984; 252: 2413–2417. // 3. Hjortdahl P, Laerum P. Continuity of care in general practice: effect on patient satisfaction. Br Med J 1992; 304: 1287–1290. // 4. McColl A, Roderick P, Gabbay J, Smith H, Moore M. Performance indicators for primary care groups: an evidence based approach. Br Med J 1998; 317: 1354–1360. // 5. Wilson A, Childs S. The relationship between consultation length, process and outcomes in general practice: a systematic review. Br J Gen Pract 2002; 52: 1012–1020. // 6. Stewart M, Brown JB, Donner A, McWhinney I, Oates J, Weston W. The impact of patient-centered care on outcomes. J Fam Pract 2000; 49: 796–804. // 7. Hartley LA. Examination of primary care characteristics in a community-based clinic. J Nurs Scholarsh 2002; 34: 377–382. // 8. Safran DJ. Defining the future of primary care: what can we learn from patients? Ann Intern Med 2003; 138: 248–255
14	181	Quality of life (CVRS and SF-36)	Outcome	Preventive	All	Patient-centered	A - General and unspecified	Quality of life (CVRS and SF-36)	1. Sans-Corrales, M. (2006). Family medicine attributes related to satisfaction, health and costs. Family Practice, 23(3), 308–316. // 2. Wasson JH, Sauvigne AE, Mogielnicki RP et al. Continuity of outpatient medical care in elderly men. A randomised trial. JAMA 1984; 252: 2413–2417. // 3. Hjortdahl P, Laerum P. Continuity of care in general practice: effect on patient satisfaction. Br Med J 1992; 304: 1287–1290. // 4. McColl A, Roderick P, Gabbay J, Smith H, Moore M. Performance indicators for primary care groups: an evidence based approach. Br Med J 1998; 317: 1354–1360. // 5. Wilson A, Childs S. The relationship between consultation length, process and outcomes in general practice: a systematic review. Br J Gen Pract 2002; 52: 1012–1020. // 6. Stewart M, Brown JB, Donner A, McWhinney I, Oates J, Weston W. The impact of patient-centered care on outcomes. J Fam Pract 2000; 49: 796–804. // 7. Hartley LA. Examination of primary care characteristics in a community-based clinic. J Nurs Scholarsh 2002; 34: 377–382. // 8. Safran DJ. Defining the future of primary care: what can we learn from patients? Ann Intern Med 2003; 138: 248–255
14	182	Questions on satisfaction, communication, personal relationship, awareness of problems and interest in the effects of the problem on personal and family quality of life	Outcome	All	All	Patient-centered	A - General and unspecified	Questions on satisfaction, communication, personal relationship, awareness of problems and interest in the effects of the problem on personal and family quality of life	1. Sans-Corrales, M. (2006). Family medicine attributes related to satisfaction, health and costs. Family Practice, 23(3), 308–316. // 2. Wasson JH, Sauvigne AE, Mogielnicki RP et al. Continuity of outpatient medical care in elderly men. A randomised trial. JAMA 1984; 252: 2413–2417. // 3. Hjortdahl P, Laerum P. Continuity of care in general practice: effect on patient satisfaction. Br Med J 1992; 304: 1287–1290. // 4. McColl A, Roderick P, Gabbay J, Smith H, Moore M. Performance indicators for primary care groups: an evidence based approach. Br Med J 1998; 317: 1354–1360. // 5. Wilson A, Childs S. The relationship between consultation length, process and outcomes in general practice: a systematic review. Br J Gen Pract 2002; 52: 1012–1020. // 6. Stewart M, Brown JB, Donner A, McWhinney I, Oates J, Weston W. The impact of patient-centered care on outcomes. J Fam Pract 2000; 49: 796–804. // 7. Hartley LA. Examination of primary care characteristics in a community-based clinic. J Nurs Scholarsh 2002; 34: 377–382. // 8. Safran DJ. Defining the future of primary care: what can we learn from patients? Ann Intern Med 2003; 138: 248–255
14	183	Reduction in absolute risk	Outcome	All	All	Effective	A - General and unspecified	Reduction in absolute risk	1. Sans-Corrales, M. (2006). Family medicine attributes related to satisfaction, health and costs. Family Practice, 23(3), 308–316. // 2. Wasson JH, Sauvigne AE, Mogielnicki RP et al. Continuity of outpatient medical care in elderly men. A randomised trial. JAMA 1984; 252: 2413–2417. // 3. Hjortdahl P, Laerum P. Continuity of care in general practice: effect on patient satisfaction. Br Med J 1992; 304: 1287–1290. // 4. McColl A, Roderick P, Gabbay J, Smith H, Moore M. Performance indicators for primary care groups: an evidence based approach. Br Med J 1998; 317: 1354–1360. // 5. Wilson A, Childs S. The relationship between consultation length, process and outcomes in general practice: a systematic review. Br J Gen Pract 2002; 52: 1012–1020. // 6. Stewart M, Brown JB, Donner A, McWhinney I, Oates J, Weston W. The impact of patient-centered care on outcomes. J Fam Pract 2000; 49: 796–804. // 7. Hartley LA. Examination of primary care characteristics in a community-based clinic. J Nurs Scholarsh 2002; 34: 377–382. // 8. Safran DJ. Defining the future of primary care: what can we learn from patients? Ann Intern Med 2003; 138: 248–255
14	184	Reduction in relative risk	Outcome	All	All	Effective	A - General and unspecified	Reduction in relative risk	1. Sans-Corrales, M. (2006). Family medicine attributes related to satisfaction, health and costs. Family Practice, 23(3), 308–316. // 2. Wasson JH, Sauvigne AE, Mogielnicki RP et al. Continuity of outpatient medical care in elderly men. A randomised trial. JAMA 1984; 252: 2413–2417. // 3. Hjortdahl P, Laerum P. Continuity of care in general practice: effect on patient satisfaction. Br Med J 1992; 304: 1287–1290. // 4. McColl A, Roderick P, Gabbay J, Smith H, Moore M. Performance indicators for primary care groups: an evidence based approach. Br Med J 1998; 317: 1354–1360. // 5. Wilson A, Childs S. The relationship between consultation length, process and outcomes in general practice: a systematic review. Br J Gen Pract 2002; 52: 1012–1020. // 6. Stewart M, Brown JB, Donner A, McWhinney I, Oates J, Weston W. The impact of patient-centered care on outcomes. J Fam Pract 2000; 49: 796–804. // 7. Hartley LA. Examination of primary care characteristics in a community-based clinic. J Nurs Scholarsh 2002; 34: 377–382. // 8. Safran DJ. Defining the future of primary care: what can we learn from patients? Ann Intern Med 2003; 138: 248–255
14	185	Resolution capacity	Process	All	All	Efficient	A - General and unspecified	Resolution capacity	1. Sans-Corrales, M. (2006). Family medicine attributes related to satisfaction, health and costs. Family Practice, 23(3), 308–316. // 2. Wasson JH, Sauvigne AE, Mogielnicki RP et al. Continuity of outpatient medical care in elderly men. A randomised trial. JAMA 1984; 252: 2413–2417. // 3. Hjortdahl P, Laerum P. Continuity of care in general practice: effect on patient satisfaction. Br Med J 1992; 304: 1287–1290. // 4. McColl A, Roderick P, Gabbay J, Smith H, Moore M. Performance indicators for primary care groups: an evidence based approach. Br Med J 1998; 317: 1354–1360. // 5. Wilson A, Childs S. The relationship between consultation length, process and outcomes in general practice: a systematic review. Br J Gen Pract 2002; 52: 1012–1020. // 6. Stewart M, Brown JB, Donner A, McWhinney I, Oates J, Weston W. The impact of patient-centered care on outcomes. J Fam Pract 2000; 49: 796–804. // 7. Hartley LA. Examination of primary care characteristics in a community-based clinic. J Nurs Scholarsh 2002; 34: 377–382. // 8. Safran DJ. Defining the future of primary care: what can we learn from patients? Ann Intern Med 2003; 138: 248–255

15	186	Allopurinol without baseline urea, electrolytes, creatinine and estimated glomerular filtration rate	Process	Chronic	Treatment	Safe	U - Urological	Allopurinol without baseline urea, electrolytes, creatinine and estimated glomerular filtration rate	1. Spencer, R., Bell, B., Avery, A. J., Gookey, G., & Campbell, S. M. (2014). Identification of an updated set of prescribing-safety indicators for GPs. British Journal of General Practice, 64(621), e181–e190. // 2. Avery AJ, Rodgers S, Cantrill JA, et al. A pharmacist-led information technology intervention for medication errors (PINCER): a multicentre, cluster randomised, controlled trial and cost-effectiveness analysis. Lancet 2012; 379:1310–1319 // 1. Guthrie B, McCowan C, Davey P, et al. High risk prescribing in primary care patients particularly vulnerable to adverse drug events: cross sectional population database analysis in Scottish general practice. BMJ 2011; 342: d3514. // 3. Rognstad S, Brekke M, Fetveit A, et al. The Norwegian General Practice (NORGEP) criteria for assessing potentially inappropriate prescriptions to elderly patients. A modified Delphi study. Scand J Prim Health Care 2009; 27(3): 153–159. // 4. Gallagher P, Ryan C, Byrne S, et al. STOPP (Screening Tool of Older Person's Prescriptions) and START (Screening Tool to Alert doctors to Right Treatment). Consensus validation. Int J Clin Pharmacol Ther 2008; 46(2): 72–83. // 5. Basger BJ, Chen TF, Moles RJ. Inappropriate medication use and prescribing indicators in elderly Australians: development of a prescribing indicators tool. Drugs Aging 2008; 25(9): 777–793. // 6. Zhan C, Correa-de-Araujo R, Bierman AS, et al. Suboptimal prescribing in elderly outpatients: potentially harmful drug-drug and drug-disease combinations. J Am Geriatr Soc 2005; 53(2): 262–267. // 7. McLeod PJ, Huang AR, Tamblyn RM, Gayton DC. Defining inappropriate practices in prescribing for elderly people: a national consensus panel. CMAJ 1997; 156(3): 385–391
15	187	Aspirin or clopidogrel prescribed to people with previous peptic ulcer or gastrointestinal bleed without gastroprotection	Process	Chronic	Treatment	Safe	D - Digestive	Aspirin or clopidogrel prescribed to people with previous peptic ulcer or gastrointestinal bleed without gastroprotection	1. Spencer, R., Bell, B., Avery, A. J., Gookey, G., & Campbell, S. M. (2014). Identification of an updated set of prescribing-safety indicators for GPs. British Journal of General Practice, 64(621), e181–e190. // 2. Avery AJ, Rodgers S, Cantrill JA, et al. A pharmacist-led information technology intervention for medication errors (PINCER): a multicentre, cluster randomised, controlled trial and cost-effectiveness analysis. Lancet 2012; 379:1310–1319 // 1. Guthrie B, McCowan C, Davey P, et al. High risk prescribing in primary care patients particularly vulnerable to adverse drug events: cross sectional population database analysis in Scottish general practice. BMJ 2011; 342: d3514. // 3. Rognstad S, Brekke M, Fetveit A, et al. The Norwegian General Practice (NORGEP) criteria for assessing potentially inappropriate prescriptions to elderly patients. A modified Delphi study. Scand J Prim Health Care 2009; 27(3): 153–159. // 4. Gallagher P, Ryan C, Byrne S, et al. STOPP (Screening Tool of Older Person's Prescriptions) and START (Screening Tool to Alert doctors to Right Treatment). Consensus validation. Int J Clin Pharmacol Ther 2008; 46(2): 72–83. // 5. Basger BJ, Chen TF, Moles RJ. Inappropriate medication use and prescribing indicators in elderly Australians: development of a prescribing indicators tool. Drugs Aging 2008; 25(9): 777–793. // 6. Zhan C, Correa-de-Araujo R, Bierman AS, et al. Suboptimal prescribing in elderly outpatients: potentially harmful drug-drug and drug-disease combinations. J Am Geriatr Soc 2005; 53(2): 262–267. // 7. McLeod PJ, Huang AR, Tamblyn RM, Gayton DC. Defining inappropriate practices in prescribing for elderly people: a national consensus panel. CMAJ 1997; 156(3): 385–391
15	188	Concurrent use of two Non-steroid anti-inflammatory drugs for more than 2 weeks (not including low-dose aspirin)	Process	Chronic	Treatment	Safe	A - General and unspecified	Concurrent use of two Non-steroid anti-inflammatory drugs for more than 2 weeks (not including low-dose aspirin)	1. Spencer, R., Bell, B., Avery, A. J., Gookey, G., & Campbell, S. M. (2014). Identification of an updated set of prescribing-safety indicators for GPs. British Journal of General Practice, 64(621), e181–e190. // 2. Avery AJ, Rodgers S, Cantrill JA, et al. A pharmacist-led information technology intervention for medication errors (PINCER): a multicentre, cluster randomised, controlled trial and cost-effectiveness analysis. Lancet 2012; 379:1310–1319 // 1. Guthrie B, McCowan C, Davey P, et al. High risk prescribing in primary care patients particularly vulnerable to adverse drug events: cross sectional population database analysis in Scottish general practice. BMJ 2011; 342: d3514. // 3. Rognstad S, Brekke M, Fetveit A, et al. The Norwegian General Practice (NORGEP) criteria for assessing potentially inappropriate prescriptions to elderly patients. A modified Delphi study. Scand J Prim Health Care 2009; 27(3): 153–159. // 4. Gallagher P, Ryan C, Byrne S, et al. STOPP (Screening Tool of Older Person's Prescriptions) and START (Screening Tool to Alert doctors to Right Treatment). Consensus validation. Int J Clin Pharmacol Ther 2008; 46(2): 72–83. // 5. Basger BJ, Chen TF, Moles RJ. Inappropriate medication use and prescribing indicators in elderly Australians: development of a prescribing indicators tool. Drugs Aging 2008; 25(9): 777–793. // 6. Zhan C, Correa-de-Araujo R, Bierman AS, et al. Suboptimal prescribing in elderly outpatients: potentially harmful drug-drug and drug-disease combinations. J Am Geriatr Soc 2005; 53(2): 262–267. // 7. McLeod PJ, Huang AR, Tamblyn RM, Gayton DC. Defining inappropriate practices in prescribing for elderly people: a national consensus panel. CMAJ 1997; 156(3): 385–391
15	189	Concurrent use of warfarin and any antibiotic without monitoring the INR within 5 days	Process	Chronic	Treatment	Safe	K - Cardiovascular	Concurrent use of warfarin and any antibiotic without monitoring the INR within 5 days	1. Spencer, R., Bell, B., Avery, A. J., Gookey, G., & Campbell, S. M. (2014). Identification of an updated set of prescribing-safety indicators for GPs. British Journal of General Practice, 64(621), e181–e190. // 2. Avery AJ, Rodgers S, Cantrill JA, et al. A pharmacist-led information technology intervention for medication errors (PINCER): a multicentre, cluster randomised, controlled trial and cost-effectiveness analysis. Lancet 2012; 379:1310–1319 // 1. Guthrie B, McCowan C, Davey P, et al. High risk prescribing in primary care patients particularly vulnerable to adverse drug events: cross sectional population database analysis in Scottish general practice. BMJ 2011; 342: d3514. // 3. Rognstad S, Brekke M, Fetveit A, et al. The Norwegian General Practice (NORGEP) criteria for assessing potentially inappropriate prescriptions to elderly patients. A modified Delphi study. Scand J Prim Health Care 2009; 27(3): 153–159. // 4. Gallagher P, Ryan C, Byrne S, et al. STOPP (Screening Tool of Older Person's Prescriptions) and START (Screening Tool to Alert doctors to Right Treatment). Consensus validation. Int J Clin Pharmacol Ther 2008; 46(2): 72–83. // 5. Basger BJ, Chen TF, Moles RJ. Inappropriate medication use and prescribing indicators in elderly Australians: development of a prescribing indicators tool. Drugs Aging 2008; 25(9): 777–793. // 6. Zhan C, Correa-de-Araujo R, Bierman AS, et al. Suboptimal prescribing in elderly outpatients: potentially harmful drug-drug and drug-disease combinations. J Am Geriatr Soc 2005; 53(2): 262–267. // 7. McLeod PJ, Huang AR, Tamblyn RM, Gayton DC. Defining inappropriate practices in prescribing for elderly people: a national consensus panel. CMAJ 1997; 156(3): 385–391
15	190	Co-prescription of itraconazole with simvastatin, or with atorvastatin at a dose ≥80mg	Process	Chronic	Treatment	Safe	K - Cardiovascular	Co-prescription of itraconazole with simvastatin, or with atorvastatin at a dose ≥80mg	1. Spencer, R., Bell, B., Avery, A. J., Gookey, G., & Campbell, S. M. (2014). Identification of an updated set of prescribing-safety indicators for GPs. British Journal of General Practice, 64(621), e181–e190. // 2. Avery AJ, Rodgers S, Cantrill JA, et al. A pharmacist-led information technology intervention for medication errors (PINCER): a multicentre, cluster randomised, controlled trial and cost-effectiveness analysis. Lancet 2012; 379:1310–1319 // 1. Guthrie B, McCowan C, Davey P, et al. High risk prescribing in primary care patients particularly vulnerable to adverse drug events: cross sectional population database analysis in Scottish general practice. BMJ 2011; 342: d3514. // 3. Rognstad S, Brekke M, Fetveit A, et al. The Norwegian General Practice (NORGEP) criteria for assessing potentially inappropriate prescriptions to elderly patients. A modified Delphi study. Scand J Prim Health Care 2009; 27(3): 153–159. // 4. Gallagher P, Ryan C, Byrne S, et al. STOPP (Screening Tool of Older Person's Prescriptions) and START (Screening Tool to Alert doctors to Right Treatment). Consensus validation. Int J Clin Pharmacol Ther 2008; 46(2): 72–83. // 5. Basger BJ, Chen TF, Moles RJ. Inappropriate medication use and prescribing indicators in elderly Australians: development of a prescribing indicators tool. Drugs Aging 2008; 25(9): 777–793. // 6. Zhan C, Correa-de-Araujo R, Bierman AS, et al. Suboptimal prescribing in elderly outpatients: potentially harmful drug-drug and drug-disease combinations. J Am Geriatr Soc 2005; 53(2): 262–267. // 7. McLeod PJ, Huang AR, Tamblyn RM, Gayton DC. Defining inappropriate practices in prescribing for elderly people: a national consensus panel. CMAJ 1997; 156(3): 385–391
15	191	Co-prescription of lithium with thiazide diuretic	Process	Chronic	Treatment	Safe	K - Cardiovascular	Co-prescription of lithium with thiazide diuretic	1. Spencer, R., Bell, B., Avery, A. J., Gookey, G., & Campbell, S. M. (2014). Identification of an updated set of prescribing-safety indicators for GPs. British Journal of General Practice, 64(621), e181–e190. // 2. Avery AJ, Rodgers S, Cantrill JA, et al. A pharmacist-led information technology intervention for medication errors (PINCER): a multicentre, cluster randomised, controlled trial and cost-effectiveness analysis. Lancet 2012; 379:1310–1319 // 1. Guthrie B, McCowan C, Davey P, et al. High risk prescribing in primary care patients particularly vulnerable to adverse drug events: cross sectional population database analysis in Scottish general practice. BMJ 2011; 342: d3514. // 3. Rognstad S, Brekke M, Fetveit A, et al. The Norwegian General Practice (NORGEP) criteria for assessing potentially inappropriate prescriptions to elderly patients. A modified Delphi study. Scand J Prim Health Care 2009; 27(3): 153–159. // 4. Gallagher P, Ryan C, Byrne S, et al. STOPP (Screening Tool of Older Person's Prescriptions) and START (Screening Tool to Alert doctors to Right Treatment). Consensus validation. Int J Clin Pharmacol Ther 2008; 46(2): 72–83. // 5. Basger BJ, Chen TF, Moles RJ. Inappropriate medication use and prescribing indicators in elderly Australians: development of a prescribing indicators tool. Drugs Aging 2008; 25(9): 777–793. // 6. Zhan C, Correa-de-Araujo R, Bierman AS, et al. Suboptimal prescribing in elderly outpatients: potentially harmful drug-drug and drug-disease combinations. J Am Geriatr Soc 2005; 53(2): 262–267. // 7. McLeod PJ, Huang AR, Tamblyn RM, Gayton DC. Defining inappropriate practices in prescribing for elderly people: a national consensus panel. CMAJ 1997; 156(3): 385–391
15	192	Co-prescription of trimethoprim with methotrexate for >7 days	Process	Chronic	Treatment	Safe	A - General and unspecified	Co-prescription of trimethoprim with methotrexate for >7 days	1. Spencer, R., Bell, B., Avery, A. J., Gookey, G., & Campbell, S. M. (2014). Identification of an updated set of prescribing-safety indicators for GPs. British Journal of General Practice, 64(621), e181–e190. // 2. Avery AJ, Rodgers S, Cantrill JA, et al. A pharmacist-led information technology intervention for medication errors (PINCER): a multicentre, cluster randomised, controlled trial and cost-effectiveness analysis. Lancet 2012; 379:1310–1319 // 1. Guthrie B, McCowan C, Davey P, et al. High risk prescribing in primary care patients particularly vulnerable to adverse drug events: cross sectional population database analysis in Scottish general practice. BMJ 2011; 342: d3514. // 3. Rognstad S, Brekke M, Fetveit A, et al. The Norwegian General Practice (NORGEP) criteria for assessing potentially inappropriate prescriptions to elderly patients. A modified Delphi study. Scand J Prim Health Care 2009; 27(3): 153–159. // 4. Gallagher P, Ryan C, Byrne S, et al. STOPP (Screening Tool of Older Person's Prescriptions) and START (Screening Tool to Alert doctors to Right Treatment). Consensus validation. Int J Clin Pharmacol Ther 2008; 46(2): 72–83. // 5. Basger BJ, Chen TF, Moles RJ. Inappropriate medication use and prescribing indicators in elderly Australians: development of a prescribing indicators tool. Drugs Aging 2008; 25(9): 777–793. // 6. Zhan C, Correa-de-Araujo R, Bierman AS, et al. Suboptimal prescribing in elderly outpatients: potentially harmful drug-drug and drug-disease combinations. J Am Geriatr Soc 2005; 53(2): 262–267. // 7. McLeod PJ, Huang AR, Tamblyn RM, Gayton DC. Defining inappropriate practices in prescribing for elderly people: a national consensus panel. CMAJ 1997; 156(3): 385–391
15	193	Metformin without yearly serum creatinine	Process	Chronic	Treatment	Safe	U - Urological	Metformin without yearly serum creatinine	1. Spencer, R., Bell, B., Avery, A. J., Gookey, G., & Campbell, S. M. (2014). Identification of an updated set of prescribing-safety indicators for GPs. British Journal of General Practice, 64(621), e181–e190. // 2. Avery AJ, Rodgers S, Cantrill JA, et al. A pharmacist-led information technology intervention for medication errors (PINCER): a multicentre, cluster randomised, controlled trial and cost-effectiveness analysis. Lancet 2012; 379:1310–1319 // 1. Guthrie B, McCowan C, Davey P, et al. High risk prescribing in primary care patients particularly vulnerable to adverse drug events: cross sectional population database analysis in Scottish general practice. BMJ 2011; 342: d3514. // 3. Rognstad S, Brekke M, Fetveit A, et al. The Norwegian General Practice (NORGEP) criteria for assessing potentially inappropriate prescriptions to elderly patients. A modified Delphi study. Scand J Prim Health Care 2009; 27(3): 153–159. // 4. Gallagher P, Ryan C, Byrne S, et al. STOPP (Screening Tool of Older Person's Prescriptions) and START (Screening Tool to Alert doctors to Right Treatment). Consensus validation. Int J Clin Pharmacol Ther 2008; 46(2): 72–83. // 5. Basger BJ, Chen TF, Moles RJ. Inappropriate medication use and prescribing indicators in elderly Australians: development of a prescribing indicators tool. Drugs Aging 2008; 25(9): 777–793. // 6. Zhan C, Correa-de-Araujo R, Bierman AS, et al. Suboptimal prescribing in elderly outpatients: potentially harmful drug-drug and drug-disease combinations. J Am Geriatr Soc 2005; 53(2): 262–267. // 7. McLeod PJ, Huang AR, Tamblyn RM, Gayton DC. Defining inappropriate practices in prescribing for elderly people: a national consensus panel. CMAJ 1997; 156(3): 385–391
15	194	Methotrexate 2.5/10mg co-prescription	Process	Chronic	Treatment	Safe	L - Musculoskeletal	Methotrexate 2.5/10mg co-prescription	1. Spencer, R., Bell, B., Avery, A. J., Gookey, G., & Campbell, S. M. (2014). Identification of an updated set of prescribing-safety indicators for GPs. British Journal of General Practice, 64(621), e181–e190. // 2. Avery AJ, Rodgers S, Cantrill JA, et al. A pharmacist-led information technology intervention for medication errors (PINCER): a multicentre, cluster randomised, controlled trial and cost-effectiveness analysis. Lancet 2012; 379:1310–1319 // 1. Guthrie B, McCowan C, Davey P, et al. High risk prescribing in primary care patients particularly vulnerable to adverse drug events: cross sectional population database analysis in Scottish general practice. BMJ 2011; 342: d3514. // 3. Rognstad S, Brekke M, Fetveit A, et al. The Norwegian General Practice (NORGEP) criteria for assessing potentially inappropriate prescriptions to elderly patients. A modified Delphi study. Scand J Prim Health Care 2009; 27(3): 153–159. // 4. Gallagher P, Ryan C, Byrne S, et al. STOPP (Screening Tool of Older Person's Prescriptions) and START (Screening Tool to Alert doctors to Right Treatment). Consensus validation. Int J Clin Pharmacol Ther 2008; 46(2): 72–83. // 5. Basger BJ, Chen TF, Moles RJ. Inappropriate medication use and prescribing indicators in elderly Australians: development of a prescribing indicators tool. Drugs Aging 2008; 25(9): 777–793. // 6. Zhan C, Correa-de-Araujo R, Bierman AS, et al. Suboptimal prescribing in elderly outpatients: potentially harmful drug-drug and drug-disease combinations. J Am Geriatr Soc 2005; 53(2): 262–267. // 7. McLeod PJ, Huang AR, Tamblyn RM, Gayton DC. Defining inappropriate practices in prescribing for elderly people: a national consensus panel. CMAJ 1997; 156(3): 385–391
15	195	Methotrexate prescribed without folic acid	Process	Chronic	Treatment	Safe	L - Musculoskeletal	Methotrexate prescribed without folic acid	1. Spencer, R., Bell, B., Avery, A. J., Gookey, G., & Campbell, S. M. (2014). Identification of an updated set of prescribing-safety indicators for GPs. British Journal of General Practice, 64(621), e181–e190. // 2. Avery AJ, Rodgers S, Cantrill JA, et al. A pharmacist-led information technology intervention for medication errors (PINCER): a multicentre, cluster randomised, controlled trial and cost-effectiveness analysis. Lancet 2012; 379:1310–1319 // 1. Guthrie B, McCowan C, Davey P, et al. High risk prescribing in primary care patients particularly vulnerable to adverse drug events: cross sectional population database analysis in Scottish general practice. BMJ 2011; 342: d3514. // 3. Rognstad S, Brekke M, Fetveit A, et al. The Norwegian General Practice (NORGEP) criteria for assessing potentially inappropriate prescriptions to elderly patients. A modified Delphi study. Scand J Prim Health Care 2009; 27(3): 153–159. // 4. Gallagher P, Ryan C, Byrne S, et al. STOPP (Screening Tool of Older Person's Prescriptions) and START (Screening Tool to Alert doctors to Right Treatment). Consensus validation. Int J Clin Pharmacol Ther 2008; 46(2): 72–83. // 5. Basger BJ, Chen TF, Moles RJ. Inappropriate medication use and prescribing indicators in elderly Australians: development of a prescribing indicators tool. Drugs Aging 2008; 25(9): 777–793. // 6. Zhan C, Correa-de-Araujo R, Bierman AS, et al. Suboptimal prescribing in elderly outpatients: potentially harmful drug-drug and drug-disease combinations. J Am Geriatr Soc 2005; 53(2): 262–267. // 7. McLeod PJ, Huang AR, Tamblyn RM, Gayton DC. Defining inappropriate practices in prescribing for elderly people: a national consensus panel. CMAJ 1997; 156(3): 385–391
15	196	Methotrexate prescriptions should state 'weekly'	Process	Chronic	Treatment	Safe	L - Musculoskeletal	Methotrexate prescriptions should state 'weekly'	1. Spencer, R., Bell, B., Avery, A. J., Gookey, G., & Campbell, S. M. (2014). Identification of an updated set of prescribing-safety indicators for GPs. British Journal of General Practice, 64(621), e181–e190. // 2. Avery AJ, Rodgers S, Cantrill JA, et al. A pharmacist-led information technology intervention for medication errors (PINCER): a multicentre, cluster randomised, controlled trial and cost-effectiveness analysis. Lancet 2012; 379:1310–1319 // 1. Guthrie B, McCowan C, Davey P, et al. High risk prescribing in primary care patients particularly vulnerable to adverse drug events: cross sectional population database analysis in Scottish general practice. BMJ 2011; 342: d3514. // 3. Rognstad S, Brekke M, Fetveit A, et al. The Norwegian General Practice (NORGEP) criteria for assessing potentially inappropriate prescriptions to elderly patients. A modified Delphi study. Scand J Prim Health Care 2009; 27(3): 153–159. // 4. Gallagher P, Ryan C, Byrne S, et al. STOPP (Screening Tool of Older Person's Prescriptions) and START (Screening Tool to Alert doctors to Right Treatment). Consensus validation. Int J Clin Pharmacol Ther 2008; 46(2): 72–83. // 5. Basger BJ, Chen TF, Moles RJ. Inappropriate medication use and prescribing indicators in elderly Australians: development of a prescribing indicators tool. Drugs Aging 2008; 25(9): 777–793. // 6. Zhan C, Correa-de-Araujo R, Bierman AS, et al. Suboptimal prescribing in elderly outpatients: potentially harmful drug-drug and drug-disease combinations. J Am Geriatr Soc 2005; 53(2): 262–267. // 7. McLeod PJ, Huang AR, Tamblyn RM, Gayton DC. Defining inappropriate practices in prescribing for elderly people: a national consensus panel. CMAJ 1997; 156(3): 385–391
15	197	Oral prednisolone prescribed at a dose ≥7.5mg daily for more than 3 months to the over 65s without co-prescription of osteoporosis-preventing treatments	Process	Chronic	Treatment	Safe	L - Musculoskeletal	Oral prednisolone prescribed at a dose ≥7.5mg daily for more than 3 months to the over 65s without co-prescription of osteoporosis-preventing treatments	1. Spencer, R., Bell, B., Avery, A. J., Gookey, G., & Campbell, S. M. (2014). Identification of an updated set of prescribing-safety indicators for GPs. British Journal of General Practice, 64(621), e181–e190. // 2. Avery AJ, Rodgers S, Cantrill JA, et al. A pharmacist-led information technology intervention for medication errors (PINCER): a multicentre, cluster randomised, controlled trial and cost-effectiveness analysis. Lancet 2012; 379:1310–1319 // 1. Guthrie B, McCowan C, Davey P, et al. High risk prescribing in primary care patients particularly vulnerable to adverse drug events: cross sectional population database analysis in Scottish general practice. BMJ 2011; 342: d3514. // 3. Rognstad S, Brekke M, Fetveit A, et al. The Norwegian General Practice (NORGEP) criteria for assessing potentially inappropriate prescriptions to elderly patients. A modified Delphi study. Scand J Prim Health Care 2009; 27(3): 153–159. // 4. Gallagher P, Ryan C, Byrne S, et al. STOPP (Screening Tool of Older Person's Prescriptions) and START (Screening Tool to Alert doctors to Right Treatment). Consensus validation. Int J Clin Pharmacol Ther 2008; 46(2): 72–83. // 5. Basger BJ, Chen TF, Moles RJ. Inappropriate medication use and prescribing indicators in elderly Australians: development of a prescribing indicators tool. Drugs Aging 2008; 25(9): 777–793. // 6. Zhan C, Correa-de-Araujo R, Bierman AS, et al. Suboptimal prescribing in elderly outpatients: potentially harmful drug-drug and drug-disease combinations. J Am Geriatr Soc 2005; 53(2): 262–267. // 7. McLeod PJ, Huang AR, Tamblyn RM, Gayton DC. Defining inappropriate practices in prescribing for elderly people: a national consensus panel. CMAJ 1997; 156(3): 385–391
15	198	Patients on an angiotensin-converting-enzyme inhibitor or angiotensin II receptor antagonist who have not had a U+E in the previous 15 months	Process	Chronic	Treatment	Safe	K - Cardiovascular	Patients on an angiotensin-converting-enzyme inhibitor or angiotensin II receptor antagonist who have not had a U+E in the previous 15 months	1. Spencer, R., Bell, B., Avery, A. J., Gookey, G., & Campbell, S. M. (2014). Identification of an updated set of prescribing-safety indicators for GPs. British Journal of General Practice, 64(621), e181–e190. // 2. Avery AJ, Rodgers S, Cantrill JA, et al. A pharmacist-led information technology intervention for medication errors (PINCER): a multicentre, cluster randomised, controlled trial and cost-effectiveness analysis. Lancet 2012; 379:1310–1319 // 1. Guthrie B, McCowan C, Davey P, et al. High risk prescribing in primary care patients particularly vulnerable to adverse drug events: cross sectional population database analysis in Scottish general practice. BMJ 2011; 342: d3514. // 3. Rognstad S, Brekke M, Fetveit A, et al. The Norwegian General Practice (NORGEP) criteria for assessing potentially inappropriate prescriptions to elderly patients. A modified Delphi study. Scand J Prim Health Care 2009; 27(3): 153–159. // 4. Gallagher P, Ryan C, Byrne S, et al. STOPP (Screening Tool of Older Person's Prescriptions) and START (Screening Tool to Alert doctors to Right Treatment). Consensus validation. Int J Clin Pharmacol Ther 2008; 46(2): 72–83. // 5. Basger BJ, Chen TF, Moles RJ. Inappropriate medication use and prescribing indicators in elderly Australians: development of a prescribing indicators tool. Drugs Aging 2008; 25(9): 777–793. // 6. Zhan C, Correa-de-Araujo R, Bierman AS, et al. Suboptimal prescribing in elderly outpatients: potentially harmful drug-drug and drug-disease combinations. J Am Geriatr Soc 2005; 53(2): 262–267. // 7. McLeod PJ, Huang AR, Tamblyn RM, Gayton DC. Defining inappropriate practices in prescribing for elderly people: a national consensus panel. CMAJ 1997; 156(3): 385–391
15	199	Prescription of a Non-steroid anti-inflammatory drug, without co-prescription of an ulcer-healing drug, to a patient with a history of peptic ulceration	Process	Chronic	Treatment	Safe	D - Digestive	Prescription of a Non-steroid anti-inflammatory drug, without co-prescription of an ulcer-healing drug, to a patient with a history of peptic ulceration	1. Spencer, R., Bell, B., Avery, A. J., Gookey, G., & Campbell, S. M. (2014). Identification of an updated set of prescribing-safety indicators for GPs. British Journal of General Practice, 64(621), e181–e190. // 2. Avery AJ, Rodgers S, Cantrill JA, et al. A pharmacist-led information technology intervention for medication errors (PINCER): a multicentre, cluster randomised, controlled trial and cost-effectiveness analysis. Lancet 2012; 379:1310–1319 // 1. Guthrie B, McCowan C, Davey P, et al. High risk prescribing in primary care patients particularly vulnerable to adverse drug events: cross sectional population database analysis in Scottish general practice. BMJ 2011; 342: d3514. // 3. Rognstad S, Brekke M, Fetveit A, et al. The Norwegian General Practice (NORGEP) criteria for assessing potentially inappropriate prescriptions to elderly patients. A modified Delphi study. Scand J Prim Health Care 2009; 27(3): 153–159. // 4. Gallagher P, Ryan C, Byrne S, et al. STOPP (Screening Tool of Older Person's Prescriptions) and START (Screening Tool to Alert doctors to Right Treatment). Consensus validation. Int J Clin Pharmacol Ther 2008; 46(2): 72–83. // 5. Basger BJ, Chen TF, Moles RJ. Inappropriate medication use and prescribing indicators in elderly Australians: development of a prescribing indicators tool. Drugs Aging 2008; 25(9): 777–793. // 6. Zhan C, Correa-de-Araujo R, Bierman AS, et al. Suboptimal prescribing in elderly outpatients: potentially harmful drug-drug and drug-disease combinations. J Am Geriatr Soc 2005; 53(2): 262–267. // 7. McLeod PJ, Huang AR, Tamblyn RM, Gayton DC. Defining inappropriate practices in prescribing for elderly people: a national consensus panel. CMAJ 1997; 156(3): 385–391
15	200	Prescription of a phosphodiesterase type-5 inhibitor, for example sildenafil, to a patient who is also receiving a nitrate or nicorandil	Process	Chronic	Treatment	Safe	U - Urological	Prescription of a phosphodiesterase type-5 inhibitor, for example sildenafil, to a patient who is also receiving a nitrate or nicorandil	1. Spencer, R., Bell, B., Avery, A. J., Gookey, G., & Campbell, S. M. (2014). Identification of an updated set of prescribing-safety indicators for GPs. British Journal of General Practice, 64(621), e181–e190. // 2. Avery AJ, Rodgers S, Cantrill JA, et al. A pharmacist-led information technology intervention for medication errors (PINCER): a multicentre, cluster randomised, controlled trial and cost-effectiveness analysis. Lancet 2012; 379:1310–1319 // 1. Guthrie B, McCowan C, Davey P, et al. High risk prescribing in primary care patients particularly vulnerable to adverse drug events: cross sectional population database analysis in Scottish general practice. BMJ 2011; 342: d3514. // 3. Rognstad S, Brekke M, Fetveit A, et al. The Norwegian General Practice (NORGEP) criteria for assessing potentially inappropriate prescriptions to elderly patients. A modified Delphi study. Scand J Prim Health Care 2009; 27(3): 153–159. // 4. Gallagher P, Ryan C, Byrne S, et al. STOPP (Screening Tool of Older Person's Prescriptions) and START (Screening Tool to Alert doctors to Right Treatment). Consensus validation. Int J Clin Pharmacol Ther 2008; 46(2): 72–83. // 5. Basger BJ, Chen TF, Moles RJ. Inappropriate medication use and prescribing indicators in elderly Australians: development of a prescribing indicators tool. Drugs Aging 2008; 25(9): 777–793. // 6. Zhan C, Correa-de-Araujo R, Bierman AS, et al. Suboptimal prescribing in elderly outpatients: potentially harmful drug-drug and drug-disease combinations. J Am Geriatr Soc 2005; 53(2): 262–267. // 7. McLeod PJ, Huang AR, Tamblyn RM, Gayton DC. Defining inappropriate practices in prescribing for elderly people: a national consensus panel. CMAJ 1997; 156(3): 385–391
15	201	Prescription of a statin without an ALT taken prior to starting treatment	Process	Chronic	Treatment	Safe	D - Digestive	Prescription of a statin without an ALT taken prior to starting treatment	1. Spencer, R., Bell, B., Avery, A. J., Gookey, G., & Campbell, S. M. (2014). Identification of an updated set of prescribing-safety indicators for GPs. British Journal of General Practice, 64(621), e181–e190. // 2. Avery AJ, Rodgers S, Cantrill JA, et al. A pharmacist-led information technology intervention for medication errors (PINCER): a multicentre, cluster randomised, controlled trial and cost-effectiveness analysis. Lancet 2012; 379:1310–1319 // 1. Guthrie B, McCowan C, Davey P, et al. High risk prescribing in primary care patients particularly vulnerable to adverse drug events: cross sectional population database analysis in Scottish general practice. BMJ 2011; 342: d3514. // 3. Rognstad S, Brekke M, Fetveit A, et al. The Norwegian General Practice (NORGEP) criteria for assessing potentially inappropriate prescriptions to elderly patients. A modified Delphi study. Scand J Prim Health Care 2009; 27(3): 153–159. // 4. Gallagher P, Ryan C, Byrne S, et al. STOPP (Screening Tool of Older Person's Prescriptions) and START (Screening Tool to Alert doctors to Right Treatment). Consensus validation. Int J Clin Pharmacol Ther 2008; 46(2): 72–83. // 5. Basger BJ, Chen TF, Moles RJ. Inappropriate medication use and prescribing indicators in elderly Australians: development of a prescribing indicators tool. Drugs Aging 2008; 25(9): 777–793. // 6. Zhan C, Correa-de-Araujo R, Bierman AS, et al. Suboptimal prescribing in elderly outpatients: potentially harmful drug-drug and drug-disease combinations. J Am Geriatr Soc 2005; 53(2): 262–267. // 7. McLeod PJ, Huang AR, Tamblyn RM, Gayton DC. Defining inappropriate practices in prescribing for elderly people: a national consensus panel. CMAJ 1997; 156(3): 385–391 // 8. Spencer R, Serumaga B. Concurrent macrolide and statin: a common interaction. Prescriber 2011; 22(17): 49–50. DOI: 10.1002/psb.796.

15	202	Prescription of a statin without an ALT taken prior to starting treatment and within 3 months of starting treatment	Process	Chronic	Treatment	Safe	D - Digestive	Prescription of a statin without an ALT taken prior to starting treatment and within 3 months of starting treatment	1. Spencer, R., Bell, B., Avery, A. J., Gookey, G., & Campbell, S. M. (2014). Identification of an updated set of prescribing-safety indicators for GPs. British Journal of General Practice, 64(621), e181–e190. // 2. Avery AJ, Rodgers S, Cantrill JA, et al. A pharmacist-led information technology intervention for medication errors (PINCER): a multicentre, cluster randomised, controlled trial and cost-effectiveness analysis. Lancet 2012; 379:1310–1319 // 1. Guthrie B, McCowan C, Davey P, et al. High risk prescribing in primary care patients particularly vulnerable to adverse drug events: cross sectional population database analysis in Scottish general practice. BMJ 2011; 342: d3514. // 3. Rognstad S, Brekke M, Fetveit A, et al. The Norwegian General Practice (NORGEP) criteria for assessing potentially inappropriate prescriptions to elderly patients. A modified Delphi study. Scand J Prim Health Care 2009; 27(3): 153–159. // 4. Gallagher P, Ryan C, Byrne S, et al. STOPP (Screening Tool of Older Person's Prescriptions) and START (Screening Tool to Alert doctors to Right Treatment). Consensus validation. Int J Clin Pharmacol Ther 2008; 46(2): 72–83. // 5. Basger BJ, Chen TF, Moles RJ. Inappropriate medication use and prescribing indicators in elderly Australians: development of a prescribing indicators tool. Drugs Aging 2008; 25(9): 777–793. // 6. Zhan C, Correa-de-Araujo R, Bierman AS, et al. Suboptimal prescribing in elderly outpatients: potentially harmful drug-drug and drug-disease combinations. J Am Geriatr Soc 2005; 53(2): 262–267. // 7. McLeod PJ, Huang AR, Tamblyn RM, Gayton DC. Defining inappropriate practices in prescribing for elderly people: a national consensus panel. CMAJ 1997; 156(3): 385–391 // 8. Spencer R, Serumaga B. Concurrent macrolide and statin: a common interaction. Prescriber 2011; 22(17): 49–50. DOI: 10.1002/psb.796.
15	203	Prescription of amiodarone without a record of liver function being measured in the previous 9 months	Process	Chronic	Treatment	Safe	K - Cardiovascular	Prescription of amiodarone without a record of liver function being measured in the previous 9 months	1. Spencer, R., Bell, B., Avery, A. J., Gookey, G., & Campbell, S. M. (2014). Identification of an updated set of prescribing-safety indicators for GPs. British Journal of General Practice, 64(621), e181–e190. // 2. Avery AJ, Rodgers S, Cantrill JA, et al. A pharmacist-led information technology intervention for medication errors (PINCER): a multicentre, cluster randomised, controlled trial and cost-effectiveness analysis. Lancet 2012; 379:1310–1319 // 1. Guthrie B, McCowan C, Davey P, et al. High risk prescribing in primary care patients particularly vulnerable to adverse drug events: cross sectional population database analysis in Scottish general practice. BMJ 2011; 342: d3514. // 3. Rognstad S, Brekke M, Fetveit A, et al. The Norwegian General Practice (NORGEP) criteria for assessing potentially inappropriate prescriptions to elderly patients. A modified Delphi study. Scand J Prim Health Care 2009; 27(3): 153–159. // 4. Gallagher P, Ryan C, Byrne S, et al. STOPP (Screening Tool of Older Person's Prescriptions) and START (Screening Tool to Alert doctors to Right Treatment). Consensus validation. Int J Clin Pharmacol Ther 2008; 46(2): 72–83. // 5. Basger BJ, Chen TF, Moles RJ. Inappropriate medication use and prescribing indicators in elderly Australians: development of a prescribing indicators tool. Drugs Aging 2008; 25(9): 777–793. // 6. Zhan C, Correa-de-Araujo R, Bierman AS, et al. Suboptimal prescribing in elderly outpatients: potentially harmful drug-drug and drug-disease combinations. J Am Geriatr Soc 2005; 53(2): 262–267. // 7. McLeod PJ, Huang AR, Tamblyn RM, Gayton DC. Defining inappropriate practices in prescribing for elderly people: a national consensus panel. CMAJ 1997; 156(3): 385–391
15	204	Prescription of amiodarone without a record of thyroid function being measured within the previous 9 months	Process	Chronic	Treatment	Safe	K - Cardiovascular	Prescription of amiodarone without a record of thyroid function being measured within the previous 9 months	1. Spencer, R., Bell, B., Avery, A. J., Gookey, G., & Campbell, S. M. (2014). Identification of an updated set of prescribing-safety indicators for GPs. British Journal of General Practice, 64(621), e181–e190. // 2. Avery AJ, Rodgers S, Cantrill JA, et al. A pharmacist-led information technology intervention for medication errors (PINCER): a multicentre, cluster randomised, controlled trial and cost-effectiveness analysis. Lancet 2012; 379:1310–1319 // 1. Guthrie B, McCowan C, Davey P, et al. High risk prescribing in primary care patients particularly vulnerable to adverse drug events: cross sectional population database analysis in Scottish general practice. BMJ 2011; 342: d3514. // 3. Rognstad S, Brekke M, Fetveit A, et al. The Norwegian General Practice (NORGEP) criteria for assessing potentially inappropriate prescriptions to elderly patients. A modified Delphi study. Scand J Prim Health Care 2009; 27(3): 153–159. // 4. Gallagher P, Ryan C, Byrne S, et al. STOPP (Screening Tool of Older Person's Prescriptions) and START (Screening Tool to Alert doctors to Right Treatment). Consensus validation. Int J Clin Pharmacol Ther 2008; 46(2): 72–83. // 5. Basger BJ, Chen TF, Moles RJ. Inappropriate medication use and prescribing indicators in elderly Australians: development of a prescribing indicators tool. Drugs Aging 2008; 25(9): 777–793. // 6. Zhan C, Correa-de-Araujo R, Bierman AS, et al. Suboptimal prescribing in elderly outpatients: potentially harmful drug-drug and drug-disease combinations. J Am Geriatr Soc 2005; 53(2): 262–267. // 7. McLeod PJ, Huang AR, Tamblyn RM, Gayton DC. Defining inappropriate practices in prescribing for elderly people: a national consensus panel. CMAJ 1997; 156(3): 385–391
15	205	Prescription of an angiotensin-converting-enzyme inhibitor or angiotensin II receptor antagonist without a record of renal function and electrolytes being measured prior to starting therapy	Process	Chronic	Treatment	Safe	K - Cardiovascular	Prescription of an angiotensin-converting-enzyme inhibitor or angiotensin II receptor antagonist without a record of renal function and electrolytes being measured prior to starting therapy	1. Spencer, R., Bell, B., Avery, A. J., Gookey, G., & Campbell, S. M. (2014). Identification of an updated set of prescribing-safety indicators for GPs. British Journal of General Practice, 64(621), e181–e190. // 2. Avery AJ, Rodgers S, Cantrill JA, et al. A pharmacist-led information technology intervention for medication errors (PINCER): a multicentre, cluster randomised, controlled trial and cost-effectiveness analysis. Lancet 2012; 379:1310–1319 // 1. Guthrie B, McCowan C, Davey P, et al. High risk prescribing in primary care patients particularly vulnerable to adverse drug events: cross sectional population database analysis in Scottish general practice. BMJ 2011; 342: d3514. // 3. Rognstad S, Brekke M, Fetveit A, et al. The Norwegian General Practice (NORGEP) criteria for assessing potentially inappropriate prescriptions to elderly patients. A modified Delphi study. Scand J Prim Health Care 2009; 27(3): 153–159. // 4. Gallagher P, Ryan C, Byrne S, et al. STOPP (Screening Tool of Older Person's Prescriptions) and START (Screening Tool to Alert doctors to Right Treatment). Consensus validation. Int J Clin Pharmacol Ther 2008; 46(2): 72–83. // 5. Basger BJ, Chen TF, Moles RJ. Inappropriate medication use and prescribing indicators in elderly Australians: development of a prescribing indicators tool. Drugs Aging 2008; 25(9): 777–793. // 6. Zhan C, Correa-de-Araujo R, Bierman AS, et al. Suboptimal prescribing in elderly outpatients: potentially harmful drug-drug and drug-disease combinations. J Am Geriatr Soc 2005; 53(2): 262–267. // 7. McLeod PJ, Huang AR, Tamblyn RM, Gayton DC. Defining inappropriate practices in prescribing for elderly people: a national consensus panel. CMAJ 1997; 156(3): 385–391
15	206	Prescription of aspirin at a dose >75mg daily for ≥1 month in a patient aged >65 years	Process	Chronic	Treatment	Safe	K - Cardiovascular	Prescription of aspirin at a dose >75mg daily for ≥1 month in a patient aged >65 years	1. Spencer, R., Bell, B., Avery, A. J., Gookey, G., & Campbell, S. M. (2014). Identification of an updated set of prescribing-safety indicators for GPs. British Journal of General Practice, 64(621), e181–e190. // 2. Avery AJ, Rodgers S, Cantrill JA, et al. A pharmacist-led information technology intervention for medication errors (PINCER): a multicentre, cluster randomised, controlled trial and cost-effectiveness analysis. Lancet 2012; 379:1310–1319 // 1. Guthrie B, McCowan C, Davey P, et al. High risk prescribing in primary care patients particularly vulnerable to adverse drug events: cross sectional population database analysis in Scottish general practice. BMJ 2011; 342: d3514. // 3. Rognstad S, Brekke M, Fetveit A, et al. The Norwegian General Practice (NORGEP) criteria for assessing potentially inappropriate prescriptions to elderly patients. A modified Delphi study. Scand J Prim Health Care 2009; 27(3): 153–159. // 4. Gallagher P, Ryan C, Byrne S, et al. STOPP (Screening Tool of Older Person's Prescriptions) and START (Screening Tool to Alert doctors to Right Treatment). Consensus validation. Int J Clin Pharmacol Ther 2008; 46(2): 72–83. // 5. Basger BJ, Chen TF, Moles RJ. Inappropriate medication use and prescribing indicators in elderly Australians: development of a prescribing indicators tool. Drugs Aging 2008; 25(9): 777–793. // 6. Zhan C, Correa-de-Araujo R, Bierman AS, et al. Suboptimal prescribing in elderly outpatients: potentially harmful drug-drug and drug-disease combinations. J Am Geriatr Soc 2005; 53(2): 262–267. // 7. McLeod PJ, Huang AR, Tamblyn RM, Gayton DC. Defining inappropriate practices in prescribing for elderly people: a national consensus panel. CMAJ 1997; 156(3): 385–391
15	207	Prescription of aspirin to a child aged ≤16 years	Process	Chronic	Treatment	Safe	K - Cardiovascular	Prescription of aspirin to a child aged ≤16 years	1. Spencer, R., Bell, B., Avery, A. J., Gookey, G., & Campbell, S. M. (2014). Identification of an updated set of prescribing-safety indicators for GPs. British Journal of General Practice, 64(621), e181–e190. // 2. Avery AJ, Rodgers S, Cantrill JA, et al. A pharmacist-led information technology intervention for medication errors (PINCER): a multicentre, cluster randomised, controlled trial and cost-effectiveness analysis. Lancet 2012; 379:1310–1319 // 1. Guthrie B, McCowan C, Davey P, et al. High risk prescribing in primary care patients particularly vulnerable to adverse drug events: cross sectional population database analysis in Scottish general practice. BMJ 2011; 342: d3514. // 3. Rognstad S, Brekke M, Fetveit A, et al. The Norwegian General Practice (NORGEP) criteria for assessing potentially inappropriate prescriptions to elderly patients. A modified Delphi study. Scand J Prim Health Care 2009; 27(3): 153–159. // 4. Gallagher P, Ryan C, Byrne S, et al. STOPP (Screening Tool of Older Person's Prescriptions) and START (Screening Tool to Alert doctors to Right Treatment). Consensus validation. Int J Clin Pharmacol Ther 2008; 46(2): 72–83. // 5. Basger BJ, Chen TF, Moles RJ. Inappropriate medication use and prescribing indicators in elderly Australians: development of a prescribing indicators tool. Drugs Aging 2008; 25(9): 777–793. // 6. Zhan C, Correa-de-Araujo R, Bierman AS, et al. Suboptimal prescribing in elderly outpatients: potentially harmful drug-drug and drug-disease combinations. J Am Geriatr Soc 2005; 53(2): 262–267. // 7. McLeod PJ, Huang AR, Tamblyn RM, Gayton DC. Defining inappropriate practices in prescribing for elderly people: a national consensus panel. CMAJ 1997; 156(3): 385–391
15	208	Prescription of lithium without a record of a lithium level being measured within the previous 6 months	Process	Chronic	Treatment	Safe	P - Psychological	Prescription of lithium without a record of a lithium level being measured within the previous 6 months	1. Spencer, R., Bell, B., Avery, A. J., Gookey, G., & Campbell, S. M. (2014). Identification of an updated set of prescribing-safety indicators for GPs. British Journal of General Practice, 64(621), e181–e190. // 2. Avery AJ, Rodgers S, Cantrill JA, et al. A pharmacist-led information technology intervention for medication errors (PINCER): a multicentre, cluster randomised, controlled trial and cost-effectiveness analysis. Lancet 2012; 379:1310–1319 // 1. Guthrie B, McCowan C, Davey P, et al. High risk prescribing in primary care patients particularly vulnerable to adverse drug events: cross sectional population database analysis in Scottish general practice. BMJ 2011; 342: d3514. // 3. Rognstad S, Brekke M, Fetveit A, et al. The Norwegian General Practice (NORGEP) criteria for assessing potentially inappropriate prescriptions to elderly patients. A modified Delphi study. Scand J Prim Health Care 2009; 27(3): 153–159. // 4. Gallagher P, Ryan C, Byrne S, et al. STOPP (Screening Tool of Older Person's Prescriptions) and START (Screening Tool to Alert doctors to Right Treatment). Consensus validation. Int J Clin Pharmacol Ther 2008; 46(2): 72–83. // 5. Basger BJ, Chen TF, Moles RJ. Inappropriate medication use and prescribing indicators in elderly Australians: development of a prescribing indicators tool. Drugs Aging 2008; 25(9): 777–793. // 6. Zhan C, Correa-de-Araujo R, Bierman AS, et al. Suboptimal prescribing in elderly outpatients: potentially harmful drug-drug and drug-disease combinations. J Am Geriatr Soc 2005; 53(2): 262–267. // 7. McLeod PJ, Huang AR, Tamblyn RM, Gayton DC. Defining inappropriate practices in prescribing for elderly people: a national consensus panel. CMAJ 1997; 156(3): 385–391
15	209	Prescription of methotrexate without a record of a full blood count within the previous 3 months	Process	Chronic	Treatment	Safe	L - Musculoskeletal	Prescription of methotrexate without a record of a full blood count within the previous 3 months	1. Spencer, R., Bell, B., Avery, A. J., Gookey, G., & Campbell, S. M. (2014). Identification of an updated set of prescribing-safety indicators for GPs. British Journal of General Practice, 64(621), e181–e190. // 2. Avery AJ, Rodgers S, Cantrill JA, et al. A pharmacist-led information technology intervention for medication errors (PINCER): a multicentre, cluster randomised, controlled trial and cost-effectiveness analysis. Lancet 2012; 379:1310–1319 // 1. Guthrie B, McCowan C, Davey P, et al. High risk prescribing in primary care patients particularly vulnerable to adverse drug events: cross sectional population database analysis in Scottish general practice. BMJ 2011; 342: d3514. // 3. Rognstad S, Brekke M, Fetveit A, et al. The Norwegian General Practice (NORGEP) criteria for assessing potentially inappropriate prescriptions to elderly patients. A modified Delphi study. Scand J Prim Health Care 2009; 27(3): 153–159. // 4. Gallagher P, Ryan C, Byrne S, et al. STOPP (Screening Tool of Older Person's Prescriptions) and START (Screening Tool to Alert doctors to Right Treatment). Consensus validation. Int J Clin Pharmacol Ther 2008; 46(2): 72–83. // 5. Basger BJ, Chen TF, Moles RJ. Inappropriate medication use and prescribing indicators in elderly Australians: development of a prescribing indicators tool. Drugs Aging 2008; 25(9): 777–793. // 6. Zhan C, Correa-de-Araujo R, Bierman AS, et al. Suboptimal prescribing in elderly outpatients: potentially harmful drug-drug and drug-disease combinations. J Am Geriatr Soc 2005; 53(2): 262–267. // 7. McLeod PJ, Huang AR, Tamblyn RM, Gayton DC. Defining inappropriate practices in prescribing for elderly people: a national consensus panel. CMAJ 1997; 156(3): 385–391
15	210	Prescription of methotrexate without a record of liver function having been measured within the previous 3 months	Process	Chronic	Treatment	Safe	D - Digestive	Prescription of methotrexate without a record of liver function having been measured within the previous 3 months	1. Spencer, R., Bell, B., Avery, A. J., Gookey, G., & Campbell, S. M. (2014). Identification of an updated set of prescribing-safety indicators for GPs. British Journal of General Practice, 64(621), e181–e190. // 2. Avery AJ, Rodgers S, Cantrill JA, et al. A pharmacist-led information technology intervention for medication errors (PINCER): a multicentre, cluster randomised, controlled trial and cost-effectiveness analysis. Lancet 2012; 379:1310–1319 // 1. Guthrie B, McCowan C, Davey P, et al. High risk prescribing in primary care patients particularly vulnerable to adverse drug events: cross sectional population database analysis in Scottish general practice. BMJ 2011; 342: d3514. // 3. Rognstad S, Brekke M, Fetveit A, et al. The Norwegian General Practice (NORGEP) criteria for assessing potentially inappropriate prescriptions to elderly patients. A modified Delphi study. Scand J Prim Health Care 2009; 27(3): 153–159. // 4. Gallagher P, Ryan C, Byrne S, et al. STOPP (Screening Tool of Older Person's Prescriptions) and START (Screening Tool to Alert doctors to Right Treatment). Consensus validation. Int J Clin Pharmacol Ther 2008; 46(2): 72–83. // 5. Basger BJ, Chen TF, Moles RJ. Inappropriate medication use and prescribing indicators in elderly Australians: development of a prescribing indicators tool. Drugs Aging 2008; 25(9): 777–793. // 6. Zhan C, Correa-de-Araujo R, Bierman AS, et al. Suboptimal prescribing in elderly outpatients: potentially harmful drug-drug and drug-disease combinations. J Am Geriatr Soc 2005; 53(2): 262–267. // 7. McLeod PJ, Huang AR, Tamblyn RM, Gayton DC. Defining inappropriate practices in prescribing for elderly people: a national consensus panel. CMAJ 1997; 156(3): 385–391
15	211	Prescription of verapamil to a patient who is also receiving a beta-blocker	Process	Chronic	Treatment	Safe	K - Cardiovascular	Prescription of verapamil to a patient who is also receiving a beta-blocker	1. Spencer, R., Bell, B., Avery, A. J., Gookey, G., & Campbell, S. M. (2014). Identification of an updated set of prescribing-safety indicators for GPs. British Journal of General Practice, 64(621), e181–e190. // 2. Avery AJ, Rodgers S, Cantrill JA, et al. A pharmacist-led information technology intervention for medication errors (PINCER): a multicentre, cluster randomised, controlled trial and cost-effectiveness analysis. Lancet 2012; 379:1310–1319 // 1. Guthrie B, McCowan C, Davey P, et al. High risk prescribing in primary care patients particularly vulnerable to adverse drug events: cross sectional population database analysis in Scottish general practice. BMJ 2011; 342: d3514. // 3. Rognstad S, Brekke M, Fetveit A, et al. The Norwegian General Practice (NORGEP) criteria for assessing potentially inappropriate prescriptions to elderly patients. A modified Delphi study. Scand J Prim Health Care 2009; 27(3): 153–159. // 4. Gallagher P, Ryan C, Byrne S, et al. STOPP (Screening Tool of Older Person's Prescriptions) and START (Screening Tool to Alert doctors to Right Treatment). Consensus validation. Int J Clin Pharmacol Ther 2008; 46(2): 72–83. // 5. Basger BJ, Chen TF, Moles RJ. Inappropriate medication use and prescribing indicators in elderly Australians: development of a prescribing indicators tool. Drugs Aging 2008; 25(9): 777–793. // 6. Zhan C, Correa-de-Araujo R, Bierman AS, et al. Suboptimal prescribing in elderly outpatients: potentially harmful drug-drug and drug-disease combinations. J Am Geriatr Soc 2005; 53(2): 262–267. // 7. McLeod PJ, Huang AR, Tamblyn RM, Gayton DC. Defining inappropriate practices in prescribing for elderly people: a national consensus panel. CMAJ 1997; 156(3): 385–391
15	212	Prescription of warfarin and aspirin in combination (without co-prescription of gastroprotection)	Process	Chronic	Treatment	Safe	K - Cardiovascular	Prescription of warfarin and aspirin in combination (without co-prescription of gastroprotection)	1. Spencer, R., Bell, B., Avery, A. J., Gookey, G., & Campbell, S. M. (2014). Identification of an updated set of prescribing-safety indicators for GPs. British Journal of General Practice, 64(621), e181–e190. // 2. Avery AJ, Rodgers S, Cantrill JA, et al. A pharmacist-led information technology intervention for medication errors (PINCER): a multicentre, cluster randomised, controlled trial and cost-effectiveness analysis. Lancet 2012; 379:1310–1319 // 1. Guthrie B, McCowan C, Davey P, et al. High risk prescribing in primary care patients particularly vulnerable to adverse drug events: cross sectional population database analysis in Scottish general practice. BMJ 2011; 342: d3514. // 3. Rognstad S, Brekke M, Fetveit A, et al. The Norwegian General Practice (NORGEP) criteria for assessing potentially inappropriate prescriptions to elderly patients. A modified Delphi study. Scand J Prim Health Care 2009; 27(3): 153–159. // 4. Gallagher P, Ryan C, Byrne S, et al. STOPP (Screening Tool of Older Person's Prescriptions) and START (Screening Tool to Alert doctors to Right Treatment). Consensus validation. Int J Clin Pharmacol Ther 2008; 46(2): 72–83. // 5. Basger BJ, Chen TF, Moles RJ. Inappropriate medication use and prescribing indicators in elderly Australians: development of a prescribing indicators tool. Drugs Aging 2008; 25(9): 777–793. // 6. Zhan C, Correa-de-Araujo R, Bierman AS, et al. Suboptimal prescribing in elderly outpatients: potentially harmful drug-drug and drug-disease combinations. J Am Geriatr Soc 2005; 53(2): 262–267. // 7. McLeod PJ, Huang AR, Tamblyn RM, Gayton DC. Defining inappropriate practices in prescribing for elderly people: a national consensus panel. CMAJ 1997; 156(3): 385–391
15	213	Prescription of warfarin in combination with an oral Non-steroid anti-inflammatory drug	Process	Chronic	Treatment	Safe	K - Cardiovascular	Prescription of warfarin in combination with an oral Non-steroid anti-inflammatory drug	1. Spencer, R., Bell, B., Avery, A. J., Gookey, G., & Campbell, S. M. (2014). Identification of an updated set of prescribing-safety indicators for GPs. British Journal of General Practice, 64(621), e181–e190. // 2. Avery AJ, Rodgers S, Cantrill JA, et al. A pharmacist-led information technology intervention for medication errors (PINCER): a multicentre, cluster randomised, controlled trial and cost-effectiveness analysis. Lancet 2012; 379:1310–1319 // 1. Guthrie B, McCowan C, Davey P, et al. High risk prescribing in primary care patients particularly vulnerable to adverse drug events: cross sectional population database analysis in Scottish general practice. BMJ 2011; 342: d3514. // 3. Rognstad S, Brekke M, Fetveit A, et al. The Norwegian General Practice (NORGEP) criteria for assessing potentially inappropriate prescriptions to elderly patients. A modified Delphi study. Scand J Prim Health Care 2009; 27(3): 153–159. // 4. Gallagher P, Ryan C, Byrne S, et al. STOPP (Screening Tool of Older Person's Prescriptions) and START (Screening Tool to Alert doctors to Right Treatment). Consensus validation. Int J Clin Pharmacol Ther 2008; 46(2): 72–83. // 5. Basger BJ, Chen TF, Moles RJ. Inappropriate medication use and prescribing indicators in elderly Australians: development of a prescribing indicators tool. Drugs Aging 2008; 25(9): 777–793. // 6. Zhan C, Correa-de-Araujo R, Bierman AS, et al. Suboptimal prescribing in elderly outpatients: potentially harmful drug-drug and drug-disease combinations. J Am Geriatr Soc 2005; 53(2): 262–267. // 7. McLeod PJ, Huang AR, Tamblyn RM, Gayton DC. Defining inappropriate practices in prescribing for elderly people: a national consensus panel. CMAJ 1997; 156(3): 385–391
15	214	Prescription of warfarin to a patient without a record of INR having been measured within the previous 12 weeks (excluding patients who self-monitor)	Process	Chronic	Treatment	Safe	K - Cardiovascular	Prescription of warfarin to a patient without a record of INR having been measured within the previous 12 weeks (excluding patients who self-monitor)	1. Spencer, R., Bell, B., Avery, A. J., Gookey, G., & Campbell, S. M. (2014). Identification of an updated set of prescribing-safety indicators for GPs. British Journal of General Practice, 64(621), e181–e190. // 2. Avery AJ, Rodgers S, Cantrill JA, et al. A pharmacist-led information technology intervention for medication errors (PINCER): a multicentre, cluster randomised, controlled trial and cost-effectiveness analysis. Lancet 2012; 379:1310–1319 // 1. Guthrie B, McCowan C, Davey P, et al. High risk prescribing in primary care patients particularly vulnerable to adverse drug events: cross sectional population database analysis in Scottish general practice. BMJ 2011; 342: d3514. // 3. Rognstad S, Brekke M, Fetveit A, et al. The Norwegian General Practice (NORGEP) criteria for assessing potentially inappropriate prescriptions to elderly patients. A modified Delphi study. Scand J Prim Health Care 2009; 27(3): 153–159. // 4. Gallagher P, Ryan C, Byrne S, et al. STOPP (Screening Tool of Older Person's Prescriptions) and START (Screening Tool to Alert doctors to Right Treatment). Consensus validation. Int J Clin Pharmacol Ther 2008; 46(2): 72–83. // 5. Basger BJ, Chen TF, Moles RJ. Inappropriate medication use and prescribing indicators in elderly Australians: development of a prescribing indicators tool. Drugs Aging 2008; 25(9): 777–793. // 6. Zhan C, Correa-de-Araujo R, Bierman AS, et al. Suboptimal prescribing in elderly outpatients: potentially harmful drug-drug and drug-disease combinations. J Am Geriatr Soc 2005; 53(2): 262–267. // 7. McLeod PJ, Huang AR, Tamblyn RM, Gayton DC. Defining inappropriate practices in prescribing for elderly people: a national consensus panel. CMAJ 1997; 156(3): 385–391
17	215	Addresses medicines-use issues appropriately (i.e. correct application of professional knowledge to patient's situation)	Process	Chronic	Treatment	Safe	Not Defined	Addresses medicines-use issues appropriately (i.e. correct application of professional knowledge to patient's situation)	1. James, D. H., Hatten, S., Roberts, D., & John, D. N. (2008). Identifying criteria for assessing the quality of medicines use review referral documentation by community pharmacists. International Journal of Pharmacy Practice, 16(6), 365–374. // 2Bellingham C. How to offer a medicines use review. Pharm J 2004;273:602. // 3. Campbell SM, Cantrill JA, Roberts D. Prescribing indicators for UK general practice: Delphi consultation study. BMJ 2000;321:425–8. // 4. Holland R, Smith R, Harvey I. Where now for pharmacist led medication review? J Epidemiol Community Health 2006;60:92–3. // 5. Petty D. Medication review by pharmacists – the evidence still suggests benefit. Pharm J 2005;274:618–19. // 6. Oluwole-Ojo S. Advanced Services Medication Use Review. 2005. www.npa.co.uk/publications/nhsdev/PCO-resources/advanced/wandsworth/ADVANCED-SERVICES-WPCT. ppt#256,1,ADVANCED SERVICES (accessed August 29, 2007). // 7. Bellingham C. How to offer a medicines use review. Pharm J 2004;273:602. // 8. Lewis T. Using the NO TEARS tool for medication review. BMJ 2004;329:434. // 9. Alexander A. General tips for successful MURs. 2005. www. psnc.org.uk/uploaded_txt/CPNews%20June05%20.pdf? PHPSESSID= e56df697ce0f89b3a112cba6b80036c (accessed August 29, 2007). // 10. MHRA. Medicines use review: understand your medicines.2006. www.mhra.gov.uk (accessed August 29, 2007).
17	216	Bases clinical recommendations on national or local guidelines	Process	All	All	Safe	Not Defined	Bases clinical recommendations on national or local guidelines	1. James, D. H., Hatten, S., Roberts, D., & John, D. N. (2008). Identifying criteria for assessing the quality of medicines use review referral documentation by community pharmacists. International Journal of Pharmacy Practice, 16(6), 365–374. // 2Bellingham C. How to offer a medicines use review. Pharm J 2004;273:602. // 3. Campbell SM, Cantrill JA, Roberts D. Prescribing indicators for UK general practice: Delphi consultation study. BMJ 2000;321:425–8. // 4. Holland R, Smith R, Harvey I. Where now for pharmacist led medication review? J Epidemiol Community Health 2006;60:92–3. // 5. Petty D. Medication review by pharmacists – the evidence still suggests benefit. Pharm J 2005;274:618–19. // 6. Oluwole-Ojo S. Advanced Services Medication Use Review. 2005. www.npa.co.uk/publications/nhsdev/PCO-resources/advanced/wandsworth/ADVANCED-SERVICES-WPCT. ppt#256,1,ADVANCED SERVICES (accessed August 29, 2007). // 7. Bellingham C. How to offer a medicines use review. Pharm J 2004;273:602. // 8. Lewis T. Using the NO TEARS tool for medication review. BMJ 2004;329:434. // 9. Alexander A. General tips for successful MURs. 2005. www. psnc.org.uk/uploaded_txt/CPNews%20June05%20.pdf? PHPSESSID= e56df697ce0f89b3a112cba6b80036c (accessed August 29, 2007). // 10. MHRA. Medicines use review: understand your medicines.2006. www.mhra.gov.uk (accessed August 29, 2007).
17	217	Differentiates appropriately between important and minor issues	Process	All	Treatment	Safe	Not Defined	Differentiates appropriately between important and minor issues	1. James, D. H., Hatten, S., Roberts, D., & John, D. N. (2008). Identifying criteria for assessing the quality of medicines use review referral documentation by community pharmacists. International Journal of Pharmacy Practice, 16(6), 365–374. // 2Bellingham C. How to offer a medicines use review. Pharm J 2004;273:602. // 3. Campbell SM, Cantrill JA, Roberts D. Prescribing indicators for UK general practice: Delphi consultation study. BMJ 2000;321:425–8. // 4. Holland R, Smith R, Harvey I. Where now for pharmacist led medication review? J Epidemiol Community Health 2006;60:92–3. // 5. Petty D. Medication review by pharmacists – the evidence still suggests benefit. Pharm J 2005;274:618–19. // 6. Oluwole-Ojo S. Advanced Services Medication Use Review. 2005. www.npa.co.uk/publications/nhsdev/PCO-resources/advanced/wandsworth/ADVANCED-SERVICES-WPCT. ppt#256,1,ADVANCED SERVICES (accessed August 29, 2007). // 7. Bellingham C. How to offer a medicines use review. Pharm J 2004;273:602. // 8. Lewis T. Using the NO TEARS tool for medication review. BMJ 2004;329:434. // 9. Alexander A. General tips for successful MURs. 2005. www. psnc.org.uk/uploaded_txt/CPNews%20June05%20.pdf? PHPSESSID= e56df697ce0f89b3a112cba6b80036c (accessed August 29, 2007). // 10. MHRA. Medicines use review: understand your medicines.2006. www.mhra.gov.uk (accessed August 29, 2007).
17	218	Does not raise patient expectations that recommendations will be implemented	Process	All	Treatment	Safe	Not Defined	Does not raise patient expectations that recommendations will be implemented	1. James, D. H., Hatten, S., Roberts, D., & John, D. N. (2008). Identifying criteria for assessing the quality of medicines use review referral documentation by community pharmacists. International Journal of Pharmacy Practice, 16(6), 365–374. // 2Bellingham C. How to offer a medicines use review. Pharm J 2004;273:602. // 3. Campbell SM, Cantrill JA, Roberts D. Prescribing indicators for UK general practice: Delphi consultation study. BMJ 2000;321:425–8. // 4. Holland R, Smith R, Harvey I. Where now for pharmacist led medication review? J Epidemiol Community Health 2006;60:92–3. // 5. Petty D. Medication review by pharmacists – the evidence still suggests benefit. Pharm J 2005;274:618–19. // 6. Oluwole-Ojo S. Advanced Services Medication Use Review. 2005. www.npa.co.uk/publications/nhsdev/PCO-resources/advanced/wandsworth/ADVANCED-SERVICES-WPCT. ppt#256,1,ADVANCED SERVICES (accessed August 29, 2007). // 7. Bellingham C. How to offer a medicines use review. Pharm J 2004;273:602. // 8. Lewis T. Using the NO TEARS tool for medication review. BMJ 2004;329:434. // 9. Alexander A. General tips for successful MURs. 2005. www. psnc.org.uk/uploaded_txt/CPNews%20June05%20.pdf? PHPSESSID= e56df697ce0f89b3a112cba6b80036c (accessed August 29, 2007). // 10. MHRA. Medicines use review: understand your medicines.2006. www.mhra.gov.uk (accessed August 29, 2007).

17	219	Endeavours to follow up the outcome of the medicines use review	Process	All	Follow up and continuity	Safe	Not Defined	Endeavours to follow up the outcome of the medicines use review	1. James, D. H., Hatten, S., Roberts, D., & John, D. N. (2008). Identifying criteria for assessing the quality of medicines use review referral documentation by community pharmacists. International Journal of Pharmacy Practice, 16(6), 365–374. // 2Bellingham C. How to offer a medicines use review. Pharm J 2004;273:602. // 3. Campbell SM, Cantrill JA, Roberts D. Prescribing indicators for UK general practice: Delphi consultation study. BMJ 2000;321:425–8. // 4. Holland R, Smith R, Harvey I. Where now for pharmacist led medication review? J Epidemiol Community Health 2006;60:92–3. // 5. Petty D. Medication review by pharmacists – the evidence still suggests benefit. Pharm J 2005;274:618–19. // 6. Oluwole-Ojo S. Advanced Services Medication Use Review. 2005. www.npa.co.uk/publications/nhsdev/PCO-resources/advanced/wandsworth/ADVANCED-SERVICES-WPCT. ppt#256,1,ADVANCED SERVICES (accessed August 29, 2007). // 7. Bellingham C. How to offer a medicines use review. Pharm J 2004;273:602. // 8. Lewis T. Using the NO TEARS tool for medication review. BMJ 2004;329:434. // 9. Alexander A. General tips for successful MURs. 2005. www. psnc.org.uk/uploaded_txt/CPNews%20June05%20.pdf? PHPSESSID= e56df697ce0f89b3a112cba6b80036c (accessed August 29, 2007). // 10. MHRA. Medicines use review: understand your medicines.2006. www.mhra.gov.uk (accessed August 29, 2007).
17	220	Fully describes the nature of the problem (rather than listing a disease or drug name)	Process	All	Treatment	Safe	Not Defined	Fully describes the nature of the problem (rather than listing a disease or drug name)	1. James, D. H., Hatten, S., Roberts, D., & John, D. N. (2008). Identifying criteria for assessing the quality of medicines use review referral documentation by community pharmacists. International Journal of Pharmacy Practice, 16(6), 365–374. // 2Bellingham C. How to offer a medicines use review. Pharm J 2004;273:602. // 3. Campbell SM, Cantrill JA, Roberts D. Prescribing indicators for UK general practice: Delphi consultation study. BMJ 2000;321:425–8. // 4. Holland R, Smith R, Harvey I. Where now for pharmacist led medication review? J Epidemiol Community Health 2006;60:92–3. // 5. Petty D. Medication review by pharmacists – the evidence still suggests benefit. Pharm J 2005;274:618–19. // 6. Oluwole-Ojo S. Advanced Services Medication Use Review. 2005. www.npa.co.uk/publications/nhsdev/PCO-resources/advanced/wandsworth/ADVANCED-SERVICES-WPCT. ppt#256,1,ADVANCED SERVICES (accessed August 29, 2007). // 7. Bellingham C. How to offer a medicines use review. Pharm J 2004;273:602. // 8. Lewis T. Using the NO TEARS tool for medication review. BMJ 2004;329:434. // 9. Alexander A. General tips for successful MURs. 2005. www. psnc.org.uk/uploaded_txt/CPNews%20June05%20.pdf? PHPSESSID= e56df697ce0f89b3a112cba6b80036c (accessed August 29, 2007). // 10. MHRA. Medicines use review: understand your medicines.2006. www.mhra.gov.uk (accessed August 29, 2007).
17	221	Liaises with relevant General Practicioner(s) before setting up medicines use review service	Process	All	Treatment	Safe	Not Defined	Liaises with relevant General Practicioner(s) before setting up medicines use review service	1. James, D. H., Hatten, S., Roberts, D., & John, D. N. (2008). Identifying criteria for assessing the quality of medicines use review referral documentation by community pharmacists. International Journal of Pharmacy Practice, 16(6), 365–374. // 2Bellingham C. How to offer a medicines use review. Pharm J 2004;273:602. // 3. Campbell SM, Cantrill JA, Roberts D. Prescribing indicators for UK general practice: Delphi consultation study. BMJ 2000;321:425–8. // 4. Holland R, Smith R, Harvey I. Where now for pharmacist led medication review? J Epidemiol Community Health 2006;60:92–3. // 5. Petty D. Medication review by pharmacists – the evidence still suggests benefit. Pharm J 2005;274:618–19. // 6. Oluwole-Ojo S. Advanced Services Medication Use Review. 2005. www.npa.co.uk/publications/nhsdev/PCO-resources/advanced/wandsworth/ADVANCED-SERVICES-WPCT. ppt#256,1,ADVANCED SERVICES (accessed August 29, 2007). // 7. Bellingham C. How to offer a medicines use review. Pharm J 2004;273:602. // 8. Lewis T. Using the NO TEARS tool for medication review. BMJ 2004;329:434. // 9. Alexander A. General tips for successful MURs. 2005. www. psnc.org.uk/uploaded_txt/CPNews%20June05%20.pdf? PHPSESSID= e56df697ce0f89b3a112cba6b80036c (accessed August 29, 2007). // 10. MHRA. Medicines use review: understand your medicines.2006. www.mhra.gov.uk (accessed August 29, 2007).
17	222	Pharmacist documents action(s) taken by themselves (e.g. provision of information)	Process	All	Treatment	Safe	Not Defined	Pharmacist documents action(s) taken by themselves (e.g. provision of information)	1. James, D. H., Hatten, S., Roberts, D., & John, D. N. (2008). Identifying criteria for assessing the quality of medicines use review referral documentation by community pharmacists. International Journal of Pharmacy Practice, 16(6), 365–374. // 2Bellingham C. How to offer a medicines use review. Pharm J 2004;273:602. // 3. Campbell SM, Cantrill JA, Roberts D. Prescribing indicators for UK general practice: Delphi consultation study. BMJ 2000;321:425–8. // 4. Holland R, Smith R, Harvey I. Where now for pharmacist led medication review? J Epidemiol Community Health 2006;60:92–3. // 5. Petty D. Medication review by pharmacists – the evidence still suggests benefit. Pharm J 2005;274:618–19. // 6. Oluwole-Ojo S. Advanced Services Medication Use Review. 2005. www.npa.co.uk/publications/nhsdev/PCO-resources/advanced/wandsworth/ADVANCED-SERVICES-WPCT. ppt#256,1,ADVANCED SERVICES (accessed August 29, 2007). // 7. Bellingham C. How to offer a medicines use review. Pharm J 2004;273:602. // 8. Lewis T. Using the NO TEARS tool for medication review. BMJ 2004;329:434. // 9. Alexander A. General tips for successful MURs. 2005. www. psnc.org.uk/uploaded_txt/CPNews%20June05%20.pdf? PHPSESSID= e56df697ce0f89b3a112cba6b80036c (accessed August 29, 2007). // 10. MHRA. Medicines use review: understand your medicines.2006. www.mhra.gov.uk (accessed August 29, 2007).
17	223	Presents issues in order of clinical importance (i.e. all high-priority issues presented first)	Process	All	Treatment	Safe	Not Defined	Presents issues in order of clinical importance (i.e. all high-priority issues presented first)	1. James, D. H., Hatten, S., Roberts, D., & John, D. N. (2008). Identifying criteria for assessing the quality of medicines use review referral documentation by community pharmacists. International Journal of Pharmacy Practice, 16(6), 365–374. // 2Bellingham C. How to offer a medicines use review. Pharm J 2004;273:602. // 3. Campbell SM, Cantrill JA, Roberts D. Prescribing indicators for UK general practice: Delphi consultation study. BMJ 2000;321:425–8. // 4. Holland R, Smith R, Harvey I. Where now for pharmacist led medication review? J Epidemiol Community Health 2006;60:92–3. // 5. Petty D. Medication review by pharmacists – the evidence still suggests benefit. Pharm J 2005;274:618–19. // 6. Oluwole-Ojo S. Advanced Services Medication Use Review. 2005. www.npa.co.uk/publications/nhsdev/PCO-resources/advanced/wandsworth/ADVANCED-SERVICES-WPCT. ppt#256,1,ADVANCED SERVICES (accessed August 29, 2007). // 7. Bellingham C. How to offer a medicines use review. Pharm J 2004;273:602. // 8. Lewis T. Using the NO TEARS tool for medication review. BMJ 2004;329:434. // 9. Alexander A. General tips for successful MURs. 2005. www. psnc.org.uk/uploaded_txt/CPNews%20June05%20.pdf? PHPSESSID= e56df697ce0f89b3a112cba6b80036c (accessed August 29, 2007). // 10. MHRA. Medicines use review: understand your medicines.2006. www.mhra.gov.uk (accessed August 29, 2007).
17	224	Presents issues without causing unnecessary anxiety to the patient	Process	All	Treatment	Safe	Not Defined	Presents issues without causing unnecessary anxiety to the patient	1. James, D. H., Hatten, S., Roberts, D., & John, D. N. (2008). Identifying criteria for assessing the quality of medicines use review referral documentation by community pharmacists. International Journal of Pharmacy Practice, 16(6), 365–374. // 2Bellingham C. How to offer a medicines use review. Pharm J 2004;273:602. // 3. Campbell SM, Cantrill JA, Roberts D. Prescribing indicators for UK general practice: Delphi consultation study. BMJ 2000;321:425–8. // 4. Holland R, Smith R, Harvey I. Where now for pharmacist led medication review? J Epidemiol Community Health 2006;60:92–3. // 5. Petty D. Medication review by pharmacists – the evidence still suggests benefit. Pharm J 2005;274:618–19. // 6. Oluwole-Ojo S. Advanced Services Medication Use Review. 2005. www.npa.co.uk/publications/nhsdev/PCO-resources/advanced/wandsworth/ADVANCED-SERVICES-WPCT. ppt#256,1,ADVANCED SERVICES (accessed August 29, 2007). // 7. Bellingham C. How to offer a medicines use review. Pharm J 2004;273:602. // 8. Lewis T. Using the NO TEARS tool for medication review. BMJ 2004;329:434. // 9. Alexander A. General tips for successful MURs. 2005. www. psnc.org.uk/uploaded_txt/CPNews%20June05%20.pdf? PHPSESSID= e56df697ce0f89b3a112cba6b80036c (accessed August 29, 2007). // 10. MHRA. Medicines use review: understand your medicines.2006. www.mhra.gov.uk (accessed August 29, 2007).
17	225	Presents issues without undermining the patient's confidence in their General Practicioner	Process	All	Treatment	Safe	Not Defined	Presents issues without undermining the patient's confidence in their General Practicioner	1. James, D. H., Hatten, S., Roberts, D., & John, D. N. (2008). Identifying criteria for assessing the quality of medicines use review referral documentation by community pharmacists. International Journal of Pharmacy Practice, 16(6), 365–374. // 2Bellingham C. How to offer a medicines use review. Pharm J 2004;273:602. // 3. Campbell SM, Cantrill JA, Roberts D. Prescribing indicators for UK general practice: Delphi consultation study. BMJ 2000;321:425–8. // 4. Holland R, Smith R, Harvey I. Where now for pharmacist led medication review? J Epidemiol Community Health 2006;60:92–3. // 5. Petty D. Medication review by pharmacists – the evidence still suggests benefit. Pharm J 2005;274:618–19. // 6. Oluwole-Ojo S. Advanced Services Medication Use Review. 2005. www.npa.co.uk/publications/nhsdev/PCO-resources/advanced/wandsworth/ADVANCED-SERVICES-WPCT. ppt#256,1,ADVANCED SERVICES (accessed August 29, 2007). // 7. Bellingham C. How to offer a medicines use review. Pharm J 2004;273:602. // 8. Lewis T. Using the NO TEARS tool for medication review. BMJ 2004;329:434. // 9. Alexander A. General tips for successful MURs. 2005. www. psnc.org.uk/uploaded_txt/CPNews%20June05%20.pdf? PHPSESSID= e56df697ce0f89b3a112cba6b80036c (accessed August 29, 2007). // 10. MHRA. Medicines use review: understand your medicines.2006. www.mhra.gov.uk (accessed August 29, 2007).
17	226	Presents no more than four issues and recommendations per patient	Process	All	Treatment	Safe	Not Defined	Presents no more than four issues and recommendations per patient	1. James, D. H., Hatten, S., Roberts, D., & John, D. N. (2008). Identifying criteria for assessing the quality of medicines use review referral documentation by community pharmacists. International Journal of Pharmacy Practice, 16(6), 365–374. // 2Bellingham C. How to offer a medicines use review. Pharm J 2004;273:602. // 3. Campbell SM, Cantrill JA, Roberts D. Prescribing indicators for UK general practice: Delphi consultation study. BMJ 2000;321:425–8. // 4. Holland R, Smith R, Harvey I. Where now for pharmacist led medication review? J Epidemiol Community Health 2006;60:92–3. // 5. Petty D. Medication review by pharmacists – the evidence still suggests benefit. Pharm J 2005;274:618–19. // 6. Oluwole-Ojo S. Advanced Services Medication Use Review. 2005. www.npa.co.uk/publications/nhsdev/PCO-resources/advanced/wandsworth/ADVANCED-SERVICES-WPCT. ppt#256,1,ADVANCED SERVICES (accessed August 29, 2007). // 7. Bellingham C. How to offer a medicines use review. Pharm J 2004;273:602. // 8. Lewis T. Using the NO TEARS tool for medication review. BMJ 2004;329:434. // 9. Alexander A. General tips for successful MURs. 2005. www. psnc.org.uk/uploaded_txt/CPNews%20June05%20.pdf? PHPSESSID= e56df697ce0f89b3a112cba6b80036c (accessed August 29, 2007). // 10. MHRA. Medicines use review: understand your medicines.2006. www.mhra.gov.uk (accessed August 29, 2007).
17	227	Presents one issue and recommendation per row of the documentation template	Process	All	Treatment	Safe	Not Defined	Presents one issue and recommendation per row of the documentation template	1. James, D. H., Hatten, S., Roberts, D., & John, D. N. (2008). Identifying criteria for assessing the quality of medicines use review referral documentation by community pharmacists. International Journal of Pharmacy Practice, 16(6), 365–374. // 2Bellingham C. How to offer a medicines use review. Pharm J 2004;273:602. // 3. Campbell SM, Cantrill JA, Roberts D. Prescribing indicators for UK general practice: Delphi consultation study. BMJ 2000;321:425–8. // 4. Holland R, Smith R, Harvey I. Where now for pharmacist led medication review? J Epidemiol Community Health 2006;60:92–3. // 5. Petty D. Medication review by pharmacists – the evidence still suggests benefit. Pharm J 2005;274:618–19. // 6. Oluwole-Ojo S. Advanced Services Medication Use Review. 2005. www.npa.co.uk/publications/nhsdev/PCO-resources/advanced/wandsworth/ADVANCED-SERVICES-WPCT. ppt#256,1,ADVANCED SERVICES (accessed August 29, 2007). // 7. Bellingham C. How to offer a medicines use review. Pharm J 2004;273:602. // 8. Lewis T. Using the NO TEARS tool for medication review. BMJ 2004;329:434. // 9. Alexander A. General tips for successful MURs. 2005. www. psnc.org.uk/uploaded_txt/CPNews%20June05%20.pdf? PHPSESSID= e56df697ce0f89b3a112cba6b80036c (accessed August 29, 2007). // 10. MHRA. Medicines use review: understand your medicines.2006. www.mhra.gov.uk (accessed August 29, 2007).
17	228	Provides a clear link between the proposed action and the problem (medicines-use issue) identified	Process	All	Treatment	Safe	Not Defined	Provides a clear link between the proposed action and the problem (medicines-use issue) identified	1. James, D. H., Hatten, S., Roberts, D., & John, D. N. (2008). Identifying criteria for assessing the quality of medicines use review referral documentation by community pharmacists. International Journal of Pharmacy Practice, 16(6), 365–374. // 2Bellingham C. How to offer a medicines use review. Pharm J 2004;273:602. // 3. Campbell SM, Cantrill JA, Roberts D. Prescribing indicators for UK general practice: Delphi consultation study. BMJ 2000;321:425–8. // 4. Holland R, Smith R, Harvey I. Where now for pharmacist led medication review? J Epidemiol Community Health 2006;60:92–3. // 5. Petty D. Medication review by pharmacists – the evidence still suggests benefit. Pharm J 2005;274:618–19. // 6. Oluwole-Ojo S. Advanced Services Medication Use Review. 2005. www.npa.co.uk/publications/nhsdev/PCO-resources/advanced/wandsworth/ADVANCED-SERVICES-WPCT. ppt#256,1,ADVANCED SERVICES (accessed August 29, 2007). // 7. Bellingham C. How to offer a medicines use review. Pharm J 2004;273:602. // 8. Lewis T. Using the NO TEARS tool for medication review. BMJ 2004;329:434. // 9. Alexander A. General tips for successful MURs. 2005. www. psnc.org.uk/uploaded_txt/CPNews%20June05%20.pdf? PHPSESSID= e56df697ce0f89b3a112cba6b80036c (accessed August 29, 2007). // 10. MHRA. Medicines use review: understand your medicines.2006. www.mhra.gov.uk (accessed August 29, 2007).
17	229	Selects patients for medicines use review appropriately (e.g. focuses on asthma patients)	Process	All	Treatment	Safe	R - Respiratory	Selects patients for medicines use review appropriately (e.g. focuses on asthma patients)	1. James, D. H., Hatten, S., Roberts, D., & John, D. N. (2008). Identifying criteria for assessing the quality of medicines use review referral documentation by community pharmacists. International Journal of Pharmacy Practice, 16(6), 365–374. // 2Bellingham C. How to offer a medicines use review. Pharm J 2004;273:602. // 3. Campbell SM, Cantrill JA, Roberts D. Prescribing indicators for UK general practice: Delphi consultation study. BMJ 2000;321:425–8. // 4. Holland R, Smith R, Harvey I. Where now for pharmacist led medication review? J Epidemiol Community Health 2006;60:92–3. // 5. Petty D. Medication review by pharmacists – the evidence still suggests benefit. Pharm J 2005;274:618–19. // 6. Oluwole-Ojo S. Advanced Services Medication Use Review. 2005. www.npa.co.uk/publications/nhsdev/PCO-resources/advanced/wandsworth/ADVANCED-SERVICES-WPCT. ppt#256,1,ADVANCED SERVICES (accessed August 29, 2007). // 7. Bellingham C. How to offer a medicines use review. Pharm J 2004;273:602. // 8. Lewis T. Using the NO TEARS tool for medication review. BMJ 2004;329:434. // 9. Alexander A. General tips for successful MURs. 2005. www. psnc.org.uk/uploaded_txt/CPNews%20June05%20.pdf? PHPSESSID= e56df697ce0f89b3a112cba6b80036c (accessed August 29, 2007). // 10. MHRA. Medicines use review: understand your medicines.2006. www.mhra.gov.uk (accessed August 29, 2007).
17	230	Shows awareness of different healthcare professionals within the primary care team (e.g. specialist nurse, supplementary prescriber, etc.)	Process	All	Treatment	Safe	Not Defined	Shows awareness of different healthcare professionals within the primary care team (e.g. specialist nurse, supplementary prescriber, etc.)	1. James, D. H., Hatten, S., Roberts, D., & John, D. N. (2008). Identifying criteria for assessing the quality of medicines use review referral documentation by community pharmacists. International Journal of Pharmacy Practice, 16(6), 365–374. // 2Bellingham C. How to offer a medicines use review. Pharm J 2004;273:602. // 3. Campbell SM, Cantrill JA, Roberts D. Prescribing indicators for UK general practice: Delphi consultation study. BMJ 2000;321:425–8. // 4. Holland R, Smith R, Harvey I. Where now for pharmacist led medication review? J Epidemiol Community Health 2006;60:92–3. // 5. Petty D. Medication review by pharmacists – the evidence still suggests benefit. Pharm J 2005;274:618–19. // 6. Oluwole-Ojo S. Advanced Services Medication Use Review. 2005. www.npa.co.uk/publications/nhsdev/PCO-resources/advanced/wandsworth/ADVANCED-SERVICES-WPCT. ppt#256,1,ADVANCED SERVICES (accessed August 29, 2007). // 7. Bellingham C. How to offer a medicines use review. Pharm J 2004;273:602. // 8. Lewis T. Using the NO TEARS tool for medication review. BMJ 2004;329:434. // 9. Alexander A. General tips for successful MURs. 2005. www. psnc.org.uk/uploaded_txt/CPNews%20June05%20.pdf? PHPSESSID= e56df697ce0f89b3a112cba6b80036c (accessed August 29, 2007). // 10. MHRA. Medicines use review: understand your medicines.2006. www.mhra.gov.uk (accessed August 29, 2007).
17	231	Summarises the issue succinctly	Process	All	Treatment	Safe	Not Defined	Summarises the issue succinctly	1. James, D. H., Hatten, S., Roberts, D., & John, D. N. (2008). Identifying criteria for assessing the quality of medicines use review referral documentation by community pharmacists. International Journal of Pharmacy Practice, 16(6), 365–374. // 2Bellingham C. How to offer a medicines use review. Pharm J 2004;273:602. // 3. Campbell SM, Cantrill JA, Roberts D. Prescribing indicators for UK general practice: Delphi consultation study. BMJ 2000;321:425–8. // 4. Holland R, Smith R, Harvey I. Where now for pharmacist led medication review? J Epidemiol Community Health 2006;60:92–3. // 5. Petty D. Medication review by pharmacists – the evidence still suggests benefit. Pharm J 2005;274:618–19. // 6. Oluwole-Ojo S. Advanced Services Medication Use Review. 2005. www.npa.co.uk/publications/nhsdev/PCO-resources/advanced/wandsworth/ADVANCED-SERVICES-WPCT. ppt#256,1,ADVANCED SERVICES (accessed August 29, 2007). // 7. Bellingham C. How to offer a medicines use review. Pharm J 2004;273:602. // 8. Lewis T. Using the NO TEARS tool for medication review. BMJ 2004;329:434. // 9. Alexander A. General tips for successful MURs. 2005. www. psnc.org.uk/uploaded_txt/CPNews%20June05%20.pdf? PHPSESSID= e56df697ce0f89b3a112cba6b80036c (accessed August 29, 2007). // 10. MHRA. Medicines use review: understand your medicines.2006. www.mhra.gov.uk (accessed August 29, 2007).
17	232	Uses appropriate language for the patient (i.e. without medical jargon and abbreviations)	Process	All	Treatment	Patient-centered	Not Defined	Uses appropriate language for the patient (i.e. without medical jargon and abbreviations)	1. James, D. H., Hatten, S., Roberts, D., & John, D. N. (2008). Identifying criteria for assessing the quality of medicines use review referral documentation by community pharmacists. International Journal of Pharmacy Practice, 16(6), 365–374. // 2Bellingham C. How to offer a medicines use review. Pharm J 2004;273:602. // 3. Campbell SM, Cantrill JA, Roberts D. Prescribing indicators for UK general practice: Delphi consultation study. BMJ 2000;321:425–8. // 4. Holland R, Smith R, Harvey I. Where now for pharmacist led medication review? J Epidemiol Community Health 2006;60:92–3. // 5. Petty D. Medication review by pharmacists – the evidence still suggests benefit. Pharm J 2005;274:618–19. // 6. Oluwole-Ojo S. Advanced Services Medication Use Review. 2005. www.npa.co.uk/publications/nhsdev/PCO-resources/advanced/wandsworth/ADVANCED-SERVICES-WPCT. ppt#256,1,ADVANCED SERVICES (accessed August 29, 2007). // 7. Bellingham C. How to offer a medicines use review. Pharm J 2004;273:602. // 8. Lewis T. Using the NO TEARS tool for medication review. BMJ 2004;329:434. // 9. Alexander A. General tips for successful MURs. 2005. www. psnc.org.uk/uploaded_txt/CPNews%20June05%20.pdf? PHPSESSID= e56df697ce0f89b3a112cba6b80036c (accessed August 29, 2007). // 10. MHRA. Medicines use review: understand your medicines.2006. www.mhra.gov.uk (accessed August 29, 2007).
17	233	Uses appropriate wording for the General Practice (i.e. providing suggestions rather than instructions)	Process	All	Treatment	Safe	Not Defined	Uses appropriate wording for the General Practice (i.e. providing suggestions rather than instructions)	1. James, D. H., Hatten, S., Roberts, D., & John, D. N. (2008). Identifying criteria for assessing the quality of medicines use review referral documentation by community pharmacists. International Journal of Pharmacy Practice, 16(6), 365–374. // 2Bellingham C. How to offer a medicines use review. Pharm J 2004;273:602. // 3. Campbell SM, Cantrill JA, Roberts D. Prescribing indicators for UK general practice: Delphi consultation study. BMJ 2000;321:425–8. // 4. Holland R, Smith R, Harvey I. Where now for pharmacist led medication review? J Epidemiol Community Health 2006;60:92–3. // 5. Petty D. Medication review by pharmacists – the evidence still suggests benefit. Pharm J 2005;274:618–19. // 6. Oluwole-Ojo S. Advanced Services Medication Use Review. 2005. www.npa.co.uk/publications/nhsdev/PCO-resources/advanced/wandsworth/ADVANCED-SERVICES-WPCT. ppt#256,1,ADVANCED SERVICES (accessed August 29, 2007). // 7. Bellingham C. How to offer a medicines use review. Pharm J 2004;273:602. // 8. Lewis T. Using the NO TEARS tool for medication review. BMJ 2004;329:434. // 9. Alexander A. General tips for successful MURs. 2005. www. psnc.org.uk/uploaded_txt/CPNews%20June05%20.pdf? PHPSESSID= e56df697ce0f89b3a112cba6b80036c (accessed August 29, 2007). // 10. MHRA. Medicines use review: understand your medicines.2006. www.mhra.gov.uk (accessed August 29, 2007).
17	234	Writes action plan legibly	Process	All	Treatment	Safe	Not Defined	Writes action plan legibly	1. James, D. H., Hatten, S., Roberts, D., & John, D. N. (2008). Identifying criteria for assessing the quality of medicines use review referral documentation by community pharmacists. International Journal of Pharmacy Practice, 16(6), 365–374. // 2Bellingham C. How to offer a medicines use review. Pharm J 2004;273:602. // 3. Campbell SM, Cantrill JA, Roberts D. Prescribing indicators for UK general practice: Delphi consultation study. BMJ 2000;321:425–8. // 4. Holland R, Smith R, Harvey I. Where now for pharmacist led medication review? J Epidemiol Community Health 2006;60:92–3. // 5. Petty D. Medication review by pharmacists – the evidence still suggests benefit. Pharm J 2005;274:618–19. // 6. Oluwole-Ojo S. Advanced Services Medication Use Review. 2005. www.npa.co.uk/publications/nhsdev/PCO-resources/advanced/wandsworth/ADVANCED-SERVICES-WPCT. ppt#256,1,ADVANCED SERVICES (accessed August 29, 2007). // 7. Bellingham C. How to offer a medicines use review. Pharm J 2004;273:602. // 8. Lewis T. Using the NO TEARS tool for medication review. BMJ 2004;329:434. // 9. Alexander A. General tips for successful MURs. 2005. www. psnc.org.uk/uploaded_txt/CPNews%20June05%20.pdf? PHPSESSID= e56df697ce0f89b3a112cba6b80036c (accessed August 29, 2007). // 10. MHRA. Medicines use review: understand your medicines.2006. www.mhra.gov.uk (accessed August 29, 2007).
25	235	Patient education	Process	All	Follow up and continuity	Patient-centered	A - General and unspecified	Patient education	1. Chin WY, Lam CL, Lo SV. Quality of care of nurse-led and allied health personnel-led primary care clinics. Hong Kong medical journal = Xianggang yi xue za zhi. 2011;17(3):217-30. // 2. Knight EL, Avorn J. Quality indicators for appropriate medication use in vulnerable elders. Ann Intern Med 2001;135:703-10. // 3. RESPECT trial team. Effectiveness of shared pharmaceutical care for older patients: RESPECT trial findings. Br J Gen Pract 2010;60:e10-9. // 4. Mackie CA, Lawson DH, Campbell A, Maclaren AG, Waigh R. A randomised controlled trial of medication review in patients receiving polypharmacy in general practice. Pharm J 1999;263:R7 // 5. Holland R, Smith R, Harvey I. Where now for pharmacist led medication review? J Epidemiol Community Health 2006;60:92-3. // 6. Mackie CA, Lawson DH, Campbell A, Maclaren AG, Waigh R. A randomised controlled trial of medication review in patients receiving polypharmacy in general practice. Pharm J 1999;263:R7 .
25	236	Medication list	Process	All	Treatment	Safe	A - General and unspecified	Medication list	1. Chin WY, Lam CL, Lo SV. Quality of care of nurse-led and allied health personnel-led primary care clinics. Hong Kong medical journal = Xianggang yi xue za zhi. 2011;17(3):217-30. // 2. Knight EL, Avorn J. Quality indicators for appropriate medication use in vulnerable elders. Ann Intern Med 2001;135:703-10. // 3. RESPECT trial team. Effectiveness of shared pharmaceutical care for older patients: RESPECT trial findings. Br J Gen Pract 2010;60:e10-9. // 4. Mackie CA, Lawson DH, Campbell A, Maclaren AG, Waigh R. A randomised controlled trial of medication review in patients receiving polypharmacy in general practice. Pharm J 1999;263:R7 // 5. Holland R, Smith R, Harvey I. Where now for pharmacist led medication review? J Epidemiol Community Health 2006;60:92-3. // 6. Mackie CA, Lawson DH, Campbell A, Maclaren AG, Waigh R. A randomised controlled trial of medication review in patients receiving polypharmacy in general practice. Pharm J 1999;263:R7 .
25	237	Response to Therapy	Process	All	Treatment	Effective	A - General and unspecified	Response to Therapy	1. Chin WY, Lam CL, Lo SV. Quality of care of nurse-led and allied health personnel-led primary care clinics. Hong Kong medical journal = Xianggang yi xue za zhi. 2011;17(3):217-30. // 2. Knight EL, Avorn J. Quality indicators for appropriate medication use in vulnerable elders. Ann Intern Med 2001;135:703-10. // 3. RESPECT trial team. Effectiveness of shared pharmaceutical care for older patients: RESPECT trial findings. Br J Gen Pract 2010;60:e10-9. // 4. Mackie CA, Lawson DH, Campbell A, Maclaren AG, Waigh R. A randomised controlled trial of medication review in patients receiving polypharmacy in general practice. Pharm J 1999;263:R7 // 5. Holland R, Smith R, Harvey I. Where now for pharmacist led medication review? J Epidemiol Community Health 2006;60:92-3. // 6. Mackie CA, Lawson DH, Campbell A, Maclaren AG, Waigh R. A randomised controlled trial of medication review in patients receiving polypharmacy in general practice. Pharm J 1999;263:R7 .
25	238	Periodic drug regimen review	Process	Chronic	Follow up and continuity	Safe	A - General and unspecified	Periodic drug regimen review	1. Chin WY, Lam CL, Lo SV. Quality of care of nurse-led and allied health personnel-led primary care clinics. Hong Kong medical journal = Xianggang yi xue za zhi. 2011;17(3):217-30. // 2. Knight EL, Avorn J. Quality indicators for appropriate medication use in vulnerable elders. Ann Intern Med 2001;135:703-10. // 3. RESPECT trial team. Effectiveness of shared pharmaceutical care for older patients: RESPECT trial findings. Br J Gen Pract 2010;60:e10-9. // 4. Mackie CA, Lawson DH, Campbell A, Maclaren AG, Waigh R. A randomised controlled trial of medication review in patients receiving polypharmacy in general practice. Pharm J 1999;263:R7 // 5. Holland R, Smith R, Harvey I. Where now for pharmacist led medication review? J Epidemiol Community Health 2006;60:92-3. // 6. Mackie CA, Lawson DH, Campbell A, Maclaren AG, Waigh R. A randomised controlled trial of medication review in patients receiving polypharmacy in general practice. Pharm J 1999;263:R7 .

25	239	Monitoring warfarin therapy	Process	Chronic	Follow up and continuity	Safe	K - Cardiovascular	Monitoring warfarin therapy	1. Chin WY, Lam CL, Lo SV. Quality of care of nurse-led and allied health personnel-led primary care clinics. Hong Kong medical journal = Xianggang yi xue za zhi. 2011;17(3):217-30. // 2. Knight EL, Avorn J. Quality indicators for appropriate medication use in vulnerable elders. Ann Intern Med 2001;135:703-10. // 3. RESPECT trial team. Effectiveness of shared pharmaceutical care for older patients: RESPECT trial findings. Br J Gen Pract 2010;60:e10-9. // 4. Mackie CA, Lawson DH, Campbell A, Maclaren AG, Waigh R. A randomised controlled trial of medication review in patients receiving polypharmacy in general practice. Pharm J 1999;263:R7 // 5. Holland R, Smith R, Harvey I. Where now for pharmacist led medication review? J Epidemiol Community Health 2006;60:92-3. // 6. Mackie CA, Lawson DH, Campbell A, Maclaren AG, Waigh R. A randomised controlled trial of medication review in patients receiving polypharmacy in general practice. Pharm J 1999;263:R7 .
25	240	Monitoring diuretic therapy	Process	Chronic	Follow up and continuity	Safe	K - Cardiovascular	Monitoring diuretic therapy	1. Chin WY, Lam CL, Lo SV. Quality of care of nurse-led and allied health personnel-led primary care clinics. Hong Kong medical journal = Xianggang yi xue za zhi. 2011;17(3):217-30. // 2. Knight EL, Avorn J. Quality indicators for appropriate medication use in vulnerable elders. Ann Intern Med 2001;135:703-10. // 3. RESPECT trial team. Effectiveness of shared pharmaceutical care for older patients: RESPECT trial findings. Br J Gen Pract 2010;60:e10-9. // 4. Mackie CA, Lawson DH, Campbell A, Maclaren AG, Waigh R. A randomised controlled trial of medication review in patients receiving polypharmacy in general practice. Pharm J 1999;263:R7 // 5. Holland R, Smith R, Harvey I. Where now for pharmacist led medication review? J Epidemiol Community Health 2006;60:92-3. // 6. Mackie CA, Lawson DH, Campbell A, Maclaren AG, Waigh R. A randomised controlled trial of medication review in patients receiving polypharmacy in general practice. Pharm J 1999;263:R7 .
25	241	Avoid use of chlorpropamide as a hypoglycaemic	Process	Chronic	Treatment	Safe	T - Endocrine/Metabolic and Nutritional	Avoid use of chlorpropamide as a hypoglycaemic	1. Chin WY, Lam CL, Lo SV. Quality of care of nurse-led and allied health personnel-led primary care clinics. Hong Kong medical journal = Xianggang yi xue za zhi. 2011;17(3):217-30. // 2. Knight EL, Avorn J. Quality indicators for appropriate medication use in vulnerable elders. Ann Intern Med 2001;135:703-10. // 3. RESPECT trial team. Effectiveness of shared pharmaceutical care for older patients: RESPECT trial findings. Br J Gen Pract 2010;60:e10-9. // 4. Mackie CA, Lawson DH, Campbell A, Maclaren AG, Waigh R. A randomised controlled trial of medication review in patients receiving polypharmacy in general practice. Pharm J 1999;263:R7 // 5. Holland R, Smith R, Harvey I. Where now for pharmacist led medication review? J Epidemiol Community Health 2006;60:92-3. // 6. Mackie CA, Lawson DH, Campbell A, Maclaren AG, Waigh R. A randomised controlled trial of medication review in patients receiving polypharmacy in general practice. Pharm J 1999;263:R7 .
25	242	Avoid drugs with strong anticholinergic properties whenever possible	Process	Chronic	Treatment	Safe	A - General and unspecified	Avoid drugs with strong anticholinergic properties whenever possible	1. Chin WY, Lam CL, Lo SV. Quality of care of nurse-led and allied health personnel-led primary care clinics. Hong Kong medical journal = Xianggang yi xue za zhi. 2011;17(3):217-30. // 2. Knight EL, Avorn J. Quality indicators for appropriate medication use in vulnerable elders. Ann Intern Med 2001;135:703-10. // 3. RESPECT trial team. Effectiveness of shared pharmaceutical care for older patients: RESPECT trial findings. Br J Gen Pract 2010;60:e10-9. // 4. Mackie CA, Lawson DH, Campbell A, Maclaren AG, Waigh R. A randomised controlled trial of medication review in patients receiving polypharmacy in general practice. Pharm J 1999;263:R7 // 5. Holland R, Smith R, Harvey I. Where now for pharmacist led medication review? J Epidemiol Community Health 2006;60:92-3. // 6. Mackie CA, Lawson DH, Campbell A, Maclaren AG, Waigh R. A randomised controlled trial of medication review in patients receiving polypharmacy in general practice. Pharm J 1999;263:R7 .
25	243	Avoid barbiturates	Process	Chronic	Treatment	Safe	P - Psychological	Avoid barbiturates	1. Chin WY, Lam CL, Lo SV. Quality of care of nurse-led and allied health personnel-led primary care clinics. Hong Kong medical journal = Xianggang yi xue za zhi. 2011;17(3):217-30. // 2. Knight EL, Avorn J. Quality indicators for appropriate medication use in vulnerable elders. Ann Intern Med 2001;135:703-10. // 3. RESPECT trial team. Effectiveness of shared pharmaceutical care for older patients: RESPECT trial findings. Br J Gen Pract 2010;60:e10-9. // 4. Mackie CA, Lawson DH, Campbell A, Maclaren AG, Waigh R. A randomised controlled trial of medication review in patients receiving polypharmacy in general practice. Pharm J 1999;263:R7 // 5. Holland R, Smith R, Harvey I. Where now for pharmacist led medication review? J Epidemiol Community Health 2006;60:92-3. // 6. Mackie CA, Lawson DH, Campbell A, Maclaren AG, Waigh R. A randomised controlled trial of medication review in patients receiving polypharmacy in general practice. Pharm J 1999;263:R7 .
25	244	Avoid meperidine as an opioid analgesic	Process	Chronic	Treatment	Safe	P - Psychological	Avoid meperidine as na opioid analgesic	1. Chin WY, Lam CL, Lo SV. Quality of care of nurse-led and allied health personnel-led primary care clinics. Hong Kong medical journal = Xianggang yi xue za zhi. 2011;17(3):217-30. // 2. Knight EL, Avorn J. Quality indicators for appropriate medication use in vulnerable elders. Ann Intern Med 2001;135:703-10. // 3. RESPECT trial team. Effectiveness of shared pharmaceutical care for older patients: RESPECT trial findings. Br J Gen Pract 2010;60:e10-9. // 4. Mackie CA, Lawson DH, Campbell A, Maclaren AG, Waigh R. A randomised controlled trial of medication review in patients receiving polypharmacy in general practice. Pharm J 1999;263:R7 // 5. Holland R, Smith R, Harvey I. Where now for pharmacist led medication review? J Epidemiol Community Health 2006;60:92-3. // 6. Mackie CA, Lawson DH, Campbell A, Maclaren AG, Waigh R. A randomised controlled trial of medication review in patients receiving polypharmacy in general practice. Pharm J 1999;263:R7 .
25	245	Monitoring renal function and potassium in patients prescribed ACE inhibitors	Process	Chronic	Follow up and continuity	Safe	U - Urological	Monitoring renal function and potassium in patients prescribed ACE inhibitors	1. Chin WY, Lam CL, Lo SV. Quality of care of nurse-led and allied health personnel-led primary care clinics. Hong Kong medical journal = Xianggang yi xue za zhi. 2011;17(3):217-30. // 2. Knight EL, Avorn J. Quality indicators for appropriate medication use in vulnerable elders. Ann Intern Med 2001;135:703-10. // 3. RESPECT trial team. Effectiveness of shared pharmaceutical care for older patients: RESPECT trial findings. Br J Gen Pract 2010;60:e10-9. // 4. Mackie CA, Lawson DH, Campbell A, Maclaren AG, Waigh R. A randomised controlled trial of medication review in patients receiving polypharmacy in general practice. Pharm J 1999;263:R7 // 5. Holland R, Smith R, Harvey I. Where now for pharmacist led medication review? J Epidemiol Community Health 2006;60:92-3. // 6. Mackie CA, Lawson DH, Campbell A, Maclaren AG, Waigh R. A randomised controlled trial of medication review in patients receiving polypharmacy in general practice. Pharm J 1999;263:R7 .
11	246	Absenteeism from Work/School for Asthma	Outcome	Chronic	Treatment	Patient-centered	R - Respiratory	Absenteeism from Work/School for Asthma	To, T., Guttmann, A., Lougheed, M. D., Gershon, A. S., Dell, S. D., Stanbrook, M. B., … Fisman, D. N. (2010). Evidence-based performance indicators of primary care for asthma: a modified RAND Appropriateness Method. International Journal for Quality in Health Care, 22(6), 476–485.
16	247	Potentially avoidable hospitalizations in patients with chronic conditions	Outcome	Chronic	Treatment	Effective	A - General and unspecified	Potentially avoidable hospitalizations in patients with chronic conditions	Olry de Labry Lima, A., García Mochón, L., & Bermúdez Tamayo, C. (2017). Identificación de indicadores de resultado en salud en atención primaria. Una revisión de revisiones sistemáticas. Revista de Calidad Asistencial, 32(5), 278–288.
3	248	Governance: (De)centralization of primary care management and service development	Structure	All	All	Effective	Not Defined	This is shaped by the level (national, regional, local) at which primary care policies are determined, the degree in which standards allow for variation in primary care practices geographically, and the development of policies on community participation in primary care management and priority setting.	Kringos DS, Boerma WG, Hutchinson A, van der Zee 465 J, Groenewegen PP. The breadth of primary care: a systematic literature review of its core dimensions. 467 BMC Health Serv Res. 2010;10:65. Published 2010 Mar 13. doi:10.1186/1472-468 6963-10-65
3	249	Academic status of the primary care discipline	Structure	All	All	Effective	Not Defined	Reflected by academic departments of family medicine/ primary care within universities	Kringos DS, Boerma WG, Hutchinson A, van der Zee 465 J, Groenewegen PP. The breadth of primary care: a systematic literature review of its core dimensions. 467 BMC Health Serv Res. 2010;10:65. Published 2010 Mar 13. doi:10.1186/1472-468 6963-10-66
3	250	Acceptability of primary care services	Process	All	All	Patient-centered	Not Defined	Patient satisfaction with the organization of primary care	Kringos DS, Boerma WG, Hutchinson A, van der Zee 465 J, Groenewegen PP. The breadth of primary care: a systematic literature review of its core dimensions. 467 BMC Health Serv Res. 2010;10:65. Published 2010 Mar 13. doi:10.1186/1472-468 6963-10-67
3	251	Accommodation of accessibility	Process	All	All	Patient-centered	Not Defined	The manner in which resources are organized to accommodate access (e.g. appointment system, after-hours care arrangements, home visits)	Kringos DS, Boerma WG, Hutchinson A, van der Zee 465 J, Groenewegen PP. The breadth of primary care: a systematic literature review of its core dimensions. 467 BMC Health Serv Res. 2010;10:65. Published 2010 Mar 13. doi:10.1186/1472-468 6963-10-68
3	252	Affordability of primary care services	Process	All	All	Effective	Not Defined	Financial barriers patients experience to receive primary care services, such as co-payments and cost-sharing arrangements	Kringos DS, Boerma WG, Hutchinson A, van der Zee 465 J, Groenewegen PP. The breadth of primary care: a systematic literature review of its core dimensions. 467 BMC Health Serv Res. 2010;10:65. Published 2010 Mar 13. doi:10.1186/1472-468 6963-10-69
3	253	Allocative and productive efficiency	Structure	All	All	Efficient	Not Defined	Respectively, minimizing patient's opportunity cost of time spent in treatment; maximizing the patient's outcome, minimizing the cost per patient	Kringos DS, Boerma WG, Hutchinson A, van der Zee 465 J, Groenewegen PP. The breadth of primary care: a systematic literature review of its core dimensions. 467 BMC Health Serv Res. 2010;10:65. Published 2010 Mar 13. doi:10.1186/1472-468 6963-10-70
3	254	Appropriate technology in primary care	Structure	All	All	Effective	Not Defined	Appropriate technology in primary care	Kringos DS, Boerma WG, Hutchinson A, van der Zee 465 J, Groenewegen PP. The breadth of primary care: a systematic literature review of its core dimensions. 467 BMC Health Serv Res. 2010;10:65. Published 2010 Mar 13. doi:10.1186/1472-468 6963-10-71
3	255	Availability of primary care services	Structure	All	All	Effective	Not Defined	Availability of primary care services	Kringos DS, Boerma WG, Hutchinson A, van der Zee 465 J, Groenewegen PP. The breadth of primary care: a systematic literature review of its core dimensions. 467 BMC Health Serv Res. 2010;10:65. Published 2010 Mar 13. doi:10.1186/1472-468 6963-10-72
3	256	Development of the primary care workforce	Structure	All	All	All	Not Defined	Development of the primary care workforce	Kringos DS, Boerma WG, Hutchinson A, van der Zee 465 J, Groenewegen PP. The breadth of primary care: a systematic literature review of its core dimensions. 467 BMC Health Serv Res. 2010;10:65. Published 2010 Mar 13. doi:10.1186/1472-468 6963-10-73
3	257	Education and retention	Structure	All	All	All	Not Defined	Vocational training requirements for primary care professionals, primary care workforce supply and retention problems, and capacity planning	Kringos DS, Boerma WG, Hutchinson A, van der Zee 465 J, Groenewegen PP. The breadth of primary care: a systematic literature review of its core dimensions. 467 BMC Health Serv Res. 2010;10:65. Published 2010 Mar 13. doi:10.1186/1472-468 6963-10-74
3	258	Efficiency in performance of primary care workforce	Structure	All	All	Efficient	Not Defined	Reflected by basic figures relating to the provision of care, such as number of consultations and their duration, frequency of prescription medicines (unnecessary use), and the number of new referrals to medical specialists	Kringos DS, Boerma WG, Hutchinson A, van der Zee 465 J, Groenewegen PP. The breadth of primary care: a systematic literature review of its core dimensions. 467 BMC Health Serv Res. 2010;10:65. Published 2010 Mar 13. doi:10.1186/1472-468 6963-10-75
3	259	Employment status of primary care workforce	Structure	All	All	All	Not Defined	Employment status of primary care workforce	Kringos DS, Boerma WG, Hutchinson A, van der Zee 465 J, Groenewegen PP. The breadth of primary care: a systematic literature review of its core dimensions. 467 BMC Health Serv Res. 2010;10:65. Published 2010 Mar 13. doi:10.1186/1472-468 6963-10-76
3	260	Equality in access	Process	All	All	Equitable	Not Defined	The extent to which access to primary care services is provided on the basis of health needs, without systematic differences on the basis of individual or social characteristics	Kringos DS, Boerma WG, Hutchinson A, van der Zee 465 J, Groenewegen PP. The breadth of primary care: a systematic literature review of its core dimensions. 467 BMC Health Serv Res. 2010;10:65. Published 2010 Mar 13. doi:10.1186/1472-468 6963-10-77
3	261	First contact for common health problems	Process	All	All	All	Not Defined	First contact for common health problems	Kringos DS, Boerma WG, Hutchinson A, van der Zee 465 J, Groenewegen PP. The breadth of primary care: a systematic literature review of its core dimensions. 467 BMC Health Serv Res. 2010;10:65. Published 2010 Mar 13. doi:10.1186/1472-468 6963-10-78
3	262	Future development of the primary care workforce	Structure	All	All	All	Not Defined	Hampering threats to the current development and expected trends in the future development of the primary care workforce, from the point of view of stakeholders	Kringos DS, Boerma WG, Hutchinson A, van der Zee 465 J, Groenewegen PP. The breadth of primary care: a systematic literature review of its core dimensions. 467 BMC Health Serv Res. 2010;10:65. Published 2010 Mar 13. doi:10.1186/1472-468 6963-10-79
3	263	Gatekeeping system	Process	All	All	Efficient	Not Defined	Presence of a gatekeeping system with a figure of a family medical doctor	Kringos DS, Boerma WG, Hutchinson A, van der Zee 465 J, Groenewegen PP. The breadth of primary care: a systematic literature review of its core dimensions. 467 BMC Health Serv Res. 2010;10:65. Published 2010 Mar 13. doi:10.1186/1472-468 6963-10-80
3	264	Geographic accessibility of primary care services	Process	All	All	Equitable	Not Defined	Geographic accessibility of primary care services	Kringos DS, Boerma WG, Hutchinson A, van der Zee 465 J, Groenewegen PP. The breadth of primary care: a systematic literature review of its core dimensions. 467 BMC Health Serv Res. 2010;10:65. Published 2010 Mar 13. doi:10.1186/1472-468 6963-10-81
3	265	Governance: Health (care) system goals	Structure	All	All	All	Not Defined	The vision and direction of a primary care system depend on explicit health or health care goals at national level	Kringos DS, Boerma WG, Hutchinson A, van der Zee 465 J, Groenewegen PP. The breadth of primary care: a systematic literature review of its core dimensions. 467 BMC Health Serv Res. 2010;10:65. Published 2010 Mar 13. doi:10.1186/1472-468 6963-10-82
3	266	Income of primary care workforce	Structure	All	All	All	Not Defined	Income of primary care workforce in a period	Kringos DS, Boerma WG, Hutchinson A, van der Zee 465 J, Groenewegen PP. The breadth of primary care: a systematic literature review of its core dimensions. 467 BMC Health Serv Res. 2010;10:65. Published 2010 Mar 13. doi:10.1186/1472-468 6963-10-84
3	267	Informational continuity of care	Process	All	Follow up and continuity	All	Not Defined	Informational continuity of care	Kringos DS, Boerma WG, Hutchinson A, van der Zee 465 J, Groenewegen PP. The breadth of primary care: a systematic literature review of its core dimensions. 467 BMC Health Serv Res. 2010;10:65. Published 2010 Mar 13. doi:10.1186/1472-468 6963-10-85
3	268	Integration of primary care in the health care system	Structure	All	Follow up and continuity	Effective	Not Defined	Integration of primary care in the health care system	Kringos DS, Boerma WG, Hutchinson A, van der Zee 465 J, Groenewegen PP. The breadth of primary care: a systematic literature review of its core dimensions. 467 BMC Health Serv Res. 2010;10:65. Published 2010 Mar 13. doi:10.1186/1472-468 6963-10-49
3	269	Integration of primary care-secondary care	Process	All	Follow up and continuity	Effective	Not Defined	Integration of primary care-secondary care	Kringos DS, Boerma WG, Hutchinson A, van der Zee 465 J, Groenewegen PP. The breadth of primary care: a systematic literature review of its core dimensions. 467 BMC Health Serv Res. 2010;10:65. Published 2010 Mar 13. doi:10.1186/1472-468 6963-10-50
3	270	Medical equipment available	Structure	All	All	Effective	Not Defined	Medical equipment available	Kringos DS, Boerma WG, Hutchinson A, van der Zee 465 J, Groenewegen PP. The breadth of primary care: a systematic literature review of its core dimensions. 467 BMC Health Serv Res. 2010;10:65. Published 2010 Mar 13. doi:10.1186/1472-468 6963-10-51
3	271	Ownership status of primary care practices	Structure	All	All	All	Not Defined	Ownership status of primary care practices	Kringos DS, Boerma WG, Hutchinson A, van der Zee 465 J, Groenewegen PP. The breadth of primary care: a systematic literature review of its core dimensions. 467 BMC Health Serv Res. 2010;10:65. Published 2010 Mar 13. doi:10.1186/1472-468 6963-10-52
3	272	Patient advocacy	Process	All	All	Patient-centered	Not Defined	Patient advocacy	Kringos DS, Boerma WG, Hutchinson A, van der Zee 465 J, Groenewegen PP. The breadth of primary care: a systematic literature review of its core dimensions. 467 BMC Health Serv Res. 2010;10:65. Published 2010 Mar 13. doi:10.1186/1472-468 6963-10-53
3	273	Governance: Policy on equity in access to primary care services	Process	All	All	Equitable	Not Defined	Equity in access can be influenced by policy development and regulation on the distribution of human resources and quality of care across geographical areas, by setting policy objectives regarding the duration of waiting time for (specific) primary care services; and by assuring universal financial coverage for primary care services by a publicly accountable body	Kringos DS, Boerma WG, Hutchinson A, van der Zee 465 J, Groenewegen PP. The breadth of primary care: a systematic literature review of its core dimensions. 467 BMC Health Serv Res. 2010;10:65. Published 2010 Mar 13. doi:10.1186/1472-468 6963-10-54
3	274	Primary care expenditures	Structure	All	All	Efficient	Not Defined	Primary care expenditures	Kringos DS, Boerma WG, Hutchinson A, van der Zee 465 J, Groenewegen PP. The breadth of primary care: a systematic literature review of its core dimensions. 467 BMC Health Serv Res. 2010;10:65. Published 2010 Mar 13. doi:10.1186/1472-468 6963-10-55
3	275	Primary care practice and team structure	Structure	All	All	Effective	Not Defined	Primary care practice and team structure	Kringos DS, Boerma WG, Hutchinson A, van der Zee 465 J, Groenewegen PP. The breadth of primary care: a systematic literature review of its core dimensions. 467 BMC Health Serv Res. 2010;10:65. Published 2010 Mar 13. doi:10.1186/1472-468 6963-10-56
3	276	Professional associations	Structure	All	All	Effective	Not Defined	The organization of professional associations for the primary care workforce	Kringos DS, Boerma WG, Hutchinson A, van der Zee 465 J, Groenewegen PP. The breadth of primary care: a systematic literature review of its core dimensions. 467 BMC Health Serv Res. 2010;10:65. Published 2010 Mar 13. doi:10.1186/1472-468 6963-10-57
3	277	Profile of primary care workforce	Structure	All	All	All	Not Defined	The type of health care professionals that are considered to be part of the primary care workforce, and their gender balance	Kringos DS, Boerma WG, Hutchinson A, van der Zee 465 J, Groenewegen PP. The breadth of primary care: a systematic literature review of its core dimensions. 467 BMC Health Serv Res. 2010;10:65. Published 2010 Mar 13. doi:10.1186/1472-468 6963-10-58
3	278	Quality management infrastructure in primary care	Process	All	All	Effective	Not Defined	Quality management infrastructure in primary care	Kringos DS, Boerma WG, Hutchinson A, van der Zee 465 J, Groenewegen PP. The breadth of primary care: a systematic literature review of its core dimensions. 467 BMC Health Serv Res. 2010;10:65. Published 2010 Mar 13. doi:10.1186/1472-468 6963-10-59
3	279	Recognition and responsibilities	Process	All	All	All	Not Defined	Whether the primary care discipline is officially recognized as a separate discipline among the medical disciplines, with recognised responsibilities	Kringos DS, Boerma WG, Hutchinson A, van der Zee 465 J, Groenewegen PP. The breadth of primary care: a systematic literature review of its core dimensions. 467 BMC Health Serv Res. 2010;10:65. Published 2010 Mar 13. doi:10.1186/1472-468 6963-10-60
3	280	Remuneration system of primary care workforce	Structure	All	All	All	Not Defined	Remuneration system of primary care workforce	Kringos DS, Boerma WG, Hutchinson A, van der Zee 465 J, Groenewegen PP. The breadth of primary care: a systematic literature review of its core dimensions. 467 BMC Health Serv Res. 2010;10:65. Published 2010 Mar 13. doi:10.1186/1472-468 6963-10-61
3	281	Skill-mix of primary care providers	Structure	All	All	Effective	Not Defined	Skill-mix of primary care providers	Kringos DS, Boerma WG, Hutchinson A, van der Zee 465 J, Groenewegen PP. The breadth of primary care: a systematic literature review of its core dimensions. 467 BMC Health Serv Res. 2010;10:65. Published 2010 Mar 13. doi:10.1186/1472-468 6963-10-62
3	282	The method of financing health care for the majority of the population	Structure	All	All	All	Not Defined	The method of financing health care for the majority of the population	Kringos DS, Boerma WG, Hutchinson A, van der Zee 465 J, Groenewegen PP. The breadth of primary care: a systematic literature review of its core dimensions. 467 BMC Health Serv Res. 2010;10:65. Published 2010 Mar 13. doi:10.1186/1472-468 6963-10-63
3	283	Technical efficiency	Structure	All	All	Efficient	Not Defined	A system is technical efficient if it cannot reduce its resource use without reducing its ability to treat patients or to reach certain outcomes	Kringos DS, Boerma WG, Hutchinson A, van der Zee 465 J, Groenewegen PP. The breadth of primary care: a systematic literature review of its core dimensions. 467 BMC Health Serv Res. 2010;10:65. Published 2010 Mar 13. doi:10.1186/1472-468 6963-10-64
3	284	Utilisation of primary care services	Process	All	All	Efficient	Not Defined	Actual consumption of primary care services	Kringos DS, Boerma WG, Hutchinson A, van der Zee 465 J, Groenewegen PP. The breadth of primary care: a systematic literature review of its core dimensions. 467 BMC Health Serv Res. 2010;10:65. Published 2010 Mar 13. doi:10.1186/1472-468 6963-10-65
5	285	Patient satisfaction	Outcome	All	All	Patient-centered	Not Defined	Patient satisfaction	Batbaatar, E., Dorjdagva, J., Luvsannyam, A., Savino, M. M., & Amenta, P. (2016). Determinants of patient satisfaction: a systematic review. Perspectives in Public Health, 137(2), 89–101.
6	286	Proportion of patients that is satisfied with the quality of contact with his care giver(s).	Outcome	All	All	Patient-centered	Not Defined	Proportion of patients that is satisfied with the quality of contact with his care giver(s).	Bekkering, G. E., Zeeuws, D., Lenaerts, E., Pas, L., Verstuyf, G., Matthys, F., … Matheï, C. (2016). Development and Validation of Quality Indicators on Continuing Care for Patients With AUD: A Delphi Study. Alcohol and Alcoholism, 51(5), 555–561.
12	287	Costs and cost effectiveness	Structure	All	All	Efficient	Not Defined	Costs and cost effectiveness	Flodgren, G., Gonçalves-Bradley, D. C., & Pomey, M.-P. (2016). External inspection of compliance with standards for improved healthcare outcomes. Cochrane Database of Systematic Reviews.
4	288	Annual review	Process	Chronic	Follow up and continuity	Effective	P - Psychological	Patients who do not attend the practice for their annual review who are identified and followed up by the practice team	1. Agency for Healthcare Research and Quality. AHRQ — quality indicators. AHRQ, 2016. www.qualityindicators.ahrq.gov (accessed 23 May 2017). || 2. Kronenberg C, Doran T, Goddard M, Kendrick T, Gilbody S, Dare CR, et al. Identifying primary care quality indicators for people with serious mental illness: a systematic review. The British journal of general practice : the journal of the Royal College of General Practitioners. 2017;67(661):e519-e30
4	289	System contact	Process	Chronic	Follow up and continuity	Effective	P - Psychological	System contact: number of patients in contact with the treatment system	1. Parameswaran SG, Spaeth-Rublee B, Pincus HA. Measuring the quality of mental health care: consensus perspectives from selected industrialized countries. Adm Policy Ment Health 2015; 42(3): 288–295. || 2. Kronenberg C, Doran T, Goddard M, Kendrick T, Gilbody S, Dare CR, et al. Identifying primary care quality indicators for people with serious mental illness: a systematic review. The British journal of general practice : the journal of the Royal College of General Practitioners. 2017;67(661):e519-e30.
4	290	Therapeutic Plan and patient communications	Process	Chronic	Treatment	Patient-Centered	P - Psychological	Patients with all current medication clearly available at all consultations — known drug dosages, frequencies, history of side effects, review date	1. Kronenberg C, Doran T, Goddard M, Kendrick T, Gilbody S, Dare CR, et al. Identifying primary care quality indicators for people with serious mental illness: a systematic review. The British journal of general practice : the journal of the Royal College of General Practitioners. 2017;67(661):e519-e30. || 2. Carlborg A, Ferntoft L, Thuresson M, Bodegard J. Population study of disease burden, management, and treatment of bipolar disorder in Sweden: a retrospective observational registry study. Bipolar disorders. 2015;17(1):76-85.
4	291	Weight gain after use of medications	Outcome	Chronic	Follow up and continuity	Safe	P - Psychological	Number of patients with weight gain and use of concomitant medication	1. Haro JM, Salvador-Carulla L. The SOHO (Schizophrenia Outpatient Health Outcome) Study: implications for the treatment of schizophrenia. CNS Drugs 2006; 20(4): 293–301. || 2. Kronenberg C, Doran T, Goddard M, Kendrick T, Gilbody S, Dare CR, et al. Identifying primary care quality indicators for people with serious mental illness: a systematic review. The British journal of general practice : the journal of the Royal College of General Practitioners. 2017;67(661):e519-e30.

4	292	Plasmatic monitoring for the use of lithium	Process	Chronic	Follow up and continuity	Safe	P - Psychological	Number of patients in use of lithium and with plasma lithium levels monitored regularly	1. Kronenberg C, Doran T, Goddard M, Kendrick T, Gilbody S, Dare CR, et al. Identifying primary care quality indicators for people with serious mental illness: a systematic review. The British journal of general practice : the journal of the Royal College of General Practitioners. 2017;67(661):e519-e30. || 2. Zaworski J, Delannoy PY, Boussekey N, Thellier D, Georges H, Leroy O. Lithium: one drug, five complications. J Intensive Care. 2017;5:70. Published 2017 Dec 20. doi:10.1186/s40560-017-0257-5 || 3. Rej S, Herrmann N, Gruneir A, Jandoc R, McArthur E, Dixon S, et al. Blood Lithium Monitoring Practices in a Population-Based Sample of Older Adults. The Journal of clinical psychiatry. 2018;79(6).
4	293	Antidepressants and anxiolytics prescription for Bipolar disorder	Process	Chronic	Treatment	Effective	P - Psychological	Percentages of bipolar service users prescribed antidepressants and anxiolytics	1. Kronenberg C, Doran T, Goddard M, Kendrick T, Gilbody S, Dare CR, et al. Identifying primary care quality indicators for people with serious mental illness: a systematic review. The British journal of general practice : the journal of the Royal College of General Practitioners. 2017;67(661):e519-e30. || 2. National Institute for Health and Care Excellence. Bipolar disorder: assessment and management. CG185. London: NICE, 2014. https://www.nice.org.uk/guidance/cg185 (accessed 23 May 2017). || 3. Caughey G, Kalisch Ellett L, Wong T. Development of evidence-based Australian medication-related indicators of potentially preventable hospitalisations: a modified RAND appropriateness method. BMJ Open 2014; 4(4): e004625. || 4. Bjørklund, L., Horsdal, H. T., Mors, O., Østergaard, S. D., & Gasse, C. (2015). Trends in the psychopharmacological treatment of bipolar disorder: a nationwide register-based study. Acta Neuropsychiatrica, 28(02), 75–84.
4	294	Screening tests for Depot antipsychotics	Process	Chronic	Screening and prevention	Effective	P - Psychological	Proportion of patients who are receiving depot antipsychotics who have appropriate laboratory screening tests	1. Kronenberg C, Doran T, Goddard M, Kendrick T, Gilbody S, Dare CR, et al. Identifying primary care quality indicators for people with serious mental illness: a systematic review. The British journal of general practice : the journal of the Royal College of General Practitioners. 2017;67(661):e519-e30. || 2. Agency for Healthcare Research and Quality. AHRQ — quality indicators. AHRQ, 2016. www.qualityindicators.ahrq.gov (accessed 23 May 2017).
4	295	Antipsychotic medication review	Process	Chronic	Treatment	Effective	P - Psychological	Patients have their antipsychotic medication reviewed regularly, considering symptoms and side effects: appropriate referral to specialist	1. Kronenberg C, Doran T, Goddard M, Kendrick T, Gilbody S, Dare CR, et al. Identifying primary care quality indicators for people with serious mental illness: a systematic review. The British journal of general practice : the journal of the Royal College of General Practitioners. 2017;67(661):e519-e30.
4	296	Polyfarmacy	Process	Chronic	Treatment	Safe	P - Psychological	Number of patients using more than four psychotropic drugs at the same time	1. Kronenberg C, Doran T, Goddard M, Kendrick T, Gilbody S, Dare CR, et al. Identifying primary care quality indicators for people with serious mental illness: a systematic review. The British journal of general practice : the journal of the Royal College of General Practitioners. 2017;67(661):e519-e30. || 2. Fornaro M, De Berardis D, Koshy AS, et al. Prevalence and clinical features associated with bipolar disorder polypharmacy: a systematic review. Neuropsychiatr Dis Treat. 2016;12:719-35. Published 2016 Mar 31. doi:10.2147/NDT.S100846
5	297	Promptness of antidepressant treatment follow-up	Process	Chronic	Follow up and continuity	Timely	P - Psychological	Adequate promptness of antidepressant treatment follow-up	1. Duhoux A, Fournier L, Menear M. Quality Indicators for Depression Treatment in Primary Care: A Systematic Literature Review. Current Psychiatry Reviews (2011) 7: 104. https://doi.org/10.2174/157340011796391166
6	298	Falls	Outcome	Chronic	Screening and prevention	Effective	P - Psychological	Vulnerable elders that should have documentation that they were asked at least annually about the occurrence of recent falls	1. Fujita K., Moles RJ, Chen TF. Quality indicators for responsible use of medicines: a systematic review BMJ Open 2018;8:e020437. doi: 10.1136/bmjopen-2017-020437 || 2. Kröger E, Tourigny A, Morin D, et al. Selecting process quality indicators for the integrated care of vulnerable older adults affected by cognitive impairment or dementia. BMC Health Serv Res 2007;7:195. || 3. Chin WY, Lam CL, Lo SV. Quality of care of nurse-led and allied health personnel-led primary care clinics. Hong Kong medical journal = Xianggang yi xue za zhi. 2011;17(3):217-30.
1 [24]	299	Antidepressant medication management: effective continuation phase treatment	Process	Chronic	Treatment	Effective	P - Psychological	Percentage of members 18 years of age and older as of April 30 of the measurement year who were diagnosed with a new episode of depression, were treated with antidepressant medication, and who remained on an antidepressant drug for at least 180 days (6 months).	Friedberg MW, Coltin KL, Pearson SD, Kleinman KP, Zheng J, Singer JA, et al: Does affiliation of physician groups with one another produce higher quality primary care? J Gen Intern Med 2007, 22:1385-1392. // Kringos DS, Boerma WG, Hutchinson A, van der Zee 465 J, Groenewegen PP. // The breadth of primary care: a systematic literature review of its core dimensions. BMC Health Serv Res. 2010;10:65. Published 2010 Mar 13. doi:10.1186/1472-468 6963-10-65
12	300	MRSA (methicillin-resistant Staphylococcus aureus) infection rates	Outcome	Chronic	Diagnosis	Safe	R - Respiratory	Percentage of MRSA (methicillin-resistant Staphylococcus aureus) infection	1. OPM evaluation team. Evaluation of the Healthcare Commission's Healthcare Associated Infections Inspection Programme. OPM Report 2009:1–23. [2881011]
26	301	Bianual assessment of the location of symptoms and/or the presence or absence of limitations in daily activities	Outcome	Chronic	Diagnosis	Effective	L - Musculoskeletal	Providers caring for patients with symptoms of osteoarthritis should document all of the following at least once in 2 years: the location of symptoms and/or the presence or absence of limitations in daily activities.	1. Asch SM, McGlynn EA, Hogan MM, Hayward RA, Shekelle P, Rubenstein L, et al. Comparison of quality of care for patients in the Veterans Health Administration and patients in a national sample. Annals of internal medicine. 2004;141(12):938-45. // 2. McGlynn EA, Asch SM, Adams J, Keesey J, Hicks J, DeCristofaro A, et al. The quality of health care delivered to adults in the United States. The New England journal of medicine.2003;348(26):2635-45.
26	302	Acetaminophen trial for patients with new diagnoses who need pharmacotherapy	Process	Chronic	Treatment	Effective	L - Musculoskeletal	Patients with a new diagnosis of osteoarthritis who wish to take medication for joint symptoms should be offered a trial of acetaminophen.	1. Asch SM, McGlynn EA, Hogan MM, Hayward RA, Shekelle P, Rubenstein L, et al. Comparison of quality of care for patients in the Veterans Health Administration and patients in a national sample. Annals of internal medicine. 2004;141(12):938-45. 2. McGlynn EA, Asch SM, Adams J, Keesey J, Hicks J, DeCristofaro A, et al. The quality of health care delivered to adults in the United States. The New England journal of medicine.2003;348(26):2635-45.
26	303	Recomendation of exercise programs	Process	Chronic	Treatment	Effective	L - Musculoskeletal	Providers caring for patients with symptoms of hip or knee osteoarthritis should recommend exercise programs at least once in 2 years	1. Asch SM, McGlynn EA, Hogan MM, Hayward RA, Shekelle P, Rubenstein L, et al. Comparison of quality of care for patients in the Veterans Health Administration and patients in a national sample. Annals of internal medicine. 2004;141(12):938-45. 2. McGlynn EA, Asch SM, Adams J, Keesey J, Hicks J, DeCristofaro A, et al. The quality of health care delivered to adults in the United States. The New England journal of medicine.2003;348(26):2635-45.
26	304	Annual assessment for vulnerable elders diagnosed and with symptoms of osteoarthritis	Outcome	Chronic	Screening and prevention	Effective	L - Musculoskeletal	IF a vulnerable elder is diagnosed with symptomatic osteoarthritis, THEN functional status and degree of pain should be assessed annually.	1. Ganz DA, Chang JT, Roth CP, Guan M, Kamberg CJ, Niu F, et al. Quality of osteoarthritis care for community-dwelling older adults. Arthritis and rheumatism. 2006;55(2):241-7. // 2. Wenger NS, Solomon DH, Roth CP, MacLean CH, Saliba D, Kamberg CJ, et al. The quality of medical care provided to vulnerable community-dwelling older patients. Annals of internal medicine. 2003;139(9):740-7.
26	305	Exercise prescription for vulnerable elders diagnosed and with symptomatic more than 3 months ago	Process	Chronic	Treatment	Effective	L - Musculoskeletal	IF an ambulatory vulnerable elder receives a new diagnosis of symptomatic osteoarthritis of the knee and has no contraindication to exercise, and is physically and mentally able to exercise, THEN a directed or supervised strengthening or aerobic exercise program should be prescribed within 3 months of diagnosis	1. Ganz DA, Chang JT, Roth CP, Guan M, Kamberg CJ, Niu F, et al. Quality of osteoarthritis care for community-dwelling older adults. Arthritis and rheumatism. 2006;55(2):241-7. // 2. Wenger NS, Solomon DH, Roth CP, MacLean CH, Saliba D, Kamberg CJ, et al. The quality of medical care provided to vulnerable community-dwelling older patients. Annals of internal medicine. 2003;139(9):740-7.
26	306	Counseling/Education about natural history , treatment and management for person age 75 or older diagnosed more than 6 months ago	Process	Chronic	Follow up and continuity	Effective	L - Musculoskeletal	IF an ambulatory person age 75 or older has had a diagnosis of symptomatic osteoarthritis for >6 months, THEN there should be evidence that education regarding the natural history, treatment, and self management of the disease was offered at least once.	1. Ganz DA, Chang JT, Roth CP, Guan M, Kamberg CJ, Niu F, et al. Quality of osteoarthritis care for community-dwelling older adults. Arthritis and rheumatism. 2006;55(2):241-7.
26	307	Refer person age 75 or older to the surgeon to make an assessment	Process	Chronic	Follow up and continuity	Effective	L - Musculoskeletal	IF a person age 75 or older with severe symptomatic osteoarthritis of the knee or hip has failed to respond to nonpharmacologic and pharmacologic therapy, THEN the patient should be offered referral to an orthopedic surgeon to be evaluated for total joint replacement within 6 months unless a contraindication to surgery is documented.	1. Ganz DA, Chang JT, Roth CP, Guan M, Kamberg CJ, Niu F, et al. Quality of osteoarthritis care for community-dwelling older adults. Arthritis and rheumatism. 2006;55(2):241-7.
26	308	First oral pharmacologic therapy	Process	Chronic	Treatment	Effective	L - Musculoskeletal	IF oral pharmacologic therapy is initiated to treat osteoarthritis, THEN acetaminophen should be the first drug used, unless there is a documented contraindication to use.	1. Ganz DA, Chang JT, Roth CP, Guan M, Kamberg CJ, Niu F, et al. Quality of osteoarthritis care for community-dwelling older adults. Arthritis and rheumatism. 2006;55(2):241-7.
26	309	Notice person age 75 or older of the risks associeted with drug of treatment	Process	Chronic	Follow up and continuity	Effective	L - Musculoskeletal	IF a person age 75 or older is treated with a nonselective nonsteroidal antiinflammatory drug, THEN the patient should be advised of the risks associated with the drug.	1. Ganz DA, Chang JT, Roth CP, Guan M, Kamberg CJ, Niu F, et al. Quality of osteoarthritis care for community-dwelling older adults. Arthritis and rheumatism. 2006;55(2):241-7.
26	310	Concomitant treatment with either misoprostol or a proton-pump inhibitor	Process	Chronic	Treatment	Effective	L - Musculoskeletal	IF a vulnerable elder is older than age 75 years or has a history of peptic ulcer disease, gastrointestinal bleeding, or current coumadin use, AND the patient is being treated with a cyclooxygenase nonselective NSAID, THEN he or she should be offered concomitant treatment with misoprostol or a proton-pump inhibitor.	1. Ganz DA, Chang JT, Roth CP, Guan M, Kamberg CJ, Niu F, et al. Quality of osteoarthritis care for community-dwelling older adults. Arthritis and rheumatism. 2006;55(2):241-7.
26	311	Diagnostic aspiration of the painfully swollenred joint	Process	Chronic	Diagnosis	Effective	L - Musculoskeletal	IF a vulnerable elder has monoarticular joint pain associated with redness, warmth, or swelling and the patient also has an oral temperature greater than 38.0 °C and does not have a previously established diagnosis of pseudogout or gout, THEN a diagnostic aspiration of the painfully swollen red joint should be performed that day.	1. Wenger NS, Solomon DH, Roth CP, MacLean CH, Saliba D, Kamberg CJ, et al. The quality of medical care provided to vulnerable community-dwelling older patients. Annals of internal medicine. 2003;139(9):740-7.
26	312	Exercise prescription for vulnerable elders diagnosed and with symptomatic more than 12 months ago	Process	Chronic	Treatment	Effective	L - Musculoskeletal	IF an ambulatory vulnerable elder has had a diagnosis of symptomatic osteoarthritis of the knee for more than 12 months, has no contraindication to exercise, and is physically and mentally able to exercise, THEN there should be evidence that a directed or supervised strengthening or aerobic exercise program was prescribed at least once since the time of diagnosis	1. Wenger NS, Solomon DH, Roth CP, MacLean CH, Saliba D, Kamberg CJ, et al. The quality of medical care provided to vulnerable community-dwelling older patients. Annals of internal medicine. 2003;139(9):740-7.
26	313	Education for vulnerable elders diagnosed more than 12 months, since the time of diagnosis	Process	Chronic	Screening and prevention	Effective	L - Musculoskeletal	IF an ambulatory vulnerable elder has had a diagnosis of symptomatic osteoarthritis of the knee for more than 12 months, THEN there should be evidence that the patient was offered education at least once since the time of diagnosis.	1. Wenger NS, Solomon DH, Roth CP, MacLean CH, Saliba D, Kamberg CJ, et al. The quality of medical care provided to vulnerable community-dwelling older patients. Annals of internal medicine. 2003;139(9):740-7.
26	314	Trial of maximum-dose acetaminophen before pharmacologic therapy from acetaminophen be changed to a different oral agent	Process	Chronic	Treatment	Effective	L - Musculoskeletal	IF oral pharmacologic therapy for osteoarthritis is changed from acetaminophen to a different oral agent, THEN there should be evidence that the patient has had a trial of maximum-dose acetaminophen (suitable for age and comorbid conditions).	1. Wenger NS, Solomon DH, Roth CP, MacLean CH, Saliba D, Kamberg CJ, et al. The quality of medical care provided to vulnerable community-dwelling older patients. Annals of internal medicine. 2003;139(9):740-7.
26	315	Notice vulnerable elders of the risks associeted with drug of treatment	Process	Chronic	Follow up and continuity	Effective	L - Musculoskeletal	IF a vulnerable elder is treated with cyclooxygenase nonselective NSAIDS, THEN there should be evidence that the patient was advised of the risks associated with these drugs	1. Wenger NS, Solomon DH, Roth CP, MacLean CH, Saliba D, Kamberg CJ, et al. The quality of medical care provided to vulnerable community-dwelling older patients. Annals of internal medicine. 2003;139(9):740-7.
26	316	Records that they have been offered health education of the disease at least once	Process	Chronic	Follow up and continuity	Effective	L - Musculoskeletal	The percentage of patients with symptomatic osteoarthritis, whose notes contain a record that they have been offered education regarding the natural history, treatment, and self-management of the disease at least once	1. Broadbent J, Maisey S, Holland R, Steel N. Recorded quality of primary care for osteoarthritis: an observational study. The British journal of general practice : the journal of the Royal College of General Practitioners. 2008;58(557):839-43.
26	317	Records that patients have been advised of the gastrointestinal and renal risks associated with treatment	Process	Chronic	Follow up and continuity	Effective	L - Musculoskeletal	The percentage of patients with osteoarthritis treated with an NSAID, whose notes contain a record that they have been advised of the gastrointestinal and renal risks associated with this drug	1. Broadbent J, Maisey S, Holland R, Steel N. Recorded quality of primary care for osteoarthritis: an observational study. The British journal of general practice : the journal of the Royal College of General Practitioners. 2008;58(557):839-43.
26	318	Records that patients treated for symptomatic osteoarthritis have been assessed for functional status in the last year	Process	Chronic	Follow up and continuity	Effective	L - Musculoskeletal	The percentage of patients treated for symptomatic osteoarthritis, whose notes contain a record that they have been assessed for functional status in the last year	1. Broadbent J, Maisey S, Holland R, Steel N. Recorded quality of primary care for osteoarthritis: an observational study. The British journal of general practice : the journal of the Royal College of General Practitioners. 2008;58(557):839-43.
26	319	Records that patients treated for symptomatic osteoarthritis have been assessed for degree of pain in the last year	Process	Chronic	Follow up and continuity	Effective	L - Musculoskeletal	The percentage of patients treated for symptomatic osteoarthritis, whose notes contain a record that they have been assessed for degree of pain in the last year	1. Broadbent J, Maisey S, Holland R, Steel N. Recorded quality of primary care for osteoarthritis: an observational study. The British journal of general practice : the journal of the Royal College of General Practitioners. 2008;58(557):839-43.
26	320	Record that patients with osteoarthritis regularly treated with an NSAID have been asked about gastrointestinal symptoms within the previous 12 months	Process	Chronic	Follow up and continuity	Effective	L - Musculoskeletal	The percentage of patients with osteoarthritis regularly treated with an NSAID, whose notes contain a record that they have been asked about gastrointestinal symptoms within the previous 12 months	1. Broadbent J, Maisey S, Holland R, Steel N. Recorded quality of primary care for osteoarthritis: an observational study. The British journal of general practice : the journal of the Royal College of General Practitioners. 2008;58(557):839-43.
26	321	Records that patients in whom first oral pharmacological therapy to treat osteoarthritis was initiated with paracetamol	Process	Chronic	Follow up and continuity	Effective	L - Musculoskeletal	The percentage of patients in whom oral pharmacological therapy was initiated to treat osteoarthritis,whose notes contain a record that they were offered paracetamol first (unless contraindicated)	1. Broadbent J, Maisey S, Holland R, Steel N. Recorded quality of primary care for osteoarthritis: an observational study. The British journal of general practice : the journal of the Royal College of General Practitioners. 2008;58(557):839-43.
26	322	Records that ibuprofen (or a cox-2 inhibitor) has been considered for first-line treatment for patients with osteoarthritis treated with an NSAID	Process	Chronic	Follow up and continuity	Effective	L - Musculoskeletal	The percentage of patients with osteoarthritis treated with an NSAID, whose notes contain a record that ibuprofen (or a cox-2 inhibitor) has been considered for first-line treatment (unless contraindicated or intolerant)	1. Broadbent J, Maisey S, Holland R, Steel N. Recorded quality of primary care for osteoarthritis: an observational study. The British journal of general practice : the journal of the Royal College of General Practitioners. 2008;58(557):839-43.
26	323	Records that referral to an orthopaedic surgeon for patients with severe symptomatic osteoarthritis of the knee or hip that has failed to respond to non-pharmacological and pharmacological therapy	Process	Chronic	Follow up and continuity	Effective	L - Musculoskeletal	The percentage of patients with severe symptomatic osteoarthritis of the knee or hip that has failed to respond to non-pharmacological and pharmacological therapy, whose notes contain a record that they were offered referral to an orthopaedic surgeon to be evaluated for total joint replacement within 6 months unless surgery is contraindicated	1. Broadbent J, Maisey S, Holland R, Steel N. Recorded quality of primary care for osteoarthritis: an observational study. The British journal of general practice : the journal of the Royal College of General Practitioners. 2008;58(557):839-43.
26	324	Register that were offered a trial of maximum-dose paracetamol for patients in whom oral pharmacological therapy was changed from paracetamol to a different oral agent	Process	Chronic	Follow up and continuity	Effective	L - Musculoskeletal	The percentage of patients in whom oral pharmacological therapy was changed from paracetamol to a different oral agent, whose notes contain a record that they were offered a trial of maximum-dose paracetamol.	1. Broadbent J, Maisey S, Holland R, Steel N. Recorded quality of primary care for osteoarthritis: an observational study. The British journal of general practice : the journal of the Royal College of General Practitioners. 2008;58(557):839-43.
26	325	Use paracetamol as first therapy oral pharmacological to treat osteoarthritis among people aged 50 or older	Process	Chronic	Treatment	Effective	L - Musculoskeletal	If oral pharmacological therapy is initiated to treat osteoarthritis among people aged 50 or older, then paracetamol should be the first drug used, unless there is a contraindication to use	1. Steel N, Bachmann M, Maisey S, Shekelle P, Breeze E, Marmot M, et al. Self reported receipt of care consistent with 32 quality indicators: national population survey of adults aged 50 or more in England. Bmj. 2008;337:a957.
26	326	Records that patients in whom first oral pharmacological therapy to treat osteoarthritis was initiated with paracetamol	Process	Chronic	Follow up and continuity	Effective	L - Musculoskeletal	IF oral pharmacological therapy is initiated to treat osteoarthritis among people aged 65 or older, THEN paracetamol should be the first drug used, unless there is a contraindication to use.	1. Steel N, Maisey S, Clark A, Fleetcroft R, Howe A. Quality of clinical primary care and targeted incentive payments: an observational study. The British journal of general practice : the journal of the Royal College of General Practitioners. 2007;57(539):449-54.
26	327	Number of patients with a new diagnosis of osteoarthritis who wish to take medication for joint symptoms and trial of paracetamol	Process	Chronic	Treatment	Effective	L - Musculoskeletal	Patients with a new diagnosis of osteoarthritis who wish to take medication for joint symptoms should be offered a trial of paracetamol if not already tried.	1. Steel N, Maisey S, Clark A, Fleetcroft R, Howe A. Quality of clinical primary care and targeted incentive payments: an observational study. The British journal of general practice : the journal of the Royal College of General Practitioners. 2007;57(539):449-54.
26	328	Trial of maximum-dose paracetamol before pharmacologic therapy from paracetamol be changed to a different oral agent	Process	Chronic	Treatment	Effective	L - Musculoskeletal	IF oral pharmacological therapy for osteoarthritis is changed fro paracetamol to a different oral agent among people aged 65 or older, THEN the patient should have had a trial of maximum dose paracetamol (suitable for age/comorbidities).	1. Steel N, Maisey S, Clark A, Fleetcroft R, Howe A. Quality of clinical primary care and targeted incentive payments: an observational study. The British journal of general practice : the journal of the Royal College of General Practitioners. 2007;57(539):449-54.
26	329	Ibuprofen has been considered for first-line treatment for patients with osteoarthritis treated with an NSAID	Process	Chronic	Treatment	Effective	L - Musculoskeletal	If NSAIDS are considered, ibuprofen should be considered for first line treatment unless contraindicated or intolerant.	1. Steel N, Maisey S, Clark A, Fleetcroft R, Howe A. Quality of clinical primary care and targeted incentive payments: an observational study. The British journal of general practice : the journal of the Royal College of General Practitioners. 2007;57(539):449-54.
26	330	Annual assessment for patients aged 65 or older diagnosed and with symptoms of osteoarthritis	Outcome	Chronic	Follow up and continuity	Effective	L - Musculoskeletal	IF a person aged 65 or older is treated for symptomatic osteoarthritis, THEN functional status and degree of pain should be assessed at least annually.	1. Steel N, Maisey S, Clark A, Fleetcroft R, Howe A. Quality of clinical primary care and targeted incentive payments: an observational study. The British journal of general practice : the journal of the Royal College of General Practitioners. 2007;57(539):449-54.
26	331	Counseling/Education about natural history , treatment and management of symtomatic osteoarthritis for patients aged 65 or older diagnosed	Process	Chronic	Follow up and continuity	Effective	L - Musculoskeletal	IF an ambulatory person aged 65 or older has a diagnosis of symptomatic osteoarthritis, THEN education regarding the natural history, treatment and self-management of the disease should be offered at least once	1. Steel N, Maisey S, Clark A, Fleetcroft R, Howe A. Quality of clinical primary care and targeted incentive payments: an observational study. The British journal of general practice : the journal of the Royal College of General Practitioners. 2007;57(539):449-54.
26	332	Advised of the risks associeted with treatment with a non-selective NSAID or COX-2 selective NSAID for pacients aged 65 or older	Process	Chronic	Treatment	Effective	L - Musculoskeletal	IF a person aged 65 or older is treated with a non-selective NSAID or with a COX-2 selective NSAID THEN the patient should be advised of the gastrointestinal and renal risks associated with this drug.	1. Steel N, Maisey S, Clark A, Fleetcroft R, Howe A. Quality of clinical primary care and targeted incentive payments: an observational study. The British journal of general practice : the journal of the Royal College of General Practitioners. 2007;57(539):449-54.
26	333	Monitoring gastrointestinal symptoms during treatment with a NSAID for pacients aged 65 or older annually	Process	Chronic	Follow up and continuity	Effective	L - Musculoskeletal	IF a person aged 65 or over is treated with an NSAID (selective or non-selective), THEN they should be asked about gastro-intestinal symptoms at least annually.	1. Steel N, Maisey S, Clark A, Fleetcroft R, Howe A. Quality of clinical primary care and targeted incentive payments: an observational study. The British journal of general practice : the journal of the Royal College of General Practitioners. 2007;57(539):449-54.
26	334	Refer patients aged 65 or older with severe symptomatic osteoarthritis of the knee or hip to the orthopedic surgeon to make an assessment of total joint replacement	Process	Acute / Chronic	Follow up and continuity	Effective	L - Musculoskeletal	IF a person aged 65 or older with severe symptomatic osteoarthritis of the knee or hip has failed to respond to non- pharmacological and pharmacological therapy, THEN the patient should be offered referral to an orthopaedic surgeon to be evaluated for total joint replacement within 6 months unless surgery is contraindicated.	1. Steel N, Maisey S, Clark A, Fleetcroft R, Howe A. Quality of clinical primary care and targeted incentive payments: an observational study. The British journal of general practice : the journal of the Royal College of General Practitioners. 2007;57(539):449-54.
26	335	Continuity of care	Process	Chronic	Follow up and continuity	Patient-centered	L - Musculoskeletal	Patient questionnaire (The Osteoarthritis Quality Indicator questionnaire) with 17 items, where each rated on aspects as disease development ,treatment alternatives, self-management, lifestyle, physical activity, referral physical activity, weight reduction, referral weight reduction, functional assessment, walking aid assessment, other aids assessment, pain assessment , acetaminophen, stronger pain killers ,NSAIDs, cortisone and referral to orthopedic surgeon.	1. Osteras N, Garratt A, Grotle M, Natvig B, Kjeken I, Kvien TK, et al. Patient-reported quality of care for osteoarthritis: development and testing of the osteoarthritis quality indicator questionnaire. Arthritis care & research. 2013;65(7):1043-51. 2. Osteras N, Jordan KP, Clausen B, Cordeiro C, Dziedzic K, Edwards J, et al. Self-reported quality care for knee osteoarthritis: comparisons across Denmark, Norway, Portugal and the UK. RMD Open. 2015;1(1):e000136
3	336	Accommodation "patient-focused on": Home visits	Process	Chronic	All	Patient-centered	Not Defined	PHC home visits	1. Kringos, D. S., Boerma, W. G., Hutchinson, A., van der Zee, J., & Groenewegen, P. P. (2010). The breadth of primary care: a systematic literature review of its core dimensions. BMC Health Services Research, 10(1) // 2. Ansari, Z. (2007). A Review of Literature on Access to Primary Health Care. Australian Journal of Primary Health, 13(2), 80.
3	337	Quality of health promotion: Obesity prevalence	Outcome	Chronic	Screening and prevention	Effective	T - Endocrine/Metabolic and Nutritional	Prevalence of obesity (19 to 64 anos) - 25> Body Mass Index <30	1. Kringos, D. S., Boerma, W. G., Hutchinson, A., van der Zee, J., & Groenewegen, P. P. (2010). The breadth of primary care: a systematic literature review of its core dimensions. BMC Health Services Research, 10(1) //
3	338	Quality of health promotion: Physical activity	Outcome	Chronic	Screening and prevention	Effective	P - Psychological	Physical activity	1. Kringos, D. S., Boerma, W. G., Hutchinson, A., van der Zee, J., & Groenewegen, P. P. (2010). The breadth of primary care: a systematic literature review of its core dimensions. BMC Health Services Research, 10(1) // 2. Marshall M, Klazinga N, Leatherman S, Hardy C, Bergmann E, Pisco L, et al: OECD Health Care Quality Indicator Project. The expert panel on primary care prevention and health promotion. Int J Qual Health Care 2006, 18(Suppl 1):21-25.

3	339	Quality of health promotion: Smoking rate	Outcome	Chronic	Screening and prevention	Effective	P - Psychological	Smoking rate	1. Kringos, D. S., Boerma, W. G., Hutchinson, A., van der Zee, J., & Groenewegen, P. P. (2010). The breadth of primary care: a systematic literature review of its core dimensions. BMC Health Services Research, 10(1) // 2. Marshall M, Klazinga N, Leatherman S, Hardy C, Bergmann E, Pisco L, et al: OECD Health Care Quality Indicator Project. The expert panel on primary care prevention and health promotion. Int J Qual Health Care 2006, 18(Suppl 1):21-25.
3	340	Quality of health promotion: Diabetes prevalence	Outcome	Chronic	Screening and prevention	Effective	T - Endocrine/Metabolic and Nutritional	Diabetes prevalence	1. Kringos, D. S., Boerma, W. G., Hutchinson, A., van der Zee, J., & Groenewegen, P. P. (2010). The breadth of primary care: a systematic literature review of its core dimensions. BMC Health Services Research, 10(1) // 2. Marshall M, Klazinga N, Leatherman S, Hardy C, Bergmann E, Pisco L, et al: OECD Health Care Quality Indicator Project. The expert panel on primary care prevention and health promotion. Int J Qual Health Care 2006, 18(Suppl 1):21-25.
3	341	Diagnosis and treatment - primary care: First visit in first trimester (CHF)	Process	Chronic	Screening and prevention	Timely	K - Cardiovascular	Diagnosis and treatment - primary care: First visit in first trimester	1. Kringos, D. S., Boerma, W. G., Hutchinson, A., van der Zee, J., & Groenewegen, P. P. (2010). The breadth of primary care: a systematic literature review of its core dimensions. BMC Health Services Research, 10(1) // 2. Marshall M, Klazinga N, Leatherman S, Hardy C, Bergmann E, Pisco L, et al: OECD Health Care Quality Indicator Project. The expert panel on primary care prevention and health promotion. Int J Qual Health Care 2006, 18(Suppl 1):21-25.
3	342	Diagnosis and treatment - primary care: Smoking cessation counselling for asthmatics	Process	Chronic	Screening and prevention	Patient-centered	R - Respiratory	Diagnosis and treatment - primary care: Smoking cessation counselling for asthmatics	1. Kringos, D. S., Boerma, W. G., Hutchinson, A., van der Zee, J., & Groenewegen, P. P. (2010). The breadth of primary care: a systematic literature review of its core dimensions. BMC Health Services Research, 10(1) // 2. Marshall M, Klazinga N, Leatherman S, Hardy C, Bergmann E, Pisco L, et al: OECD Health Care Quality Indicator Project. The expert panel on primary care prevention and health promotion. Int J Qual Health Care 2006, 18(Suppl 1):21-25.
3	343	Diagnosis and treatment - primary care: Blood pressure measurement	Process	Chronic	Diagnosis / Treatment	Effective	K - Cardiovascular	Diagnosis and treatment - primary care: Blood pressure measurement	1. Kringos, D. S., Boerma, W. G., Hutchinson, A., van der Zee, J., & Groenewegen, P. P. (2010). The breadth of primary care: a systematic literature review of its core dimensions. BMC Health Services Research, 10(1) // 2. Marshall M, Klazinga N, Leatherman S, Hardy C, Bergmann E, Pisco L, et al: OECD Health Care Quality Indicator Project. The expert panel on primary care prevention and health promotion. Int J Qual Health Care 2006, 18(Suppl 1):21-25.
3	344	Diagnosis and treatment - primary care: Re-measurement of blood pressure for those with high blood pressure	Process	Chronic	Follow up and continuity	Effective	K - Cardiovascular	Diagnosis and treatment - primary care: Re-measurement of blood pressure for those with high blood pressure	1. Kringos, D. S., Boerma, W. G., Hutchinson, A., van der Zee, J., & Groenewegen, P. P. (2010). The breadth of primary care: a systematic literature review of its core dimensions. BMC Health Services Research, 10(1) // 2. Marshall M, Klazinga N, Leatherman S, Hardy C, Bergmann E, Pisco L, et al: OECD Health Care Quality Indicator Project. The expert panel on primary care prevention and health promotion. Int J Qual Health Care 2006, 18(Suppl 1):21-25.
4	345	Continuity with provider	Process	Chronic	Follow up and continuity	Patient-centered	A - General and unspecified	Continuity with provider	1. Menear, M., Doré, I., Cloutier, A.-M., Perrier, L., Roberge, P., Duhoux, A., … Fournier, L. (2015). Chronic physical comorbidity burden and the quality of depression treatment in primary care: A systematic review. Journal of Psychosomatic Research, 78(4), 314–323. // 2. Houle J, et al. Inequities in medical follow-up for depression: a population-based study in Montreal. Psychiatr Serv 2010;61:258–63.
4	346	Intensity of follow-up	Process	Chronic	Follow up and continuity	Patient-centered	A - General and unspecified	Intensity of follow-up	1. Menear, M., Doré, I., Cloutier, A.-M., Perrier, L., Roberge, P., Duhoux, A., … Fournier, L. (2015). Chronic physical comorbidity burden and the quality of depression treatment in primary care: A systematic review. Journal of Psychosomatic Research, 78(4), 314–323. // 2. Houle J, et al. Inequities in medical follow-up for depression: a population-based study in Montreal. Psychiatr Serv 2010;61:258–63.
4	347	Promptness of follow-up	Process	Chronic	Follow up and continuity	Timely	A - General and unspecified	Promptness of follow-up	1. Menear, M., Doré, I., Cloutier, A.-M., Perrier, L., Roberge, P., Duhoux, A., … Fournier, L. (2015). Chronic physical comorbidity burden and the quality of depression treatment in primary care: A systematic review. Journal of Psychosomatic Research, 78(4), 314–323. // 2. Houle J, et al. Inequities in medical follow-up for depression: a population-based study in Montreal. Psychiatr Serv 2010;61:258–63.
8	348	Body mass index (BMI) screening and lifestyle counseling	Process	Chronic	Screening and prevention	Patient-centered	A - General and unspecified	A) IF a patient has RA, THEN their BMI should be documented at least once every year, AND B) if they are overweight or obese according to national guidelines, they should be counseled to modify their lifestyle	1. Barber, C. E. H., Marshall, D. A., Alvarez, N., Mancini, G. B. J., Lacaille, D., … Keeling, S. (2015). Development of Cardiovascular Quality Indicators for Rheumatoid Arthritis: Results from an International Expert Panel Using a Novel Online Process. The Journal of Rheumatology, 42(9), 1548–1555. // 2. Barber CE, Smith A, Esdaile JM, Barnabe C, Martin LO, Faris P, et al. Best practices for cardiovascular disease prevention in rheumatoid arthritis: a systematic review of guideline recommendations and quality indicators. Arthritis Care Res 2015;67:169-79.
8	349	Communication of increased CV risk in RA	Process	Chronic	Screening and prevention	Patient-centered	K - Cardiovascular	IF a patient has RA, THEN the treating rheumatologist should communicate to the primary care physician (PCP), at least once within the last 2 years, that patients with RA have an increased CV risk.	1. Barber, C. E. H., Marshall, D. A., Alvarez, N., Mancini, G. B. J., Lacaille, D., … Keeling, S. (2015). Development of Cardiovascular Quality Indicators for Rheumatoid Arthritis: Results from an International Expert Panel Using a Novel Online Process. The Journal of Rheumatology, 42(9), 1548–1555. // 2. Barber CE, Smith A, Esdaile JM, Barnabe C, Martin LO, Faris P, et al. Best practices for cardiovascular disease prevention in rheumatoid arthritis: a systematic review of guideline recommendations and quality indicators. Arthritis Care Res 2015;67:169-79.
8	350	CV risk assessment	Process	Chronic	Screening and prevention	Effective	K - Cardiovascular	A) IF a patient has RA, THEN a formal CV risk assessment according to national guidelines should be done at least once in the first 2 years after evaluation by a rheumatologist; AND B) if low risk, it should be repeated once every 5 years; OR C) if initial assessment suggests intermediate or high-risk, THEN treatment of risk factors according to national guidelines should be recommended.	1. Barber, C. E. H., Marshall, D. A., Alvarez, N., Mancini, G. B. J., Lacaille, D., … Keeling, S. (2015). Development of Cardiovascular Quality Indicators for Rheumatoid Arthritis: Results from an International Expert Panel Using a Novel Online Process. The Journal of Rheumatology, 42(9), 1548–1555. // 2. Barber CE, Smith A, Esdaile JM, Barnabe C, Martin LO, Faris P, et al. Best practices for cardiovascular disease prevention in rheumatoid arthritis: a systematic review of guideline recommendations and quality indicators. Arthritis Care Res 2015;67:169-79.
8	351	Communication to PCP about a documented high blood pressure	Process	Chronic	Screening and prevention	Effective	K - Cardiovascular	IF a patient has RA AND has a blood pressure measure during a rheumatology clinic visit that is elevated (systolic blood pressure ≥ 140 and/or diastolic blood pressure ≥ 90), THEN the rheumatologist should recommend that it be repeated and treatment initiated or adjusted if indicated.	1. Barber, C. E. H., Marshall, D. A., Alvarez, N., Mancini, G. B. J., Lacaille, D., … Keeling, S. (2015). Development of Cardiovascular Quality Indicators for Rheumatoid Arthritis: Results from an International Expert Panel Using a Novel Online Process. The Journal of Rheumatology, 42(9), 1548–1555. // 2. Barber CE, Smith A, Esdaile JM, Barnabe C, Martin LO, Faris P, et al. Best practices for cardiovascular disease prevention in rheumatoid arthritis: a systematic review of guideline recommendations and quality indicators. Arthritis Care Res 2015;67:169-79.
8	352	Measurement of a lipid profile	Process	Chronic	Screening and prevention	Effective	T - Endocrine/Metabolic and Nutritional	IF a patient has RA, THEN a lipid profile should be done at least once in the first 2 years after evaluation by a rheumatologist AND A) if low risk according to CV risk scores, the lipid profile should be repeated once every 5 years; OR B) if CV risk assessment suggests intermediate or high risk, then treatment according to national guidelines should be recommended.	1. Barber, C. E. H., Marshall, D. A., Alvarez, N., Mancini, G. B. J., Lacaille, D., … Keeling, S. (2015). Development of Cardiovascular Quality Indicators for Rheumatoid Arthritis: Results from an International Expert Panel Using a Novel Online Process. The Journal of Rheumatology, 42(9), 1548–1555. // 2. Barber CE, Smith A, Esdaile JM, Barnabe C, Martin LO, Faris P, et al. Best practices for cardiovascular disease prevention in rheumatoid arthritis: a systematic review of guideline recommendations and quality indicators. Arthritis Care Res 2015;67:169-79.
8	353	Minimizing corticosteroid usage	Process	Chronic	Follow up and continuity	Safe	A - General and unspecified	IF a patient with RA is taking oral corticosteroids, THEN there should be evidence of intent to taper the corticosteroids or reduce to the lowest possible dose.	1. Barber, C. E. H., Marshall, D. A., Alvarez, N., Mancini, G. B. J., Lacaille, D., … Keeling, S. (2015). Development of Cardiovascular Quality Indicators for Rheumatoid Arthritis: Results from an International Expert Panel Using a Novel Online Process. The Journal of Rheumatology, 42(9), 1548–1555. // 2. Barber CE, Smith A, Esdaile JM, Barnabe C, Martin LO, Faris P, et al. Best practices for cardiovascular disease prevention in rheumatoid arthritis: a systematic review of guideline recommendations and quality indicators. Arthritis Care Res 2015;67:169-79.
8	354	Screening for diabetes	Process	Chronic	Screening and prevention	Effective	T - Endocrine/Metabolic and Nutritional	IF a patient has RA, THEN diabetes should be screened for as part of a CV risk assessment at least once within the first 2 years of evaluation by a rheumatologist and A) once every 5 years in low-risk patients or B) yearly in intermediate- or high-risk patients AND if screening is abnormal, this information should be communicated to the primary care provider for appropriate followup and management, if indicated. Note: Risk here denotes risk of diabetes and assessment of diabetes risk is described in detail in the full specifications for the quality indicators (shown in the Supplementary Table, available online at jrheum.org).	1. Barber, C. E. H., Marshall, D. A., Alvarez, N., Mancini, G. B. J., Lacaille, D., … Keeling, S. (2015). Development of Cardiovascular Quality Indicators for Rheumatoid Arthritis: Results from an International Expert Panel Using a Novel Online Process. The Journal of Rheumatology, 42(9), 1548–1555. // 2. Barber CE, Smith A, Esdaile JM, Barnabe C, Martin LO, Faris P, et al. Best practices for cardiovascular disease prevention in rheumatoid arthritis: a systematic review of guideline recommendations and quality indicators. Arthritis Care Res 2015;67:169-79.
8	355	Screening for hypertension	Process	Chronic	Screening and prevention	Effective	K - Cardiovascular	IF a patient has RA, THEN their blood pressure should be measured and documented in the medical record at ≥ 80% of clinic visits.	1. Barber, C. E. H., Marshall, D. A., Alvarez, N., Mancini, G. B. J., Lacaille, D., … Keeling, S. (2015). Development of Cardiovascular Quality Indicators for Rheumatoid Arthritis: Results from an International Expert Panel Using a Novel Online Process. The Journal of Rheumatology, 42(9), 1548–1555. // 2. Barber CE, Smith A, Esdaile JM, Barnabe C, Martin LO, Faris P, et al. Best practices for cardiovascular disease prevention in rheumatoid arthritis: a systematic review of guideline recommendations and quality indicators. Arthritis Care Res 2015;67:169-79.
8	356	Smoking status and cessation counseling	Process	Chronic	Screening and prevention	Effective	A - General and unspecified	A) IF a patient has RA, THEN their smoking and tobacco use status should be documented at least once in the last year, AND B) if they are current smokers or tobacco users they should be counseled to stop smoking.	1. Barber, C. E. H., Marshall, D. A., Alvarez, N., Mancini, G. B. J., Lacaille, D., … Keeling, S. (2015). Development of Cardiovascular Quality Indicators for Rheumatoid Arthritis: Results from an International Expert Panel Using a Novel Online Process. The Journal of Rheumatology, 42(9), 1548–1555. // 2. Barber CE, Smith A, Esdaile JM, Barnabe C, Martin LO, Faris P, et al. Best practices for cardiovascular disease prevention in rheumatoid arthritis: a systematic review of guideline recommendations and quality indicators. Arthritis Care Res 2015;67:169-79.
3 (24)	357	Comprehensive diabetes care: HbA1c testing	Process	Chronic	Screening and prevention	Effective	T - Endocrine/Metabolic and Nutritional	Percentage of patients with type 1 or type 2 diabetes who were 18–75 years old and had a hemoglobin A1c test during the measurement year.	1. Kringos, D. S., Boerma, W. G., Hutchinson, A., van der Zee, J., & Groenewegen, P. P. (2010). The breadth of primary care: a systematic literature review of its core dimensions. BMC Health Services Research, 10(1) // Friedberg MW, Coltin KL, Pearson SD, Kleinman KP, Zheng J, Singer JA, et al: Does affiliation of physician groups with one another produce higher quality primary care? J Gen Intern Med 2007, 22:1385-1392.
3 (24)	358	Comprehensive diabetes care: eye exams	Process	Chronic	Screening and prevention	Effective	T - Endocrine/Metabolic and Nutritional	Percentage of patients with type 1 or type 2 diabetes who were 18–75 years old and had a retinal or dilated eye exam by an eye care professional in the measurement year or a negative retinal exam (no evidence of retinopathy) by an eye care professional in the year before the measurement year. A retinal or dilated eye exam by an eye care professional in the measurement year (regardless of results) or A retinal or dilated eye exam by an eye care professional in the year prior to the measurement year that was negative for retinopathy.	1. Kringos, D. S., Boerma, W. G., Hutchinson, A., van der Zee, J., & Groenewegen, P. P. (2010). The breadth of primary care: a systematic literature review of its core dimensions. BMC Health Services Research, 10(1) // Friedberg MW, Coltin KL, Pearson SD, Kleinman KP, Zheng J, Singer JA, et al: Does affiliation of physician groups with one another produce higher quality primary care? J Gen Intern Med 2007, 22:1385-1392.
3 (24)	359	Comprehensive diabetes care: LDL-C screening	Process	Chronic	Screening and prevention	Effective	T - Endocrine/Metabolic and Nutritional	Percentage of patients with type 1 or type 2 diabetes who were 18–75 years old and had a low-density lipoprotein cholesterol (LDL-C) test performed during the measurement year or year before the measurement year. Measure the percentage of members 18 to 75 years of age with diabetes (type 1 and type 2) who have had their cholesterol level checked and have had their cholesterol level controlled.	1. Kringos, D. S., Boerma, W. G., Hutchinson, A., van der Zee, J., & Groenewegen, P. P. (2010). The breadth of primary care: a systematic literature review of its core dimensions. BMC Health Services Research, 10(1) // Friedberg MW, Coltin KL, Pearson SD, Kleinman KP, Zheng J, Singer JA, et al: Does affiliation of physician groups with one another produce higher quality primary care? J Gen Intern Med 2007, 22:1385-1392.
3 (24)	360	Comprehensive diabetes care: monitoring diabetic nephropathy	Process	Chronic	Screening and prevention	Effective	T - Endocrine/Metabolic and Nutritional	Percentage of patients with type 1 or type 2 diabetes who were 18–75 years old and have been screened, during the measurement year or year before the measurement year, for urine microalbumin or have nephropathy, as demonstrated by either evidence of medical attention for nephropathy, a visit to nephrologist, or a positive urine macroalbumin test.	1. Kringos, D. S., Boerma, W. G., Hutchinson, A., van der Zee, J., & Groenewegen, P. P. (2010). The breadth of primary care: a systematic literature review of its core dimensions. BMC Health Services Research, 10(1) // Friedberg MW, Coltin KL, Pearson SD, Kleinman KP, Zheng J, Singer JA, et al: Does affiliation of physician groups with one another produce higher quality primary care? J Gen Intern Med 2007, 22:1385-1392.
3 (24)	361	Appropriate asthma medications for adults ages 18 to 56	Process	Chronic	Treatment	Effective	R - Respiratory	Percentage of enrolled members aged 18 to 56 years during the measurement year who were identified as having persistent asthma during the year before the measurement year and who were appropriately prescribed medication during the measurement year (i.e., those who had at least 1 dispensed prescription for inhaled corticosteroids, nedocromil, cromolyn sodium, leukotriene modifiers, or methylxanthines during the measurement year).	1. Kringos, D. S., Boerma, W. G., Hutchinson, A., van der Zee, J., & Groenewegen, P. P. (2010). The breadth of primary care: a systematic literature review of its core dimensions. BMC Health Services Research, 10(1) // Friedberg MW, Coltin KL, Pearson SD, Kleinman KP, Zheng J, Singer JA, et al: Does affiliation of physician groups with one another produce higher quality primary care? J Gen Intern Med 2007, 22:1385-1392.
3 (24)	362	Cholesterol screening test after acute cardiovascular events	Process	Chronic	Screening and prevention	Effective	T - Endocrine/Metabolic and Nutritional	Percentage of patients ages 18 through 75 who, from January 1 through November 1 of the year before the measurement year, were discharged alive for acute myocardial infarction (AMI), coronary artery bypass graft (CABG), or percutaneous transluminal coronary angioplasty (PTCA), or who had a diagnosis of ischemic vascular disease (IVD) and who had low-density lipoprotein cholesterol (LDL-C) test performed any time during the measurement year.	1. Kringos, D. S., Boerma, W. G., Hutchinson, A., van der Zee, J., & Groenewegen, P. P. (2010). The breadth of primary care: a systematic literature review of its core dimensions. BMC Health Services Research, 10(1) // Friedberg MW, Coltin KL, Pearson SD, Kleinman KP, Zheng J, Singer JA, et al: Does affiliation of physician groups with one another produce higher quality primary care? J Gen Intern Med 2007, 22:1385-1392.
3 (25)	363	Anti-hypertensive medications prescribed: % of the recommended	Process	Chronic	Treatment	Safe	K - Cardiovascular	Compliance to guidelines.	1. Kringos, D. S., Boerma, W. G., Hutchinson, A., van der Zee, J., & Groenewegen, P. P. (2010). The breadth of primary care: a systematic literature review of its core dimensions. BMC Health Services Research, 10(1) // Gene-Badia J, Ascaso C, Escaramis-Babiano G, Sampietro-Colom L, Catalan- Ramos A, Sans-Corrales M, et al: Personalised care, access, quality and team coordination are the main dimensions of family medicine output Fam Pract 2007, 24:41-47.
3 (25)	364	Anti-diabetes medications prescribed: % of the recommended	Process	Chronic	Treatment	Safe	T - Endocrine/Metabolic and Nutritional	Compliance to guidelines.	1. Kringos, D. S., Boerma, W. G., Hutchinson, A., van der Zee, J., & Groenewegen, P. P. (2010). The breadth of primary care: a systematic literature review of its core dimensions. BMC Health Services Research, 10(1) // Gene-Badia J, Ascaso C, Escaramis-Babiano G, Sampietro-Colom L, Catalan- Ramos A, Sans-Corrales M, et al: Personalised care, access, quality and team coordination are the main dimensions of family medicine output Fam Pract 2007, 24:41-47.
3 (25)	365	Anti-asthma medications prescribed: % of the recommended	Process	Chronic	Treatment	Safe	R - Respiratory	Compliance to guidelines.	1. Kringos, D. S., Boerma, W. G., Hutchinson, A., van der Zee, J., & Groenewegen, P. P. (2010). The breadth of primary care: a systematic literature review of its core dimensions. BMC Health Services Research, 10(1) // Gene-Badia J, Ascaso C, Escaramis-Babiano G, Sampietro-Colom L, Catalan- Ramos A, Sans-Corrales M, et al: Personalised care, access, quality and team coordination are the main dimensions of family medicine output Fam Pract 2007, 24:41-47.
3 (52)	366	Preventable adverse events in primary care related to drugs	Outcome	Chronic	Treatment	Safe	A - General and unspecified	Incorrect drug, Incorrect dose, Delayed administration, Omitted administration	1. Kringos, D. S., Boerma, W. G., Hutchinson, A., van der Zee, J., & Groenewegen, P. P. (2010). The breadth of primary care: a systematic literature review of its core dimensions. BMC Health Services Research, 10(1) // Ansari Z: A review of literature on access to primary health care. Aust J Prim Health 2007, 13:80-95.
3 (52)	367	Preventable adverse events in primary care related to diagnosis	Outcome	Chronic	Diagnosis	Safe	A - General and unspecified	Misdiagnosis: Missed diagnosis, Delayed diagnosis	1. Kringos, D. S., Boerma, W. G., Hutchinson, A., van der Zee, J., & Groenewegen, P. P. (2010). The breadth of primary care: a systematic literature review of its core dimensions. BMC Health Services Research, 10(1) // Ansari Z: A review of literature on access to primary health care. Aust J Prim Health 2007, 13:80-95.
8 (21)	368	Communication of increased cardiovascular disease risk in rheumatoid arthritis	Process	Chronic	Diagnosis	Effective	L - Musculoskeletal	Communication of increased CV risk in RA: IF a patient has RA, THEN the treating rheumatologist should communicate to the primary care physician (PCP), at least once within the last 2 years, that patients with RA have an increased CV risk.	1. Barber, C. E. H., Marshall, D. A., Alvarez, N., Mancini, G. B. J., Lacaille, D., … Keeling, S. (2015). Development of Cardiovascular Quality Indicators for Rheumatoid Arthritis: Results from an International Expert Panel Using a Novel Online Process. The Journal of Rheumatology, 42(9), 1548–1555. // Barber CE, Smith A, Esdaile JM, Barnabe C, Martin LO, Faris P, et al. Best practices for cardiovascular disease prevention in rheumatoid arthritis: a systematic review of guideline recommendations and quality indicators. Arthritis Care Res 2015;67:169-79.
8 (21)	369	Cardiovascular disease risk assessment	Process	Chronic	Screening and prevention	Effective	K - Cardiovascular	CV risk assessment: A) IF a patient has RA, THEN a formal CV risk assessment according to national guidelines should be done at least once in the first 2 years after evaluation by a rheumatologist; AND B) if low risk, it should be repeated once every 5 years; OR C) if initial assessment suggests intermediate or high-risk, THEN treatment of risk factors according to national guidelines should be recommended.	1. Barber, C. E. H., Marshall, D. A., Alvarez, N., Mancini, G. B. J., Lacaille, D., … Keeling, S. (2015). Development of Cardiovascular Quality Indicators for Rheumatoid Arthritis: Results from an International Expert Panel Using a Novel Online Process. The Journal of Rheumatology, 42(9), 1548–1555. // Barber CE, Smith A, Esdaile JM, Barnabe C, Martin LO, Faris P, et al. Best practices for cardiovascular disease prevention in rheumatoid arthritis: a systematic review of guideline recommendations and quality indicators. Arthritis Care Res 2015;67:169-79.
8 (21)	370	Smoking status and cessation counseling	Process	Chronic	Screening and prevention	Effective	A - General and unspecified	Smoking status and cessation counseling: A) IF a patient has RA, THEN their smoking and tobacco use status should be documented at least once in the last year, AND B) if they are current smokers or tobacco users they should be counseled to stop smoking.	1. Barber, C. E. H., Marshall, D. A., Alvarez, N., Mancini, G. B. J., Lacaille, D., … Keeling, S. (2015). Development of Cardiovascular Quality Indicators for Rheumatoid Arthritis: Results from an International Expert Panel Using a Novel Online Process. The Journal of Rheumatology, 42(9), 1548–1555. // Barber CE, Smith A, Esdaile JM, Barnabe C, Martin LO, Faris P, et al. Best practices for cardiovascular disease prevention in rheumatoid arthritis: a systematic review of guideline recommendations and quality indicators. Arthritis Care Res 2015;67:169-79.
8 (21)	371	Screening for hypertension	Process	Chronic	Screening and prevention	Effective	K - Cardiovascular	Screening for hypertension: IF a patient has RA, THEN their blood pressure should be measured and documented in the medical record at ≥ 80% of clinic visits	1. Barber, C. E. H., Marshall, D. A., Alvarez, N., Mancini, G. B. J., Lacaille, D., … Keeling, S. (2015). Development of Cardiovascular Quality Indicators for Rheumatoid Arthritis: Results from an International Expert Panel Using a Novel Online Process. The Journal of Rheumatology, 42(9), 1548–1555. // Barber CE, Smith A, Esdaile JM, Barnabe C, Martin LO, Faris P, et al. Best practices for cardiovascular disease prevention in rheumatoid arthritis: a systematic review of guideline recommendations and quality indicators. Arthritis Care Res 2015;67:169-79.
8 (21)	372	Communication to primary care physician about a documented high blood pressure	Process	Chronic	Diagnosis	Effective	K - Cardiovascular	Communication to PCP about a documented high blood pressure: IF a patient has RA AND has a blood pressure measure during a rheumatology clinic visit that is elevated (systolic blood pressure ≥ 140 and/or diastolic blood pressure ≥ 90), THEN the rheumatologist should recommend that it be repeated and treatment initiated or adjusted if indicated.	1. Barber, C. E. H., Marshall, D. A., Alvarez, N., Mancini, G. B. J., Lacaille, D., … Keeling, S. (2015). Development of Cardiovascular Quality Indicators for Rheumatoid Arthritis: Results from an International Expert Panel Using a Novel Online Process. The Journal of Rheumatology, 42(9), 1548–1555. // Barber CE, Smith A, Esdaile JM, Barnabe C, Martin LO, Faris P, et al. Best practices for cardiovascular disease prevention in rheumatoid arthritis: a systematic review of guideline recommendations and quality indicators. Arthritis Care Res 2015;67:169-79.
8 (21)	373	Measurement of a lipid profile	Process	Chronic	Screening and prevention	Safe	T - Endocrine/Metabolic and Nutritional	Measurement of a lipid profile: IF a patient has RA, THEN a lipid profile should be done at least once in the first 2 years after evaluation by a rheumatologist AND A) if low risk according to CV risk scores, the lipid profile should be repeated once every 5 years; OR B) if CV risk assessment suggests intermediate or high risk, then treatment according to national guidelines should be recommended	1. Barber, C. E. H., Marshall, D. A., Alvarez, N., Mancini, G. B. J., Lacaille, D., … Keeling, S. (2015). Development of Cardiovascular Quality Indicators for Rheumatoid Arthritis: Results from an International Expert Panel Using a Novel Online Process. The Journal of Rheumatology, 42(9), 1548–1555. // Barber CE, Smith A, Esdaile JM, Barnabe C, Martin LO, Faris P, et al. Best practices for cardiovascular disease prevention in rheumatoid arthritis: a systematic review of guideline recommendations and quality indicators. Arthritis Care Res 2015;67:169-79.
8 (21)	374	Screening for diabetes	Process	Chronic	Screening and prevention	Safe	T - Endocrine/Metabolic and Nutritional	Screening for diabetes: IF a patient has RA, THEN diabetes should be screened for as part of a CV risk assessment at least once within the first 2 years of evaluation by a rheumatologist and A) once every 5 years in low-risk patients or B) yearly in intermediate- or high-risk patients AND if screening is abnormal, this information should be communicated to the primary care provider for appropriate followup and management, if indicated. Note: Risk here denotes risk of diabetes and assessment of diabetes risk is described in detail in the full specifications for the quality indicators (shown in the Supplementary Table, available online at jrheum.org).	1. Barber, C. E. H., Marshall, D. A., Alvarez, N., Mancini, G. B. J., Lacaille, D., … Keeling, S. (2015). Development of Cardiovascular Quality Indicators for Rheumatoid Arthritis: Results from an International Expert Panel Using a Novel Online Process. The Journal of Rheumatology, 42(9), 1548–1555. // Barber CE, Smith A, Esdaile JM, Barnabe C, Martin LO, Faris P, et al. Best practices for cardiovascular disease prevention in rheumatoid arthritis: a systematic review of guideline recommendations and quality indicators. Arthritis Care Res 2015;67:169-79.
8 (21)	375	Exercise	Outcome	Chronic	Screening and prevention	Patient-centered	A - General and unspecified	Exercise: IF a patient has RA, THEN physical activity goals should be discussed with their rheumatologist at least once yearly	1. Barber, C. E. H., Marshall, D. A., Alvarez, N., Mancini, G. B. J., Lacaille, D., … Keeling, S. (2015). Development of Cardiovascular Quality Indicators for Rheumatoid Arthritis: Results from an International Expert Panel Using a Novel Online Process. The Journal of Rheumatology, 42(9), 1548–1555. // Barber CE, Smith A, Esdaile JM, Barnabe C, Martin LO, Faris P, et al. Best practices for cardiovascular disease prevention in rheumatoid arthritis: a systematic review of guideline recommendations and quality indicators. Arthritis Care Res 2015;67:169-79.
8 (21)	376	Body mass index screening and lifestyle counseling	Process	Chronic	Screening and prevention	Effective	A - General and unspecified	Body mass index (BMI) screening and lifestyle counseling: A) IF a patient has RA, THEN their BMI should be documented at least once every year, AND B) if they are overweight or obese according to national guidelines, they should be counseled to modify their lifestyle.	1. Barber, C. E. H., Marshall, D. A., Alvarez, N., Mancini, G. B. J., Lacaille, D., … Keeling, S. (2015). Development of Cardiovascular Quality Indicators for Rheumatoid Arthritis: Results from an International Expert Panel Using a Novel Online Process. The Journal of Rheumatology, 42(9), 1548–1555. // Barber CE, Smith A, Esdaile JM, Barnabe C, Martin LO, Faris P, et al. Best practices for cardiovascular disease prevention in rheumatoid arthritis: a systematic review of guideline recommendations and quality indicators. Arthritis Care Res 2015;67:169-79.
8 (21)	377	Minimizing corticosteroid usage	Process	Chronic	Treatment	Safe	A - General and unspecified	Minimizing corticosteroid usage: IF a patient with RA is taking oral corticosteroids, THEN there should be evidence of intent to taper the corticosteroids or reduce to the lowest possible dose.	1. Barber, C. E. H., Marshall, D. A., Alvarez, N., Mancini, G. B. J., Lacaille, D., … Keeling, S. (2015). Development of Cardiovascular Quality Indicators for Rheumatoid Arthritis: Results from an International Expert Panel Using a Novel Online Process. The Journal of Rheumatology, 42(9), 1548–1555. // Barber CE, Smith A, Esdaile JM, Barnabe C, Martin LO, Faris P, et al. Best practices for cardiovascular disease prevention in rheumatoid arthritis: a systematic review of guideline recommendations and quality indicators. Arthritis Care Res 2015;67:169-79.
8 (21)	378	Communication about risks/benefits of antiinflammatories in patients at high risk of cardiovascular events	Process	Chronic	Screening and prevention	Effective	K - Cardiovascular	Communication about risks/benefits of antiinflammatories in patients at high risk of CV events: IF a patient has RA, AND has established CV disease OR is at intermediate or high CV risk AND is taking a nonsteroidal antiinflammatory drug (or COX-2 inhibitor), THEN a discussion about the potential CV risks should occur and be documented	1. Barber, C. E. H., Marshall, D. A., Alvarez, N., Mancini, G. B. J., Lacaille, D., … Keeling, S. (2015). Development of Cardiovascular Quality Indicators for Rheumatoid Arthritis: Results from an International Expert Panel Using a Novel Online Process. The Journal of Rheumatology, 42(9), 1548–1555. // Barber CE, Smith A, Esdaile JM, Barnabe C, Martin LO, Faris P, et al. Best practices for cardiovascular disease prevention in rheumatoid arthritis: a systematic review of guideline recommendations and quality indicators. Arthritis Care Res 2015;67:169-79.
10 (31)	379	Coronary heart disease: blood pressure achievement	Outcome	Chronic	Diagnosis	Effective	K - Cardiovascular	Coronary heart disease: blood pressure achievement	Boeckxstaens, P., Smedt, D. D., Maeseneer, J. D., Annemans, L., & Willems, S. (2011). The equity dimension in evaluations of the quality and outcomes framework: A systematic review. BMC Health Services Research, 11(1). // Crawley D, Ng A, Mainous AG, Majeed A, Millett C: Impact of pay for performance on quality of chronic disease management by social class group in England. J R Soc Med 2009, 102(3):103-107.
10 (31)	380	Coronary heart disease: Cholesterol achievement	Outcome	Chronic	Diagnosis	Effective	K - Cardiovascular	Coronary heart disease: Cholesterol achievement	Boeckxstaens, P., Smedt, D. D., Maeseneer, J. D., Annemans, L., & Willems, S. (2011). The equity dimension in evaluations of the quality and outcomes framework: A systematic review. BMC Health Services Research, 11(1). // Crawley D, Ng A, Mainous AG, Majeed A, Millett C: Impact of pay for performance on quality of chronic disease management by social class group in England. J R Soc Med 2009, 102(3):103-107.
10 (31)	381	Coronary heart disease: Use of antihypertensives	Process	Chronic	Treatment	Safe	K - Cardiovascular	Coronary heart disease: Use of antihypertensives	Boeckxstaens, P., Smedt, D. D., Maeseneer, J. D., Annemans, L., & Willems, S. (2011). The equity dimension in evaluations of the quality and outcomes framework: A systematic review. BMC Health Services Research, 11(1). // Crawley D, Ng A, Mainous AG, Majeed A, Millett C: Impact of pay for performance on quality of chronic disease management by social class group in England. J R Soc Med 2009, 102(3):103-107.
10 (31)	382	Coronary heart disease: Use of lipid lowering drugs	Process	Chronic	Treatment	Safe	K - Cardiovascular	Coronary heart disease: Use of lipid lowering drugs	Boeckxstaens, P., Smedt, D. D., Maeseneer, J. D., Annemans, L., & Willems, S. (2011). The equity dimension in evaluations of the quality and outcomes framework: A systematic review. BMC Health Services Research, 11(1). // Crawley D, Ng A, Mainous AG, Majeed A, Millett C: Impact of pay for performance on quality of chronic disease management by social class group in England. J R Soc Med 2009, 102(3):103-107.
10 (13)	383	Diabetes patients: blood pressure measured	Process	Chronic	Screening and prevention	Effective	T - Endocrine/Metabolic and Nutritional	Diabetes patients: blood pressure measured	Boeckxstaens, P., Smedt, D. D., Maeseneer, J. D., Annemans, L., & Willems, S. (2011). The equity dimension in evaluations of the quality and outcomes framework: A systematic review. BMC Health Services Research, 11(1). // Millett C, Bottle A, Ng A, Curcin V, Molokhia M, Saxena S, Majeed A: Pay for perfomance and the quality of diabetes management in individuals with and without co-morbid medical conditions. J R Soc Med 2009, 102(9):369-377.
10 (13)	384	Diabetes patients: recorded smoking status	Process	Chronic	Screening and prevention	Patient-centered	T - Endocrine/Metabolic and Nutritional	Diabetes patients: recorded smoking status	Boeckxstaens, P., Smedt, D. D., Maeseneer, J. D., Annemans, L., & Willems, S. (2011). The equity dimension in evaluations of the quality and outcomes framework: A systematic review. BMC Health Services Research, 11(1). // Millett C, Bottle A, Ng A, Curcin V, Molokhia M, Saxena S, Majeed A: Pay for perfomance and the quality of diabetes management in individuals with and without co-morbid medical conditions. J R Soc Med 2009, 102(9):369-377.

10 (13)	385	Diabetes patients: Smoking cessation advice	Process	Chronic	Screening and prevention	Patient-centered	T - Endocrine/Metabolic and Nutritional	Diabetes patients: Smoking cessation advice	Boeckxstaens, P., Smedt, D. D., Maeseneer, J. D., Annemans, L., & Willems, S. (2011). The equity dimension in evaluations of the quality and outcomes framework: A systematic review. BMC Health Services Research, 11(1). // Millett C, Bottle A, Ng A, Curcin V, Molokhia M, Saxena S, Majeed A: Pay for perfomance and the quality of diabetes management in individuals with and without co-morbid medical conditions. J R Soc Med 2009, 102(9):369-377.
10 (13)	386	Diabetes patients: smoking prevalence	Outcome	Chronic	Diagnosis	Effective	T - Endocrine/Metabolic and Nutritional	Diabetes patients: smoking prevalence	Boeckxstaens, P., Smedt, D. D., Maeseneer, J. D., Annemans, L., & Willems, S. (2011). The equity dimension in evaluations of the quality and outcomes framework: A systematic review. BMC Health Services Research, 11(1). // Millett C, Bottle A, Ng A, Curcin V, Molokhia M, Saxena S, Majeed A: Pay for perfomance and the quality of diabetes management in individuals with and without co-morbid medical conditions. J R Soc Med 2009, 102(9):369-377.
10 (13)	387	Diabetes: Cholesterol achievement	Outcome	Chronic	Diagnosis	Effective	T - Endocrine/Metabolic and Nutritional	Diabetes: Cholesterol achievement	Boeckxstaens, P., Smedt, D. D., Maeseneer, J. D., Annemans, L., & Willems, S. (2011). The equity dimension in evaluations of the quality and outcomes framework: A systematic review. BMC Health Services Research, 11(1). // Millett C, Bottle A, Ng A, Curcin V, Molokhia M, Saxena S, Majeed A: Pay for perfomance and the quality of diabetes management in individuals with and without co-morbid medical conditions. J R Soc Med 2009, 102(9):369-377.
10 (13)	388	Diabetes: HbA1c achievement	Outcome	Chronic	Diagnosis	Effective	T - Endocrine/Metabolic and Nutritional	Diabetes: HbA1c achievement	Boeckxstaens, P., Smedt, D. D., Maeseneer, J. D., Annemans, L., & Willems, S. (2011). The equity dimension in evaluations of the quality and outcomes framework: A systematic review. BMC Health Services Research, 11(1). // Millett C, Bottle A, Ng A, Curcin V, Molokhia M, Saxena S, Majeed A: Pay for perfomance and the quality of diabetes management in individuals with and without co-morbid medical conditions. J R Soc Med 2009, 102(9):369-377.
10 (13)	389	Diabetes: Use of antihypertensives	Process	Chronic	Treatment	Effective	T - Endocrine/Metabolic and Nutritional	Diabetes: Use of antihypertensives	Boeckxstaens, P., Smedt, D. D., Maeseneer, J. D., Annemans, L., & Willems, S. (2011). The equity dimension in evaluations of the quality and outcomes framework: A systematic review. BMC Health Services Research, 11(1). // Millett C, Bottle A, Ng A, Curcin V, Molokhia M, Saxena S, Majeed A: Pay for perfomance and the quality of diabetes management in individuals with and without co-morbid medical conditions. J R Soc Med 2009, 102(9):369-377.
10 (13)	390	Diabetes: Use of lipid lowering drugs	Process	Chronic	Treatment	Effective	T - Endocrine/Metabolic and Nutritional	Diabetes: Use of lipid lowering drugs	Boeckxstaens, P., Smedt, D. D., Maeseneer, J. D., Annemans, L., & Willems, S. (2011). The equity dimension in evaluations of the quality and outcomes framework: A systematic review. BMC Health Services Research, 11(1). // Millett C, Bottle A, Ng A, Curcin V, Molokhia M, Saxena S, Majeed A: Pay for perfomance and the quality of diabetes management in individuals with and without co-morbid medical conditions. J R Soc Med 2009, 102(9):369-377.
10 (13)	391	Diabetes: Use of oral hypoglycaemic agents	Process	Chronic	Treatment	Effective	T - Endocrine/Metabolic and Nutritional	Diabetes: Use of oral hypoglycaemic agents	Boeckxstaens, P., Smedt, D. D., Maeseneer, J. D., Annemans, L., & Willems, S. (2011). The equity dimension in evaluations of the quality and outcomes framework: A systematic review. BMC Health Services Research, 11(1). // Millett C, Bottle A, Ng A, Curcin V, Molokhia M, Saxena S, Majeed A: Pay for perfomance and the quality of diabetes management in individuals with and without co-morbid medical conditions. J R Soc Med 2009, 102(9):369-377.
10 (13)	392	Hypertension: Proportion of patients with hypertension, with at least one record of Body Mass Index in the last 12 months.	Process	Chronic	Follow up and continuity	Effective	K - Cardiovascular	Hypertension: Proportion of patients with hypertension, with at least one record of Body Mass Index in the last 12 months.	Boeckxstaens, P., Smedt, D. D., Maeseneer, J. D., Annemans, L., & Willems, S. (2011). The equity dimension in evaluations of the quality and outcomes framework: A systematic review. BMC Health Services Research, 11(1). // Millett C, Bottle A, Ng A, Curcin V, Molokhia M, Saxena S, Majeed A: Pay for perfomance and the quality of diabetes management in individuals with and without co-morbid medical conditions. J R Soc Med 2009, 102(9):369-377.
10	393	Hypertension: Use of antihypertensives	Process	Chronic	Treatment	Effective	K - Cardiovascular	Hypertension: Use of antihypertensives	Boeckxstaens, P., Smedt, D. D., Maeseneer, J. D., Annemans, L., & Willems, S. (2011). The equity dimension in evaluations of the quality and outcomes framework: A systematic review. BMC Health Services Research, 11(1).
10 (33)	394	Cerebrovascular Disease: MRI/CT scan	Process	Chronic	Diagnosis	Effective	K - Cardiovascular	Cerebrovascular Disease: MRI/CT scan	Boeckxstaens, P., Smedt, D. D., Maeseneer, J. D., Annemans, L., & Willems, S. (2011). The equity dimension in evaluations of the quality and outcomes framework: A systematic review. BMC Health Services Research, 11(1). // Mc Govern MB Massoud, Taylor Michael, Williams David, Hannaford Philip, Lefevre Karen, Simpson Colin: The effect of the UK incentive based contract on the management of patients with coronary hearth disease in primary care. Family Practice 2008, 25:33-39.
10 (33)	395	Cerebrovascular Disease: Smoking status and Smokers given advice	Process	Chronic	Screening and prevention	Effective	K - Cardiovascular	Cerebrovascular Disease: Smoking status and Smokers given advice	Boeckxstaens, P., Smedt, D. D., Maeseneer, J. D., Annemans, L., & Willems, S. (2011). The equity dimension in evaluations of the quality and outcomes framework: A systematic review. BMC Health Services Research, 11(1). // Mc Govern MB Massoud, Taylor Michael, Williams David, Hannaford Philip, Lefevre Karen, Simpson Colin: The effect of the UK incentive based contract on the management of patients with coronary hearth disease in primary care. Family Practice 2008, 25:33-39.
10 (33)	396	Cerebrovascular Disease: Cholesterol with measurement ≤5 mmol/L	Process	Chronic	Screening and prevention	Effective	K - Cardiovascular	Cerebrovascular Disease: Cholesterol with measurement ≤5 mmol/L	Boeckxstaens, P., Smedt, D. D., Maeseneer, J. D., Annemans, L., & Willems, S. (2011). The equity dimension in evaluations of the quality and outcomes framework: A systematic review. BMC Health Services Research, 11(1). // Mc Govern MB Massoud, Taylor Michael, Williams David, Hannaford Philip, Lefevre Karen, Simpson Colin: The effect of the UK incentive based contract on the management of patients with coronary hearth disease in primary care. Family Practice 2008, 25:33-39.
10 (33)	397	Cerebrovascular Disease: Blood pressure controlled	Process	Chronic	Screening and prevention	Effective	K - Cardiovascular	Cerebrovascular Disease: Blood pressure controlled	Boeckxstaens, P., Smedt, D. D., Maeseneer, J. D., Annemans, L., & Willems, S. (2011). The equity dimension in evaluations of the quality and outcomes framework: A systematic review. BMC Health Services Research, 11(1). // Mc Govern MB Massoud, Taylor Michael, Williams David, Hannaford Philip, Lefevre Karen, Simpson Colin: The effect of the UK incentive based contract on the management of patients with coronary hearth disease in primary care. Family Practice 2008, 25:33-39.
10 (33)	398	Cerebrovascular Disease: Antiplatelet or anticoagulant therapy usage	Process	Chronic	Treatment	Effective	K - Cardiovascular	Cerebrovascular Disease: Antiplatelet or anticoagulant therapy usage	Boeckxstaens, P., Smedt, D. D., Maeseneer, J. D., Annemans, L., & Willems, S. (2011). The equity dimension in evaluations of the quality and outcomes framework: A systematic review. BMC Health Services Research, 11(1). // Mc Govern MB Massoud, Taylor Michael, Williams David, Hannaford Philip, Lefevre Karen, Simpson Colin: The effect of the UK incentive based contract on the management of patients with coronary hearth disease in primary care. Family Practice 2008, 25:33-39.
10 (33)	399	Cerebrovascular Disease: Flu vaccination recorded	Process	Chronic	Screening and prevention	Safe	K - Cardiovascular	Cerebrovascular Disease: Flu vaccination recorded	Boeckxstaens, P., Smedt, D. D., Maeseneer, J. D., Annemans, L., & Willems, S. (2011). The equity dimension in evaluations of the quality and outcomes framework: A systematic review. BMC Health Services Research, 11(1). // Mc Govern MB Massoud, Taylor Michael, Williams David, Hannaford Philip, Lefevre Karen, Simpson Colin: The effect of the UK incentive based contract on the management of patients with coronary hearth disease in primary care. Family Practice 2008, 25:33-39.
10 (33)	400	Cerebrovascular Disease: Body mass index	Process	Chronic	Screening and prevention	Safe	K - Cardiovascular	Cerebrovascular Disease: Body mass index	Boeckxstaens, P., Smedt, D. D., Maeseneer, J. D., Annemans, L., & Willems, S. (2011). The equity dimension in evaluations of the quality and outcomes framework: A systematic review. BMC Health Services Research, 11(1). // Mc Govern MB Massoud, Taylor Michael, Williams David, Hannaford Philip, Lefevre Karen, Simpson Colin: The effect of the UK incentive based contract on the management of patients with coronary hearth disease in primary care. Family Practice 2008, 25:33-39.
11	401	Primary Care Visits for Asthma	Process	Chronic	Follow up and continuity	Equitable	R - Respiratory	Primary Care Visits for Asthma	To, T., Guttmann, A., Lougheed, M. D., Gershon, A. S., Dell, S. D., Stanbrook, M. B., … Fisman, D. N. (2010). Evidence-based performance indicators of primary care for asthma: a modified RAND Appropriateness Method. International Journal for Quality in Health Care, 22(6), 476–485.
11	402	Asthma Education from Certified Asthma Educator	Process	Chronic	Screening and prevention	Patient-centered	R - Respiratory	Asthma Education from Certified Asthma Educator	To, T., Guttmann, A., Lougheed, M. D., Gershon, A. S., Dell, S. D., Stanbrook, M. B., … Fisman, D. N. (2010). Evidence-based performance indicators of primary care for asthma: a modified RAND Appropriateness Method. International Journal for Quality in Health Care, 22(6), 476–485.
11	403	Pulmonary Function Monitoring	Process	Chronic	Follow up and continuity	Effective	R - Respiratory	Pulmonary Function Monitoring	To, T., Guttmann, A., Lougheed, M. D., Gershon, A. S., Dell, S. D., Stanbrook, M. B., … Fisman, D. N. (2010). Evidence-based performance indicators of primary care for asthma: a modified RAND Appropriateness Method. International Journal for Quality in Health Care, 22(6), 476–485.
11	404	Asthma Control Monitoring	Process	Chronic	Follow up and continuity	Effective	R - Respiratory	Asthma Control Monitoring	To, T., Guttmann, A., Lougheed, M. D., Gershon, A. S., Dell, S. D., Stanbrook, M. B., … Fisman, D. N. (2010). Evidence-based performance indicators of primary care for asthma: a modified RAND Appropriateness Method. International Journal for Quality in Health Care, 22(6), 476–485.
11	405	Controller Medication - Overall use	Process	Chronic	Treatment	Effective	R - Respiratory	Controller Medication - Overall use	To, T., Guttmann, A., Lougheed, M. D., Gershon, A. S., Dell, S. D., Stanbrook, M. B., … Fisman, D. N. (2010). Evidence-based performance indicators of primary care for asthma: a modified RAND Appropriateness Method. International Journal for Quality in Health Care, 22(6), 476–485.
11	406	Controller Medication - Prescriptions	Process	Chronic	Treatment	Effective	R - Respiratory	Controller Medication - Prescriptions	To, T., Guttmann, A., Lougheed, M. D., Gershon, A. S., Dell, S. D., Stanbrook, M. B., … Fisman, D. N. (2010). Evidence-based performance indicators of primary care for asthma: a modified RAND Appropriateness Method. International Journal for Quality in Health Care, 22(6), 476–485.
11	407	Asthma Control - Overall	Process	Chronic	Treatment	Effective	R - Respiratory	Asthma Control - Overall	To, T., Guttmann, A., Lougheed, M. D., Gershon, A. S., Dell, S. D., Stanbrook, M. B., … Fisman, D. N. (2010). Evidence-based performance indicators of primary care for asthma: a modified RAND Appropriateness Method. International Journal for Quality in Health Care, 22(6), 476–485.
11	408	Asthma Control - Symptom-free Days	Outcome	Chronic	Treatment	Effective	R - Respiratory	Asthma Control - Symptom-free Days	To, T., Guttmann, A., Lougheed, M. D., Gershon, A. S., Dell, S. D., Stanbrook, M. B., … Fisman, D. N. (2010). Evidence-based performance indicators of primary care for asthma: a modified RAND Appropriateness Method. International Journal for Quality in Health Care, 22(6), 476–485.
11	409	Asthma Control - Absenteeism from Work/School for Asthma	Outcome	Chronic	Treatment	Effective	R - Respiratory	Asthma Control - Absenteeism from Work/School for Asthma	To, T., Guttmann, A., Lougheed, M. D., Gershon, A. S., Dell, S. D., Stanbrook, M. B., … Fisman, D. N. (2010). Evidence-based performance indicators of primary care for asthma: a modified RAND Appropriateness Method. International Journal for Quality in Health Care, 22(6), 476–485.
11	410	Pulmonary Function Test	Process	Chronic	Diagnosis	Effective	R - Respiratory	Pulmonary Function Test	To, T., Guttmann, A., Lougheed, M. D., Gershon, A. S., Dell, S. D., Stanbrook, M. B., … Fisman, D. N. (2010). Evidence-based performance indicators of primary care for asthma: a modified RAND Appropriateness Method. International Journal for Quality in Health Care, 22(6), 476–485.
11	411	Asthma: Received Action Plan	Process	Chronic	Treatment	Effective	R - Respiratory	Asthma: Received Action Plan	To, T., Guttmann, A., Lougheed, M. D., Gershon, A. S., Dell, S. D., Stanbrook, M. B., … Fisman, D. N. (2010). Evidence-based performance indicators of primary care for asthma: a modified RAND Appropriateness Method. International Journal for Quality in Health Care, 22(6), 476–485.
11	412	Asthma: Reliever Medication Use	Process	Chronic	Treatment	Effective	R - Respiratory	Asthma: Reliever Medication Use	To, T., Guttmann, A., Lougheed, M. D., Gershon, A. S., Dell, S. D., Stanbrook, M. B., … Fisman, D. N. (2010). Evidence-based performance indicators of primary care for asthma: a modified RAND Appropriateness Method. International Journal for Quality in Health Care, 22(6), 476–485.
11	413	Asthma: Seen by a Specialist	Process	Chronic	Follow up and continuity	Effective	R - Respiratory	Asthma: Seen by a Specialist	To, T., Guttmann, A., Lougheed, M. D., Gershon, A. S., Dell, S. D., Stanbrook, M. B., … Fisman, D. N. (2010). Evidence-based performance indicators of primary care for asthma: a modified RAND Appropriateness Method. International Journal for Quality in Health Care, 22(6), 476–485.
11	414	Asthma: Smoking Cessation	Outcome	Chronic	Follow up and continuity	Effective	R - Respiratory	Asthma: Smoking Cessation	To, T., Guttmann, A., Lougheed, M. D., Gershon, A. S., Dell, S. D., Stanbrook, M. B., … Fisman, D. N. (2010). Evidence-based performance indicators of primary care for asthma: a modified RAND Appropriateness Method. International Journal for Quality in Health Care, 22(6), 476–485.
11	415	Asthma Exacerbations	Outcome	All	Follow up and continuity	Effective	R - Respiratory	Asthma Exacerbations	To, T., Guttmann, A., Lougheed, M. D., Gershon, A. S., Dell, S. D., Stanbrook, M. B., … Fisman, D. N. (2010). Evidence-based performance indicators of primary care for asthma: a modified RAND Appropriateness Method. International Journal for Quality in Health Care, 22(6), 476–485.
11	416	Asthma: Weight Reduction	Outcome	Chronic	Follow up and continuity	Effective	R - Respiratory	Weight Reduction	To, T., Guttmann, A., Lougheed, M. D., Gershon, A. S., Dell, S. D., Stanbrook, M. B., … Fisman, D. N. (2010). Evidence-based performance indicators of primary care for asthma: a modified RAND Appropriateness Method. International Journal for Quality in Health Care, 22(6), 476–485.
11	417	Referred to Asthma Education Program/Asthma Centre	Process	Chronic	Follow up and continuity	Effective	R - Respiratory	Referred to Asthma Education Program/Asthma Centre	To, T., Guttmann, A., Lougheed, M. D., Gershon, A. S., Dell, S. D., Stanbrook, M. B., … Fisman, D. N. (2010). Evidence-based performance indicators of primary care for asthma: a modified RAND Appropriateness Method. International Journal for Quality in Health Care, 22(6), 476–485.
11	418	Patient Quality of Life	Outcome	Chronic	Follow up and continuity	Patient-centered	R - Respiratory	Patient Quality of Life	To, T., Guttmann, A., Lougheed, M. D., Gershon, A. S., Dell, S. D., Stanbrook, M. B., … Fisman, D. N. (2010). Evidence-based performance indicators of primary care for asthma: a modified RAND Appropriateness Method. International Journal for Quality in Health Care, 22(6), 476–485.
11	419	Inhaler Technique Monitoring	Process	Chronic	Follow up and continuity	Effective	R - Respiratory	Inhaler Technique Monitoring	To, T., Guttmann, A., Lougheed, M. D., Gershon, A. S., Dell, S. D., Stanbrook, M. B., … Fisman, D. N. (2010). Evidence-based performance indicators of primary care for asthma: a modified RAND Appropriateness Method. International Journal for Quality in Health Care, 22(6), 476–485.
11	420	Routine Care Provider for asthma	Process	Chronic	Follow up and continuity	Patient-centered	R - Respiratory	Routine Care Provider for asthma	To, T., Guttmann, A., Lougheed, M. D., Gershon, A. S., Dell, S. D., Stanbrook, M. B., … Fisman, D. N. (2010). Evidence-based performance indicators of primary care for asthma: a modified RAND Appropriateness Method. International Journal for Quality in Health Care, 22(6), 476–485.
15	421	Allopurinol prescribed at a dose of >200mg/day to patients with renal impairment (estimated glomerular filtration rate <30)	Process	Chronic	Treatment	Safe	U - Urological	Allopurinol prescribed at a dose of >200mg/day to patients with renal impairment (estimated glomerular filtration rate <30)	Spencer, R., Bell, B., Avery, A. J., Gookey, G., & Campbell, S. M. (2014). Identification of an updated set of prescribing-safety indicators for GPs. British Journal of General Practice, 64(621), e181–e190.
15	422	Amitriptyline at dose >75mg prescribed to a patient with heart failure, arrhythmia, heart block. or postural hypotension	Process	Chronic	Treatment	Safe	K - Cardiovascular	Amitriptyline at dose >75mg prescribed to a patient with heart failure, arrhythmia, heart block. or postural hypotension	Spencer, R., Bell, B., Avery, A. J., Gookey, G., & Campbell, S. M. (2014). Identification of an updated set of prescribing-safety indicators for GPs. British Journal of General Practice, 64(621), e181–e190.
15	423	Bupropion prescribed to a patient with epilepsy	Process	Chronic	Treatment	Safe	P - Psychological	Bupropion prescribed to a patient with epilepsy	Spencer, R., Bell, B., Avery, A. J., Gookey, G., & Campbell, S. M. (2014). Identification of an updated set of prescribing-safety indicators for GPs. British Journal of General Practice, 64(621), e181–e190.
15	424	Glitazone prescribed to patient with heart failure	Process	Chronic	Treatment	Safe	K - Cardiovascular	Glitazone prescribed to patient with heart failure	Spencer, R., Bell, B., Avery, A. J., Gookey, G., & Campbell, S. M. (2014). Identification of an updated set of prescribing-safety indicators for GPs. British Journal of General Practice, 64(621), e181–e190.
15	425	Metformin prescribed to a patient with renal impairment where the estimated glomerular filtration rate is ≤30ml/min	Process	Chronic	Treatment	Safe	T - Endocrine/Metabolic and Nutritional / U - Urological	Metformin prescribed to a patient with renal impairment where the estimated glomerular filtration rate is ≤30ml/min	Spencer, R., Bell, B., Avery, A. J., Gookey, G., & Campbell, S. M. (2014). Identification of an updated set of prescribing-safety indicators for GPs. British Journal of General Practice, 64(621), e181–e190.
15	426	Modified-release potassium supplements prescribed to a patient with a history of peptic ulcer disease	Process	Chronic	Treatment	Safe	D - Digestive	Modified-release potassium supplements prescribed to a patient with a history of peptic ulcer disease	Spencer, R., Bell, B., Avery, A. J., Gookey, G., & Campbell, S. M. (2014). Identification of an updated set of prescribing-safety indicators for GPs. British Journal of General Practice, 64(621), e181–e190.
15	427	Prescription of a beta-blocker to a patient with asthma	Process	Chronic	Treatment	Safe	R - Respiratory	Prescription of a beta-blocker to a patient with asthma	Spencer, R., Bell, B., Avery, A. J., Gookey, G., & Campbell, S. M. (2014). Identification of an updated set of prescribing-safety indicators for GPs. British Journal of General Practice, 64(621), e181–e190.
15	428	Prescription of a long-acting beta-2 agonist inhaler to a patient with asthma who is not also prescribed an inhaled corticosteroid	Process	Chronic	Treatment	Safe	R - Respiratory	Prescription of a long-acting beta-2 agonist inhaler to a patient with asthma who is not also prescribed an inhaled corticosteroid	Spencer, R., Bell, B., Avery, A. J., Gookey, G., & Campbell, S. M. (2014). Identification of an updated set of prescribing-safety indicators for GPs. British Journal of General Practice, 64(621), e181–e190.
15	429	Prescription of a Non-steroid anti-inflammatory drug in a patient with chronic renal failure with an estimated glomerular filtration rate <45	Process	Chronic	Treatment	Safe	U - Urological	Prescription of a Non-steroid anti-inflammatory drug in a patient with chronic renal failure with an estimated glomerular filtration rate <45	Spencer, R., Bell, B., Avery, A. J., Gookey, G., & Campbell, S. M. (2014). Identification of an updated set of prescribing-safety indicators for GPs. British Journal of General Practice, 64(621), e181–e190.
15	430	Prescription of a Non-steroid anti-inflammatory drug in a patient with heart failure	Process	Chronic	Treatment	Safe	K - Cardiovascular	Prescription of a Non-steroid anti-inflammatory drug in a patient with heart failure	Spencer, R., Bell, B., Avery, A. J., Gookey, G., & Campbell, S. M. (2014). Identification of an updated set of prescribing-safety indicators for GPs. British Journal of General Practice, 64(621), e181–e190.
15	431	Prescription of a potassium salt or potassium-sparing diuretic (excluding aldosterone antagonists) to a patient who is also receiving an angiotensin-converting-enzyme inhibitor or angiotensin II receptor antagonist	Process	Chronic	Treatment	Safe	K - Cardiovascular	Prescription of a potassium salt or potassium-sparing diuretic (excluding aldosterone antagonists) to a patient who is also receiving an angiotensin-converting-enzyme inhibitor or angiotensin II receptor antagonist	Spencer, R., Bell, B., Avery, A. J., Gookey, G., & Campbell, S. M. (2014). Identification of an updated set of prescribing-safety indicators for GPs. British Journal of General Practice, 64(621), e181–e190.
15	432	Prescription of digoxin at a dose >125 mg daily in a patient with renal impairment (for example, chronic kidney disease 3 or worse)	Process	Chronic	Treatment	Safe	K - Cardiovascular / U - Urological	Prescription of digoxin at a dose >125 mg daily in a patient with renal impairment (for example, chronic kidney disease 3 or worse)	Spencer, R., Bell, B., Avery, A. J., Gookey, G., & Campbell, S. M. (2014). Identification of an updated set of prescribing-safety indicators for GPs. British Journal of General Practice, 64(621), e181–e190.
15	433	Prescription of digoxin at a dose of greater than 125 mg daily for a patient with heart failure who is in sinus rhythm	Process	Chronic	Treatment	Safe	K - Cardiovascular	Prescription of digoxin at a dose of greater than 125 mg daily for a patient with heart failure who is in sinus rhythm	Spencer, R., Bell, B., Avery, A. J., Gookey, G., & Campbell, S. M. (2014). Identification of an updated set of prescribing-safety indicators for GPs. British Journal of General Practice, 64(621), e181–e190.
15	434	Prescription of diltiazem or verapamil in a patient with heart failure	Process	Chronic	Treatment	Safe	K - Cardiovascular	Prescription of diltiazem or verapamil in a patient with heart failure	Spencer, R., Bell, B., Avery, A. J., Gookey, G., & Campbell, S. M. (2014). Identification of an updated set of prescribing-safety indicators for GPs. British Journal of General Practice, 64(621), e181–e190.
15	435	Prescription of mefloquine to a patient with a history of convulsions	Process	Chronic	Treatment	Safe	N - Neurological	Prescription of mefloquine to a patient with a history of convulsions	Spencer, R., Bell, B., Avery, A. J., Gookey, G., & Campbell, S. M. (2014). Identification of an updated set of prescribing-safety indicators for GPs. British Journal of General Practice, 64(621), e181–e190.
15	436	Use of a hypothyroid agent without monitoring relevant thyroid function tests within 2–4 months of initiation or dosage change and at least every 15 months thereafter	Process	Chronic	Treatment	Safe	T - Endocrine/Metabolic and Nutritional	Use of a hypothyroid agent without monitoring relevant thyroid function tests within 2–4 months of initiation or dosage change and at least every 15 months thereafter	Spencer, R., Bell, B., Avery, A. J., Gookey, G., & Campbell, S. M. (2014). Identification of an updated set of prescribing-safety indicators for GPs. British Journal of General Practice, 64(621), e181–e190.
16	437	Health-related QoL in patients with chronic conditions and their carers	Outcome	Chronic	Follow up and continuity	Patient-centered	A - General and unspecified	Health-related QoL in patients with chronic conditions and their carers	Olry de Labry Lima, A., García Mochón, L., & Bermúdez Tamayo, C. (2017). Identificación de indicadores de resultado en salud en atención primaria. Una revisión de revisiones sistemáticas. Revista de Calidad Asistencial, 32(5), 278–288.
16	438	Hypertension: control of blood pressure level in high risk patients	Process	Chronic	Follow up and continuity	Effective	K - Cardiovascular	Hypertension: control of blood pressure level in high risk patients	Olry de Labry Lima, A., García Mochón, L., & Bermúdez Tamayo, C. (2017). Identificación de indicadores de resultado en salud en atención primaria. Una revisión de revisiones sistemáticas. Revista de Calidad Asistencial, 32(5), 278–288.
16	439	Lipid control in patients with ischaemic heart disease	Process	Chronic	Follow up and continuity	Effective	T - Endocrine/Metabolic and Nutritional	Lipid control in patients with ischaemic heart disease	Olry de Labry Lima, A., García Mochón, L., & Bermúdez Tamayo, C. (2017). Identificación de indicadores de resultado en salud en atención primaria. Una revisión de revisiones sistemáticas. Revista de Calidad Asistencial, 32(5), 278–288.
16	440	Patients with chronic conditions attended in primary care according to stratification profiles of their health status	Outcome	Chronic	Screening and prevention	Effective	A - General and unspecified	Patients with chronic conditions attended in primary care according to stratification profiles of their health status	Olry de Labry Lima, A., García Mochón, L., & Bermúdez Tamayo, C. (2017). Identificación de indicadores de resultado en salud en atención primaria. Una revisión de revisiones sistemáticas. Revista de Calidad Asistencial, 32(5), 278–288.
16	441	Patients with chronic conditions attended in primary health care by a social services professional (sanitary action)	Outcome	Chronic	Screening and prevention	Patient-centered	A - General and unspecified	Patients with chronic conditions attended in primary health care by a social services professional (sanitary action)	Olry de Labry Lima, A., García Mochón, L., & Bermúdez Tamayo, C. (2017). Identificación de indicadores de resultado en salud en atención primaria. Una revisión de revisiones sistemáticas. Revista de Calidad Asistencial, 32(5), 278–288.
16	442	Patients with multiple chronic conditions and medications attended in primary care	Outcome	Chronic	Screening and Prevention	Patient-centered	A - General and unspecified	Patients with multiple chronic conditions and medications attended in primary care	Olry de Labry Lima, A., García Mochón, L., & Bermúdez Tamayo, C. (2017). Identificación de indicadores de resultado en salud en atención primaria. Una revisión de revisiones sistemáticas. Revista de Calidad Asistencial, 32(5), 278–288.
16	443	Prevention of pressure ulcers in patients included in the chronic dependent patients care program	Outcome	Chronic	Screening and prevention	Safe	A - General and unspecified	Prevention of pressure ulcers in patients included in the chronic dependent patients care program	Olry de Labry Lima, A., García Mochón, L., & Bermúdez Tamayo, C. (2017). Identificación de indicadores de resultado en salud en atención primaria. Una revisión de revisiones sistemáticas. Revista de Calidad Asistencial, 32(5), 278–288.
16	444	Primary care use by patients with chronic conditions	Process	Chronic	Screening and prevention	Patient-centered	A - General and unspecified	Primary care use by patients with chronic conditions	Olry de Labry Lima, A., García Mochón, L., & Bermúdez Tamayo, C. (2017). Identificación de indicadores de resultado en salud en atención primaria. Una revisión de revisiones sistemáticas. Revista de Calidad Asistencial, 32(5), 278–288.
18	445	Comprehensive physical health assessment with appropriate advice	Process	Chronic	Screening and prevention	Effective	A - General and unspecified	Comprehensive physical health assessment with appropriate advice	Kronenberg, C., Doran, T., Goddard, M., Kendrick, T., Gilbody, S., Dare, C. R., … Jacobs, R. (2017). Identifying primary care quality indicators for people with serious mental illness: a systematic review. British Journal of General Practice, 67(661), e519–e530.

18	446	Counselling on physical activity and/or nutrition for those with documented elevated BMI	Process	Chronic	Screening and prevention	Effective	A - General and unspecified	Counselling on physical activity and/or nutrition for those with documented elevated BMI	Kronenberg, C., Doran, T., Goddard, M., Kendrick, T., Gilbody, S., Dare, C. R., … Jacobs, R. (2017). Identifying primary care quality indicators for people with serious mental illness: a systematic review. British Journal of General Practice, 67(661), e519–e530.
18	447	Diabetes and cholesterol monitoring for people with schizophrenia and diabetes	Process	Chronic	Follow up and continuity	Effective	T - Endocrine/Metabolic and Nutritional	Diabetes and cholesterol monitoring for people with schizophrenia and diabetes	Kronenberg, C., Doran, T., Goddard, M., Kendrick, T., Gilbody, S., Dare, C. R., … Jacobs, R. (2017). Identifying primary care quality indicators for people with serious mental illness: a systematic review. British Journal of General Practice, 67(661), e519–e530.
18	448	Diabetes monitoring for people with diabetes and schizophrenia	Process	Chronic	Follow up and continuity	Effective	T - Endocrine/Metabolic and Nutritional	Diabetes monitoring for people with diabetes and schizophrenia	Kronenberg, C., Doran, T., Goddard, M., Kendrick, T., Gilbody, S., Dare, C. R., … Jacobs, R. (2017). Identifying primary care quality indicators for people with serious mental illness: a systematic review. British Journal of General Practice, 67(661), e519–e530.
18	449	Diabetes screening for people who are using antipsychotic medications	Process	Chronic	Screening and prevention	Effective	T - Endocrine/Metabolic and Nutritional	Diabetes screening for people who are using antipsychotic medications	Kronenberg, C., Doran, T., Goddard, M., Kendrick, T., Gilbody, S., Dare, C. R., … Jacobs, R. (2017). Identifying primary care quality indicators for people with serious mental illness: a systematic review. British Journal of General Practice, 67(661), e519–e530.
18	450	Foot exam for patients with serious mental illness who have diabetes	Process	Chronic	Screening and prevention	Effective	T - Endocrine/Metabolic and Nutritional	Foot exam for patients with serious mental illness who have diabetes	Kronenberg, C., Doran, T., Goddard, M., Kendrick, T., Gilbody, S., Dare, C. R., … Jacobs, R. (2017). Identifying primary care quality indicators for people with serious mental illness: a systematic review. British Journal of General Practice, 67(661), e519–e530.
18	451	Hypertension counselling: patients with hypertension who received education services related to hypertension, nutrition, cooking, physical activity, or exercise	Process	Chronic	Screening and prevention	Patient-centered	K - Cardiovascular	Hypertension counselling: patients with hypertension who received education services related to hypertension, nutrition, cooking, physical activity, or exercise	Kronenberg, C., Doran, T., Goddard, M., Kendrick, T., Gilbody, S., Dare, C. R., … Jacobs, R. (2017). Identifying primary care quality indicators for people with serious mental illness: a systematic review. British Journal of General Practice, 67(661), e519–e530.
18	452	Hypertension: recording and monitoring patients with hypertension and high blood cholesterol (LDL)	Process	Chronic	Follow up and continuity	Effective	K - Cardiovascular	Hypertension: recording and monitoring patients with hypertension and high blood cholesterol (LDL)	Kronenberg, C., Doran, T., Goddard, M., Kendrick, T., Gilbody, S., Dare, C. R., … Jacobs, R. (2017). Identifying primary care quality indicators for people with serious mental illness: a systematic review. British Journal of General Practice, 67(661), e519–e530.
18	453	Medical attention for nephropathy	Process	Chronic	Screening and prevention	Safe	U - Urological	Medical attention for nephropathy	Kronenberg, C., Doran, T., Goddard, M., Kendrick, T., Gilbody, S., Dare, C. R., … Jacobs, R. (2017). Identifying primary care quality indicators for people with serious mental illness: a systematic review. British Journal of General Practice, 67(661), e519–e530.
18	454	Patients with diabetes who received psychoeducation related to weight (BMI), diabetes (blood glucose levels)	Process	Chronic	Screening and prevention	Patient-centered	T - Endocrine/Metabolic and Nutritional / P - Psychological	Patients with diabetes who received psychoeducation related to weight (BMI), diabetes (blood glucose levels)	Kronenberg, C., Doran, T., Goddard, M., Kendrick, T., Gilbody, S., Dare, C. R., … Jacobs, R. (2017). Identifying primary care quality indicators for people with serious mental illness: a systematic review. British Journal of General Practice, 67(661), e519–e530.
18	455	Proportion of patients who have an increased blood glucose level	Outcome	Chronic	Diagnosis	Effective	T - Endocrine/Metabolic and Nutritional / P - Psychological	Proportion of patients who have an increased blood glucose level	Kronenberg, C., Doran, T., Goddard, M., Kendrick, T., Gilbody, S., Dare, C. R., … Jacobs, R. (2017). Identifying primary care quality indicators for people with serious mental illness: a systematic review. British Journal of General Practice, 67(661), e519–e530.
18	456	Proportion of patients who have an increased blood pressure	Outcome	Chronic	Diagnosis	Effective	K - Cardiovascular	Proportion of patients who have an increased blood pressure	Kronenberg, C., Doran, T., Goddard, M., Kendrick, T., Gilbody, S., Dare, C. R., … Jacobs, R. (2017). Identifying primary care quality indicators for people with serious mental illness: a systematic review. British Journal of General Practice, 67(661), e519–e530.
18	457	Proportion of patients who have increased level of blood lipids	Outcome	Chronic	Diagnosis	Effective	T - Endocrine/Metabolic and Nutritional / P - Psychological	Proportion of patients who have increased level of blood lipids	Kronenberg, C., Doran, T., Goddard, M., Kendrick, T., Gilbody, S., Dare, C. R., … Jacobs, R. (2017). Identifying primary care quality indicators for people with serious mental illness: a systematic review. British Journal of General Practice, 67(661), e519–e530.
18	458	Proportion of patients who have low levels of glycosylated haemoglobin	Outcome	Chronic	Diagnosis	Effective	T - Endocrine/Metabolic and Nutritional / P - Psychological	Proportion of patients who have low levels of glycosylated haemoglobin	Kronenberg, C., Doran, T., Goddard, M., Kendrick, T., Gilbody, S., Dare, C. R., … Jacobs, R. (2017). Identifying primary care quality indicators for people with serious mental illness: a systematic review. British Journal of General Practice, 67(661), e519–e530.
18	459	Proportion with increased BMI / abdominal waist line	Outcome	Chronic	Diagnosis	Effective	T - Endocrine/Metabolic and Nutritional / P - Psychological	Proportion with increased BMI/abdominal waistline	Kronenberg, C., Doran, T., Goddard, M., Kendrick, T., Gilbody, S., Dare, C. R., … Jacobs, R. (2017). Identifying primary care quality indicators for people with serious mental illness: a systematic review. British Journal of General Practice, 67(661), e519–e530.
18	460	Retinal exam for patients with serious mental illness who have diabetes	Process	Chronic	Screening and prevention	Effective	T - Endocrine/Metabolic and Nutritional / P - Psychological	Retinal exam for patients with serious mental illness who have diabetes	Kronenberg, C., Doran, T., Goddard, M., Kendrick, T., Gilbody, S., Dare, C. R., … Jacobs, R. (2017). Identifying primary care quality indicators for people with serious mental illness: a systematic review. British Journal of General Practice, 67(661), e519–e530.
18	461	Weight management/BMI monitoring	Process	Chronic	Screening and prevention	Effective	T - Endocrine/Metabolic and Nutritional / P - Psychological	Weight management/BMI monitoring	Kronenberg, C., Doran, T., Goddard, M., Kendrick, T., Gilbody, S., Dare, C. R., … Jacobs, R. (2017). Identifying primary care quality indicators for people with serious mental illness: a systematic review. British Journal of General Practice, 67(661), e519–e530.
19	462	Asthma: Adequate technique for childhood asthma	Process	Chronic	Treatment	Effective	R - Respiratory	Asthma: Adequate technique for childhood asthma	Ruiz-Canela-Cáceres, J., Aquino-Llinares, N., Sánchez-Diaz, J. M., García-Gestoso, M. L., de Jaime-Revuelta, M. E., & Praena-Crespo, M. (2015). Indicators for childhood asthma in Spain, using the Rand method. Allergologia et Immunopathologia, 43(2), 147–156.
19	463	Asthma: Asthmatic patients with screening for depression	Process	Chronic	Screening and prevention	Safe	R - Respiratory	Asthma: Asthmatic patients with screening for depression	Ruiz-Canela-Cáceres, J., Aquino-Llinares, N., Sánchez-Diaz, J. M., García-Gestoso, M. L., de Jaime-Revuelta, M. E., & Praena-Crespo, M. (2015). Indicators for childhood asthma in Spain, using the Rand method. Allergologia et Immunopathologia, 43(2), 147–156.
19	464	Asthma: Basic medication has been prescribed for childhood asthma	Process	Chronic	Treatment	Effective	R - Respiratory	Asthma: Basic medication has been prescribed for childhood asthma	Ruiz-Canela-Cáceres, J., Aquino-Llinares, N., Sánchez-Diaz, J. M., García-Gestoso, M. L., de Jaime-Revuelta, M. E., & Praena-Crespo, M. (2015). Indicators for childhood asthma in Spain, using the Rand method. Allergologia et Immunopathologia, 43(2), 147–156.
19	465	Asthma: Children assessed by a nurse in the past six months	Process	Chronic	Screening and prevention	Effective	R - Respiratory	Asthma: Children assessed by a nurse in the past six months	Ruiz-Canela-Cáceres, J., Aquino-Llinares, N., Sánchez-Diaz, J. M., García-Gestoso, M. L., de Jaime-Revuelta, M. E., & Praena-Crespo, M. (2015). Indicators for childhood asthma in Spain, using the Rand method. Allergologia et Immunopathologia, 43(2), 147–156.
19	466	Asthma: Children over eight years of age with variability measurements	Process	Chronic	Diagnosis	Effective	R - Respiratory	Asthma: Children over eight years of age with variability measurements	Ruiz-Canela-Cáceres, J., Aquino-Llinares, N., Sánchez-Diaz, J. M., García-Gestoso, M. L., de Jaime-Revuelta, M. E., & Praena-Crespo, M. (2015). Indicators for childhood asthma in Spain, using the Rand method. Allergologia et Immunopathologia, 43(2), 147–156.
19	467	Asthma: Children with a severity classification for their asthma at least once a year	Process	Chronic	Diagnosis	Effective	R - Respiratory	Asthma: Children with a severity classification for their asthma at least once a year	Ruiz-Canela-Cáceres, J., Aquino-Llinares, N., Sánchez-Diaz, J. M., García-Gestoso, M. L., de Jaime-Revuelta, M. E., & Praena-Crespo, M. (2015). Indicators for childhood asthma in Spain, using the Rand method. Allergologia et Immunopathologia, 43(2), 147–156.
19	468	Asthma: Days free of symptoms in the two previous weeks	Outcome	Chronic	Follow up and continuity	Effective	R - Respiratory	Asthma: Days free of symptoms in the two previous weeks	Ruiz-Canela-Cáceres, J., Aquino-Llinares, N., Sánchez-Diaz, J. M., García-Gestoso, M. L., de Jaime-Revuelta, M. E., & Praena-Crespo, M. (2015). Indicators for childhood asthma in Spain, using the Rand method. Allergologia et Immunopathologia, 43(2), 147–156.
19	469	Asthma: Educational objectives in the last 12 months	Outcome	Chronic	Follow up and continuity	Patient-centered	R - Respiratory	Asthma: Educational objectives in the last 12 months	Ruiz-Canela-Cáceres, J., Aquino-Llinares, N., Sánchez-Diaz, J. M., García-Gestoso, M. L., de Jaime-Revuelta, M. E., & Praena-Crespo, M. (2015). Indicators for childhood asthma in Spain, using the Rand method. Allergologia et Immunopathologia, 43(2), 147–156.
19	470	Asthma: Alternative prescription is adequate	Process	Chronic	Treatment	Effective	R - Respiratory	Asthma: Alternative prescription is adequate	Ruiz-Canela-Cáceres, J., Aquino-Llinares, N., Sánchez-Diaz, J. M., García-Gestoso, M. L., de Jaime-Revuelta, M. E., & Praena-Crespo, M. (2015). Indicators for childhood asthma in Spain, using the Rand method. Allergologia et Immunopathologia, 43(2), 147–156.
19	471	Asthma: first choice indicates inhaled corticoids (correct prescription) for childhood asthma	Process	Chronic	Treatment	Effective	R - Respiratory	Asthma: first choice indicates inhaled corticoids (correctprescription) for childhood asthma	Ruiz-Canela-Cáceres, J., Aquino-Llinares, N., Sánchez-Diaz, J. M., García-Gestoso, M. L., de Jaime-Revuelta, M. E., & Praena-Crespo, M. (2015). Indicators for childhood asthma in Spain, using the Rand method. Allergologia et Immunopathologia, 43(2), 147–156.
19	472	Asthma: Number of inhaled corticoid dosages in one year	Process	Chronic	Treatment	Safe	R - Respiratory	Asthma: Number of inhaled corticoiddosages in one year	Ruiz-Canela-Cáceres, J., Aquino-Llinares, N., Sánchez-Diaz, J. M., García-Gestoso, M. L., de Jaime-Revuelta, M. E., & Praena-Crespo, M. (2015). Indicators for childhood asthma in Spain, using the Rand method. Allergologia et Immunopathologia, 43(2), 147–156.
19	473	Asthma: Number of school days missed in the past four weeks	Outcome	Chronic	Follow up and continuity	Patient-centered	R - Respiratory	Asthma: Number of school days missedin the past four weeks	Ruiz-Canela-Cáceres, J., Aquino-Llinares, N., Sánchez-Diaz, J. M., García-Gestoso, M. L., de Jaime-Revuelta, M. E., & Praena-Crespo, M. (2015). Indicators for childhood asthma in Spain, using the Rand method. Allergologia et Immunopathologia, 43(2), 147–156.
19	474	Asthma: Patient undergoing continuous basic treatment with four or more visits per year	Outcome	Chronic	Treatment	Effective	R - Respiratory	Asthma: Patient undergoing continuous basic treatment with four or more visits per year	Ruiz-Canela-Cáceres, J., Aquino-Llinares, N., Sánchez-Diaz, J. M., García-Gestoso, M. L., de Jaime-Revuelta, M. E., & Praena-Crespo, M. (2015). Indicators for childhood asthma in Spain, using the Rand method. Allergologia et Immunopathologia, 43(2), 147–156.
19	475	Asthma: Patient with two or more rounds of corticoids due to an attack in three months and with no prescribed basic treatment	Outcome	Chronic	Treatment	Safe	R - Respiratory	Asthma: Patient with two or more rounds of corticoids due to an attack in three months and with no prescribed basic treatment	Ruiz-Canela-Cáceres, J., Aquino-Llinares, N., Sánchez-Diaz, J. M., García-Gestoso, M. L., de Jaime-Revuelta, M. E., & Praena-Crespo, M. (2015). Indicators for childhood asthma in Spain, using the Rand method. Allergologia et Immunopathologia, 43(2), 147–156.
19	476	Asthma: Percentage of children assessed for day-time/night-time symptoms	Process	Chronic	Screening and prevention	Patient-centered	R - Respiratory	Asthma: Percentage of children assessed for day-time/night-time symptoms	Ruiz-Canela-Cáceres, J., Aquino-Llinares, N., Sánchez-Diaz, J. M., García-Gestoso, M. L., de Jaime-Revuelta, M. E., & Praena-Crespo, M. (2015). Indicators for childhood asthma in Spain, using the Rand method. Allergologia et Immunopathologia, 43(2), 147–156.
19	477	Asthma: Percentage of children assessed for treatment, with check-ups in less than three weeks	Process	Chronic	Screening and prevention	Effective	R - Respiratory	Asthma: Percentage of children assessed for treatment, with check-ups in less than three weeks	Ruiz-Canela-Cáceres, J., Aquino-Llinares, N., Sánchez-Diaz, J. M., García-Gestoso, M. L., de Jaime-Revuelta, M. E., & Praena-Crespo, M. (2015). Indicators for childhood asthma in Spain, using the Rand method. Allergologia et Immunopathologia, 43(2), 147–156.
19	478	Asthma: Percentage of children visiting a specialist due to moderate-severe asthma (one year)	Outcome	Chronic	Follow up and continuity	Effective	R - Respiratory	Asthma: Percentage of children visiting aspecialist due to moderate- severe asthma (one year)	Ruiz-Canela-Cáceres, J., Aquino-Llinares, N., Sánchez-Diaz, J. M., García-Gestoso, M. L., de Jaime-Revuelta, M. E., & Praena-Crespo, M. (2015). Indicators for childhood asthma in Spain, using the Rand method. Allergologia et Immunopathologia, 43(2), 147–156.
19	479	Asthma: Percentage of children with auscultation in crisis	Process	Chronic	Follow up and continuity	Effective	R - Respiratory	Asthma: Percentage of children with auscultation in crisis	Ruiz-Canela-Cáceres, J., Aquino-Llinares, N., Sánchez-Diaz, J. M., García-Gestoso, M. L., de Jaime-Revuelta, M. E., & Praena-Crespo, M. (2015). Indicators for childhood asthma in Spain, using the Rand method. Allergologia et Immunopathologia, 43(2), 147–156.
19	480	Asthma: Percentage of children with follow-up from the same doctor for at least 80% of their visits	Process	Chronic	Follow up and continuity	Effective	R - Respiratory	Asthma: Percentage of children with follow-up from the same doctor for at least 80% of their visits	Ruiz-Canela-Cáceres, J., Aquino-Llinares, N., Sánchez-Diaz, J. M., García-Gestoso, M. L., de Jaime-Revuelta, M. E., & Praena-Crespo, M. (2015). Indicators for childhood asthma in Spain, using the Rand method. Allergologia et Immunopathologia, 43(2), 147–156.
19	481	Asthma: Percentage of children with moderately severe asthma, with personal improvement score	Outcome	Chronic	Follow up and continuity	Effective	R - Respiratory	Asthma: Percentage of children with moderately severe asthma, with personal improvement score	Ruiz-Canela-Cáceres, J., Aquino-Llinares, N., Sánchez-Diaz, J. M., García-Gestoso, M. L., de Jaime-Revuelta, M. E., & Praena-Crespo, M. (2015). Indicators for childhood asthma in Spain, using the Rand method. Allergologia et Immunopathologia, 43(2), 147–156.
19	482	Asthma: Percentage of children with one visit per year	Process	Chronic	Follow up and continuity	Effective	R - Respiratory	Asthma: Percentage of children with one visit to GP per year	Ruiz-Canela-Cáceres, J., Aquino-Llinares, N., Sánchez-Diaz, J. M., García-Gestoso, M. L., de Jaime-Revuelta, M. E., & Praena-Crespo, M. (2015). Indicators for childhood asthma in Spain, using the Rand method. Allergologia et Immunopathologia, 43(2), 147–156.
19	483	Asthma: Percentage of children with two established visits and active asthma	Process	Chronic	Follow up and continuity	Effective	R - Respiratory	Asthma: Percentage of children with two established visits and active asthma	Ruiz-Canela-Cáceres, J., Aquino-Llinares, N., Sánchez-Diaz, J. M., García-Gestoso, M. L., de Jaime-Revuelta, M. E., & Praena-Crespo, M. (2015). Indicators for childhood asthma in Spain, using the Rand method. Allergologia et Immunopathologia, 43(2), 147–156.
19	484	Asthma: Percentage of children with peak flow usage and self-management frequency	Process	Chronic	Follow up and continuity	Effective	R - Respiratory	Asthma: Percentage of children with peak flow usage and self-management frequency	Ruiz-Canela-Cáceres, J., Aquino-Llinares, N., Sánchez-Diaz, J. M., García-Gestoso, M. L., de Jaime-Revuelta, M. E., & Praena-Crespo, M. (2015). Indicators for childhood asthma in Spain, using the Rand method. Allergologia et Immunopathologia, 43(2), 147–156.
19	485	Asthma: Percentage of moderate-severe with fluvaccine the previous year	Process	Chronic	Follow up and continuity	Effective	R - Respiratory	Asthma: Percentage of patients with moderate-severe asthma and fluvaccine the previous year	Ruiz-Canela-Cáceres, J., Aquino-Llinares, N., Sánchez-Diaz, J. M., García-Gestoso, M. L., de Jaime-Revuelta, M. E., & Praena-Crespo, M. (2015). Indicators for childhood asthma in Spain, using the Rand method. Allergologia et Immunopathologia, 43(2), 147–156.
19	486	Asthma: Percentage of patients diagnosed with asthma using FT, spirometry ormethacholine or exercise	Process	Chronic	Follow up and continuity	Effective	R - Respiratory	Asthma: Percentage of patients diagnosed with asthma using FT, spirometry ormethacholine or exercise	Ruiz-Canela-Cáceres, J., Aquino-Llinares, N., Sánchez-Diaz, J. M., García-Gestoso, M. L., de Jaime-Revuelta, M. E., & Praena-Crespo, M. (2015). Indicators for childhood asthma in Spain, using the Rand method. Allergologia et Immunopathologia, 43(2), 147–156.
19	487	Asthma: Percentage of patients indicating their exposure to tobacco smoke	Process	Chronic	Follow up and continuity	Patient-centered	R - Respiratory	Asthma: Percentage of patients indicating their exposure to tobacco smoke	Ruiz-Canela-Cáceres, J., Aquino-Llinares, N., Sánchez-Diaz, J. M., García-Gestoso, M. L., de Jaime-Revuelta, M. E., & Praena-Crespo, M. (2015). Indicators for childhood asthma in Spain, using the Rand method. Allergologia et Immunopathologia, 43(2), 147–156.
19	488	Asthma: Percentage of patients tested for allergies	Process	Chronic	Follow up and continuity	Effective	R - Respiratory	Asthma: Percentage of patients tested for allergies	Ruiz-Canela-Cáceres, J., Aquino-Llinares, N., Sánchez-Diaz, J. M., García-Gestoso, M. L., de Jaime-Revuelta, M. E., & Praena-Crespo, M. (2015). Indicators for childhood asthma in Spain, using the Rand method. Allergologia et Immunopathologia, 43(2), 147–156.
19	489	Asthma: Percentage of patients with self-management objectives in 12 months	Process	Chronic	Follow up and continuity	Patient-centered	R - Respiratory	Asthma: Percentage of patients with self-management objectives in 12 months	Ruiz-Canela-Cáceres, J., Aquino-Llinares, N., Sánchez-Diaz, J. M., García-Gestoso, M. L., de Jaime-Revuelta, M. E., & Praena-Crespo, M. (2015). Indicators for childhood asthma in Spain, using the Rand method. Allergologia et Immunopathologia, 43(2), 147–156.
19	490	Asthma: Routine Care Provider	Process	Chronic	Follow up and continuity	Patient-centered	R - Respiratory	Asthma: Routine Care Provider	Ruiz-Canela-Cáceres, J., Aquino-Llinares, N., Sánchez-Diaz, J. M., García-Gestoso, M. L., de Jaime-Revuelta, M. E., & Praena-Crespo, M. (2015). Indicators for childhood asthma in Spain, using the Rand method. Allergologia et Immunopathologia, 43(2), 147–156.
19	491	Asthma: Spirometry in the past 12 months indicated in medical records	Process	Chronic	Follow up and continuity	Efficient	R - Respiratory	Asthma: Spirometry in the past 12 monthsindicated in medical records	Ruiz-Canela-Cáceres, J., Aquino-Llinares, N., Sánchez-Diaz, J. M., García-Gestoso, M. L., de Jaime-Revuelta, M. E., & Praena-Crespo, M. (2015). Indicators for childhood asthma in Spain, using the Rand method. Allergologia et Immunopathologia, 43(2), 147–156.
19	492	Asthma: Technical inhaler verification for childhood asthma	Process	Chronic	Follow up and continuity	Effective	R - Respiratory	Asthma: Technical inhaler verification for childhood asthma	Ruiz-Canela-Cáceres, J., Aquino-Llinares, N., Sánchez-Diaz, J. M., García-Gestoso, M. L., de Jaime-Revuelta, M. E., & Praena-Crespo, M. (2015). Indicators for childhood asthma in Spain, using the Rand method. Allergologia et Immunopathologia, 43(2), 147–156.
19	493	Asthma: Theophylline and crisis	Process	Chronic	Treatment	Safe	R - Respiratory	Asthma: Theophylline and crisis	Ruiz-Canela-Cáceres, J., Aquino-Llinares, N., Sánchez-Diaz, J. M., García-Gestoso, M. L., de Jaime-Revuelta, M. E., & Praena-Crespo, M. (2015). Indicators for childhood asthma in Spain, using the Rand method. Allergologia et Immunopathologia, 43(2), 147–156.
19	494	Asthma: Undergoing high dosage treatment and growth not verified at least once a year	Process	Chronic	Treatment	Safe	R - Respiratory	Asthma: Undergoing high dosage treatment and growth not verifiedat least once a year	Ruiz-Canela-Cáceres, J., Aquino-Llinares, N., Sánchez-Diaz, J. M., García-Gestoso, M. L., de Jaime-Revuelta, M. E., & Praena-Crespo, M. (2015). Indicators for childhood asthma in Spain, using the Rand method. Allergologia et Immunopathologia, 43(2), 147–156.
19	495	Asthma: Usage of beta2 on demand and not used in basic treatment	Process	Chronic	Treatment	Safe	R - Respiratory	Asthma: Usage of beta2 on demand andnot used in basic treatment	Ruiz-Canela-Cáceres, J., Aquino-Llinares, N., Sánchez-Diaz, J. M., García-Gestoso, M. L., de Jaime-Revuelta, M. E., & Praena-Crespo, M. (2015). Indicators for childhood asthma in Spain, using the Rand method. Allergologia et Immunopathologia, 43(2), 147–156.
19	496	Asthma: Use of anti-asthma products-inhalers or oral for childhood asthma	Process	Chronic	Treatment	Safe	R - Respiratory	Asthma: Use of anti-asthma products-inhalers or oral for childhood asthma	Ruiz-Canela-Cáceres, J., Aquino-Llinares, N., Sánchez-Diaz, J. M., García-Gestoso, M. L., de Jaime-Revuelta, M. E., & Praena-Crespo, M. (2015). Indicators for childhood asthma in Spain, using the Rand method. Allergologia et Immunopathologia, 43(2), 147–156.
19	497	Asthma: Use of oral corticoids if FEV1 isless than 70% following crisis	Process	Chronic	Treatment	Safe	R - Respiratory	Asthma: Use of oral corticoids if FEV1 isless than 70% following crisis	Ruiz-Canela-Cáceres, J., Aquino-Llinares, N., Sánchez-Diaz, J. M., García-Gestoso, M. L., de Jaime-Revuelta, M. E., & Praena-Crespo, M. (2015). Indicators for childhood asthma in Spain, using the Rand method. Allergologia et Immunopathologia, 43(2), 147–156.
19	498	Asthma: Uses spacer chamber for childhood asthma	Process	Chronic	Treatment	Effective	R - Respiratory	Asthma: Uses spacer chamber for childhood asthma	Ruiz-Canela-Cáceres, J., Aquino-Llinares, N., Sánchez-Diaz, J. M., García-Gestoso, M. L., de Jaime-Revuelta, M. E., & Praena-Crespo, M. (2015). Indicators for childhood asthma in Spain, using the Rand method. Allergologia et Immunopathologia, 43(2), 147–156.
19	499	Asthma: Using trial continuous oral corticoids inhaled at high dosage	Process	Chronic	Treatment	Safe	R - Respiratory	Asthma: Using trial continuous oral corticoids inhaled at high dosage	Ruiz-Canela-Cáceres, J., Aquino-Llinares, N., Sánchez-Diaz, J. M., García-Gestoso, M. L., de Jaime-Revuelta, M. E., & Praena-Crespo, M. (2015). Indicators for childhood asthma in Spain, using the Rand method. Allergologia et Immunopathologia, 43(2), 147–156.
20	500	Chronic Kidney Disease: Adherence to treatment	Process	Chronic	Treatment	Patient-centered	U - Urological	Chronic Kidney Disease: Adherence to treatment	Smits, K. P. J., Sidorenkov, G., Bilo, H. J. G., Bouma, M., Navis, G. J., & Denig, P. (2016). Process quality indicators for chronic kidney disease risk management: a systematic literature review. International Journal of Clinical Practice, 70(10), 861–869.
20	501	Chronic Kidney Disease: Inappropriate dosages	Process	Chronic	Treatment	Safe	U - Urological	Chronic Kidney Disease: Inappropriate dosages	Smits, K. P. J., Sidorenkov, G., Bilo, H. J. G., Bouma, M., Navis, G. J., & Denig, P. (2016). Process quality indicators for chronic kidney disease risk management: a systematic literature review. International Journal of Clinical Practice, 70(10), 861–869.
20	502	Chronic Kidney Disease: Inappropriate drugs	Process	Chronic	Treatment	Safe	U - Urological	Chronic Kidney Disease: Inappropriate drugs	Smits, K. P. J., Sidorenkov, G., Bilo, H. J. G., Bouma, M., Navis, G. J., & Denig, P. (2016). Process quality indicators for chronic kidney disease risk management: a systematic literature review. International Journal of Clinical Practice, 70(10), 861–869.
20	503	Chronic Kidney Disease: Monitoring of anaemia	Process	Chronic	Follow up and continuity	Effective	U - Urological	Chronic Kidney Disease: Monitoring of anaemia	Smits, K. P. J., Sidorenkov, G., Bilo, H. J. G., Bouma, M., Navis, G. J., & Denig, P. (2016). Process quality indicators for chronic kidney disease risk management: a systematic literature review. International Journal of Clinical Practice, 70(10), 861–869.
20	504	Chronic Kidney Disease: Monitoring of Blood pressure	Process	Chronic	Follow up and continuity	Effective	U - Urological	Chronic Kidney Disease: Monitoring of Blood pressure	Smits, K. P. J., Sidorenkov, G., Bilo, H. J. G., Bouma, M., Navis, G. J., & Denig, P. (2016). Process quality indicators for chronic kidney disease risk management: a systematic literature review. International Journal of Clinical Practice, 70(10), 861–869.
20	505	Chronic Kidney Disease: Monitoring of body composition	Process	Chronic	Follow up and continuity	Effective	U - Urological	Chronic Kidney Disease: Monitoring of body composition	Smits, K. P. J., Sidorenkov, G., Bilo, H. J. G., Bouma, M., Navis, G. J., & Denig, P. (2016). Process quality indicators for chronic kidney disease risk management: a systematic literature review. International Journal of Clinical Practice, 70(10), 861–869.
20	506	Chronic Kidney Disease: Monitoring of diet	Process	Chronic	Follow up and continuity	Effective	U - Urological	Chronic Kidney Disease: Monitoring of diet	Smits, K. P. J., Sidorenkov, G., Bilo, H. J. G., Bouma, M., Navis, G. J., & Denig, P. (2016). Process quality indicators for chronic kidney disease risk management: a systematic literature review. International Journal of Clinical Practice, 70(10), 861–869.
20	507	Chronic Kidney Disease: Monitoring of HbA1c	Process	Chronic	Follow up and continuity	Effective	U - Urological	Chronic Kidney Disease: Monitoring of HbA1c	Smits, K. P. J., Sidorenkov, G., Bilo, H. J. G., Bouma, M., Navis, G. J., & Denig, P. (2016). Process quality indicators for chronic kidney disease risk management: a systematic literature review. International Journal of Clinical Practice, 70(10), 861–869.
20	508	Chronic Kidney Disease: Monitoring of kidney function	Process	Chronic	Follow up and continuity	Effective	U - Urological	Chronic Kidney Disease: Monitoring of kidney function	Smits, K. P. J., Sidorenkov, G., Bilo, H. J. G., Bouma, M., Navis, G. J., & Denig, P. (2016). Process quality indicators for chronic kidney disease risk management: a systematic literature review. International Journal of Clinical Practice, 70(10), 861–869.
20	509	Chronic Kidney Disease: Monitoring of lipid levels	Process	Chronic	Follow up and continuity	Effective	U - Urological	Chronic Kidney Disease: Monitoring of lipid levels	Smits, K. P. J., Sidorenkov, G., Bilo, H. J. G., Bouma, M., Navis, G. J., & Denig, P. (2016). Process quality indicators for chronic kidney disease risk management: a systematic literature review. International Journal of Clinical Practice, 70(10), 861–869.
20	510	Chronic Kidney Disease: Monitoring of mineral and bone disorder	Process	Chronic	Follow up and continuity	Effective	U - Urological	Chronic Kidney Disease: Monitoring of mineral and bone disorder	Smits, K. P. J., Sidorenkov, G., Bilo, H. J. G., Bouma, M., Navis, G. J., & Denig, P. (2016). Process quality indicators for chronic kidney disease risk management: a systematic literature review. International Journal of Clinical Practice, 70(10), 861–869.
20	511	Chronic Kidney Disease: Monitoring of plasma C-reactive protein/homocysteine	Process	Chronic	Follow up and continuity	Effective	U - Urological	Chronic Kidney Disease: Monitoring of plasma C-reactive protein/homocysteine	Smits, K. P. J., Sidorenkov, G., Bilo, H. J. G., Bouma, M., Navis, G. J., & Denig, P. (2016). Process quality indicators for chronic kidney disease risk management: a systematic literature review. International Journal of Clinical Practice, 70(10), 861–869.

20	512	Chronic Kidney Disease: Referrals to Nephrologist	Process	Chronic	Follow up and continuity	Effective	U - Urological	Chronic Kidney Disease: Referrals to Nephrologist	Smits, K. P. J., Sidorenkov, G., Bilo, H. J. G., Bouma, M., Navis, G. J., & Denig, P. (2016). Process quality indicators for chronic kidney disease risk management: a systematic literature review. International Journal of Clinical Practice, 70(10), 861–869.
20	513	Chronic Kidney Disease: Referrals to other specialities	Process	Chronic	Follow up and continuity	Effective	U - Urological	Chronic Kidney Disease: Referrals to other specialities	Smits, K. P. J., Sidorenkov, G., Bilo, H. J. G., Bouma, M., Navis, G. J., & Denig, P. (2016). Process quality indicators for chronic kidney disease risk management: a systematic literature review. International Journal of Clinical Practice, 70(10), 861–869.
20	514	Chronic Kidney Disease: Treatment of anaemia	Process	Chronic	Treatment	Safe	U - Urological	Chronic Kidney Disease: Treatment of anaemia	Smits, K. P. J., Sidorenkov, G., Bilo, H. J. G., Bouma, M., Navis, G. J., & Denig, P. (2016). Process quality indicators for chronic kidney disease risk management: a systematic literature review. International Journal of Clinical Practice, 70(10), 861–869.
20	515	Chronic Kidney Disease: Treatment of mineral bone disease	Process	Chronic	Treatment	Safe	U - Urological	Chronic Kidney Disease: Treatment of mineral bone disease	Smits, K. P. J., Sidorenkov, G., Bilo, H. J. G., Bouma, M., Navis, G. J., & Denig, P. (2016). Process quality indicators for chronic kidney disease risk management: a systematic literature review. International Journal of Clinical Practice, 70(10), 861–869.
20	516	Chronic Kidney Disease: Use of angiotensin-converting-enzyme inhibitor or angiotensin II receptor blockers	Process	Chronic	Treatment	Effective	U - Urological	Chronic Kidney Disease: Use of angiotensin-converting-enzyme inhibitor or angiotensin II receptor blockers	Smits, K. P. J., Sidorenkov, G., Bilo, H. J. G., Bouma, M., Navis, G. J., & Denig, P. (2016). Process quality indicators for chronic kidney disease risk management: a systematic literature review. International Journal of Clinical Practice, 70(10), 861–869.
20	517	Chronic Kidney Disease: Use of aspirin	Process	Chronic	Treatment	Effective	U - Urological	Chronic Kidney Disease: Use of aspirin	Smits, K. P. J., Sidorenkov, G., Bilo, H. J. G., Bouma, M., Navis, G. J., & Denig, P. (2016). Process quality indicators for chronic kidney disease risk management: a systematic literature review. International Journal of Clinical Practice, 70(10), 861–869.
20	518	Chronic Kidney Disease: Use of glucose lowering drugs	Process	Chronic	Treatment	Effective	U - Urological	Chronic Kidney Disease: Use of glucose lowering drugs	Smits, K. P. J., Sidorenkov, G., Bilo, H. J. G., Bouma, M., Navis, G. J., & Denig, P. (2016). Process quality indicators for chronic kidney disease risk management: a systematic literature review. International Journal of Clinical Practice, 70(10), 861–869.
20	519	Chronic Kidney Disease: Use of lipid lowering drugs	Process	Chronic	Treatment	Effective	U - Urological	Chronic Kidney Disease: Use of lipid lowering drugs	Smits, K. P. J., Sidorenkov, G., Bilo, H. J. G., Bouma, M., Navis, G. J., & Denig, P. (2016). Process quality indicators for chronic kidney disease risk management: a systematic literature review. International Journal of Clinical Practice, 70(10), 861–869.
20	520	Chronic Kidney Disease: Use of Non-steroid anti-inflammatory drugs	Process	Chronic	Treatment	Safe	U - Urological	Chronic Kidney Disease: Use of Non-steroid anti-inflammatory drugs	Smits, K. P. J., Sidorenkov, G., Bilo, H. J. G., Bouma, M., Navis, G. J., & Denig, P. (2016). Process quality indicators for chronic kidney disease risk management: a systematic literature review. International Journal of Clinical Practice, 70(10), 861–869.
20	521	Chronic Kidney Disease: Use of other antihypertensives	Process	Chronic	Treatment	Effective	U - Urological	Chronic Kidney Disease: Use of other antihypertensives	Smits, K. P. J., Sidorenkov, G., Bilo, H. J. G., Bouma, M., Navis, G. J., & Denig, P. (2016). Process quality indicators for chronic kidney disease risk management: a systematic literature review. International Journal of Clinical Practice, 70(10), 861–869.
21	522	Systemic Lupus Erythematosus: Adequate treatment of proliferative nephritis	Process	Chronic	Treatment	Effective	L - Musculoskeletal	Systemic Lupus Erythematosus: Adequate treatment of proliferative nephritis	Yazdany, J., Panopalis, P., Gillis, J. Z., Schmajuk, G., MacLean, C. H., … Wofsy, D. (2009). A quality indicator set for systemic lupus erythematosus. Arthritis & Rheumatism, 61(3), 370–377.
21	523	Systemic Lupus Erythematosus: Analytical control after initiating a new drug	Process	Chronic	Treatment	Safe	L - Musculoskeletal	Systemic Lupus Erythematosus: Analytical control after initiating a new drug	Yazdany, J., Panopalis, P., Gillis, J. Z., Schmajuk, G., MacLean, C. H., … Wofsy, D. (2009). A quality indicator set for systemic lupus erythematosus. Arthritis & Rheumatism, 61(3), 370–377.
21	524	Systemic Lupus Erythematosus: Analytical follow-up	Process	Chronic	Follow up and continuity	Effective	L - Musculoskeletal	Systemic Lupus Erythematosus: Analytical follow-up	Yazdany, J., Panopalis, P., Gillis, J. Z., Schmajuk, G., MacLean, C. H., … Wofsy, D. (2009). A quality indicator set for systemic lupus erythematosus. Arthritis & Rheumatism, 61(3), 370–377.
21	525	Systemic Lupus Erythematosus: Analytical study in patients with renal disease activity	Process	Chronic	Follow up and continuity	Effective	L - Musculoskeletal	Systemic Lupus Erythematosus: Analytical study in patients with renal disease activity	Yazdany, J., Panopalis, P., Gillis, J. Z., Schmajuk, G., MacLean, C. H., … Wofsy, D. (2009). A quality indicator set for systemic lupus erythematosus. Arthritis & Rheumatism, 61(3), 370–377.
21	526	Systemic Lupus Erythematosus: Analytical study in pregnant women	Process	Chronic	Screening and prevention	Effective	L - Musculoskeletal	Systemic Lupus Erythematosus: Analytical study in pregnant women	Yazdany, J., Panopalis, P., Gillis, J. Z., Schmajuk, G., MacLean, C. H., … Wofsy, D. (2009). A quality indicator set for systemic lupus erythematosus. Arthritis & Rheumatism, 61(3), 370–377.
21	527	Systemic Lupus Erythematosus: Bone mineral density testing in patients under corticosteroid therapy	Process	Chronic	Screening and prevention	Safe	L - Musculoskeletal	Systemic Lupus Erythematosus: Bone mineral density testing in patients under corticosteroid therapy	Yazdany, J., Panopalis, P., Gillis, J. Z., Schmajuk, G., MacLean, C. H., … Wofsy, D. (2009). A quality indicator set for systemic lupus erythematosus. Arthritis & Rheumatism, 61(3), 370–377.
21	528	Systemic Lupus Erythematosus: Calcium and vitamin D in patients under corticosteroid therapy	Process	Chronic	Treatment	Safe	L - Musculoskeletal	Systemic Lupus Erythematosus: Calcium and vitamin D in patients under corticosteroid therapy	Yazdany, J., Panopalis, P., Gillis, J. Z., Schmajuk, G., MacLean, C. H., … Wofsy, D. (2009). A quality indicator set for systemic lupus erythematosus. Arthritis & Rheumatism, 61(3), 370–377.
21	529	Systemic Lupus Erythematosus: Control of hypertension in patients with renal impairment	Process	Chronic	Follow up and continuity	Safe	L - Musculoskeletal	Systemic Lupus Erythematosus: Control of hypertension in patients with renal impairment	Yazdany, J., Panopalis, P., Gillis, J. Z., Schmajuk, G., MacLean, C. H., … Wofsy, D. (2009). A quality indicator set for systemic lupus erythematosus. Arthritis & Rheumatism, 61(3), 370–377.
21	530	Systemic Lupus Erythematosus: Diagnosis and analytic study	Process	Chronic	Diagnosis	Effective	L - Musculoskeletal	Systemic Lupus Erythematosus: Diagnosis and analytic study	Yazdany, J., Panopalis, P., Gillis, J. Z., Schmajuk, G., MacLean, C. H., … Wofsy, D. (2009). A quality indicator set for systemic lupus erythematosus. Arthritis & Rheumatism, 61(3), 370–377.
21	531	Systemic Lupus Erythematosus: Discussion about teratogenic risks of medication	Process	Chronic	Screening and prevention	Safe	L - Musculoskeletal	Systemic Lupus Erythematosus: Discussion about teratogenic risks of medication	Yazdany, J., Panopalis, P., Gillis, J. Z., Schmajuk, G., MacLean, C. H., … Wofsy, D. (2009). A quality indicator set for systemic lupus erythematosus. Arthritis & Rheumatism, 61(3), 370–377.
21	532	Systemic Lupus Erythematosus: Discussion of risks and benefits of medication	Process	Chronic	Screening and prevention	Safe	L - Musculoskeletal	Systemic Lupus Erythematosus: Discussion of risks and benefits of medication	Yazdany, J., Panopalis, P., Gillis, J. Z., Schmajuk, G., MacLean, C. H., … Wofsy, D. (2009). A quality indicator set for systemic lupus erythematosus. Arthritis & Rheumatism, 61(3), 370–377.
21	533	Systemic Lupus Erythematosus: Drug toxicity monitoring	Process	Chronic	Treatment	Safe	L - Musculoskeletal	Systemic Lupus Erythematosus: Drug toxicity monitoring	Yazdany, J., Panopalis, P., Gillis, J. Z., Schmajuk, G., MacLean, C. H., … Wofsy, D. (2009). A quality indicator set for systemic lupus erythematosus. Arthritis & Rheumatism, 61(3), 370–377.
21	534	Systemic Lupus Erythematosus: Education about sun avoidance	Process	Chronic	Screening and prevention	Patient-centered	L - Musculoskeletal	Systemic Lupus Erythematosus: Education about sun avoidance	Yazdany, J., Panopalis, P., Gillis, J. Z., Schmajuk, G., MacLean, C. H., … Wofsy, D. (2009). A quality indicator set for systemic lupus erythematosus. Arthritis & Rheumatism, 61(3), 370–377.
21	535	Systemic Lupus Erythematosus: Evaluation of cardiovascular risk factors	Process	Chronic	Follow up and continuity	Effective	L - Musculoskeletal	Systemic Lupus Erythematosus: Evaluation of cardiovascular risk factors	Yazdany, J., Panopalis, P., Gillis, J. Z., Schmajuk, G., MacLean, C. H., … Wofsy, D. (2009). A quality indicator set for systemic lupus erythematosus. Arthritis & Rheumatism, 61(3), 370–377.
21	536	Systemic Lupus Erythematosus: Influenza vaccination in imunossupressed patients	Process	Chronic	Screening and prevention	Safe	L - Musculoskeletal	Systemic Lupus Erythematosus: Influenza vaccination in imunossupressed patients	Yazdany, J., Panopalis, P., Gillis, J. Z., Schmajuk, G., MacLean, C. H., … Wofsy, D. (2009). A quality indicator set for systemic lupus erythematosus. Arthritis & Rheumatism, 61(3), 370–377.
21	537	Systemic Lupus Erythematosus: Pneumococcal vaccination in imunossupressed patients	Process	Chronic	Screening and prevention	Safe	L - Musculoskeletal	Systemic Lupus Erythematosus: Pneumococcal vaccination in imunossupressed patients	Yazdany, J., Panopalis, P., Gillis, J. Z., Schmajuk, G., MacLean, C. H., … Wofsy, D. (2009). A quality indicator set for systemic lupus erythematosus. Arthritis & Rheumatism, 61(3), 370–377.
21	538	Systemic Lupus Erythematosus: Prevention of pregnancy complications	Process	Chronic	Screening and prevention	Safe	L - Musculoskeletal	Systemic Lupus Erythematosus: Prevention of pregnancy complications	Yazdany, J., Panopalis, P., Gillis, J. Z., Schmajuk, G., MacLean, C. H., … Wofsy, D. (2009). A quality indicator set for systemic lupus erythematosus. Arthritis & Rheumatism, 61(3), 370–377.
21	539	Systemic Lupus Erythematosus: Steroid sparing	Process	Chronic	Screening and prevention	Effective	L - Musculoskeletal	Systemic Lupus Erythematosus: Steroid sparing	Yazdany, J., Panopalis, P., Gillis, J. Z., Schmajuk, G., MacLean, C. H., … Wofsy, D. (2009). A quality indicator set for systemic lupus erythematosus. Arthritis & Rheumatism, 61(3), 370–377.
21	540	Systemic Lupus Erythematosus: Treatment of osteoporosis in patients under corticosteroid therapy	Process	Chronic	Treatment	Effective	L - Musculoskeletal	Systemic Lupus Erythematosus: Treatment of osteoporosis in patients under corticosteroid therapy	Yazdany, J., Panopalis, P., Gillis, J. Z., Schmajuk, G., MacLean, C. H., … Wofsy, D. (2009). A quality indicator set for systemic lupus erythematosus. Arthritis & Rheumatism, 61(3), 370–377.
21	541	Systemic Lupus Erythematosus: Use of angiotensin-converting-enzyme inhibitor or angiotensin II receptor blockers in patients with proteinuria	Process	Chronic	Treatment	Effective	L - Musculoskeletal	Systemic Lupus Erythematosus: Use of angiotensin-converting-enzyme inhibitor or angiotensin II receptor blockers in patients with proteinuria	Yazdany, J., Panopalis, P., Gillis, J. Z., Schmajuk, G., MacLean, C. H., … Wofsy, D. (2009). A quality indicator set for systemic lupus erythematosus. Arthritis & Rheumatism, 61(3), 370–377.
24	542	Patients with chronic kidney disease in PHC	Outcome	Chronic	All	Patient-centered	U - Urological	Patients with chronic kidney disease in PHC	Fujita, K., Moles, R. J., & Chen, T. F. (2018). Quality indicators for responsible use of medicines: a systematic review. BMJ Open, 8(7), e020437.
24	543	General practice for vulnerable elders	Process	Chronic	All	Patient-centered	A - General and unspecified	General practice for vulnerable elders	Fujita, K., Moles, R. J., & Chen, T. F. (2018). Quality indicators for responsible use of medicines: a systematic review. BMJ Open, 8(7), e020437.
25	544	Quality of Life in patients with urinary Incontinence	Outcome	Chronic	Follow up and continuity	Patient-centered	U - Urological	Quality of Life in patients with urinary Incontinence	Chin WY, Lam CL, Lo SV. Quality of care of nurse-led and allied health personnel-led primary care clinics. Hong Kong medical journal = Xianggang yi xue za zhi. 2011;17(3):217-30.
25	545	Wound care clinics	Structure	Chronic	Follow up and continuity	Effective	S - Skin	Wound care clinics	Chin WY, Lam CL, Lo SV. Quality of care of nurse-led and allied health personnel-led primary care clinics. Hong Kong medical journal = Xianggang yi xue za zhi. 2011;17(3):217-30.
27	546	Register of diabetic patients under General Practice follow-up	Process	Chronic	Follow up and continuity	Effective	T - Endocrine/Metabolic and Nutritional	Register of diabetic patients under General Practice follow-up	Lake, R., Georgiou, A., Li, J., Li, L., Byrne, M., Robinson, M., & Westbrook, J. I. (2017). The quality, safety and governance of telephone triage and advice services – an overview of evidence from systematic reviews. BMC Health Services Research, 17(1).
28	547	Composite measures for DM	Process	Chronic	Follow up and continuity	Effective	T - Endocrine/Metabolic and Nutritional	Blood glucose done, HbA1c done, creatinine done, cholesterol done, urine analysis done, blood pressure done, foot examination done, full eye examination done, smoking history recorded, weight done (Score (0-40 range scale) for assessing HbA1c (5 points), lipids (5 points), microalbuminuria (MA; 5 points), blood pressure (5 points), treating MA with angiotensin-converting-enzyme inhibitor (10 points), achieving HbA1c < 8% (10 points), blood pressure < 140/90 mmHg (10 points), LDL cholesterol < 130 mg/dl (10 points))	Sidorenkov, G., Haaijer-Ruskamp, F. M., de Zeeuw, D., Bilo, H., & Denig, P. (2011). Review: Relation Between Quality-of-Care Indicators for Diabetes and Patient Outcomes: A Systematic Literature Review. Medical Care Research and Review, 68(3), 263–289.
28	548	Patients receiving aspirin and/or statin treatment when eligible	Process	Chronic	Treatment	Effective	T - Endocrine/Metabolic and Nutritional	Patients receiving aspirin and/or statin treatment when eligible	Sidorenkov, G., Haaijer-Ruskamp, F. M., de Zeeuw, D., Bilo, H., & Denig, P. (2011). Review: Relation Between Quality-of-Care Indicators for Diabetes and Patient Outcomes: A Systematic Literature Review. Medical Care Research and Review, 68(3), 263–289.
28	549	Number of medication changes for DM	Process	Chronic	Treatment	Safe	T - Endocrine/Metabolic and Nutritional	Score expressing relative number of medication changes during 1- to 1.5-year follow-up (intensity of glucose-lowering therapy)	Sidorenkov, G., Haaijer-Ruskamp, F. M., de Zeeuw, D., Bilo, H., & Denig, P. (2011). Review: Relation Between Quality-of-Care Indicators for Diabetes and Patient Outcomes: A Systematic Literature Review. Medical Care Research and Review, 68(3), 263–289.
28	550	Treatment intensification for DM	Process	Chronic	Treatment	All	T - Endocrine/Metabolic and Nutritional	Proportion of patients receiving increase in number of drug classes, dosage of at least one medication, or a switch to another medication within 3 months following an initial observation of poor control	Sidorenkov, G., Haaijer-Ruskamp, F. M., de Zeeuw, D., Bilo, H., & Denig, P. (2011). Review: Relation Between Quality-of-Care Indicators for Diabetes and Patient Outcomes: A Systematic Literature Review. Medical Care Research and Review, 68(3), 263–289.
28	551	Quartile class performance regarding annual HbA1c testing	Outcome	Chronic	Screening and prevention	Effective	T - Endocrine/Metabolic and Nutritional	Quartile class performance regarding annual HbA1c testing	Sidorenkov, G., Haaijer-Ruskamp, F. M., de Zeeuw, D., Bilo, H., & Denig, P. (2011). Review: Relation Between Quality-of-Care Indicators for Diabetes and Patient Outcomes: A Systematic Literature Review. Medical Care Research and Review, 68(3), 263–289.
29	552	Anticoagulant therapy in those with atrial fibrillation and high risk of stroke	Process	Chronic	Treatment	Effective	K - Cardiovascular	Patients using anticoagulant therapy (in those with atrial fibrillation and high risk of stroke)	Forbes, L. J., Marchand, C., Doran, T., & Peckham, S. (2017). The role of the Quality and Outcomes Framework in the care of long-term conditions: a systematic review. British Journal of General Practice, 67(664), e775–e784.
29	553	Patients newly diagnosed with diabetes referred to a structured education programme	Process	Chronic	Follow up and continuity	Effective	T - Endocrine/Metabolic and Nutritional	Patients newly diagnosed with diabetes referred to a structured education programme	Forbes, L. J., Marchand, C., Doran, T., & Peckham, S. (2017). The role of the Quality and Outcomes Framework in the care of long-term conditions: a systematic review. British Journal of General Practice, 67(664), e775–e784.
29	554	Percentage of patients 50–74 yo with confirmed osteoporosis taking bone-sparing agent	Process	Chronic	Diagnosis	Effective	L - Musculoskeletal	Percentage of patients 50–74 with confirmed osteoporosis taking bone-sparing agent	Forbes, L. J., Marchand, C., Doran, T., & Peckham, S. (2017). The role of the Quality and Outcomes Framework in the care of long-term conditions: a systematic review. British Journal of General Practice, 67(664), e775–e784.
29	555	Percentage of patients aged >75 with osteoporosis taking bone-sparing agent	Process	Chronic	Diagnosis	Effective	L - Musculoskeletal	Percentage of patients aged >75 with osteoporosis taking bone-sparing agent	Forbes, L. J., Marchand, C., Doran, T., & Peckham, S. (2017). The role of the Quality and Outcomes Framework in the care of long-term conditions: a systematic review. British Journal of General Practice, 67(664), e775–e784.
29	556	Percentage of patients with a new diagnosis of dementia with record of tests to exclude reversible cause	Process	Chronic	Diagnosis	Effective	p - Psychological	Percentage of patients with a new diagnosis of dementia with record of tests to exclude reversible cause	Forbes, L. J., Marchand, C., Doran, T., & Peckham, S. (2017). The role of the Quality and Outcomes Framework in the care of long-term conditions: a systematic review. British Journal of General Practice, 67(664), e775–e784.
29	557	Percentage of patients with asthma and measures of variability or reversibility recorded	Process	Chronic	Follow up and continuity	Effective	R - Respiratory	Percentage of patients with asthma and measures of variability or reversibility recorded	Forbes, L. J., Marchand, C., Doran, T., & Peckham, S. (2017). The role of the Quality and Outcomes Framework in the care of long-term conditions: a systematic review. British Journal of General Practice, 67(664), e775–e784.
29	558	Percentage of patients with asthma who have had control assessed	Process	Chronic	Follow up and continuity	Effective	R - Respiratory	Percentage of patients with asthma who have had control assessed	Forbes, L. J., Marchand, C., Doran, T., & Peckham, S. (2017). The role of the Quality and Outcomes Framework in the care of long-term conditions: a systematic review. British Journal of General Practice, 67(664), e775–e784.
29	559	Percentage of patients with asthma with record of smoking status	Process	Chronic	Follow up and continuity	Effective	R - Respiratory	Percentage of patients with asthma with record of smoking status	Forbes, L. J., Marchand, C., Doran, T., & Peckham, S. (2017). The role of the Quality and Outcomes Framework in the care of long-term conditions: a systematic review. British Journal of General Practice, 67(664), e775–e784.
29	560	Percentage of patients with atrial fibrillation in whom stroke risk has been assessed	Process	Chronic	Screening and prevention	Effective	K - Cardiovascular	Percentage of patients with atrial fibrillation in whom stroke risk has been assessed	Forbes, L. J., Marchand, C., Doran, T., & Peckham, S. (2017). The role of the Quality and Outcomes Framework in the care of long-term conditions: a systematic review. British Journal of General Practice, 67(664), e775–e784.
29	561	Percentage of patients with Chronic Obstructive Pulmonary Disease who have had a review with assessment of breathlessness	Process	Chronic	Screening and prevention	Effective	R - Respiratory	Percentage of patients with Chronic Obstructive Pulmonary Disease who have had a review with assessment of breathlessness	Forbes, L. J., Marchand, C., Doran, T., & Peckham, S. (2017). The role of the Quality and Outcomes Framework in the care of long-term conditions: a systematic review. British Journal of General Practice, 67(664), e775–e784.
29	562	Percentage of patients with Chronic Obstructive Pulmonary Disease who have had influenza immunisation	Process	Preventive	Screening and prevention	Effective	R - Respiratory	Percentage of patients with Chronic Obstructive Pulmonary Disease who have had influenza immunisation	Forbes, L. J., Marchand, C., Doran, T., & Peckham, S. (2017). The role of the Quality and Outcomes Framework in the care of long-term conditions: a systematic review. British Journal of General Practice, 67(664), e775–e784.
29	563	Percentage of patients with Chronic Obstructive Pulmonary Disease with a record of forced expiratory volume in 1 second (FEV1)	Process	Chronic	Diagnosis	Effective	R - Respiratory	The percentage of patients with COPD with a record of FEV1 in the preceding 12 months	There is a gradual deterioration in lung function in patients with COPD. This deterioration accelerates with the passage of time. There are important interventions which can improve quality of life in patients with severe COPD. It is therefore important to monitor respiratory function in order to identify patients who might benefit from pulmonary rehabilitation or continuous oxygen therapy. // The NICE clinical guideline on COPD recommends that FEV1 and inhaler technique are assessed at least annually for patients with mild/moderate/severe COPD (and at least twice a year for patients with very severe COPD). The purpose of regular monitoring is to identify patients with increasing severity of disease who may benefit from referral for more intensive treatments/diagnostic review. // Forbes, L. J., Marchand, C., Doran, T., & Peckham, S. (2017). The role of the Quality and Outcomes Framework in the care of long-term conditions: a systematic review. British Journal of General Practice, 67(664), e775–e784.
29	564	Percentage of patients with coronary heart disease taking aspirin, an alternative antiplatelet therapy, or an anticoagulant	Process	Chronic	Treatment	Effective	K - Cardiovascular	The percentage of patients with coronary heart disease with a record in the preceding 12 months that aspirin, an alternative anti-platelet therapy, or an anti-coagulant is being taken	Forbes, L. J., Marchand, C., Doran, T., & Peckham, S. (2017). The role of the Quality and Outcomes Framework in the care of long-term conditions: a systematic review. British Journal of General Practice, 67(664), e775–e784. // Both NICE and SIGN clinical guidelines recommend that aspirin (75 150 mg per day) is given routinely and continued for life in all patients with CHD unless there is a contraindication. Clopidogrel (75 mg/day) is an effective alternative in patients with contraindications to aspirin, or who are intolerant of aspirin.
29	565	Percentage of patients with coronary heart disease with blood pressure 150/90 mmHg or less	Outcome	Chronic	Screening and prevention	Effective	K - Cardiovascular	The percentage of patients with coronary heart disease in whom the last blood pressure reading (measured in the preceding 12 months) is 150/90 mmHg or less	This indicator measures the intermediate health outcome of a blood pressure of 150/90 mmHg or less in patients with hypertension and CHD. Its intent is to promote the secondary prevention of cardiovascular disease (CVD) through satisfactory blood pressure control. This intermediate outcome can be achieved through lifestyle advice and the use of drug therapy.<br>Forbes, L. J., Marchand, C., Doran, T., & Peckham, S. (2017). The role of the Quality and Outcomes Framework in the care of long-term conditions: a systematic review. British Journal of General Practice, 67(664), e775–e784.<br>The NICE clinical guideline on hypertension23 sets blood pressure thresholds for the initiation of drug treatment of hypertension and these are outlined in the hypertension indicator set. To summarise, patients with CHD and stage one hypertension are recommended drug therapy for hypertension.<br><br>The NICE clinical guideline on hypertension recommends a target blood pressure below 140/90 mmHg in patients aged 79 or under with treated hypertension and a clinic blood pressure below 150/90 mmHg in patients aged 80 or over, with treated hypertension. For the purpose of QOF, an audit standard of 150/90 mmHg has been adopted for this indicator.<br><br>A major overview of randomised trials showed that a reduction of 5 6 mmHg in blood pressure sustained over five years reduces coronary events by 20 25 per cent in patients with CHD.
29	566	Percentage of patients with dementia whose care plan has been reviewed face-to-face	Process	Chronic	Follow up and continuity	Patient-centered	P - Psychological	The percentage of patients diagnosed with dementia whose care has been reviewed in a face-toface review in the preceding 12 months	The face-to-face review focuses on support needs of the patient and their carer. In particular the review addresses four key issues:<br>1. an appropriate physical and mental health review for the patient,<br>2. information commensurate with the stage of the illness and his or her and the patient's health and social care needs,<br>3. if applicable, the impact of caring on the care-giver,<br>4. communication and co-ordination arrangements with secondary care (if applicable).<br>[...]
29	567	Percentage of patients with diabetes with blood pressure 140/80 mmHg or less	Outcome	Chronic	Screening and prevention	Effective	T - Endocrine/Metabolic and Nutritional	The percentage of patients with diabetes, on the register, in whom the last blood pressure reading (measured in the preceding 12 months) is 140/80 mmHg or less	Blood pressure lowering in patients with diabetes reduces the risk of macrovascular and microvascular disease.<br><br>This indicator sets a target of 140/80 mmHg as per the target recommended by NICE

29	568	Percentage of patients with diabetes with blood pressure 150/90 mmHg or less	Outcome	Chronic	Screening and prevention	Effective	T - Endocrine/Metabolic and Nutritional / K - Cardiovascular	The percentage of patients with diabetes, on the register, in whom the last blood pressure reading (measured in the preceding 12 months)is 150/90 mmHg or less	Blood pressure lowering in patients with diabetes reduces the risk of macrovascular and microvascular disease. the target of 150/90 mmHg has been set for those patients who cannot manage this, such as those with retinopathy, micro-albuminuria or cerebrovascular disease.<br><br>Setting a blood pressure target at a higher level, but expecting most patients to have blood pressure below this, is intended to encourage practitioners to address the needs of the minority of patients whose blood pressure is hard to control and will avoid the possibility of perverse incentives to focus efforts away from those at highest absolute risk.
29	569	Percentage of patients with diabetes with glycosylated haemoglobin 59 mmol/mol or less	Outcome	Chronic	Screening and prevention	Effective	T - Endocrine/Metabolic and Nutritional	The percentage of patients with diabetes, on the register, in whom the last IFCCHbA1c is 59 mmol/mol or less in the preceding 12 months	The three target levels for HbA1c (59, 64 and 75 mmol/mol) in QOF are designed to provide an incentive to improve glycaemic control across the distribution of HbA1c values. The lower level may not be achievable or appropriate for all patients. The 2009 NICE clinical guideline on the management of type 2 diabetes advises against pursuing highly intensive management to levels below 48 mmol/mol in certain patient sub-groups.<br><br>There is a near linear relationship between glycaemic control and death rate in patients with type 2 diabetes63. In the EPIC Norfolk population cohort, a one per cent higher HbA1c was independently associated with 28 per cent higher risk of death, an association that extended below the diagnostic cut off for diabetes. These results suggest that, as with blood pressure and cholesterol, over the longer term at least, the lower the HbA1c the better64.<br><br>However, the Action to Control Cardiovascular Risk in Diabetes (ACCORD) trial highlighted the risks of adopting an aggressive treatment strategy for patients at risk of CVD.In the trial's intervention group, HbA1c fell from 8.1 per cent to 6.4 per cent, but this was associated with increased mortality65. However, a recent metaanalysis did not confirm such an increase in risk66 and reassuringly, the ADVANCE study67 and the Veteran Affairs Diabetes Trial68 found no increase in all-cause mortality in their intensive treatment groups. Also, long-term follow up of the UK Prospective Diabetes Study demonstrated a "legacy effect" with fewer deaths after ten years in those initially managed intensively69.<br><br>A retrospective analysis of cohort data from the UK General Practice Research Database (GPRD) has reopened the debate about how low to aim70. The study found that, among people whose treatment had been intensified by the addition of insulin or a sulphonylurea, there was no benefit in reducing HbA1c below 59 mmol/mol, although these differences were not statistically significant. The mortality rate was higher among those with the tightest control (this lowest decile of cohort had HbA1c below 6.7 per cent; median = 6.4 per cent). The reasons for these findings are unclear, but they raise further questions about the possibility of some groups of patients for whom a tight glycaemic target is inappropriate.<br><br>The NICE clinical guideline on type 2 diabetes identifies the following key priorities for implementation to help people with type 2 diabetes achieve better glycaemic control:<br>· Offer structured education to every patient and/or their carer at and around the time of diagnosis, with annual reinforcement and review. Inform patients and their carers that structured education is an integral part of diabetes care.<br>· Provide individualised and ongoing nutritional advice from a healthcare professional with specific expertise and competencies in nutrition When setting a target HbA1c:<br>1. involve the patient in decisions about their individual HbA1c target level, which may be above that of 48 mmol/mol set for people with type 2 diabetes in general<br>2. encourage the patient to maintain their individual target unless the resulting side effects (including hypoglycaemia) or their efforts to achieve this impair their quality of life<br>3. offer therapy (lifestyle and medication) to help achieve and maintain the HbA1c target level<br>4. inform a patient with higher HbA1c that reduction in HbA1c towards the agreed target is advantageous to future health<br>5. avoid pursuing highly intensive management to levels of less than 48 mmol/mol.<br><br>The NICE and SIGN clinical guidelines are consistent.<br><br>Given that there is strong evidence to support tight glycaemic control in type 1 diabetes, which is reflected in current NICE and SIGN guidelines, this indicator aims to balance risks and benefits for patients with type 2 diabetes. Younger patients with little co-morbidity are more likely to reap the benefits of tighter control, whereas less stringent goals may be more appropriate for patients with established CVD, those with a history of hypoglycaemia, or those requiring multiple medications or insulin to achieve a NICE suggested target HbA1c of 48 mmol/mol.
29	570	Percentage of patients with diabetes with glycosylated haemoglobin 64 mmol/mol or less	Outcome	Chronic	Screening and prevention	Effective	T - Endocrine/Metabolic and Nutritional	The percentage of patients with diabetes, on the register, in whom the last IFCCHbA1c is 64 mmol/mol or less in the preceding 12 months	Auditing the proportion of patients with an HbA1c below 64 mmol/mol is designed to provide an incentive to improve glycaemic control across the range of HbA1c values
29	571	Percentage of patients with diabetes with glycosylated haemoglobin 75 mmol/mol or less	Outcome	Chronic	Screening and prevention	Effective	T - Endocrine/Metabolic and Nutritional	The percentage of patients with diabetes, on the register, in whom the last IFCCHbA1c is 75 mmol/mol or less in the preceding 12 months	Auditing the proportion of patients with an HbA1c below 75 mmol/mol is designed to provide an incentive to improve glycaemic control amongst those with high levels of HbA1c who are at particular risk
29	572	Percentage of patients with diabetes with total cholesterol 5 mmol/l or less	Outcome	Chronic	Screening and prevention	Effective	T - Endocrine/Metabolic and Nutritional	The percentage of patients with diabetes, on the register, whose last measured total cholesterol (measured within the preceding 12 months) is 5 mmol/l or less	It is advised that statin therapy to reduce cholesterol is initiated and titrated as necessary to reduce total cholesterol to less than 5 mmol/l. There is ongoing debate concerning the intervention levels of serum cholesterol in diabetic patients who do not apparently have CVD.<br><br>The NICE clinical guideline on type 2 diabetes newer agents59 recommends initiating lipid lowering therapy in all patients with type 2 diabetes aged over 40 and for patients aged 39 or under recommends initiating drug therapy in patients with type 2 diabetes who have a poor cardiovascular risk factor profile.<br><br>The SIGN clinical guideline on the management of diabetes60 recommends lipid lowering drug therapy for primary prevention in patients with type 2 diabetes aged 40 or over irrespective of baseline cholesterol. For patients with type 1 diabetes SIGN recommends lipid lowering drug therapy for patients aged 40 or over and for patients aged 39 or under with both type 1 and type 2 diabetes, recommends considering lipid lowering drug therapy.
29	573	Percentage of patients with heart failure confirmed by an echocardiogram or by specialist assessment	Process	Chronic	Diagnosis	Effective	K - Cardiovascular	The percentage of patients with a diagnosis of heart failure (diagnosed on or after 1 April 2006) which has been confirmed by an echocardiogram or by specialist assessment 3 months before or 12 months after entering on to the register	This indicator requires that all patients with suspected HF are investigated29 and this is expected to involve, as a minimum, further specialist investigation (such as echocardiography) and often specialist opinion. Serum natriuretic peptides can be used to determine whether patients with clinically suspected HF need a referral for echocardiography and their use is recommended as below. Specialists may include GPs identified by NHS England as having a special interest in HF. Many HF patients will be diagnosed following specialist referral or during hospital admission and some will also have their diagnosis confirmed by tests such as cardiac scintography or angiography rather than echocardiography.<br><br>Current NICE guidance30, 31 recommends that patients with suspected HF receive both echocardiography and specialist assessment. The guidance also recommends that serum natriuretic peptides are measured in patients with suspected HF without previous MI. Patients with suspected HF who have had a previous MI or who have very high levels of serum natriuretic peptide are considered to require urgent referral due to their poor prognosis. The SIGN clinical guideline on the management of chronic HF32 recommends that echocardiography is performed in patients with suspected HF who have either a raised serum natriuretic peptide or abnormal electrocardiograph result to confirm the diagnosis and establish the underlying cause.
29	574	Percentage of patients with heart failure taking angiotensin-converting-enzyme inhibitors or angiotensin II receptor blockers	Process	Chronic	Treatment	Effective	K - Cardiovascular	In those patients with a current diagnosis of heart failure due to left ventricular systolic dysfunction, the percentage of patients who are currently treated with an ACE-I or ARB	There is strong clinical and cost-effectiveness evidence to support the use of ACE-I in all patients with HF with LVSD. ACE-I improve symptoms, reduce the hospitalisation rate and improve the survival rate. This is applicable in all age groups. ARBs are also effective in the treatment of patients with HF due to LVSD, but may only be used in patients intolerant of ACE-I.<br><br>It is possible to have a diagnosis of LVSD without HF, for example, asymptomatic people who might be identified coincidently but who are at high risk of developing subsequent HF. In such cases, ACE-I's delay the onset of symptomatic HF, reduce cardiovascular events and improve long-term survival. This indicator only applies to patients with HF and therefore excludes this other group of patients who are nevertheless to be considered for treatment with ACE-I.<br><br>NICE clinical guideline CG108 and SIGN clinical guideline 95 recommend that ACE-I is used as first-line therapy in all patients with HF due to LVSD and that ARBs are used only in patients who are intolerant of ACE-I.
29	575	Percentage of patients with hypertension and high cardiovascular risk treated with statins	Process	Chronic	Treatment	Effective	K - Cardiovascular	In those patients with a new diagnosis of hypertension aged 30 or over and who have not attained the age of 75, recorded between the preceding 1 April to 31 March (excluding those with pre-existing CHD, diabetes, stroke and/or TIA), who have a recorded CVD risk assessment score (using an assessment tool agreed with NHS England) of >=20% in the preceding 12 months: the percentage who are currently treated with statins	For primary prevention of CVD, people at risk need to be identified before CVD has become established. To assess risk in those likely to be at high-risk (for example, people with hypertension) a validated assessment tool is needed that evaluates a range of modifiable and non-modifiable risk factors.<br><br>The NICE clinical guideline on lipid modification163 recommends statin therapy for the primary prevention of CVD for adults who have an estimated 20 per cent or greater 10-year risk of developing CVD
29	576	Percentage of patients with hypertension with blood pressure of 150/90 mmHg or less	Outcome	Chronic	Diagnosis	Effective	K - Cardiovascular	The percentage of patients with hypertension in whom the last blood pressure reading (measured in the preceding 12 months) is 150/90 mmHg or less	This indicator measures the intermediate health outcome of a blood pressure of 150/90 mmHg or less in patients with hypertension. Its intent is to promote the primary and secondary prevention of CVD through satisfactory blood pressure control. This intermediate outcome can be achieved through lifestyle advice and the use of drug therapy.
29	577	Percentage of patients with long-term conditions with record of smoking status	Process	Chronic	Diagnosis	Patient-centered	A - General and unspecified	The percentage of patients with any or any combination of the following conditions: CHD, PAD, stroke or TIA, hypertension, diabetes, COPD, CKD, asthma, schizophrenia, bipolar affective disorder or other psychoses whose notes record smoking status in the preceding 12 months	The aim of this domain is to increase the proportion of successful smoking quit attempts by providing the best available support and treatment. A wide range of diseases and conditions are caused by cigarette smoking, including cancers, respiratory diseases, CHD and other circulatory diseases, stomach and duodenal ulcers, ED and infertility, osteoporosis, cataracts, age-related macular degeneration and periodontitis (US DH and Human Services 2004). Women who smoke during pregnancy are also at substantially higher risk of spontaneous abortion (miscarriage) than those who do no smoke. Smoking can also cause complications in pregnancy and labour, including ectopic pregnancy, bleeding during pregnancy, premature detachment of the placenta and premature rupture of the membranes174. CHD Smoking is known to be associated with an increased risk of CHD.<br><br>SIGN clinical guideline 97. Risk estimation and the prevention of CVD. 2007. http://www.sign.ac.uk/guidelines/fulltext/97/index.html<br><br>ESC. European Guidelines. CVD Prevention in clinical practice. 2007. http://www.sign.ac.uk/guidelines/fulltext/97/index.html<br><br>PAD PAD is associated with older age and with smoking. Cigarette smoking is a very important contributor to PAD and as such the management of PAD includes smoking cessation.<br><br>Stroke or TIA There are few RCTs of the effects of risk factor modification in the secondary prevention of ischaemic or haemorrhagic stroke. However, inferences can be drawn from the finds of primary prevention trials that cessation of cigarette smoking be advocated.<br><br>SIGN clinical guideline 108. Management of patients with stroke or TIA: assessment, investigation, immediate management and secondary prevention. 2008. http://www.sign.ac.uk/guidelines/fulltext/108/index.html<br><br>Hypertension There is no strong direct link between smoking and blood pressure. However, there is overwhelming evidence of the relationship between smoking and cardiovascular and pulmonary diseases. The NICE clinical guideline on hypertension182 recommends that patients who smoke are offered advice and help to stop smoking.<br><br>Diabetes The risk of vascular complications in patients with diabetes is substantially increased. Smoking is an established risk factor for cardiovascular and other diseases.<br><br>COPD Smoking cessation is the single most effective and cost-effective intervention to reduce the risk of developing COPD and stop its progression.<br><br>NICE clinical guideline CG101. Management of COPD in adults in primary and secondary care. 2010. http://guidance.nice.org.uk/CG101<br><br>GOLD Guidelines. http://www.goldcopd.org/<br><br>Asthma There are a surprisingly small number of studies on smoking related asthma. Starting smoking as a teenager increases the risk of persisting asthma. One controlled cohort study suggested that exposure to passive smoke at home delayed recovery from an acute attack. Smoking reduces the benefits of inhaled steroids and this adds further justification for recording this outcome183.There is also epidemiological evidence that smoking is associated with poor asthma control184.<br><br>CKD There is good evidence from observational studies that patients with CKD are at increased cardiovascular risk and hence the rationale for including CKD here.<br><br>[illegible]
29	578	Percentage of patients with non-haemorrhagic stroke or transien ischaemic attack taking antiplatelet agent, or anticoagulant	Process	Chronic	Treatment	Effective	K - Cardiovascular	The percentage of patients with a stroke shown to be non-haemorrhagic, or a history of TIA, who have a record in the preceding 12 months that an anti-platelet agent, or an anti-coagulant is being taken	Long-term anti-platelet therapy reduces the risk of serious vascular events following a stroke by about a quarter. It is advised that anti-platelet therapy is prescribed for the secondary prevention of recurrent stroke and other vascular events in patients who have sustained an ischaemic cerebrovascular event.
29	579	Percentage of patients with peripheral arterial disease taking aspirin or an alternative antiplatelet	Process	Chronic	Treatment	Effective	K - Cardiovascular	The percentage of patients with peripheral arterial disease with a record in the preceding 12 months that aspirin or an alternative anti-platelet is being taken	Most cases of PAD are managed in primary care. The focus of management is on the secondary prevention of CVD. It is important to reduce the cardiovascular complications of atherosclerosis through appropriate cardiovascular risk factor management. Two small UK studies assessing clinical risk management based on the patient records of patients with PAD41, 42 suggest that these patients have poor hypertension control, use low levels of statin and anti-platelet therapy, and receive low levels of smoking cessation advice. This indicator addresses the issue of prescribing anti-platelet therapy.<br><br>The SIGN clinical guideline on PAD43 states that anti-platelet therapy is recommended for patients with symptomatic PAD.<br><br>The Antithrombotic Trialists Collaboration (ATC) meta-analysis showed a 23 per cent reduction in serious vascular events in a subgroup of 9214 people with PAD who were treated with anti-platelet drugs44. Similar results were found in a second systematic review of the effects of anti-platelet therapy in patients with PAD45. When comparing the effects of different anti-platelet drugs, the ATC found no evidence of statistically significant differences between anti-platelets.
29	580	Percentage of patients with peripheral arterial disease with blood pressure 150/90 mmHg or less	Outcome	Chronic	Diagnosis	Effective	K - Cardiovascular	The percentage of patients with peripheral arterial disease in whom the last blood pressure reading (measured in the preceding 12 months) is 150/90 mmHg or less	Most cases of PAD are managed in primary care. The focus of treatment is on the cardiovascular complications of atherosclerosis (managing cardiovascular risk factors such as high blood pressure). Two small UK studies assessing clinical risk management based on the patient records of patients with PAD38,39 suggest that these patients have poor hypertension control, use low levels of statin and antiplatelet therapy and receive low levels of smoking cessation advice. This indicator addresses the issue of blood pressure control.
29	581	Percentage of patients with severe Chronic Obstructive Pulmonary Disease with record of oxygen saturation	Process	Chronic	Diagnosis	Effective	R - Respiratory	The percentage of patients with COPD and Medical Research Council dyspnoea grade 3 at any time in the preceding 12 months, with a record of oxygen saturation value within the preceding 12 months	As COPD progresses, patients often become hypoxaemic. Many patients tolerate mild hypoxaemia well, but once the resting partial pressure of oxygen in arterial blood (PaO2) falls below 8 KPa patients begin to develop signs of right-sided HF (corpulmonale), principally peripheral oedema. The prognosis is poor and if untreated the five year survival is less than 50 per cent.<br><br>In stable COPD, patients use oxygen therapy for long periods during the day and night. Long-term oxygen therapy can improve survival in patients with COPD who have severe hypoxaemia, where PaO2 is less than 8 KPa. It can also reduce the incidence of polycythaemia (that is, raised red cell count), reducing the progression of pulmonary hypertension and improving psychological wellbeing.<br><br>NICE clinical guideline CG101 recommends that patients with oxygen saturations of 92 per cent or lower when breathing air, be considered for oxygen therapy. Pulse oximetry (SpO2) provides an estimate of arterial oxygen saturation (SaO2) and is noninvasive.
29	582	Percentage of patients with STIA referred for further investigation	Process	Chronic	Follow up and continuity	Effective	K - Cardiovascular	The percentage of patients with a stroke or TIA (diagnosed on or after 1 April 2014) who have a record of a referral for further investigation between 3 months before or 1 month after the date of the latest recorded stroke or the first TIA	Specialist investigations are often only accessible by a referral to secondary care services, therefore this indicator reflects referral activity rather than confirmation by specific scanning investigations.<br><br>Previously this indicator required that practices recorded a referral for further investigation after the last recorded stroke or TIA. From April 2014 this indicator was amended so that practices are only required to record a referral for further investigations following the first TIA or latest stroke for achievement. This is to allow for clinical discretion for referral of subsequent TIAs. However, practices are reminded that current NICE and Royal College of Physician guidelines for stroke recommend that patients with suspected TIA should receive specialist assessment and investigation within a timeframe based on stroke risk. A TIA is an opportunity to prevent a stroke and therefore good practice is to refer people in line with current national clinical guidelines
29	583	Percentage of patients with STIA with blood pressure 150/90 mmHg or less	Outcome	Chronic	Diagnosis	Effective	K - Cardiovascular	The percentage of patients with a history of stroke or TIA in whom the last blood pressure reading (measured in the preceding 12 months) is 150/90 mmHg or less	This indicator measures the intermediate health outcome of a blood pressure of 150/90 mmHg or less in patients with hypertension and CHD. Its intent is to promote the secondary prevention of CVD through satisfactory blood pressure control. This intermediate outcome can be achieved through lifestyle advice and the use of drug therapy.

29	584	Percentage of QOF patients with diabetes with a record of a foot examination and foot risk classification	Process	Chronic	Diagnosis	Effective	T - Endocrine/Metabolic and Nutritional	The percentage of patients with diabetes, on the register, with a record of foot examination and risk classification: 1) low risk (normal sensation, palpable pulses), 2) increased risk (neuropathy or absent pulses), 3) high risk (neuropathy or absent pulses plus deformity or skin changes in previous ulcer) or 4) ulcerated foot within the preceding 12 months	Patients with diabetes are at high risk of foot complications. Evaluation of skin, soft tissue, musculoskeletal, vascular and neurological condition on an annual basis is important for the detection of feet at raised risk of ulceration.
29	585	Percentage of smokers with long-term conditions offered smoking cessation support	Process	Chronic	Treatment	Effective	A - General and unspecified	The percentage of patients with any or any combination of the following conditions: CHD, PAD, stroke or TIA, hypertension, diabetes, COPD, CKD, asthma, schizophrenia, dipolar affective disorder or other psychoses who are recorded as current smokers who have a record of an offer of support and treatment within the preceding 12 months	The aim of this domain is to increase the proportion of successful smoking quit attempts by providing the best available support and treatment. A wide range of diseases and conditions are caused by cigarette smoking, including cancers, respiratory diseases, CHD and other circulatory diseases, stomach and duodenal ulcers, ED and infertility, osteoporosis, cataracts, age-related macular degeneration and periodontitis (US DH and Human Services 2004). Women who smoke during pregnancy are also at substantially higher risk of spontaneous abortion (miscarriage) than those who do no smoke. Smoking can also cause complications in pregnancy and labour, including ectopic pregnancy, bleeding during pregnancy, premature detachment of the placenta and premature rupture of the membranes174
29	586	Register of patients with atrial fibrillation	Outcome	Chronic	Diagnosis	Patient-centered	K - Cardiovascular	The contractor establishes and maintains a register of patients with atrial fibrillation	
31(50)	587	<=0.6 prescriptions/100 PUs for drugs with limited indications (e.g. cerebral and peripheral vasodilators) (BNF sections 2.6.3/2.6.4)	Process	Chronic	Treatment	Effective	K - Cardiovascular	The percentage of general practitioner with <=0.6 prescriptions/100 PUs for drugs with limited indications [Cerebral and peripheral vasodilators (BNF sections 2.6.3/2.6.4)]	The aim in setting the standards was to choose a level which, when achieved, would reflect good prescribing. It was regarded as inevitable that for some criteria, where the group felt there was widespread poor prescribing, the standard would be achieved by only a small proportion of practices. Numeric standards were set in three ways: first, from the rate of generic prescribing; secondly, setting the proportion of a specific therapeutic area accounted for by 'preferred' drug(s); and thirdly, for markers of poor prescribing, by setting absolute levels of prescribing. For some markers of poor prescribing, the standard was based on a general practitioner issuing just over one prescription per month (15 items or fewer per year), which translated to 0.6 items per 100 prescribing units per year.
31(50)	588	<=0.6 prescriptions/100 PUs for Diuretic-potassium combinations with limited clinical value (BNFsection 2.2.8)	Process	Chronic	Treatment	Effective	K - Cardiovascular	The percentage of general practitioner with <=0.6 prescriptions/100 PUs for Diuretic-potassium combinations with limited clinical value (BNFsection 2.2.8)	
31(50)	589	Frusemide and bendrofluazide (as % of BNFsection 2.2 drugs) >=55	Process	Chronic	Treatment	Effective	K - Cardiovascular	The percentage of general practitioner choosing Frusemide and bendrofluazide from BNFsection 2.2 drugs >=55 times	
31(50)	590	Atenolol and propranolol (as % of BNFsection 2.4 drugs) >=75	Process	Chronic	Treatment	Effective	K - Cardiovascular	The percentage of general practitioner choosing Atenolol and propranolol from BNFsection 2.4 drugs >=75 times	
31(16)	591	Change amlodipine to felodipine	Process	Chronic	Treatment	Effective	T - Endocrine/Metabolic and Nutritional / K - Cardiovascular	N/A	A few indicators appeared idiosyncratic such as increasing atorvastatin and cerivastatin as a proportion of all statins (cerivastatin was withdrawn in 2001 following reports of serious side-effects) or changing amlodipine to felodipine
31	592	Co-prescriptions to be avoided	Process	Chronic	Treatment	Safe	A - General and unspecified	Co-prescriptions to be avoided, e.g. of statins with macrolides, diuretic, ACE-inhibitor with potassium or NSAID, metformin with glibenclamide, etc.	
31(52)	593	Prescription of high dose hydrochlorthiazide	Process	Chronic	Treatment	Effective	K - Cardiovascular	Percentage of patients with heart failure hospital discharge prescribed with hydralazine/isordil or angiotensin II receptor antagonist at discharge or 6 months after discharge	
31 (16)	594	Prescription of low dose bendrofluazide	Process	Chronic	Treatment	Effective	K - Cardiovascular	Proportion of low dose bendrofluazide (2.5 mg)	N/A
31(23, 25, 26, 27)	595	Once-or twice- daily dosing of antihypertensives in elderly	Process	Chronic	Treatment	Effective	K - Cardiovascular	IF a vulnerable elder requires pharmacotherapy for treatment of hypertension in the outpatient setting, THEN a once- or twice-daily medication should be used unless there is documentation about the need for agents that require more frequent dosing.	To be a meaningful measure of quality, a process of care must be related to improved patient outcomes. For many quality indicators, this relationship is based on evidence of efficacy from randomized, controlled trials, usually among a select patient population.
31(47)	596	Cost of treatment per unit	Structure	Chronic	Treatment	Efficient	A - General and unspecified	DDD (dosis diaria definida) ARA-II/DDD IECA + ARA-II; DDD (dosis diaria definida) estatinas de elección/DDD estatinas; DDD (dosis diaria definida) antihipertensivos de elección/total antihipertensivos; DHD (dosis por mil habitantes y día) estatinas; Dose/1000persons/day of lipid lowering drugs; AINE primera elección/total AINE	
31(4)	597	Drugs to be avoided	Process	Preventive	Treatment	Safe	K - Cardiovascular	Medication Management in the Elderly (DAE/DDE): Potentially Harmful Drug-Disease Interactions in the Elderly: Assesses adults 65 and older who have a specific disease or condition (chronic renal failure, dementia, history of falls) and were dispensed a prescription for a medication that could exacerbate it. Use of High-Risk Medications in the Elderly: Assesses adults 65 and older who had at least one dispensing event for a high-risk medication or who had at least 2 dispensing events for the same high-risk medication.	
31(15)	598	Drugs to be avoided	Process	Preventive	Treatment	Safe	K - Cardiovascular	Potentially inappropriate drug selection (PIDS): The rate of PIDS was calculated for each category as the number of prescriptions for the potentially inappropriate drug or drugs in a category divided by the total number of prescriptions for that category. The percentage of elderly exposed to a potentially inappropriate drug was calculated as the number of individuals exposed to a potentially inappropriate drug divided by the total eligible population of 355600	
31(21)	599	Drugs to be avoided	Process	Preventive	Treatment	Safe	K - Cardiovascular	Appropriate aspirin prescription in cardiac ischaemia: Nitrate with aspirin prescribing (Indicator reports result as a percentage of residents prescribed the key drug/drug group (nitrate, ß2 agonist, digoxin/amiodarone)	
31(21)	600	Drugs to be avoided	Process	Preventive	Treatment	Safe	K - Cardiovascular	Appropriate anticoagulant/aspirin 300 mg prescription in atrial fibrillation: Digoxin/amiodarone with warfarin or aspirin 300 mg in AF prescribing (Indicator reports result as a percentage of residents prescribed the key drug/drug group (nitrate, ß2 agonist, digoxin/amiodarone).)	
31(23, 25, 26, 27)	601	Drugs to be avoided	Process	Preventive	Treatment	Safe	K - Cardiovascular	Calcium Channel Blockers: IF a VE has heart failure, LVEF of less than 40%, and no atrial fibrillation, THEN he or she should not be treated with a first- or second-generation calcium channel blocker	https://onlinelibrary.wiley.com/doi/full/10.1111/j.1532-5415.2007.01341.x
31(23, 25, 26, 27)	602	Drugs to be avoided	Process	Preventive	Treatment	Safe	K - Cardiovascular	Type I Antiarrhythmic Agents: IF a VE has heart failure and an LVEF of less than 40%, THEN he or she should not be treated with a type I antiarrhythmic agent unless an implantable cardioverter defibrillator is in place.	https://onlinelibrary.wiley.com/doi/full/10.1111/j.1532-5415.2007.01341.x
31(23, 25, 26, 27)	603	Drugs to be avoided	Process	Preventive	Treatment	Safe	K - Cardiovascular	Patient Counseling: IF a VE is newly diagnosed or hospitalized with heart failure, THEN patient counseling regarding medication use, dosage, intervals, side effects; low-salt diet; exercise and physical activity; smoking cessation; weight monitoring; symptom management; avoiding or minimizing use of nonsteroidal antiinflammatory drugs; and prognosis and end-of-life concerns should be provided and documented.	
31(23, 25, 26, 27)	604	Drugs to be avoided	Process	Preventive	Treatment	Safe	K - Cardiovascular	Digoxin Monitoring: IF a VE with heart failure is taking digoxin and has signs of toxicity, THEN a digoxin level should be checked or digoxin discontinued within 1 week	
31(28)	605	Drugs to be avoided	Process	Preventive	Treatment	Safe	K - Cardiovascular	2002 Criteria for Potentially Inappropriate Medication Use in Older Adults: Considering Diagnoses or Conditions: Disopyramide (Norpace), and high sodium content drugs (sodium and sodium salts [alginate bicarbonate, biphosphate, citrate, phosphate, salicylate, and sulfate]); Phenylpropanolamine hydrochloride (removed from the market in 2001), pseudoephedrine; diet pills, and amphetamines; Tricyclic antidepressants (imipramine hydrochloride, doxepin hydrochloride, and amitriptyline hydrochloride)	
31(52)	606	Drugs to be avoided	Process	Preventive	Treatment	Safe	K - Cardiovascular	No chronic use of nonsteroidal anti-inflammatory drugs by 6 months after discharge: Percentage of patients with heart failure hospital discharge and no chronic use of nonsteroidal anti-inflammatory drugs by 6 months after discharge	DiSalvo TG, Normand SL, Hauptman PJ, Guadagnoli E, Palmer RH, McNeil BJ. Pitfalls in assessing the quality of care for patients with cardiovascular disease. The American journal of medicine. 2001 Sep 1;111(4):297-303.
31(52)	607	Drugs to be avoided	Process	Preventive	Treatment	Safe	K - Cardiovascular	No prescription of short-acting nifedipine: Percentage of patients with hypertension and no prescription of short-acting nifedipine	DiSalvo TG, Normand SL, Hauptman PJ, Guadagnoli E, Palmer RH, McNeil BJ. Pitfalls in assessing the quality of care for patients with cardiovascular disease. The American journal of medicine. 2001 Sep 1;111(4):297-303.
31(52)	608	Drugs to be avoided	Process	Preventive	Treatment	Safe	K - Cardiovascular	No prescription of hydrochlorothiazide >=50 mg/day: Percentage of patients with hypertension and no prescription of hydrochlorothiazide >=50 mg/day	DiSalvo TG, Normand SL, Hauptman PJ, Guadagnoli E, Palmer RH, McNeil BJ. Pitfalls in assessing the quality of care for patients with cardiovascular disease. The American journal of medicine. 2001 Sep 1;111(4):297-303.
31(43)	609	Number of different brands with the same active substance DU90% within a specific drug class	Process	Preventive	Treatment	Effective	A - General and unspecified	For indicators directed towards making a limited choice from a group of drugs the DU90% method was used [23]. This method relates the quality of prescribing to the number of different drugs (in terms of DDDs) that are responsible for 90% of the drug use, the DU90% index. The method assumes that good prescribing is correlated with a relatively limited choice from the available range of drugs. DU90% Oralcontraceptives (n=14->DU90%=5); DU90%NSAIDs (n=21->DU90%=4); DU90% topicalcorticosteroids (n=21->DU90%=7); DU90% ACEinhibitors (n=10->DU90%=4); DU90%Betablockers (n=16->DU90%=5)	
31(10)	610	Number of prescriptions for (preferred) drugs per PU (or ASTRO-PU)	Process	Chronic	Treatment	Effective	K - Cardiovascular	Percentage of lipid lowering drugs per ASTRO-PU	
31 (16,59)	611	Patients on long acting isosorbide nitrate, glibenclamide, combinations of diuretics or a-glucosidase inhibitor, etc.	Process	Chronic	Treatment	Effective	K - Cardiovascular	Reduce proportion of long acting preparations of isosorbide mononitrate [31(16)]; Porcentaje de inhibidores de la alfaglucosidasa/total de ADO [31(59)]	
31 (47, 59)	612	Patients on novelty drugs, such as angiotensin II receptor blockers or thiazolidinediones, of all patients receiving antihypertensives or oral glucose-lowering drugs	Process	Chronic	Treatment	Effective	K - Cardiovascular	DDD ARA-II/DDD IECA + ARA-II [31(47)]; Porcentaje de antagonistas de los receptores de la angiotensina II (ARA-II)/total de antihipertensivos. [31(59)]	
31 (47,59)	613	Patients on preferred drug classes (e.g., diuretics or b-blockers) of all antihypertensives	Process	Chronic	Treatment	Effective	K - Cardiovascular	DDD antihipertensivos de elección/total antihipertensivos [31(47)]; Porcentaje de diuréticos y bloqueadores beta/total de antihipertensivos [31(59)]	
31(43)	614	Patients prescribed angiotensin II receptor blockers and prior to this an angiotensin-converting-enzyme inhibitor of all patients prescribed angiotensin II receptor blockers	Process	Chronic	Treatment	Effective	K - Cardiovascular	Patients who were prescribed na angiotensin II - antagonist (AT-2)and prior to this na ACE inhibitor, divided by no. Of patients who received na AT-2 · 100%	
31 (16,59)	615	Percentage of First choice drug class (e.g. biguanides) of all oral antidiabetic drugs	Process	Chronic	Treatment	Effective	K - Cardiovascular	Porcentaje de metformina/total de antidiabéticos orales (ADO).[31(59)] Of greater concern was one PCG that had a diabetic indicator aiming for over 80% of oral hypoglycemics to be metformin. Such an indicator, if over-enthusiastically enforced, could have adversely affected diabetic control in the population of that PCG. [31(16)]	
3	616	Diagnosis and treatment - primary care: Hospitalization for ambulatory care sensitive conditions	Outcome	Acute / Chronic	Treatment	Effective	Not Defined	Diagnosis and treatment - primary care: Hospitalization for ambulatory care sensitive conditions	1. Kringos, D. S., Boerma, W. G., Hutchinson, A., van der Zee, J., & Groenewegen, P. P. (2010). The breadth of primary care: a systematic literature review of its core dimensions. BMC Health Services Research, 10(1) // 2. Marshall M, Klazinga N, Leatherman S, Hardy C, Bergmann E, Pisco L, et al: OECD Health Care Quality Indicator Project. The expert panel on primary care prevention and health promotion. Int J Qual Health Care 2006, 18(Suppl 1):21-25.
3	617	ACSC adjusted	Outcome	Acute / Chronic	Treatment	Effective	Not Defined	After adjustment for age, sex, and severity of illness, significant predictors of higher admission rates of ACSCs within rural areas include lack of insurance, emergency admissions, higher degree of remoteness, lower population density, lower number of general practitioners/10000 population by local government area (LGA), lower number of general practitioner visits per person by LGA, and areas with lower socio-economic status, education and occupation, and economic resources.	1. Kringos, D. S., Boerma, W. G., Hutchinson, A., van der Zee, J., & Groenewegen, P. P. (2010). The breadth of primary care: a systematic literature review of its core dimensions. BMC Health Services Research, 10(1) // 2. Ansari Z, Barbetti T, Carson NJ, Auckland MJ, Cicuttini F: The Victorian ambulatory care sensitive conditions study: rural and urban perspectives. Soz Praventivmed 2003, 48:33-43.
4	618	Counselling/help to stop smoking for patients with serious mental illness	Process	Preventive	Treatment	Effective	P - Psychological	Patients with serious mental illness who smoke who are offered tobacco counselling/help to stop smoking	1. National Institute for Health and Care Excellence. Psychosis and schizophrenia in adults: prevention and management. CG178. London: NICE, 2014. https://www.nice.org.uk/guidance/cg178 (accessed 20 Mar 2019). || 2. Kronenberg C, Doran T, Goddard M, Kendrick T, Gilbody S, Dare CR, et al. Identifying primary care quality indicators for people with serious mental illness: a systematic review. The British journal of general practice : the journal of the Royal College of General Practitioners. 2017;67(661):e519-e30. || 3. Huang, H.,et al. Psychopathology and extrapyramidal side effects in smoking and non-smoking patients with schizophrenia: Systematic review and meta-analysis of comparative studies. Progress in Neuro-Psychopharmacology and Biological Psychiatry. (2019) doi:10.1016/j.pnpbp.2019.02.011
4	619	Alcohol misuse screening	Process	Preventive	Screening and prevention	Effective	P - Psychological	Number of patients with spectrum of behaviors, including risky or hazardous alcohol use (e.g. harmful alcohol use and alcohol abuse or dependence).	1. Agency for Healthcare Research and Quality. AHRQ — quality indicators. AHRQ, 2016. www.qualityindicators.ahrq.gov (accessed 20 Mar 2019) || 2. Kronenberg C, Doran T, Goddard M, Kendrick T, Gilbody S, Dare CR, et al. Identifying primary care quality indicators for people with serious mental illness: a systematic review. The British journal of general practice : the journal of the Royal College of General Practitioners. 2017;67(661):e519-e30.
4	620	Ilicit drugs misuse screening	Process	Preventive	Screening and prevention	Effective	P - Psychological	Number of patient screenings for illicit drug use, type, quantity, and frequency	1. Agency for Healthcare Research and Quality. AHRQ — quality indicators. AHRQ, 2016. www.qualityindicators.ahrq.gov (accessed 20 Mar 2019). || 2. Kronenberg C, Doran T, Goddard M, Kendrick T, Gilbody S, Dare CR, et al. Identifying primary care quality indicators for people with serious mental illness: a systematic review. The British journal of general practice : the journal of the Royal College of General Practitioners. 2017;67(661):e519-e30.
4	621	Refferal to substance misuse disorder specialty care	Process	Chronic	Treatment	Effective	P - Psychological	Number of appropriate referral to substance misuse disorder specialty care.	1. Agency for Healthcare Research and Quality. AHRQ — quality indicators. AHRQ, 2016. www.qualityindicators.ahrq.gov (accessed 20 Mar 2019) || 2.Kronenberg C, Doran T, Goddard M, Kendrick T, Gilbody S, Dare CR, et al. Identifying primary care quality indicators for people with serious mental illness: a systematic review. The British journal of general practice : the journal of the Royal College of General Practitioners. 2017;67(661):e519-e30.
4	622	HIV screening with co-occurring substance misuse	Process	Chronic	Screening and prevention	Effective	P - Psychological	HIV screening with co-occurring substance misuse for serious mental illness service users	1. Swartz L, MacGregor H. Integrating services, marginalizing patients: psychiatric patients and primary health care in South Africa. Transcult Psychiatry 2002; 39(2): 155–172. || 2. Kronenberg C, Doran T, Goddard M, Kendrick T, Gilbody S, Dare CR, et al. Identifying primary care quality indicators for people with serious mental illness: a systematic review. The British journal of general practice : the journal of the Royal College of General Practitioners. 2017;67(661):e519-e30.
4	623	Surveillance to prevent relapse	Process	Preventive	Follow up and continuity	Safe	P - Psychological	Surveillance to prevent relapse	1. Sweeney A, Rose D, Clement S, et al. Understanding service user-defined continuity of care and its relationship to health and social measures: a crosssectional study. BMC Health Serv Res 2012; 12: 145 || 2. Kronenberg C, Doran T, Goddard M, Kendrick T, Gilbody S, Dare CR, et al. Identifying primary care quality indicators for people with serious mental illness: a systematic review. The British journal of general practice : the journal of the Royal College of General Practitioners. 2017;67(661):e519-e30
4	624	Extra pyramidal effects monitoring	Process	Preventive	Follow up and continuity	Safe	P - Psychological	Patients suffering extra pyramidal effects monitored and check compliance	1. Kronenberg C, Doran T, Goddard M, Kendrick T, Gilbody S, Dare CR, et al. Identifying primary care quality indicators for people with serious mental illness: a systematic review. The British journal of general practice : the journal of the Royal College of General Practitioners. 2017;67(661):e519-e30. || 2. Lader M. Consensus statements on standards of care in schizophrenia. Prim Care Psychiatr 1997; 3(3): 145–149.
6	625	Vulnerable elders screened to detect problem drinking and hazardous drinking	Process	Preventive	Screening and prevention	Effective	P - Psychological	Number of vulnerable elders screened at least once to detect problem drinking and hazardous drinking by taking a history of alcohol use or by using standardized screening questionnaires	1. Fujita K., Moles RJ, Chen TF. Quality indicators for responsible use of medicines: a systematic review BMJ Open 2018;8:e020437. doi: 10.1136/bmjopen-2017-020437 || 2. Kröger E, Tourigny A, Morin D, et al. Selecting process quality indicators for the integrated care of vulnerable older adults affected by cognitive impairment or dementia. BMC Health Serv Res 2007;7:195. || 3. Chin WY, Lam CL, Lo SV. Quality of care of nurse-led and allied health personnel-led primary care clinics. Hong Kong medical journal = Xianggang yi xue za zhi. 2011;17(3):217-30.
6	626	Vulnerable elders screened to tobacco use	Process	Preventive	Screening and prevention	Effective	P - Psychological	Number of vulnerable elders screened at least once to detect whether they use tobacco regularly	1. Fujita K., Moles RJ, Chen TF. Quality indicators for responsible use of medicines: a systematic review BMJ Open 2018;8:e020437. doi: 10.1136/bmjopen-2017-020437 || 2. Kröger E, Tourigny A, Morin D, et al. Selecting process quality indicators for the integrated care of vulnerable older adults affected by cognitive impairment or dementia. BMC Health Serv Res 2007;7:195. || 3. Chin WY, Lam CL, Lo SV. Quality of care of nurse-led and allied health personnel-led primary care clinics. Hong Kong medical journal = Xianggang yi xue za zhi. 2011;17(3):217-30.
6	627	Vulnerable elders counselling to quit smoking	Process	Preventive	Treatment	Effective	P - Psychological	Number of vulnerable elder offered counselling and/or pharmacological therapy at least once to stop tobacco use	1. Fujita K., Moles RJ, Chen TF. Quality indicators for responsible use of medicines: a systematic review BMJ Open 2018;8:e020437. doi: 10.1136/bmjopen-2017-020437 || 2. Kröger E, Tourigny A, Morin D, et al. Selecting process quality indicators for the integrated care of vulnerable older adults affected by cognitive impairment or dementia. BMC Health Serv Res 2007;7:195. || 3. Chin WY, Lam CL, Lo SV. Quality of care of nurse-led and allied health personnel-led primary care clinics. Hong Kong medical journal = Xianggang yi xue za zhi. 2011;17(3):217-30.
6	628	Vulnerable elders in physical activity	Process	Preventive	Screening and prevention	Effective	P - Psychological	Vulnerable elders that should receive an assessment of their level of physical activity at least once a year and, if necessary be provided with counselling about appropriate resources	1. Fujita K., Moles RJ, Chen TF. Quality indicators for responsible use of medicines: a systematic review BMJ Open 2018;8:e020437. doi: 10.1136/bmjopen-2017-020437 || 2. Kröger E, Tourigny A, Morin D, et al. Selecting process quality indicators for the integrated care of vulnerable older adults affected by cognitive impairment or dementia. BMC Health Serv Res 2007;7:195. || 3. Chin WY, Lam CL, Lo SV. Quality of care of nurse-led and allied health personnel-led primary care clinics. Hong Kong medical journal = Xianggang yi xue za zhi. 2011;17(3):217-30.
8	629	Percentage of patients with serious mental health problems with record of blood pressure	Process	Preventive	Screening and prevention	Effective	P - Psychological	% patients with serious mental health problems with record of blood pressure	Lake R, Georgiou A, Li J, Li L, Byrne M, Robinson M, et al. The quality, safety and governance of telephone triage and advice services - an overview of evidence from systematic reviews. BMC health services research. 2017;17(1):621
8	630	Percentage of patients with serious mental health problems with record of alcohol consumption	Process	Preventive	Screening and prevention	Effective	P - Psychological	% patients with serious mental health problems with record of alcohol consumption	Lake R, Georgiou A, Li J, Li L, Byrne M, Robinson M, et al. The quality, safety and governance of telephone triage and advice services - an overview of evidence from systematic reviews. BMC health services research. 2017;17(1):622
8	631	Percentage of women with serious mental health problems with cervical screening test performed	Process	Preventive	Screening and prevention	Effective	P - Psychological	% women with serious mental health problems with cervical screening test performed	Lake R, Georgiou A, Li J, Li L, Byrne M, Robinson M, et al. The quality, safety and governance of telephone triage and advice services - an overview of evidence from systematic reviews. BMC health services research. 2017;17(1):623
8	632	Percentage of patients on lithium therapy having renal and thyroid function monitored	Process	Preventive	Screening and prevention	Safe	P - Psychological	% patients on lithium therapy having renal and thyroid function monitored	Lake R, Georgiou A, Li J, Li L, Byrne M, Robinson M, et al. The quality, safety and governance of telephone triage and advice services - an overview of evidence from systematic reviews. BMC health services research. 2017;17(1):624

8	633	Percentage of patients on lithium therapy with lithium levels in therapeutic range	Process	Preventive	Screening and prevention	Safe	P - Psychological	% patients on lithium therapy with lithium levels in therapeutic range	Lake R, Georgiou A, Li J, Li L, Byrne M, Robinson M, et al. The quality, safety and governance of telephone triage and advice services - an overview of evidence from systematic reviews. BMC health services research. 2017;17(1):625
24	634	Usage of systemic antibiotics in dental treatments without indication for antibiotics	Process	Acute	Treatment	Safe	D - Digestive	This indicator describes unnecessary antibiotic prescriptions in dental treatments	Hussein RJ, Krohn R, Kaufmann-Kolle P, et al. Quality indicators for the use of systemic antibiotics in dentistry. Z Evid Fortbild Qual Gesundhwes 2017;122:1–8.
2	635	Emergency contraception	Process	Acute	Treatment	Effective	X - Female Genital	NA	1. A. Mazur, C. D. Brindis e M. D. J. , “Assessing youth-friendly sexual and reproductive health services: a systematic review,” BMC Health Services Research, pp. 1-12, 2018.
2	636	Pap smears and pregnancy tests	Process	Preventive	Screening and prevention	Effective	X - Female Genital	NA	1. A. Mazur, C. D. Brindis e M. D. J. , “Assessing youth-friendly sexual and reproductive health services: a systematic review,” BMC Health Services Research, pp. 1-12, 2018.
2	637	Hormonal contraceptive provision without appointment for pelvic exam	Process	Preventive	Treatment	Safe	X - Female Genital	Hormonal contraceptive provision without appointment for pelvic exam	1. A. Mazur, C. D. Brindis e M. D. J. , “Assessing youth-friendly sexual and reproductive health services: a systematic review,” BMC Health Services Research, pp. 1-12, 2018. 2. Henderson, J. T., Sawaya, G. F., Blum, M., Stratton, L., & Harper, C. C. (2010). Pelvic examinations and access to oral hormonal contraception. Obstetrics and gynecology, 116(6), 1257–1264. doi:10.1097/AOG.0b013e3181fb540f
2	638	Pregnant and parenting teen services	Structure	Preventive	Follow up and continuity	Effective	W - Pregnancy, Childbearing, Family Planning	Pregnant and parenting teen services	1. A. Mazur, C. D. Brindis e M. D. J. , “Assessing youth-friendly sexual and reproductive health services: a systematic review,” BMC Health Services Research, pp. 1-12, 2018.
15	639	Prescription of a combined hormonal contraceptive to a woman with a history of venous or arterial thromboembolism	Process	Preventive	Treatment	Safe	W - Pregnancy, Childbearing, Family Planning	Prescription of a combined hormonal contraceptive to a woman with a history of venous or arterial thromboembolism	1. R. Spencer, B. Bell, A. J Avery, G. Gookey e S. M Campbell, “Identification of an updated set of prescribing-safety indicators for GPs,” British Journal of General Practice, pp. e181-e190, 2014.
15	640	Prescription of oral or transdermal oestrogens to a woman with a history of breast cancer	Process	Preventive	Treatment	Safe	X - Female Genital	Prescription of oral or transdermal oestrogens to a woman with a history of breast cancer	1. R. Spencer, B. Bell, A. J Avery, G. Gookey e S. M Campbell, “Identification of an updated set of prescribing-safety indicators for GPs,” British Journal of General Practice, pp. e181-e190, 2014.
15	641	Prescription of oral or transdermal oestrogen without a progestogen in a woman with an intact uterus	Process	Preventive	Treatment	Safe	X - Female Genital	Prescription of oral or transdermal oestrogen without a progestogen in a woman with an intact uterus	1. R. Spencer, B. Bell, A. J Avery, G. Gookey e S. M Campbell, “Identification of an updated set of prescribing-safety indicators for GPs,” British Journal of General Practice, pp. e181-e190, 2014.
15	642	Prescription of a combined hormonal contraceptive to a woman aged ≥35 years who is a current smoker	Process	Preventive	Treatment	Safe	W - Pregnancy, Childbearing, Family Planning	Prescription of a combined hormonal contraceptive to a woman aged ≥35 years who is a current smoker	1. R. Spencer, B. Bell, A. J Avery, G. Gookey e S. M Campbell, “Identification of an updated set of prescribing-safety indicators for GPs,” British Journal of General Practice, pp. e181-e190, 2014.
15	643	Prescription of a combined hormonal contraceptive to a woman with a body mass index of ≥40	Process	Preventive	Treatment	Safe	W - Pregnancy, Childbearing, Family Planning	Prescription of a combined hormonal contraceptive to a woman with a body mass index of ≥40	1. R. Spencer, B. Bell, A. J Avery, G. Gookey e S. M Campbell, “Identification of an updated set of prescribing-safety indicators for GPs,” British Journal of General Practice, pp. e181-e190, 2014.
1	644	Adult health examination	Process	Preventive	Screening and prevention	Patient-centered	A - General and unspecified	Percentage of patients aged over 40 years utilizing adult health examination service	1. Jan, C.-F., Chiu, T.-Y., Chen, C.-Y., Guo, F.-R., & Lee, M.-C. (2017). A 10-year review of health care reform on Family Practice Integrated Care Project—Taiwan experience. Family Practice, 35(4), 352–357. // 2. Pan CH, Tung YC. The Effect of Family Physician Integrated Care Program on Healthcare Utilization and Outcomes. 2014. Master thesis. http://handle. ncl.edu.tw/11296/ndltd/90389048537140727816 (accessed on 14 August 2017). // 3. National Health Insurance Administration, Ministry of Health and Welfare, Taiwan. National Health Insurance 2015–2016 Annual Report. 2016; 38–9. // 4. Bodenheimer T, Wagner EH, Grumbach K. Improving primary care for patients with chronic illness: the chronic care model, Part 2. JAMA 2002; 288: 1909–14. // 5. WHO. Framework on integrated people-centered health services. 2015. http://www.who.int/servicedeliverysafety/areas/people-centred-care/en/ (accessed on 13 March 2017). // 6. Liau CY, Lin CC, Lin YK, Lin BY. Partnership disengagement from primary community care networks (PCCNs): a qualitative study for a national demonstration project. BMC Health Serv Res 2010; 10: 87.
1	645	Elderly Influenza Vaccination	Process	Preventive	Screening and prevention	Safe	A - General and unspecified	Percentage of patients older than 65 years receiving the annual influenza shot	1. Jan, C.-F., Chiu, T.-Y., Chen, C.-Y., Guo, F.-R., & Lee, M.-C. (2017). A 10-year review of health care reform on Family Practice Integrated Care Project—Taiwan experience. Family Practice, 35(4), 352–357. // 2. Pan CH, Tung YC. The Effect of Family Physician Integrated Care Program on Healthcare Utilization and Outcomes. 2014. Master thesis. http://handle. ncl.edu.tw/11296/ndltd/90389048537140727816 (accessed on 14 August 2017). // 3. National Health Insurance Administration, Ministry of Health and Welfare, Taiwan. National Health Insurance 2015–2016 Annual Report. 2016; 38–9. // 4. Bodenheimer T, Wagner EH, Grumbach K. Improving primary care for patients with chronic illness: the chronic care model, Part 2. JAMA 2002; 288: 1909–14. // 5. WHO. Framework on integrated people-centered health services. 2015. http://www.who.int/servicedeliverysafety/areas/people-centred-care/en/ (accessed on 13 March 2017). // 6. Liau CY, Lin CC, Lin YK, Lin BY. Partnership disengagement from primary community care networks (PCCNs): a qualitative study for a national demonstration project. BMC Health Serv Res 2010; 10: 87.
1	646	Pap smear rate	Process	Preventive	Screening and prevention	Effective	X - Female Genital	Percentage of sexually active female patients older than 30 years receiving Pap smear service for cervical cancer screening versus percentage in non-members	1. Jan, C.-F., Chiu, T.-Y., Chen, C.-Y., Guo, F.-R., & Lee, M.-C. (2017). A 10-year review of health care reform on Family Practice Integrated Care Project—Taiwan experience. Family Practice, 35(4), 352–357. // 2. Pan CH, Tung YC. The Effect of Family Physician Integrated Care Program on Healthcare Utilization and Outcomes. 2014. Master thesis. http://handle. ncl.edu.tw/11296/ndltd/90389048537140727816 (accessed on 14 August 2017). // 3. National Health Insurance Administration, Ministry of Health and Welfare, Taiwan. National Health Insurance 2015–2016 Annual Report. 2016; 38–9. // 4. Bodenheimer T, Wagner EH, Grumbach K. Improving primary care for patients with chronic illness: the chronic care model, Part 2. JAMA 2002; 288: 1909–14. // 5. WHO. Framework on integrated people-centered health services. 2015. http://www.who.int/servicedeliverysafety/areas/people-centred-care/en/ (accessed on 13 March 2017). // 6. Liau CY, Lin CC, Lin YK, Lin BY. Partnership disengagement from primary community care networks (PCCNs): a qualitative study for a national demonstration project. BMC Health Serv Res 2010; 10: 87.
1	647	Immunochemical faecal occult blood test (iFOBT)	Process	Preventive	Screening and prevention	Effective	D - Digestive	Percentage of patients aged over 50 years receiving the stool iFOBT for colon cancer screening	1. Jan, C.-F., Chiu, T.-Y., Chen, C.-Y., Guo, F.-R., & Lee, M.-C. (2017). A 10-year review of health care reform on Family Practice Integrated Care Project—Taiwan experience. Family Practice, 35(4), 352–357. // 2. Pan CH, Tung YC. The Effect of Family Physician Integrated Care Program on Healthcare Utilization and Outcomes. 2014. Master thesis. http://handle. ncl.edu.tw/11296/ndltd/90389048537140727816 (accessed on 14 August 2017). // 3. National Health Insurance Administration, Ministry of Health and Welfare, Taiwan. National Health Insurance 2015–2016 Annual Report. 2016; 38–9. // 4. Bodenheimer T, Wagner EH, Grumbach K. Improving primary care for patients with chronic illness: the chronic care model, Part 2. JAMA 2002; 288: 1909–14. // 5. WHO. Framework on integrated people-centered health services. 2015. http://www.who.int/servicedeliverysafety/areas/people-centred-care/en/ (accessed on 13 March 2017). // 6. Liau CY, Lin CC, Lin YK, Lin BY. Partnership disengagement from primary community care networks (PCCNs): a qualitative study for a national demonstration project. BMC Health Serv Res 2010; 10: 87.
1	648	Fixed Doctors	Process	Preventive	All	All	A - General and unspecified	Percentage of patients visiting family doctors in the same patient group	1. Jan, C.-F., Chiu, T.-Y., Chen, C.-Y., Guo, F.-R., & Lee, M.-C. (2017). A 10-year review of health care reform on Family Practice Integrated Care Project—Taiwan experience. Family Practice, 35(4), 352–357. // 2. Pan CH, Tung YC. The Effect of Family Physician Integrated Care Program on Healthcare Utilization and Outcomes. 2014. Master thesis. http://handle. ncl.edu.tw/11296/ndltd/90389048537140727816 (accessed on 14 August 2017). // 3. National Health Insurance Administration, Ministry of Health and Welfare, Taiwan. National Health Insurance 2015–2016 Annual Report. 2016; 38–9. // 4. Bodenheimer T, Wagner EH, Grumbach K. Improving primary care for patients with chronic illness: the chronic care model, Part 2. JAMA 2002; 288: 1909–14. // 5. WHO. Framework on integrated people-centered health services. 2015. http://www.who.int/servicedeliverysafety/areas/people-centred-care/en/ (accessed on 13 March 2017). // 6. Liau CY, Lin CC, Lin YK, Lin BY. Partnership disengagement from primary community care networks (PCCNs): a qualitative study for a national demonstration project. BMC Health Serv Res 2010; 10: 87.
2	649	Contraceptive services	Process	Preventive	Diagnosis	Patient-centered	A - General and unspecified / W - Pregnancy, Childbearing, Family Planning	Provides contraceptive services for users	1. Mazur, A., Brindis, C. D., & Decker, M. J. (2018). Assessing youth-friendly sexual and reproductive health services: a systematic review. BMC Health Services Research, 18(1). // 2. Geary RS, Webb EL, Clarke L, Norris SA. Evaluating youth-friendly health services: young people's perspectives from a simulated client study in urban South Africa. Glob Health Action. 2015;8:1–9 // 3. Mmari KN, Magnani RJ. Does making clinic-based reproductive health services more youth-friendly increase service use by adolescents? Evidence from Lusaka, Zambia. J Adolesc Health. 2003;33(4):259–70. // 4. McHome Z, Richards E, Nnko S, Dusabe J, Mapella E, Obasi A. A ‘mystery client’ evaluation of adolescent sexual and reproductive health services in health facilities from two regions in Tanzania. PLoS One. 2015;10(3):e0120822. // 5. Baumgartner JN, Otieno-Masaba R, Weaver MA, Grey TW, Reynolds HW. Service delivery characteristics associated with contraceptive use among youth clients in integrated voluntary counseling and HIV testing clinics in Kenya. Aids Care-Psychol Socio-Med Aspects Aids/Hiv. 2012;24(10):1290–301.
2	650	Sexual Counselling	Process	Preventive	Follow up and continuity	Effective	X - Female Genital	Provides sexual education / condom demonstration	1. Mazur, A., Brindis, C. D., & Decker, M. J. (2018). Assessing youth-friendly sexual and reproductive health services: a systematic review. BMC Health Services Research, 18(1). // 2. Brindis CD, Loo VS, Adler NE, Bolan GA, Wasserheit JN. Service integration and teen friendliness in practice: a program assessment of sexual and reproductive health services for adolescents. J Adolesc Health. 2005;37(2): 155–62. // 3. Dickson KE, Ashton J, Smith JM. Does setting adolescent-friendly standards improve the quality of care in clinics? Evidence from South Africa. Int J Qual Health C. 2007;19(2):80–9. // 4. Geary RS, Gomez-Olive FX, Kahn K, Tollman S, Norris SA. Barriers to and facilitators of the provision of a youth-friendly health services programme in rural South Africa. BMC Health Serv Res. 2014;14:259. // 5. Mathews C, Guttmacher SJ, Hisher AJ, Mtshizana YY, Nelson T, McCarthy J, Daries V. The quality of HIV testing Services for Adolescents in cape town, South Africa: do adolescent-friendly services make a difference? J Adolesc Health. 2009;44(2):188–90. // 6. Lesedi C, Hoque ME, Ntuli-Ngcobo B. Investigating user-friendliness of the sexual and reproductive health services among youth in Botswana. Se Asian J Trop Med. 2011;42(6):1431–43. // 7. Mayeye FB, Lewis HA, Oguntibeju OO. An assessment of adolescent satisfaction with reproductive primary healthcare Services in the Eastern Cape Province, South Africa. W Indian Med J. 2010;59(3):274–9. // 8.Thomée S, Malm D, Christianson M, Hurtig A-K, Wiklund M, Waenerlund A-K, Goicolea I. Challenges and strategies for sustaining youth-friendly health services — a qualitative study from the perspective of professionals at youth clinics in northern Sweden. Reprod Health. 2016;13:147.
2	651	Sexual Counselling: Pregnant and parenting teen services	Process	Preventive	Screening and prevention	Effective	W - Pregnancy, Childbearing, Family Planning	Provides Pregnant and parenting teen services	1. Mazur, A., Brindis, C. D., & Decker, M. J. (2018). Assessing youth-friendly sexual and reproductive health services: a systematic review. BMC Health Services Research, 18(1). // 2. Bensussen-Walls W, Saewyc EM. Teen-focused care versus adult-focused care for the high-risk pregnant adolescent: an outcomes evaluation. Public Health Nurs. 2001;18(6):424–35.
2	652	Sexual Counselling: Reproductive and sexual transmited disease test results	Process	Preventive	Screening and prevention	Effective	W - Pregnancy, Childbearing, Family Planning	Promote sexual counselling on reproductive and sexual transmited disease test results	1. Mazur, A., Brindis, C. D., & Decker, M. J. (2018). Assessing youth-friendly sexual and reproductive health services: a systematic review. BMC Health Services Research, 18(1). // 2. Dehne KL, Riedner G. Sexually transmitted infections among adolescents: the need for adequate health services. Geneva: World Health Organization; 2005. // 3. Newton-Levinson A, Leichliter JS, Chandra-Mouli V. Sexually transmitted infection Services for Adolescents and Youth in low- and middle-incomecountries: perceived and experienced barriers to accessing care. J Adolesc Health. 2016;59(1):7–16.
2	653	Supplies available onsite (medical testing)	Process	Preventive	Screening and prevention	Effective	X - Female Genital	Supplies available onsite (medical testing)	1. Mazur, A., Brindis, C. D., & Decker, M. J. (2018). Assessing youth-friendly sexual and reproductive health services: a systematic review. BMC Health Services Research, 18(1). // 2. Dickson KE, Ashton J, Smith JM. Does setting adolescent-friendly standards improve the quality of care in clinics? Evidence from South Africa. Int J Qual Health C. 2007;19(2):80–9. // 3. Godia PM, Olenja JM, Hofman JJ, van den Broek N. Young people's perception of sexual and reproductive health services in Kenya. BMC Health Serv Res. 2014;14:172. // 4. Larke N, Cleophas-Mazige B, Plummer ML, Obasi AI, Rwakatare M, Todd J, Changalucha J, Weiss HA, Hayes RJ, Ross DA. Impact of the MEMA kwa Vijana adolescent sexual and reproductive health interventions on use of health services by young people in rural Mwanza, Tanzania: results of a cluster randomized trial. J Adolesc Health. 2010;47(5):512–22. // 5. McHome Z, Richards E, Nnko S, Dusabe J, Mapella E, Obasi A. A ‘mystery client’ evaluation of adolescent sexual and reproductive health services in health facilities from two regions in Tanzania. PLoS One. 2015;10(3):e0120822. // 6. Lesedi C, Hoque ME, Ntuli-Ngcobo B. Investigating user-friendliness of the sexual and reproductive health services among youth in Botswana. Se Asian J Trop Med. 2011;42(6):1431–43. // 7. Mayeye FB, Lewis HA, Oguntibeju OO. An assessment of adolescent satisfaction with reproductive primary healthcare Services in the Eastern Cape Province, South Africa. W Indian Med J. 2010;59(3):274–9.
2	654	Providers are medically competent	Process	Preventive	All	Effective	Not Defined	Providers are medically competent	1. Mazur, A., Brindis, C. D., & Decker, M. J. (2018). Assessing youth-friendly sexual and reproductive health services: a systematic review. BMC Health Services Research, 18(1). // 2. Godia PM, Olenja JM, Hofman JJ, van den Broek N. Young people's perception of sexual and reproductive health services in Kenya. BMC Health Serv Res. 2014;14:172. // 3. Lesedi C, Hoque ME, Ntuli-Ngcobo B. Investigating user-friendliness of the sexual and reproductive health services among youth in Botswana. Se Asian J Trop Med. 2011;42(6):1431–43.
2	655	Infection control procedures are followed	Process	Preventive	Follow up and continuity	Effective	Not Defined	Infection control procedures followed	1. Mazur, A., Brindis, C. D., & Decker, M. J. (2018). Assessing youth-friendly sexual and reproductive health services: a systematic review. BMC Health Services Research, 18(1). // 2. Newton-Levinson A, Leichliter JS, Chandra-Mouli V. Sexually transmitted infection Services for Adolescents and Youth in low- and middle-income countries: perceived and experienced barriers to accessing care. J Adolesc Health. 2016;59(1):7–16. // 3. Dehne KL, Riedner G. Sexually transmitted infections among adolescents: the need for adequate health services. Geneva: World Health Organization; 2005.
2	656	Plan for follow up care explained and scheduled	Process	Preventive	Follow up and continuity	Patient-centered	Not Defined	Dispose a plan for follow up and explain/scheduled	1. Mazur, A., Brindis, C. D., & Decker, M. J. (2018). Assessing youth-friendly sexual and reproductive health services: a systematic review. BMC Health Services Research, 18(1). // 2. Brittain AW, Williams JR, Zapata LB, Pazol K, Romero LM, Weik TS. Youthfriendly family planning services for young people: a systematic review. Am J Prev Med. 2015;49(2):S73–84. // 3. Kavanaugh ML, Jerman J, Ethier K, Moskosky S. Meeting the contraceptive needs of teens and young adults: youth-friendly and long-acting reversible contraceptive Services in U.S. Family planning facilities. J Adolesc Health. 2013;52(3):284–92. // 4. Mayeye FB, Lewis HA, Oguntibeju OO. An assessment of adolescent satisfaction with reproductive primary healthcare Services in the Eastern Cape Province, South Africa. W Indian Med J. 2010;59(3):274–9.
2	657	Referral care	Process	Chronic	Follow up and continuity	Effective	A - General and unspecified	Referral care available, explained, and scheduled	1. Mazur, A., Brindis, C. D., & Decker, M. J. (2018). Assessing youth-friendly sexual and reproductive health services: a systematic review. BMC Health Services Research, 18(1). // 2. Senderwoitz C, Solter C, Hainsworth G. Clinic assessment of youth friendly services: a tool for assessing and improving reproductive health services for youth. Watertown: Pathfinder International; 2002. // 3. Dickson KE, Ashton J, Smith JM. Does setting adolescent-friendly standards improve the quality of care in clinics? Evidence from South Africa. Int J Qual Health C. 2007;19(2):80–9. // 4. Geary RS, Gomez-Olive FX, Kahn K, Tollman S, Norris SA. Barriers to and facilitators of the provision of a youth-friendly health services programme in rural South Africa. BMC Health Serv Res. 2014;14:259. // 5. Mathews C, Guttmacher SJ, Hisher AJ, Mtshizana YY, Nelson T, McCarthy J, Daries V. The quality of HIV testing Services for Adolescents in cape town, South Africa: do adolescent-friendly services make a difference? J Adolesc Health. 2009;44(2):188–90. // 6. Tanner AE, Philbin MM, Duval A, Ellen J, Kapogiannis B, Fortenberry JD, Adolescent Trials Network for HIVAI. “youth friendly” clinics: considerations for linking and engaging HIV-infected adolescents into care. AIDS Care. 2014;26(2):199–205. // 7. Sovd T, Mmari K, Lipovsek V, Manaseki-Holland S. Acceptability as a key determinant of client satisfaction: lessons from an evaluation of adolescent friendly health services in Mongolia. J Adolesc Health. 2006;38(5):519–26. // 8. Lesedi C, Hoque ME, Ntuli-Ngcobo B. Investigating user-friendliness of the sexual and reproductive health services among youth in Botswana. Se Asian J Trop Med. 2011;42(6):1431–43. // 9.. Mayeye FB, Lewis HA, Oguntibeju OO. An assessment of adolescent satisfaction with reproductive primary healthcare Services in the Eastern Cape Province, South Africa. W Indian Med J. 2010;59(3):274–9.
2	658	Sufficient time for consultation	Process	All	All	Effective	Not Defined	Sufficient time for consultation . Not less than 15 minutes for consultation time	1. Mazur, A., Brindis, C. D., & Decker, M. J. (2018). Assessing youth-friendly sexual and reproductive health services: a systematic review. BMC Health Services Research, 18(1). // 2. World Health Organization. Global consultation on adolescent friendly health services: a consensus statement. Geneva: World Health Organization; 2001. // 3. Speizer IS, Magnani RJ, Colvin CE. The effectiveness of adolescent reproductive health interventions in developing countries: a review of the evidence. J Adolesc Health. 2003;33(5):324–48.
2	659	Adequate information from provider	Process	All	All	Effective	Not Defined	Staff characteristics and compentency: Client recieves adequate and non judgemental information from provider	1. Mazur, A., Brindis, C. D., & Decker, M. J. (2018). Assessing youth-friendly sexual and reproductive health services: a systematic review. BMC Health Services Research, 18(1). // 2. Chandra-Mouli V, Chatterjee S, Bose K. Do efforts to standardize, assess and improve the quality of health service provision to adolescents by government-run health services in low and middle income countries, lead to improvements in service-quality and service-utilization by adolescents? Reprod Health. 2016;13:10. // 3. Baumgartner JN, Otieno-Masaba R, Weaver MA, Grey TW, Reynolds HW. Service delivery characteristics associated with contraceptive use among youth clients in integrated voluntary counseling and HIV testing clinics in Kenya. Aids Care-Psychol Socio-Med Aspects Aids/Hiv. 2012;24(10): 1290–301. // 4. Brindis CD, Loo VS, Adler NE, Bolan GA, Wasserheit JN. Service integration and teen friendliness in practice: a program assessment of sexual and reproductive health services for adolescents. J Adolesc Health. 2005;37(2): 155–62. // 5. Dickson KE, Ashton J, Smith JM. Does setting adolescent-friendly standards improve the quality of care in clinics? Evidence from South Africa. Int J Qual Health C. 2007;19(2):80–9.
2	660	Comfort in communicating	Process	All	All	Patient-centered	Not Defined	Staff characteristics and compentency: Comfort in communicating	1. Mazur, A., Brindis, C. D., & Decker, M. J. (2018). Assessing youth-friendly sexual and reproductive health services: a systematic review. BMC Health Services Research, 18(1). // 2. Dickson KE, Ashton J, Smith JM. Does setting adolescent-friendly standards improve the quality of care in clinics? Evidence from South Africa. Int J Qual Health C. 2007;19(2):80–9. // 3. Geary RS, Webb EL, Clarke L, Norris SA. Evaluating youth-friendly health services: young people's perspectives from a simulated client study in urban South Africa. Glob Health Action. 2015;8:1–9. // 4. Ingram J, Salmon D. No worries! Young people’s experiences of nurseled drop-in sexual health services in south West England. J Res Nurs. 2007;12(4):305–15. // 5. McHome Z, Richards E, Nnko S, Dusabe J, Mapella E, Obasi A. A ‘mystery client’ evaluation of adolescent sexual and reproductive health services in health facilities from two regions in Tanzania. PLoS One. 2015;10(3):e0120822. // 6. Mauerhofer A, Berchtold A, Akre C, Michaud PA, Suris JC. Female adolescents’ views on a youth-friendly clinic. Swiss Med Weekly. 2010;140(1–2):18–23.
2	661	Privacy and Confidentiality	Process	All	All	Patient-centered	Not Defined	Client consultation cannot be heard or seen by other clients or staff	1. Mazur, A., Brindis, C. D., & Decker, M. J. (2018). Assessing youth-friendly sexual and reproductive health services: a systematic review. BMC Health Services Research, 18(1). // 2. Kavanaugh ML, Jerman J, Ethier K, Moskosky S. Meeting the contraceptive needs of teens and young adults: youth-friendly and long-acting reversible contraceptive Services in U.S. Family planning facilities. J Adolesc Health. 2013;52(3):284–92. // 3. Mathews C, Guttmacher SJ, Hisher AJ, Mtshizana YY, Nelson T, McCarthy J, Daries V. The quality of HIV testing Services for Adolescents in cape town, South Africa: do adolescent-friendly services make a difference? J Adolesc Health. 2009;44(2):188–90. // 4. Mashamba A, Robson E. Youth reproductive health services in Bulawayo, Zimbabwe. Health Place. 2002;8(4):273–83. // 5. Perry C, Thurston A. Meeting the sexual health care needs of young people: a model that works? Child Care Hlth Dev. 2008;34(1):98–103. // 6. Godia PM, Olenja JM, Hofman JJ, van den Broek N. Young people's perception of sexual and reproductive health services in Kenya. BMC Health Serv Res. 2014;14:172. // 7. Mathews C, Guttmacher SJ, Hisher AJ, Mtshizana YY, Nelson T, McCarthy J, Daries V. The quality of HIV testing Services for Adolescents in cape town, South Africa: do adolescent-friendly services make a difference? J Adolesc Health. 2009;44(2):188–90. // 8. Tanner AE, Philbin MM, Duval A, Ellen J, Kapogiannis B, Fortenberry JD, Adolescent Trials Network for HIVAI. “youth friendly” clinics: considerations for linking and engaging HIV-infected adolescents into care. AIDS Care. 2014;26(2):199–205.

2	662	Follow up by the same clinician	Process	Chronic	Follow up and continuity	Patient-centered	Not Defined	Choice and availability to be seen with same clinician during return visit	1. Mazur, A., Brindis, C. D., & Decker, M. J. (2018). Assessing youth-friendly sexual and reproductive health services: a systematic review. BMC Health Services Research, 18(1). // 2. Kavanaugh ML, Jerman J, Ethier K, Moskosky S. Meeting the contraceptive needs of teens and young adults: youth-friendly and long-acting reversible contraceptive Services in U.S. Family planning facilities. J Adolesc Health. 2013;52(3):284–92. // 3. Alli F, Maharaj P, Vawda MY. Interpersonal relations between health care workers and young clients: barriers to accessing sexual and reproductive health care. J Community Health. 2013;38(1):150–5. // 4. Godia PM, Olenja JM, Hofman JJ, van den Broek N. Young people's perception of sexual and reproductive health services in Kenya. BMC Health Serv Res. 2014;14:172. // 5. Mauerhofer A, Berchtold A, Akre C, Michaud PA, Suris JC. Female adolescents' views on a youth-friendly clinic. Swiss Med Weekly. 2010;140(1–2):18–23. // 6. Mayeye FB, Lewis HA, Oguntibeju OO. An assessment of adolescent satisfaction with reproductive primary healthcare Services in the Eastern Cape Province, South Africa. W Indian Med J. 2010;59(3):274–9.
2	663	Passive disclosure of services avoided	Process	Preventive	All	Patient-centered	Not Defined	Being seen in the waiting room discloses reason client is seeking service	1. Mazur, A., Brindis, C. D., & Decker, M. J. (2018). Assessing youth-friendly sexual and reproductive health services: a systematic review. BMC Health Services Research, 18(1). // 2. Mashamba A, Robson E. Youth reproductive health services in Bulawayo, Zimbabwe. Health Place. 2002;8(4):273–83. // 3. Tanner AE, Philbin MM, Duval A, Ellen J, Kapogiannis B, Fortenberry JD, Adolescent Trials Network for HIVAI. "youth friendly" clinics: considerations for linking and engaging HIV-infected adolescents into care. AIDS Care. 2014;26(2):199–205. // 4. Perry C, Thurston A. Meeting the sexual health care needs of young people: a model that works? Child Care Hlth Dev. 2008;34(1):98–103.
2	664	Reproductive and sexual infection disease tests	Process	Preventive	Screening and prevention	Effective	W - Pregnancy, Childbearing, Family Planning	Reproductive and sexual infection disease tests are handled confidentialy	1. Mazur, A., Brindis, C. D., & Decker, M. J. (2018). Assessing youth-friendly sexual and reproductive health services: a systematic review. BMC Health Services Research, 18(1). // 2. Brindis CD, Loo VS, Adler NE, Bolan GA, Wasserheit JN. Service integration and teen friendliness in practice: a program assessment of sexual and reproductive health services for adolescents. J Adolesc Health. 2005;37(2): 155–62. // Dickson KE, Ashton J, Smith JM. Does setting adolescent-friendly standards improve the quality of care in clinics? Evidence from South Africa. Int J Qual Health C. 2007;19(2):80–9. // 3. Geary RS, Gomez-Olive FX, Kahn K, Tollman S, Norris SA. Barriers to and facilitators of the provision of a youth-friendly health services programme in rural South Africa. BMC Health Serv Res. 2014;14:259. // 4. McHome Z, Richards E, Nnko S, Dusabe J, Mapella E, Obasi A. A 'mystery client' evaluation of adolescent sexual and reproductive health services in health facilities from two regions in Tanzania. PLoS One. 2015;10(3):e0120822. // 5. Lesedi C, Hoque ME, Ntuli-Ngcobo B. Investigating user-friendliness of the sexual and reproductive health services among youth in Botswana. Se Asian J Trop Med. 2011;42(6):1431–43. // 6. Mauerhofer A, Berchtold A, Akre C, Michaud PA, Suris JC. Female adolescents' views on a youth-friendly clinic. Swiss Med Weekly. 2010;140(1–2):18–23. // 7. Mayeye FB, Lewis HA, Oguntibeju OO. An assessment of adolescent satisfaction with reproductive primary healthcare Services in the Eastern Cape Province, South Africa. W Indian Med J. 2010;59(3):274–9.
2	665	Text message for follow-up or education	Process	Preventive	Follow up and continuity	Patient-centered	Not Defined	Text message for follow-up or education	1. Mazur, A., Brindis, C. D., & Decker, M. J. (2018). Assessing youth-friendly sexual and reproductive health services: a systematic review. BMC Health Services Research, 18(1). // 2. Dickson KE, Ashton J, Smith JM. Does setting adolescent-friendly standards improve the quality of care in clinics? Evidence from South Africa. Int J Qual Health C. 2007;19(2):80–9. // 3. World Health Organization. Quality assessment guidebook: a guide to assessing health services for adolescent clients. Geneva: World Health Organization; 2009. // Mashamba A, Robson E. Youth reproductive health services in Bulawayo, Zimbabwe. Health Place. 2002;8(4):273–83. // Godia PM, Olenja JM, Hofman JJ, van den Broek N. Young people's perception of sexual and reproductive health services in Kenya. BMC Health Serv Res. 2014;14:172. // 4. Tanner AE, Philbin MM, Duval A, Ellen J, Kapogiannis B, Fortenberry JD Adolescent Trials Network for HIVAI. "youth friendly" clinics: considerations for linking and engaging HIV-infected adolescents into care. AIDS Care. 2014;26(2):199–205. // 5. Mauerhofer A, Berchtold A, Akre C, Michaud PA, Suris JC. Female adolescents' views on a youth-friendly clinic. Swiss Med Weekly. 2010;140(1–2):18–23. // 6. Mayeye FB, Lewis HA, Oguntibeju OO. An assessment of adolescent satisfaction with reproductive primary healthcare Services in the Eastern Cape Province, South Africa. W Indian Med J. 2010;59(3):274–9.
2	666	Waiting times in Youth-Friendly Services	Process	Preventive	All	Timely	Not Defined	Waiting delay for attendence	Mazur, A., Brindis, C. D., & Decker, M. J. (2018). Assessing youth-friendly sexual and reproductive health services: a systematic review. BMC Health Services Research, 18(1). // Ingram J, Salmon D. No worries! Young people's experiences of nurseled drop-in sexual health services in south West England. J Res Nurs. 2007;12(4):305–15 // Mashamba A, Robson E. Youth reproductive health services in Bulawayo, Zimbabwe. Health Place. 2002;8(4):273–83. // Godia PM, Olenja JM, Hofman JJ, van den Broek N. Young people's perception of sexual and reproductive health services in Kenya. BMC Health Serv Res. 2014;14:172. // Mathews C, Guttmacher SJ, Hisher AJ, Mtshizana YY, Nelson T, McCarthy J, Daries V. The quality of HIV testing Services for Adolescents in cape town, South Africa: do adolescent-friendly services make a difference? J Adolesc Health. 2009;44(2):188–90. // Sovd T, Mmari K, Lipovsek V, Manaseki-Holland S. Acceptability as a key determinant of client satisfaction: lessons from an evaluation of adolescent friendly health services in Mongolia. J Adolesc Health. 2006;38(5):519–26 // Baumgartner JN, Otieno-Masaba R, Weaver MA, Grey TW, Reynolds HW. Service delivery characteristics associated with contraceptive use among youth clients in integrated voluntary counseling and HIV testing clinics in Kenya. Aids Care-Psychol Socio-Med Aspects Aids/Hiv. 2012;24(10): 1290–301. // Mauerhofer A, Berchtold A, Akre C, Michaud PA, Suris JC. Female adolescents' views on a youth-friendly clinic. Swiss Med Weekly. 2010;140(1–2):18–23.
2	667	Holistic approach	Process	Preventive	All	Patient-centered	Not Defined	Services available beyond reproductive health including mental, psychosocial, lifeskills etc	1. Mazur, A., Brindis, C. D., & Decker, M. J. (2018). Assessing youth-friendly sexual and reproductive health services: a systematic review. BMC Health Services Research, 18(1). // 2. Patton MQ. Qualitative evaluation and research methods. Newbury Park: SAGE Publications; 1990. // 3. Senderwoitz C, Solter C, Hainsworth G. Clinic assessment of youth friendly services: a tool for assessing and improving reproductive health services for youth. Watertown: Pathfinder International; 2002.
2	668	Non-health services	Process	Preventive	All	Effective	Not Defined	Youth development services, domestic violence	1. Mazur, A., Brindis, C. D., & Decker, M. J. (2018). Assessing youth-friendly sexual and reproductive health services: a systematic review. BMC Health Services Research, 18(1). // 2. Patton MQ. Qualitative evaluation and research methods. Newbury Park: SAGE Publications; 1990. // 3. Senderwoitz C, Solter C, Hainsworth G. Clinic assessment of youth friendly services: a tool for assessing and improving reproductive health services for youth. Watertown: Pathfinder International; 2002.
2	669	General satisfaction	Outcome	Preventive	Follow up and continuity	Patient-centered	Not Defined	Patient Satisfaction	1. Mazur, A., Brindis, C. D., & Decker, M. J. (2018). Assessing youth-friendly sexual and reproductive health services: a systematic review. BMC Health Services Research, 18(1). // 2. Mayeye FB, Lewis HA, Oguntibeju OO. An assessment of adolescent satisfaction with reproductive primary healthcare Services in the Eastern Cape Province, South Africa. W Indian Med J. 2010;59(3):274–9. // 3. Ingram J, Salmon D. No worries! Young people's experiences of nurseled drop-in sexual health services in south West England. J Res Nurs. 2007;12(4):305–15. // 4. Mayeye FB, Lewis HA, Oguntibeju OO. An assessment of adolescent satisfaction with reproductive primary healthcare Services in the Eastern Cape Province, South Africa. W Indian Med J. 2010;59(3):274–9. // 5. Perry C, Thurston A. Meeting the sexual health care needs of young people: a model that works? Child Care Hlth Dev. 2008;34(1):98–103. // 6. Sovd T, Mmari K, Lipovsek V, Manaseki-Holland S. Acceptability as a key determinant of client satisfaction: lessons from an evaluation of adolescent friendly health services in Mongolia. J Adolesc Health. 2006;38(5):519–26.
2	670	Sexually transmitted infections services	Structure	Preventive	Diagnosis	Effective	X - Female Genital / Y - Male Genital	Provision of Sexually Transmitted Infection Services (counselling, testing, treatment and prevention)	1. Mazur, A., Brindis, C. D., & Decker, M. J. (2018). Assessing youth-friendly sexual and reproductive health services: a systematic review. BMC Health Services Research, 18(1). // 2. Newton-Levinson A, Leichliter JS, Chandra-Mouli V. Sexually transmitted infection Services for Adolescents and Youth in low- and middle-income countries: perceived and experienced barriers to accessing care. J Adolesc Health. 2016;59(1):7–16. // 3. Dehne KL, Riedner G. Sexually transmitted infections among adolescents: the need for adequate health services. Geneva: World Health Organization; 2005.
2	671	Voluntary counseling and testing available / HIV services	Process	Preventive	Screening and prevention	Patient-centered	X - Female Genital / Y - Male Genital	Voluntary counseling and testing available/HIV services	Mazur, A., Brindis, C. D., & Decker, M. J. (2018). Assessing youth-friendly sexual and reproductive health services: a systematic review. BMC Health Services Research, 18(1). // Baumgartner JN, Otieno-Masaba R, Weaver MA, Grey TW, Reynolds HW. Service delivery characteristics associated with contraceptive use among youth clients in integrated voluntary counseling and HIV testing clinics in Kenya. Aids Care-Psychol Socio-Med Aspects Aids/Hiv. 2012;24(10): 1290–301.
3	672	Primary care-supportive governmental policies for delivery of preventive care	Process	Preventive	Screening and prevention	All	Not Defined	Improvement of access of care, continuity and coordination of care and delivery of preventive care	1. Kringos, D. S., Boerma, W. G., Hutchinson, A., van der Zee, J., & Groenewegen, P. P. (2010). The breadth of primary care: a systematic literature review of its core dimensions. BMC Health Services Research, 10(1) //
3	673	Coordination of Care	Process	Preventive	Follow up and continuity	Patient-centered	Not Defined	The coordination of care dimension reflects the ability of primary care providers to coordinate use of other levels of health care (Gatekeeping system, Primary care practice and team structure, Skill-mix of primary care providers, Integration of primary care-secondary care, Integration of primary care and public health)	1. Kringos, D. S., Boerma, W. G., Hutchinson, A., van der Zee, J., & Groenewegen, P. P. (2010). The breadth of primary care: a systematic literature review of its core dimensions. BMC Health Services Research, 10(1) //
3	674	Longitudinal continuity of care	Process	Preventive	Follow up and continuity	Patient-centered	Not Defined	Having a long-term relationship between primary care providers and their patients in their practice beyond specific episodes of disease or illness, and the quality of the longitudinal relationship between primary care providers and patients	1. Kringos, D. S., Boerma, W. G., Hutchinson, A., van der Zee, J., & Groenewegen, P. P. (2010). The breadth of primary care: a systematic literature review of its core dimensions. BMC Health Services Research, 10(1) // 2. Macinko J, Starfield B, Shi L: The contribution of primary care systems to health outcomes within Organization for Economic Cooperation and Development (OECD) countries, 1970-1998. Health Serv Res 2003, 38:831-865. // 3. Starfield B, Shi L, Macinko J: Contribution of primary care to health systems and health. Milbank Q 2005, 83:457-502. // 4. Ashworth M, Armstrong D: The relationship between general practice characteristics and quality of care: a national survey of quality indicators used in the UK Quality and Outcomes Framework, 2004-5. BMC Fam Pract 2006, 7:68. // 5. Bower P, Roland M, Campbell J, Mead N: Setting standards based on patients' views on access and continuity: secondary analysis of data from the general practice assessment survey. BMJ 2003, 326:258. // 6. Christakis DA, Wright JA, Zimmerman FJ, Bassett AL, Connell FA: Continuity of care is associated with well-coordinated care. Ambul Pediatr 2003, 3:82-86. // 7. Grytten J, Sorensen R: Primary Physician Services–List Size and Primary Physicians' Service Production. J Health Econ 2007, 26:721-741. // 8. Naithani S, Gulliford M, Morgan M: Patients' perceptions and experiences of 'continuity of care' in diabetes. Health Expect 2006, 9:118-129. // 9. Nutting PA, Goodwin MA, Flocke SA, Zyzanski SJ, Stange KC: Continuity of primary care: to whom does it matter and when? Ann Fam Med 2003, 1:149-155. // 10. Parkerton PH, Smith DG, Straley HL: Primary care practice coordination versus physician continuity. Fam Med 2004, 36:15-21. // 11. Shi L, Starfield B, Xu J, Politzer R, Regan J: Primary care quality: community health center and health maintenance organization. South Med J 2003, 96:787-795. // 12. Stokes T, Tarrant C, Mainous AG III, Schers H, Freeman G, Baker R: Continuity of care: Is the personal doctor still important? A survey of general practitioners and family physicians in England and Wales, the United States, and the Netherlands. Ann Fam Med 2005, 3:353-359. // 13. Cabana MD, Jee SH: Does continuity of care improve patient outcomes? J Fam Pract 2004, 53:974-980. // 14. Jee SH, Cabana MD: Indices for continuity of care: a systematic review of the literature. Medical Care Research & Review 2006, 63:158-188.// 15. Saultz JW: Defining and measuring interpersonal continuity of care. Ann Fam Med 2003, 1:134-143. // 16. van Servellen G, Fongwa M, Mockus D'Errico E: Continuity of care andquality care outcomes for people experiencing chronic conditions: A literature review. Nurs Health Sci 2006, 8:185-195. // 17. van Servellen G, Fongwa M, Mockus D'Errico E: Continuity of care and quality care outcomes for people experiencing chronic conditions: A literature review. Nurs Health Sci 2006, 8:185-195.
3	675	Prescribing behaviour of primary care providers: Quality of prescriptions standard	Process	Chronic	Treatment	Safe	Not Defined	Prescriptions follow standards	1. Kringos, D. S., Boerma, W. G., Hutchinson, A., van der Zee, J., & Groenewegen, P. P. (2010). The breadth of primary care: a systematic literature review of its core dimensions. BMC Health Services Research, 10(1) // Gene-Badia, J., Ascaso, C., Escaramis-Babiano, G., Sampietro-Colom, L., Catalan-Ramos, A., Sans-Corrales, M., & Pujol-Ribera, E. (2006). Personalised care, access, quality and team coordination are the main dimensions of family medicine output. Family Practice, 24(1), 41–47. //
3	676	Prescribing behaviour of primary care providers: Anti-depressants prescribed: % of the recommended	Process	Chronic	Treatment	Effective	P - Psychological	Anti-depressants prescribed: % of the recommended	1. Kringos, D. S., Boerma, W. G., Hutchinson, A., van der Zee, J., & Groenewegen, P. P. (2010). The breadth of primary care: a systematic literature review of its core dimensions. BMC Health Services Research, 10(1) // Gene-Badia, J., Ascaso, C., Escaramis-Babiano, G., Sampietro-Colom, L., Catalan-Ramos, A., Sans-Corrales, M., & Pujol-Ribera, E. (2006). Personalised care, access, quality and team coordination are the main dimensions of family medicine output. Family Practice, 24(1), 41–47. //
3	677	Prescribing behaviour of primary care providers: Tranquilisers prescribed: % of the recommended	Process	Chronic	Treatment	Effective	P - Psychological	Tranquilisers prescribed: % of the recommended	1. Kringos, D. S., Boerma, W. G., Hutchinson, A., van der Zee, J., & Groenewegen, P. P. (2010). The breadth of primary care: a systematic literature review of its core dimensions. BMC Health Services Research, 10(1) // Gene-Badia, J., Ascaso, C., Escaramis-Babiano, G., Sampietro-Colom, L., Catalan-Ramos, A., Sans-Corrales, M., & Pujol-Ribera, E. (2006). Personalised care, access, quality and team coordination are the main dimensions of family medicine output. Family Practice, 24(1), 41–47. //
3	678	Prescribing behaviour of primary care providers: Anti-hypertensive medications prescribed: % of the recommended	Process	Chronic	Treatment	Effective	K - Cardiovascular	Anti-hypertensive medications prescribed: % of the recommended	1. Kringos, D. S., Boerma, W. G., Hutchinson, A., van der Zee, J., & Groenewegen, P. P. (2010). The breadth of primary care: a systematic literature review of its core dimensions. BMC Health Services Research, 10(1) // Gene-Badia, J., Ascaso, C., Escaramis-Babiano, G., Sampietro-Colom, L., Catalan-Ramos, A., Sans-Corrales, M., & Pujol-Ribera, E. (2006). Personalised care, access, quality and team coordination are the main dimensions of family medicine output. Family Practice, 24(1), 41–47. //
3	679	Prescribing behaviour of primary care providers: Anti-diabetes medications prescribed: % of the recommended	Process	Chronic	Treatment	Effective	T - Endocrine/Metabolic and Nutritional	Anti-diabetes medications prescribed: % of the recommended	1. Kringos, D. S., Boerma, W. G., Hutchinson, A., van der Zee, J., & Groenewegen, P. P. (2010). The breadth of primary care: a systematic literature review of its core dimensions. BMC Health Services Research, 10(1) // Gene-Badia, J., Ascaso, C., Escaramis-Babiano, G., Sampietro-Colom, L., Catalan-Ramos, A., Sans-Corrales, M., & Pujol-Ribera, E. (2006). Personalised care, access, quality and team coordination are the main dimensions of family medicine output. Family Practice, 24(1), 41–47. //
3	680	Prescribing behaviour of primary care providers: Anti-asthma medications prescribed: % of the recommended	Process	Chronic	Treatment	Effective	R - Respiratory	Anti-asthma medications prescribed: % of the recommended	1. Kringos, D. S., Boerma, W. G., Hutchinson, A., van der Zee, J., & Groenewegen, P. P. (2010). The breadth of primary care: a systematic literature review of its core dimensions. BMC Health Services Research, 10(1) // Gene-Badia, J., Ascaso, C., Escaramis-Babiano, G., Sampietro-Colom, L., Catalan-Ramos, A., Sans-Corrales, M., & Pujol-Ribera, E. (2006). Personalised care, access, quality and team coordination are the main dimensions of family medicine output. Family Practice, 24(1), 41–47. //
3	681	Relationship between user and health care professional: Adult visits	Process	Chronic	All	Patient-centered	A - General and unspecified	Addressing the social and psychological problems	1. Kringos, D. S., Boerma, W. G., Hutchinson, A., van der Zee, J., & Groenewegen, P. P. (2010). The breadth of primary care: a systematic literature review of its core dimensions. BMC Health Services Research, 10(1) // Gene-Badia, J., Ascaso, C., Escaramis-Babiano, G., Sampietro-Colom, L., Catalan-Ramos, A., Sans-Corrales, M., & Pujol-Ribera, E. (2006). Personalised care, access, quality and team coordination are the main dimensions of family medicine output. Family Practice, 24(1), 41–47. //
3	682	Relationship between user and health care professional: Time of dedication	Process	Chronic	Screening and prevention	Patient-centered	Not Defined	Addressing the social and psychological problems	1. Kringos, D. S., Boerma, W. G., Hutchinson, A., van der Zee, J., & Groenewegen, P. P. (2010). The breadth of primary care: a systematic literature review of its core dimensions. BMC Health Services Research, 10(1) // Gene-Badia, J., Ascaso, C., Escaramis-Babiano, G., Sampietro-Colom, L., Catalan-Ramos, A., Sans-Corrales, M., & Pujol-Ribera, E. (2006). Personalised care, access, quality and team coordination are the main dimensions of family medicine output. Family Practice, 24(1), 41–47. //
3	683	Quality of diagnosis and treatment in primary care	Process	All	Diagnosis / Treatment	Effective	Not Defined	Right diagnosis and right treatment	1. Kringos, D. S., Boerma, W. G., Hutchinson, A., van der Zee, J., & Groenewegen, P. P. (2010). The breadth of primary care: a systematic literature review of its core dimensions. BMC Health Services Research, 10(1) // 2. Ansari, Z. (2007). A Review of Literature on Access to Primary Health Care. Australian Journal of Primary Health, 13(2), 80.
3	684	Diagnosis and treatment - primary care: Initial laboratory investigations for hypertension	Process	Chronic	Diagnosis / Treatment	Effective	K - Cardiovascular	Diagnosis and treatment - primary care: Initial laboratory investigations for hypertension	1. Kringos, D. S., Boerma, W. G., Hutchinson, A., van der Zee, J., & Groenewegen, P. P. (2010). The breadth of primary care: a systematic literature review of its core dimensions. BMC Health Services Research, 10(1) // 2. Marshall M, Klazinga N, Leatherman S, Hardy C, Bergmann E, Pisco L, et al: OECD Health Care Quality Indicator Project. The expert panel on primary care prevention and health promotion. Int J Qual Health Care 2006, 18(Suppl 1):21-25.
18	685	Breast cancer screening for women	Process	Chronic	Screening and prevention	Effective	X - Female genital	Breast cancer screening for women	1. Kronenberg C, Doran T, Goddard M, et al. Identifying primary care quality indicators for people with serious mental illness: a systematic review. Br J Gen Pract. 2017;67(661):e519–e530. doi:10.3399/bjgp17X691721 // 2. Agency for Healthcare Research and Quality. AHRQ — quality indicators. AHRQ, 2016. www.qualityindicators.ahrq.gov (accessed 23 May 2017).
18	686	Colorectal cancer screening	Process	Chronic	Screening and prevention	Effective	D - Digestive	Colorectal cancer screening	1. Kronenberg C, Doran T, Goddard M, et al. Identifying primary care quality indicators for people with serious mental illness: a systematic review. Br J Gen Pract. 2017;67(661):e519–e530. doi:10.3399/bjgp17X691721 // 2. Agency for Healthcare Research and Quality. AHRQ — quality indicators. AHRQ, 2016. www.qualityindicators.ahrq.gov (accessed 23 May 2017).
24	687	Child healthcare in general practice	Process	Chronic	All	Patient-centered	A - General and unspecified	Child healthcare in general practice	1. Fujita, K., Moles, R. J., & Chen, T. F. (2018). Quality indicators for responsible use of medicines: a systematic review. BMJ Open, 8(7), e020437. // 2. Ruiz-Canela-Cáceres J, Aquino-Llinares N, Sánchez-Diaz JM, et al. Indicators for childhood asthma in Spain, using the Rand method. Allergol Immunopathol 2015;43:147–56.
24	688	Prescribing for children in PHC	Process	Chronic	Treatment	Effective	A - General and unspecified	Indicators for prescribing for children in PHC	1. Fujita, K., Moles, R. J., & Chen, T. F. (2018). Quality indicators for responsible use of medicines: a systematic review. BMJ Open, 8(7), e020437. // 2. 126. Barry E, O'Brien K, Moriarty F, et al. PIPc study: development of indicators of potentially inappropriate prescribing in children (PIPc) in primary care using a modified Delphi technique. BMJ Open 2016;6:e012079.
24	689	Preventing drug-related morbidity in PHC	Process	Chronic	Treatment	Safe	A - General and unspecified	Indicators for Preventing drug-related morbidity in PHC	1. Fujita, K., Moles, R. J., & Chen, T. F. (2018). Quality indicators for responsible use of medicines: a systematic review. BMJ Open, 8(7), e020437. // 2. 141. Morris CJ, Cantrill JA. Preventing drug-related morbidity - the development of quality indicators. J Clin Pharm Ther 2003;28:295–305
24	690	Long term prescribing in PHC	Process	Chronic	Treatment	Effective	A - General and unspecified	Long term prescribing in PHC	1. Fujita, K., Moles, R. J., & Chen, T. F. (2018). Quality indicators for responsible use of medicines: a systematic review. BMJ Open, 8(7), e020437. // 2. Cantrill JA, Sibbald B, Buetow S. Indicators of the appropriateness of long-term prescribing in general practice in the United Kingdom: consensus development, face and content validity, feasibility, and reliability. Qual Health Care 1998;7:130–5.
24	691	Medications related indicators for PHC	Process	Chronic	Treatment	Effective	A - General and unspecified	Medications related indicators for PHC	1. Fujita, K., Moles, R. J., & Chen, T. F. (2018). Quality indicators for responsible use of medicines: a systematic review. BMJ Open, 8(7), e020437. //
25	692	Detection of Falls	Process	Preventive	Screening and prevention	Safe	A - General and unspecified	Detection of Falls	1. Chin WY, Lam CL, Lo SV. Quality of care of nurse-led and allied health personnel-led primary care clinics. Hong Kong medical journal = Xianggang yi xue za zhi. 2011;17(3):217-30. // 2. Gillespie LD, Robertson MC, Gillespie WJ, et al. Interventions for preventing falls in older people living in the community. Cochrane Database Syst Rev 2009;(2):CD007146. // 3. Chang JT, Ganz DA. Quality indicators for falls and mobility problems in vulnerable elders. J Am Geriatr Soc 2007;55 Suppl 2:S327-34. // 4. Hendriks MR, Bleijlevens MH, van Haastregt JC, et al. Lack of effectiveness of a multidisciplinary fall-prevention program in elderly people at risk: a randomized, controlled trial. J Am Geriatr Soc 2008;56:1390-7. // 5. Gates S, Fisher JD, Cooke MW, Carter YH, Lamb SE. Multifactorial assessment and targeted intervention for preventing falls and injuries among older people in community and emergency care settings: systematic review and meta-analysis. BMJ 2008;336:130-3.
25	693	Falls: Basic Fall History	Process	Preventive	Screening and prevention	Patient-centered	A - General and unspecified	Falls: Basic Fall History	1. Chin WY, Lam CL, Lo SV. Quality of care of nurse-led and allied health personnel-led primary care clinics. Hong Kong medical journal = Xianggang yi xue za zhi. 2011;17(3):217-30. // 2. Gillespie LD, Robertson MC, Gillespie WJ, et al. Interventions for preventing falls in older people living in the community. Cochrane Database Syst Rev 2009;(2):CD007146. // 3. Chang JT, Ganz DA. Quality indicators for falls and mobility problems in vulnerable elders. J Am Geriatr Soc 2007;55 Suppl 2:S327-34. // 4. Hendriks MR, Bleijlevens MH, van Haastregt JC, et al. Lack of effectiveness of a multidisciplinary fall-prevention program in elderly people at risk: a randomized, controlled trial. J Am Geriatr Soc 2008;56:1390-7. // 5. Gates S, Fisher JD, Cooke MW, Carter YH, Lamb SE. Multifactorial assessment and targeted intervention for preventing falls and injuries among older people in community and emergency care settings: systematic review and meta-analysis. BMJ 2008;336:130-3.
25	694	Falls: Orthostatic Vital Signs	Process	Preventive	Screening and prevention	Effective	A - General and unspecified	Falls: Orthostatic Vital Signs	1. Chin WY, Lam CL, Lo SV. Quality of care of nurse-led and allied health personnel-led primary care clinics. Hong Kong medical journal = Xianggang yi xue za zhi. 2011;17(3):217-30. // 2. Gillespie LD, Robertson MC, Gillespie WJ, et al. Interventions for preventing falls in older people living in the community. Cochrane Database Syst Rev 2009;(2):CD007146. // 3. Chang JT, Ganz DA. Quality indicators for falls and mobility problems in vulnerable elders. J Am Geriatr Soc 2007;55 Suppl 2:S327-34. // 4. Hendriks MR, Bleijlevens MH, van Haastregt JC, et al. Lack of effectiveness of a multidisciplinary fall-prevention program in elderly people at risk: a randomized, controlled trial. J Am Geriatr Soc 2008;56:1390-7. // 5. Gates S, Fisher JD, Cooke MW, Carter YH, Lamb SE. Multifactorial assessment and targeted intervention for preventing falls and injuries among older people in community and emergency care settings: systematic review and meta-analysis. BMJ 2008;336:130-3.
25	695	Falls: Visual Acuity Testing	Process	Preventive	Screening and prevention	Effective	F - Eye	Falls: Visual Acuity Testing	1. Chin WY, Lam CL, Lo SV. Quality of care of nurse-led and allied health personnel-led primary care clinics. Hong Kong medical journal = Xianggang yi xue za zhi. 2011;17(3):217-30. // 2. Gillespie LD, Robertson MC, Gillespie WJ, et al. Interventions for preventing falls in older people living in the community. Cochrane Database Syst Rev 2009;(2):CD007146. // 3. Chang JT, Ganz DA. Quality indicators for falls and mobility problems in vulnerable elders. J Am Geriatr Soc 2007;55 Suppl 2:S327-34. // 4. Hendriks MR, Bleijlevens MH, van Haastregt JC, et al. Lack of effectiveness of a multidisciplinary fall-prevention program in elderly people at risk: a randomized, controlled trial. J Am Geriatr Soc 2008;56:1390-7. // 5. Gates S, Fisher JD, Cooke MW, Carter YH, Lamb SE. Multifactorial assessment and targeted intervention for preventing falls and injuries among older people in community and emergency care settings: systematic review and meta-analysis. BMJ 2008;336:130-3.

25	696	Gait and Balance Evaluation for Falls and Mobility Disorders	Process	Preventive	Screening and prevention	Effective	N - Neurological	Gait and Balance Evaluation for Falls and Mobility Disorders	1. Chin WY, Lam CL, Lo SV. Quality of care of nurse-led and allied health personnel-led primary care clinics. Hong Kong medical journal = Xianggang yi xue za zhi. 2011;17(3):217-30. // 2. Gillespie LD, Robertson MC, Gillespie WJ, et al. Interventions for preventing falls in older people living in the community. Cochrane Database Syst Rev 2009;(2):CD007146. // 3. Chang JT, Ganz DA. Quality indicators for falls and mobility problems in vulnerable elders. J Am Geriatr Soc 2007;55 Suppl 2:S327-34. // 4. Hendriks MR, Bleijlevens MH, van Haastregt JC, et al. Lack of effectiveness of a multidisciplinary fall-prevention program in elderly people at risk: a randomized, controlled trial. J Am Geriatr Soc 2008;56:1390-7. // 5. Gates S, Fisher JD, Cooke MW, Carter YH, Lamb SE. Multifactorial assessment and targeted intervention for preventing falls and injuries among older people in community and emergency care settings: systematic review and meta-analysis. BMJ 2008;336:130-3.
25	697	Falls: Cognitive Assessment	Process	Preventive	Screening and prevention	Effective	P - Psychological	Falls: Cognitive Assessment	1. Chin WY, Lam CL, Lo SV. Quality of care of nurse-led and allied health personnel-led primary care clinics. Hong Kong medical journal = Xianggang yi xue za zhi. 2011;17(3):217-30. // 2. Gillespie LD, Robertson MC, Gillespie WJ, et al. Interventions for preventing falls in older people living in the community. Cochrane Database Syst Rev 2009;(2):CD007146. // 3. Chang JT, Ganz DA. Quality indicators for falls and mobility problems in vulnerable elders. J Am Geriatr Soc 2007;55 Suppl 2:S327-34. // 4. Hendriks MR, Bleijlevens MH, van Haastregt JC, et al. Lack of effectiveness of a multidisciplinary fall-prevention program in elderly people at risk: a randomized, controlled trial. J Am Geriatr Soc 2008;56:1390-7. // 5. Gates S, Fisher JD, Cooke MW, Carter YH, Lamb SE. Multifactorial assessment and targeted intervention for preventing falls and injuries among older people in community and emergency care settings: systematic review and meta-analysis. BMJ 2008;336:130-3.
25	698	Falls: Home Hazard Assessment and Modification	Process	Preventive	Screening and prevention	Safe	A - General and unspecified	Falls: Home Hazard Assessment and Modification	1. Chin WY, Lam CL, Lo SV. Quality of care of nurse-led and allied health personnel-led primary care clinics. Hong Kong medical journal = Xianggang yi xue za zhi. 2011;17(3):217-30. // 2. Gillespie LD, Robertson MC, Gillespie WJ, et al. Interventions for preventing falls in older people living in the community. Cochrane Database Syst Rev 2009;(2):CD007146. // 3. Chang JT, Ganz DA. Quality indicators for falls and mobility problems in vulnerable elders. J Am Geriatr Soc 2007;55 Suppl 2:S327-34. // 4. Hendriks MR, Bleijlevens MH, van Haastregt JC, et al. Lack of effectiveness of a multidisciplinary fall-prevention program in elderly people at risk: a randomized, controlled trial. J Am Geriatr Soc 2008;56:1390-7. // 5. Gates S, Fisher JD, Cooke MW, Carter YH, Lamb SE. Multifactorial assessment and targeted intervention for preventing falls and injuries among older people in community and emergency care settings: systematic review and meta-analysis. BMJ 2008;336:130-3.
25	699	Falls: Benzodiazepine Discontinuation	Process	Preventive	Screening and prevention	Safe	P - Psychological	Falls: Benzodiazepine Discontinuation	1. Chin WY, Lam CL, Lo SV. Quality of care of nurse-led and allied health personnel-led primary care clinics. Hong Kong medical journal = Xianggang yi xue za zhi. 2011;17(3):217-30. // 2. Gillespie LD, Robertson MC, Gillespie WJ, et al. Interventions for preventing falls in older people living in the community. Cochrane Database Syst Rev 2009;(2):CD007146. // 3. Chang JT, Ganz DA. Quality indicators for falls and mobility problems in vulnerable elders. J Am Geriatr Soc 2007;55 Suppl 2:S327-34. // 4. Hendriks MR, Bleijlevens MH, van Haastregt JC, et al. Lack of effectiveness of a multidisciplinary fall-prevention program in elderly people at risk: a randomized, controlled trial. J Am Geriatr Soc 2008;56:1390-7. // 5. Gates S, Fisher JD, Cooke MW, Carter YH, Lamb SE. Multifactorial assessment and targeted intervention for preventing falls and injuries among older people in community and emergency care settings: systematic review and meta-analysis. BMJ 2008;336:130-3.
25	700	Falls: Assistive Device	Process	Preventive	Screening and prevention	Effective	A - General and unspecified	Falls: Assistive Device	1. Chin WY, Lam CL, Lo SV. Quality of care of nurse-led and allied health personnel-led primary care clinics. Hong Kong medical journal = Xianggang yi xue za zhi. 2011;17(3):217-30. // 2. Gillespie LD, Robertson MC, Gillespie WJ, et al. Interventions for preventing falls in older people living in the community. Cochrane Database Syst Rev 2009;(2):CD007146. // 3. Chang JT, Ganz DA. Quality indicators for falls and mobility problems in vulnerable elders. J Am Geriatr Soc 2007;55 Suppl 2:S327-34. // 4. Hendriks MR, Bleijlevens MH, van Haastregt JC, et al. Lack of effectiveness of a multidisciplinary fall-prevention program in elderly people at risk: a randomized, controlled trial. J Am Geriatr Soc 2008;56:1390-7. // 5. Gates S, Fisher JD, Cooke MW, Carter YH, Lamb SE. Multifactorial assessment and targeted intervention for preventing falls and injuries among older people in community and emergency care settings: systematic review and meta-analysis. BMJ 2008;336:130-3.
25	701	Falls: Exercise Programs	Process	Preventive	Screening and prevention	Effective	A - General and unspecified	Falls: Exercise Programs	1. Chin WY, Lam CL, Lo SV. Quality of care of nurse-led and allied health personnel-led primary care clinics. Hong Kong medical journal = Xianggang yi xue za zhi. 2011;17(3):217-30. // 2. Gillespie LD, Robertson MC, Gillespie WJ, et al. Interventions for preventing falls in older people living in the community. Cochrane Database Syst Rev 2009;(2):CD007146. // 3. Chang JT, Ganz DA. Quality indicators for falls and mobility problems in vulnerable elders. J Am Geriatr Soc 2007;55 Suppl 2:S327-34. // 4. Hendriks MR, Bleijlevens MH, van Haastregt JC, et al. Lack of effectiveness of a multidisciplinary fall-prevention program in elderly people at risk: a randomized, controlled trial. J Am Geriatr Soc 2008;56:1390-7. // 5. Gates S, Fisher JD, Cooke MW, Carter YH, Lamb SE. Multifactorial assessment and targeted intervention for preventing falls and injuries among older people in community and emergency care settings: systematic review and meta-analysis. BMJ 2008;336:130-3.
25	702	Medication review and pharmaceutical care	Process	Preventive	Follow up and continuity	Safe / Patient-centered	A - General and unspecified	Medication review and pharmaceutical care	1. Chin WY, Lam CL, Lo SV. Quality of care of nurse-led and allied health personnel-led primary care clinics. Hong Kong medical journal = Xianggang yi xue za zhi. 2011;17(3):217-30. // 2. Knight EL, Avorn J. Quality indicators for appropriate medication use in vulnerable elders. Ann Intern Med 2001;135:703-10. // 3. RESPECT trial team. Effectiveness of shared pharmaceutical care for older patients: RESPECT trial findings. Br J Gen Pract 2010;60:e10-9. // 4. Mackie CA, Lawson DH, Campbell A, Maclaren AG, Waigh R. A randomised controlled trial of medication review in patients receiving polypharmacy in general practice. Pharm J 1999;263:R7 // 5. Holland R, Smith R, Harvey I. Where now for pharmacist led medication review? J Epidemiol Community Health 2006;60:92-3. // 6. Mackie CA, Lawson DH, Campbell A, Maclaren AG, Waigh R. A randomised controlled trial of medication review in patients receiving polypharmacy in general practice. Pharm J 1999;263:R7 .
25	703	Pulmonary rehabilitation	Process	Chronic	Follow up and continuity	Effective	R - Respiratory	% of Patients with clinically significant COPD that had access to pulmonary rehabilitation	1. Chin WY, Lam CL, Lo SV. Quality of care of nurse-led and allied health personnel-led primary care clinics. Hong Kong medical journal = Xianggang yi xue za zhi. 2011;17(3):217-30. // 2. Ambrosino N, Casaburi R, Ford G, et al. Developing concepts in the pulmonary rehabilitation of COPD. Respir Med 2008;102 Suppl 1:S17-26. // 3. Nici L, Donner C, Wouters E, et al. American Thoracic Society/European Respiratory Society statement on pulmonary rehabilitation. Am J Respir Crit Care Med 2006;173:1390-413. // 4. Lacasse Y, Martin S, Lasserson TJ, Goldstein RS. Metaanalysis of respiratory rehabilitation in chronic obstructive pulmonary disease. A Cochrane systematic review. Eura Medicophys 2007;43:475-85.
27	704	Availability of telephone triage and advice services	Process	Preventive	All	Timely	A - General and unspecified	Changes in health service utilisation brought about by the availability of telephone triage and advice services (reduction of General Practice consultations)	1. Lake R, Georgiou A, Li J, et al. The quality, safety and governance of telephone triage and advice services - an overview of evidence from systematic reviews. BMC Health Serv Res. 2017;17(1):614. Published 2017 Aug 30. doi:10.1186/s12913-017-2564-x
27	705	Frequency of adverse events, errors and hospitalisation rates	Outcome	Preventive	Treatment	Safe	A - General and unspecified	Frequency of adverse events, errors and hospitalisation rates	1. Lake R, Georgiou A, Li J, et al. The quality, safety and governance of telephone triage and advice services - an overview of evidence from systematic reviews. BMC Health Serv Res. 2017;17(1):614. Published 2017 Aug 30. doi:10.1186/s12913-017-2564-x
27	706	Number of deaths in seven days between those whose calls were handled by doctors or nurses	Outcome	Preventive	Treatment	Effective	A - General and unspecified	Number of deaths in seven days between those whose calls were handled by doctors or nurses	1. Lake R, Georgiou A, Li J, et al. The quality, safety and governance of telephone triage and advice services - an overview of evidence from systematic reviews. BMC Health Serv Res. 2017;17(1):614. Published 2017 Aug 30. doi:10.1186/s12913-017-2564-x
27	707	Patient compliance to advice given to seek emergency care	Process	Acute	Follow up and continuity	Effective	A - General and unspecified	Patient compliance to advice given to seek emergency care	1. Lake R, Georgiou A, Li J, et al. The quality, safety and governance of telephone triage and advice services - an overview of evidence from systematic reviews. BMC Health Serv Res. 2017;17(1):614. Published 2017 Aug 30. doi:10.1186/s12913-017-2564-x // 2. Purc-Stephenson RJ, Thrasher C. Patient compliance with telephone triage recommendations: a meta-analytic review. Patient Educ Couns. 2012;87(2):135–42. // 3. Carrasqueiro S, Oliveira M, Encarnação P. Evaluation of telephone triage and advice services: a systematic review on methods, metrics and results. Stud Health Technol Inform. 2011;169:407–11. // 4. Blank L, Coster J, O'Cathain A, Knowles E, Tosh J, Turner J, Nicholl J. The appropriateness of, and compliance with, telephone triage decisions: a systematic review and narrative synthesis. J Adv Nurs. 2012;68(12):2610–21.
27	708	Patient compliance to advice given to seek GP	Process	All	Follow up and continuity	Effective	A - General and unspecified	Patient compliance to advice given to seek GP	1. Lake R, Georgiou A, Li J, et al. The quality, safety and governance of telephone triage and advice services - an overview of evidence from systematic reviews. BMC Health Serv Res. 2017;17(1):614. Published 2017 Aug 30. doi:10.1186/s12913-017-2564-x // 2. Purc-Stephenson RJ, Thrasher C. Patient compliance with telephone triage recommendations: a meta-analytic review. Patient Educ Couns. 2012;87(2):135–42. // 3. Carrasqueiro S, Oliveira M, Encarnação P. Evaluation of telephone triage and advice services: a systematic review on methods, metrics and results. Stud Health Technol Inform. 2011;169:407–11. // 4. Blank L, Coster J, O'Cathain A, Knowles E, Tosh J, Turner J, Nicholl J. The appropriateness of, and compliance with, telephone triage decisions: a systematic review and narrative synthesis. J Adv Nurs. 2012;68(12):2610–21.
27	709	Patient satisfaction of telephone triage and advice services	Outcome	Preventive	Follow up and continuity	Patient-centered	A - General and unspecified	Patient satisfaction of telephone triage and advice services	1. Lake R, Georgiou A, Li J, et al. The quality, safety and governance of telephone triage and advice services - an overview of evidence from systematic reviews. BMC Health Serv Res. 2017;17(1):614. Published 2017 Aug 30. doi:10.1186/s12913-017-2564-x // 2. Leibowitz R, Day S, Dunt D. A systematic review of the effect of different models of after-hours primary medical care services on clinical outcome, medical workload, and patient and GP satisfaction. Fam Pract. 2003;20(3):311–7.
27	710	Percentage of calls able to be handled with telephone advice alone	Process	Preventive	Follow up and continuity	Efficient	A - General and unspecified	Percentage of calls able to be handled with telephone advice alone	1. Lake R, Georgiou A, Li J, et al. The quality, safety and governance of telephone triage and advice services - an overview of evidence from systematic reviews. BMC Health Serv Res. 2017;17(1):614. Published 2017 Aug 30. doi:10.1186/s12913-017-2564-x
27	711	Reduction in hospital admissions	Outcome	Preventive	Follow up and continuity	Efficient	A - General and unspecified	Number of phone service run leading to a reduction in admissions at 12 months	1. Lake R, Georgiou A, Li J, et al. The quality, safety and governance of telephone triage and advice services - an overview of evidence from systematic reviews. BMC Health Serv Res. 2017;17(1):614. Published 2017 Aug 30. doi:10.1186/s12913-017-2564-x
3 (25)	712	Patient satisfaction with the family physician/specialist coordination of care	Outcome	Chronic	Follow up and continuity	Patient-centered	Not Defined	By the use of patient questionnaire, assess patient satisfaction with the coordination of care provide by the GP/family physisican/specialist	1. Kringos, D. S., Boerma, W. G., Hutchinson, A., van der Zee, J., & Groenewegen, P. P. (2010). The breadth of primary care: a systematic literature review of its core dimensions. BMC Health Services Research, 10(1) // Gene-Badia J, Ascaso C, Escaramis-Babiano G, Sampietro-Colom L, Catalan- Ramos A, Sans-Corrales M, et al: Personalised care, access, quality and team coordination are the main dimensions of family medicine output Fam Pract 2007, 24:41-47.
11	713	Adherence to Asthma Medications	Process	Chronic	Treatment	Effective	R - Respiratory	Adherence to Asthma Medications	To, T., Guttmann, A., Lougheed, M. D., Gershon, A. S., Dell, S. D., Stanbrook, M. B., … Fisman, D. N. (2010). Evidence-based performance indicators of primary care for asthma: a modified RAND Appropriateness Method. International Journal for Quality in Health Care, 22(6), 476–485.
29	714	Percentage of patients with coronary heart disease who have had influenza immunisation	Outcome	Preventive	Screening and prevention	Effective	K - Cardiovascular	The percentage of patients with coronary heart disease who have had influenza immunisation in the preceding 1 August to 31 March	Forbes, L. J., Marchand, C., Doran, T., & Peckham, S. (2017). The role of the Quality and Outcomes Framework in the care of long-term conditions: a systematic review. British Journal of General Practice, 67(664), e775–e784. // current recommendation from the Chief Medical Officer (CMO) and the Joint Committee on Vaccination and Immunisation (JCVI). Free seasonal influenza vaccine is funded for several groups at higher risk of complications from influenza including all individuals aged 5 years and over with medical risk conditions, namely: cardiac disease, including cyanotic congenital heart disease, coronary artery disease and congestive heart failure chronic respiratory conditions, including suppurative lung disease, chronic obstructive pulmonary disease and severe asthma other chronic illnesses requiring regular medical follow up or hospitalisation in the previous year, including diabetes mellitus, chronic metabolic diseases, chronic renal failure, and haemoglobinopathies chronic neurological conditions that impact on respiratory function, including multiple sclerosis, spinal cord injuries, and seizure disorders impaired immunity, including HIV, malignancy and chronic steroid use children aged 6 months to 10 years on long term aspirin therapy.all people aged 65 years and over.
29	715	Percentage of patients with diabetes who have had influenza immunisation	Outcome	Preventive	Screening and prevention	Effective	T - Endocrine/Metabolic and Nutritional	The percentage of patients with diabetes, on the register, who have had influenza immunisation in the preceding 1 August to 31 March	This is a current recommendation from the CMO and the JCVI. (diabetes is one of the risk groups covered with free seasonal influenza vaccine). The burden of influenza falls mostly on those who have clinical risk factors for influenza and older people and most especially on older people with clinical risk factors. These are the groups currently targeted for annual influenza vaccination. The current seasonal influenza programme is highly likely to be cost effective compared with no vaccination, particularly when considered over a number years, but for some individual years there may be little benefit to vaccination when the influenza season is mild, or the vaccine is not well-matched to the prevalent strains. Cost effectiveness is sensitive to estimates of the number of influenza-related deaths and by the number of influenza-related deaths that may be prevented by vaccination. //Data suggested that increasing uptake to 75% in clinical risk groups within the current vaccination programme would be beneficial.// The HPA study provided further evidence that those with clinical risk factors are at greatly increased risk of hospitalisation and death from influenza and there would be significant additional benefit from increasing vaccine uptake to 75% in those with clinical risk factors and aged below 65 years. Therefore, the committee advised that increasing vaccine uptake in clinical risk groups should remain the priority in order that those at greatest risk of influenza receive direct protection from vaccination. As increased vaccine uptake in clinical risk groups would influence the cost effectiveness of extensions to the programme, further analyses would be required to establish the cost effectiveness of current programme at a level of 75% vaccine uptake in clinical risk groups and then review the incremental cost effectiveness of extending the programme to age groups of children.
29	716	Percentage of patients with STIA who have had influenza immunisation	Outcome	Preventive	Screening and prevention	Effective	K - Cardiovascular	The percentage of patients with stroke or TIA who have had influenza immunisation in the preceding 1 August to 31 March	While there have been no RCTs looking at the impact of flu vaccination specifically in patients with a history of stroke or TIA, there is evidence from observation studies that flu vaccination reduces risk of stroke52
24	717	Percentage of penicillin prescriptions in dental treatments	Process	Acute / Preventive	Treatment	Effective	D - Digestive	Beta-lactamase sensitive penicillins (ATC code: J01C), such as phenoxymethylpenicillin or amoxicillin are considered the first line antibacterial medications in dentistry for patients without penicillin allergy due to their effectiveness against oral bacterial infections. Penicillins are more effective and/or have less side effects as well as result in less health complications compared to other antibiotics, e.g. cephalosporin or clindamycin	Hussein RJ, Krohn R, Kaufmann-Kolle P, et al. Quality indicators for the use of systemic antibiotics in dentistry. Z Evid Fortbild Qual Gesundhwes 2017;122:1–8.
24	718	Percentage of clindamycin prescriptions in dental treatments	Process	Preventive	Treatment	Effective	D - Digestive	Clindamycin is not a specific antibiotic for orofacial infections and it can be used for the treatment of various bacterial infections such as those of bone and joints as well as infections of the respiratory system. Clindamycin has more side effects and health complications compared to penicillin	Hussein RJ, Krohn R, Kaufmann-Kolle P, et al. Quality indicators for the use of systemic antibiotics in dentistry. Z Evid Fortbild Qual Gesundhwes 2017;122:1–8.
3	719	Health care funding system	Structure	All	All	All	Not Defined		Kringos DS, Boerma WG, Hutchinson A, van der Zee 465 J, Groenewegen PP. The breadth of primary care: a systematic literature review of its core dimensions. 467 BMC Health Serv Res. 2010;10:65. Published 2010 Mar 13. doi:10.1186/1472-468 6963-10-83
31(3)	720	Percentage of First choice drugs (e.g., enalapril or simvastatin) of all drugs prescribed within its therapeutic class (angiotensin-converting-enzyme inhibitors or lipid lowering drugs).	Process	Chronic	Treatment	Effective	K - Cardiovascular	number of different brands per active agent	Martirosyan L, Voorham J, Haaijer-Ruskamp FM, Braspenning J, Wolffenbuttel BH, Denig P. A systematic literature review: prescribing indicators related to type 2 diabetes mellitus and cardiovascular risk management. Pharmacoepidemiology and drug safety. 2010;19(4):319-34.
31(16)	721		Process	Chronic	Treatment	Effective	K - Cardiovascular	Proportion of atorvastatin and cerivastatin	Martirosyan L, Voorham J, Haaijer-Ruskamp FM, Braspenning J, Wolffenbuttel BH, Denig P. A systematic literature review: prescribing indicators related to type 2 diabetes mellitus and cardiovascular risk management. Pharmacoepidemiology and drug safety. 2010;19(4):319-34.
31(43)	722		Process	Chronic	Treatment	Effective	K - Cardiovascular	DDD senalapril and captopril divided by DDDs of all ACE inhibitors · 100%	Martirosyan L, Voorham J, Haaijer-Ruskamp FM, Braspenning J, Wolffenbuttel BH, Denig P. A systematic literature review: prescribing indicators related to type 2 diabetes mellitus and cardiovascular risk management. Pharmacoepidemiology and drug safety. 2010;19(4):319-34.
31(47)	723		Process	Chronic	Treatment	Effective	K - Cardiovascular	DDD estatinas de elección/DDD estatinas	Martirosyan L, Voorham J, Haaijer-Ruskamp FM, Braspenning J, Wolffenbuttel BH, Denig P. A systematic literature review: prescribing indicators related to type 2 diabetes mellitus and cardiovascular risk management. Pharmacoepidemiology and drug safety. 2010;19(4):319-34.
31(59)	724		Process	Chronic	Treatment	Effective	K - Cardiovascular	Porcentaje de estatinas para las que se había demostrado una disminución de morbimortalidad cardiovascular (simvastatina, pravastatina y lovastatina)/total de estatinas	Martirosyan L, Voorham J, Haaijer-Ruskamp FM, Braspenning J, Wolffenbuttel BH, Denig P. A systematic literature review: prescribing indicators related to type 2 diabetes mellitus and cardiovascular risk management. Pharmacoepidemiology and drug safety. 2010;19(4):319-34.
31(3)	725	Precentage of prescribed generic drugs	Process	Chronic	Treatment	Efficient	A - General and unspecified	Percentage of generic prescribing	Martirosyan L, Voorham J, Haaijer-Ruskamp FM, Braspenning J, Wolffenbuttel BH, Denig P. A systematic literature review: prescribing indicators related to type 2 diabetes mellitus and cardiovascular risk management. Pharmacoepidemiology and drug safety. 2010;19(4):319-34.
31 (19,47)	726	Prescribe more than 1 drug from the same therapeutic group simultaneously	Process	Chronic	Treatment	Effective	A - General and unspecified	Patients prescribed more than one thiazide diuretic (of those prescribed a thiazide diuretic); Patients prescribed more than one sulphonylurea hypoglycaemic (of those prescribed a sulphonylurea hypoglycaemic) [31(19)]; (% pacientes en prevención secundaria cardiovascular tratados con estatinas [31(47)]?)	Martirosyan L, Voorham J, Haaijer-Ruskamp FM, Braspenning J, Wolffenbuttel BH, Denig P. A systematic literature review: prescribing indicators related to type 2 diabetes mellitus and cardiovascular risk management. Pharmacoepidemiology and drug safety. 2010;19(4):319-34.
31(10)	727	Ratio of preferred: less preferred drugs (e.g., plain:combination diuretics)	Process	Chronic	Treatment	Safe	A - General and unspecified	plain' xombination diuretic ratio	Martirosyan L, Voorham J, Haaijer-Ruskamp FM, Braspenning J, Wolffenbuttel BH, Denig P. A systematic literature review: prescribing indicators related to type 2 diabetes mellitus and cardiovascular risk management. Pharmacoepidemiology and drug safety. 2010;19(4):319-34.
